# ﻿The Leptophlebiidae of New Guinea (Ephemeroptera, Choroterpinae, Thraulini)

**DOI:** 10.3897/zookeys.1245.141723

**Published:** 2025-07-15

**Authors:** Michel Sartori, Frederico F. Salles

**Affiliations:** 1 Naturéum, Department of zoology, Palais de Rumine, Place Riponne 6, CH-1005 Lausanne, Switzerland Naturéum, Department of zoology Lausanne Switzerland; 2 Department of Ecology and Evolution, Biophore, University of Lausanne, CH-1015 Lausanne, Switzerland University of Lausanne Lausanne Switzerland; 3 Museu de Entomologia, Departamento de Entomologia, Universidade Federal de Viçosa, Viçosa, Brazil Universidade Federal de Viçosa Viçosa Brazil

**Keywords:** Biogeography, Indonesia Papua, new genus, new species, Papua New Guinea, Seram, taxonomy, Timor

## Abstract

The first global account of the diversity of the Ephemeroptera family Leptophlebiidae in New Guinea is provided, based on collections of nymphs collected throughout the island and in some neighboring islands by Michael Balke (Munich) and his collaborators mainly between 2006 and 2011. All specimens belong to the subfamily Choroterpinae and to the tribe Thraulini. All species can be accommodated in three genera: *Thraulus* Eaton, 1881, *Nonnullidens* Grant & Peters, 1993, and *Kosminympha***gen. nov.** The genus *Thraulus* is divided into two subgenera, namely *Thraulus* and *Masharikella* Peters, Gilles & Edmunds, 1964, **stat. nov.** which is removed from synonymy with *Thraulus*. Thirty-one new species are described herein. The subgenus Thraulus is reported with nine new species: Thraulus (Thraulus) eloisae**sp. nov.**, Thraulus (Thraulus) granti**sp. nov.**, Thraulus (Thraulus) longinquus**sp. nov.**, Thraulus (Thraulus) nabire**sp. nov**., Thraulus (Thraulus) noe**sp. nov.**, Thraulus (Thraulus) ogea**sp. nov.**, Thraulus (Thraulus) timorensis**sp. nov**., Thraulus (Thraulus) wemale**sp. nov**., and Thraulus (Thraulus) sp. A. Five new species of the subgenus Masharikella are described: Thraulus (Masharikella) iteris**sp. nov.**, Thraulus (Masharikella) johannisluci**sp. nov**., Thraulus (Masharikella) pascalae**sp. nov.**, Thraulus (Masharikella) samueli**sp. nov**., and Thraulus (Masharikella) sp. B. Besides the seven known species placed in the genus *Nonnullidens*, 11 new species are described: *Nonnullidensalvarezi***sp. nov**., *Nonnullidensanga***sp. nov.**, *Nonnullidensboonsoongi***sp. nov**., *Nonnullidenscozzarolae***sp. nov.**, *Nonnullidensfuyugensis***sp. nov**., *Nonnullidenskaltenbachi***sp. nov.**, *Nonnullidensmarcelae***sp. nov.**, *Nonnullidensmoiorum***sp. nov**., *Nonnullidenssilvaepumilorum***sp. nov**., and two unnamed species. Similar to *Nonnullidens*, the new genus *Kosminympha***gen. nov.** is endemic to New Guinea and is currently restricted to the eastern part of Papua New Guinea. It is composed of six new species: *Kosminymphaasarorum***sp. nov**., *Kosminymphabalkei***sp. nov**., *Kosminymphabaruya***sp. nov.**, *Kosminymphakalamorum***sp. nov.**, *Kosminymphapaulinae***sp. nov.**, and *Kosminymphasabrinae***sp. nov.***Nonnullidensmariae* (Peters & Tsui, 1972), the type species of the genus *Barba* Grant & Peters, 1993 is redescribed, and the synonymy of *Barba* with *Nonnullidens* is followed but shown to be not completely satisfactory. A key for all currently known species of leptophlebiid nymphs occurring in the area is provided. Compared to the work of Cozzarolo et al. (2019) who investigated the genetic diversity of this fauna, our results appear to be more conservative, some molecular operational taxonomic units (MOTUs) being lumped together based on morphology. The affinities of this fauna with neighbouring areas of Southeast Asia and Australia are also discussed. Although this study reveals a large part of the hidden biodiversity of New Guinea, it is far from being complete and a huge number of new species of Leptophlebiidae await formal description.

“It is certainly a wonderful and unexpected fact, that an accurate knowledge of the distribution of birds and insects should enable us to map out lands and continents which disappeared beneath the ocean long before the earliest traditions of the human race.”

A.R. Wallace, The Malay Archipelago, 1869, p. 27

## ﻿Introduction

The family Leptophlebiidae is one of the most diversified families of mayflies around the world. It is present worldwide, except on some oceanic islands, and encompasses approximately 800 species shared within more than 150 genera (Jacobus et al. 2019; personal dataset of authors). A recent study by Cozzarolo et al. (2019) showed that Leptophlebiidae found in Papua New Guinea form a monophyletic group, called the *Thraulus* lineage. This lineage, or the tribe Thraulini Kluge, 2012, belongs to the subfamily Choroterpinae Kluge, 2012, and is composed currently of the genera *Thraulus* Eaton, 1881 and *Nonnullidens* Grant & Peters, 1993 (Monjardim et al. 2020). Another genus, *Sangpradubina* Boonsoong & Sartori, 2016 presents a nymphal stage similar to Thraulini and an adult stage similar to Choroterpini Kluge, 2012; despite superficial similarities in the gill morphology, *Sangpradubina* exhibits important unique characters (e.g., fore- and mid-femora with a dense row of long setae on outer margin, single filamentous gill I, ventral lamellae of gills II–VII larger than dorsal one, cerci and paracercus with whorls of thin setae), which suggests it could belong to a separate tribe in the subfamily (Boonsoong and Sartori 2016).

The genus *Thraulus* is by far the most diversified, with approximately 21 known species in the Oriental, Palearctic, and Afrotropical regions, until a recent monograph by Grant (2024) revising all known species together with the description of nine new species brought the diversity of the genus *Thraulus* to 30 species. In contrast, the genus *Nonnullidens* contains seven species, all restricted to Papua New Guinea (Kluge 2013). The presence of species belonging to *Thraulus* in Papua New Guinea is nevertheless attested by Peters and Tsui (1972) and Cozzarolo et al. (2019) but remained unstudied until now. Broadly, both genera can be identified by their gill morphology at the nymphal stage; *Thraulus* species possess gills on segments II–VII composed of two lamellae entirely fringed with numerous filaments, whereas gills on the first abdominal segment is different in shape, composed of a slender bifid (rarely single) filament; *Nonnullidens* nymphs possess gills I–VII alike, composed of two lamellae (rarely one) which are fringed with filaments only at the apex of the lamellae. Both genera are hardly distinguishable at adult stages, as no unique autapomorphy can be found (Kluge 2013).

The taxonomy of these two genera has been the subject to some changes over time. First, Peters et al. (1964) proposed the genus *Masharikella* for three species from Africa, previously placed in the Neotropical genus *Hagenulus* Eaton, 1882; *M.fasciata* (Kimmins, 1956) from Uganda and the shores of Lake Tanganyika, *M.torrentis* Gillies (in Peters et al. 1964) from Usambara Mountains in Tanzania, and *M.turbinata* (Ulmer, 1909) from Comoros Islands, together with the Oriental species *M.semicastanea* (Gillies, 1951) reported from India, but for which nymph remains unknown. The nymph of *Masharikella* is characterised by gills II–VII similar to *Thraulus*, whereas gill I is composed of a dorsal slender and lanceolate lamella, whereas the ventral lamella is ovate with fringed margins. Peters and Edmunds (1970) placed *Masharikella* in strict synonymy with *Thraulus*. Their justification was based on comparison with an unnamed group of *Thraulus*-like species from New Guinea (the genus *Nonnullidens* described later by Grant and Peters 1993), which introduce some overlapping in character distribution among these “three natural groups” (Peters and Edmunds 1970: 206–207). No reliable characters were found at the adult stages, and although the shape of gill I clearly separates the three groups, it was interpreted as physiological adaptation of oxygen content, thus without phylogenetic signal. In the same paper where they described *Nonnullidens*, Grant and Peters (1993) also described the genus *Barba*, which differed from *Nonnullidens* by the ventral lamella of gill II–VII less reduced and the tarsal claw with more denticles than in *Nonnullidens*. Both genera were put into synonymy by Kluge (2013) because no reliable characters to separate them could be found in imaginal stages.

**Figure 1.**
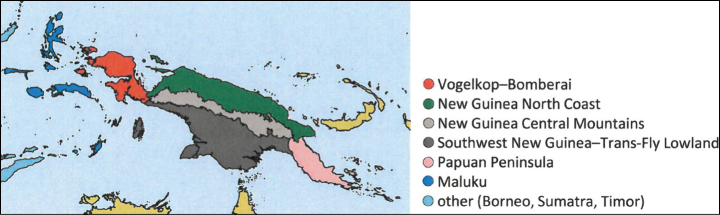
Freshwater ecoregions of New Guinea following Abell et al. (2008), redrawn from Cozzarolo et al. (2019).

*Thraulus* nymphs possessing a first pair of gills similar to *Masharikella* can be found in Africa, with the species *Thraulustorrentis* and *T.fasciatus* (see above), and in southern India, with *T.gopalani* Grant & Sivaramakrishnan, 1985, *T.malabarensis* Vasanth, Subramanian & Selvakumar, 2022, *T.thiagarajani* Balasubramanian & Muthukatturaja, 2019. They are also reported from Australia, where *T.opifer* Grant, 2024 (*Thraulus* sp. AV1 sensu Dean 1999) was described from Northern Territories, as well as two unnamed species from Queensland (*Thraulus* sp. AV2 and *Thraulus* sp. AV3) recorded by Dean (1999). Demoulin (1969) reported the presence of *Masharikelladuliti* (Demoulin, 1954) based on nymphs and imagos collected on Manus Island and New Britain; although the specific attribution is erroneous according to Grant and Peters (1993), this is the only other report of *Masharikella*-like *Thraulus* close to Papua New Guinea. The island of Papua New Guinea and surrounding small islets is a very complex landmass, which current shape and geography has been shaped by million years of tectonic plate movements and orogenesis events combined with fluctuations in climate and see level (Toussaint et al. 2014, 2021; Letsch et al. 2023). Therefore, different regions of the island have different geological history, leading to different freshwater ecoregions (Fig. 1; Abell et al. 2008). According to Cozzarolo et al. (2019) the *Thraulus* lineage appeared in the region during the Eocene (56–34 My BP), colonising the proto-Papuan archipelago. Subsequent geological events shaped drastically the island, among which the orogenesis of the central highlands played a major role in species diversification by allopatric processes (Toussaint et al. 2014). Papua New Guinea exhibits a rather depauperate mayfly fauna at the family and generic level, with only five families and fewer than 15 genera (Edmunds and Polhemus 1990). Nevertheless, some genera have been through rapid and important radiations, exhibiting a unique species diversity, such as *Labiobaetis* Novikova & Kluge, 1987, with 43 species (Kaltenbach et al. 2023). This trend is also reported for other aquatic insects, such as the water beetle genus *Exocelina* (Toussaint et al. 2014, 2021).

The aim of the present study is to morphologically investigate the material studied by Cozzarolo et al. (2019), to assess the validity of the current systematics, and to propose a first overview of the real diversity of the *Thraulus* lineage in Papua New Guinea and some surrounding islands.

## ﻿Materials and methods

All the specimens used in this study originate from the numerous field trips of Michael Balke (Munich, Germany) in Indonesia and New Guinea mainly between 2006 and 2011. Specimens included by Cozzarolo et al. (2019) were treated for genetic analysis according to Vuataz et al. (2011), meaning that the entire specimen was soaked in the buffer solution overnight, then removed and placed in 100% ethanol. Later, each specimen was placed in Cellosolve (2–ethoxyethanol), dissected, and mounted on slide with Euparal as medium. All specimens were spread into morphospecies which were tentatively aggregated into four morphogenera: *Thraulus*, *Masharikella*, *Nonnullidens*, and *Barba*. We placed into this last operational taxonomic unit (OTU) specimens which possess a first pair of gills similar to the subsequent ones, as for *Nonnullidens* but differ by their much larger size (8.0–12.5 mm, vs 4.5–7.5 mm in *Nonnullidens*), often colourful abdomen, and gills with a ventral lamella only slightly reduced compared to the dorsal one.

Nymphal habitus were photographed using a Canon EOS 6D camera and the Visionary Digital Passport imaging system (formerly available and distributed by Dun Inc., Virginia) and processed with Adobe Photoshop Lightroom and Helicon Focus version 5.3. Microscopic pictures were taken using an Olympus BX51 microscope coupled with an Olympus SC50 camera; photographs were enhanced with Olympus Stream Basic 2.3.2 stacking software and Adobe Photoshop Elements 2022 20.0.

The taxonomic descriptions and the key presented herein were generated with a DELTA (Dallwitz 1980, Dallwitz et al. 1999) database containing the morphological states of characters of Leptophlebiidae of New Guinea. Distribution maps were generated using Simple Mappr (Shorthouse 2010).

### ﻿Type material depository

All the studied material has been deposited in the following institutions:

**MZB**Museum Zoologicum Bogoriense (Indonesia)

**MZL**Naturéum, Department of zoology, Lausanne (Switzerland)

**UFVB**Museu de Entomologia, Viçosa, Viçosa (Brazil)

**ZSM**Zoologische Staatssammlung München (Germany)

Each occurrence (vial or slide) has been equipped by a unique QR code beginning with GBIFCH and followed by 8 digits; accession number for CO1 and 16S sequences in GenBank were retrieved from Cozzarolo et al. (2019) when available, and genetic sequence nomenclature follow Chakrabarty et al. (2013); all information is available in Table 1.

**Table 1.** Detailed information on the specimens used in this study.

Taxonomic name	Specimens	Identifier number	Genetic ID (Cozzarolo et al. 2019)	GenBank #		GenSeq nomenclature (Chakrabarty et al. 2013)	Altitude (m)
				CO1	16S		
Thraulus (Thraulus) eloisae	TH5_PNG87_2	GBIFCH01223089	sp. 52	MN038391	MN023306	genseq-2 CO1, 16S	1500–1700
	TH5_PNG87_1	GBIFCH01223087	sp. 52	MN038390	MN023233	genseq-1 CO1, 16S	
	TH3_PNG167_1	GBIFCH01223088	n/a	n/a	n/a	n/a	
	TH5_PNG87_3	GBIFCH01223090	sp. 52	n/a	MN023307	genseq-2 16S	
Thraulus (Thraulus) granti	TH5_PNG124_2	GBIFCH01223093	sp. 3	n/a	MN023278	genseq-2 16S	1400–2700
	TH3_PNG173_1	GBIFCH01223094	sp. 4	n/a	MN023257	genseq-2 16S	
	TH5_PNG124_3	GBIFCH01223092	sp. 3	n/a	MN023279	genseq-1 16S	
Thraulus (Thraulus) longinquus	TH5_PNG156_2	GBIFCH01223098	sp. 2	n/a	MN023277	genseq-1 16S	150
Thraulus (Thraulus) nabire	TH5_PAP11_1	GBIFCH01223096	sp. 12	n/a	MN023263	genseq-1 16S	350
	TH5_PAP11_3	GBIFCH01223097	sp. 12	n/a	MN023300	genseq-2 16S	
Thraulus (Thraulus) noe	BA3_PNG166_1	GBIFCH01223111	sp. 51	MN038352	n/a	genseq-2 CO1	700
	BA3_PNG166_2	GBIFCH01223112	sp. 51	MN038353	MN023242	genseq-2 CO1, 16S	
	BA3_PNG166_3	GBIFCH01223114	sp. 51	MN038354	MN023243	genseq-1 CO1, 16S	
	NO2_PNG166_2	GBIFCH01223113	sp. 51	MN038373	n/a	genseq-2 CO1	
Thraulus (Thraulus) ogea	BA3_PNG179_1	GBIFCH01223108	sp. 9	n/a	MN023260	genseq-1 16S	30
	BA3_PNG179_2	GBIFCH01223106	sp. 9	n/a	MN023261	genseq-2 16S	
	BA3_PNG179_3	GBIFCH01223107	sp. 9	n/a	MN023262	genseq-2 16S	
Thraulus (Thraulus) timorensis	TH1_TIM11_1	GBIFCH01223095	sp. 50	n/a	MN023312	genseq-1 16S	150
Thraulus (Thraulus) wemale	BA3_AMB6_1	GBIFCH01223104	sp. 8	n/a	MN023273	genseq-2 16S	100–1350
	BA3_AMB6_2	GBIFCH01223103	sp. 8	n/a	MN023274	genseq-2 16S	
	BA3_AMB7_1	GBIFCH01223105	sp. 8	MN038351	MN023302	genseq-2 CO1, 16S	
	BA3_AMB7_2	GBIFCH01223102	sp. 8	n/a	MN023275	genseq-2 16S	
	BA3_AMB7_3	GBIFCH01223099	sp. 8	n/a	MN023309	genseq-1 16S	
	TH6_AMB3_1	GBIFCH01223149	sp. 6	n/a	MN023251	genseq-2 16S	
	BA3_AMB10_1	GBIFCH01223100	sp. 7	n/a	MN023271	genseq-2 16S	
	BA3_AMB13_1	GBIFCH01223101	sp. 7	n/a	MN023272	genseq-2 16S	
Thraulus (Thraulus) sp. A	TH5_PNG119_3	GBIFCH01223091	sp. 1	n/a	MN023267	genseq-3 16S	400
Thraulus (Masharikella) iteris	TH2_PNG169_1	GBIFCH01223115	sp. 43	n/a	MN023248	genseq-1 16S	300–900
	TH2_PNG169_3	GBIFCH01223116	n/a	n/a	n/a	n/a	
	TH2_PNG168_1	GBIFCH01223117	n/a	n/a	n/a	n/a	
Thraulus (Masharikella) johannisluci	TH2_PNG169_2	GBIFCH01223118	sp. 32	MN038388	MN023247	genseq-1 CO1, 16S	900
Thraulus (Masharikella) pascalae	TH3_PAP13_1	GBIFCH01223150	sp. 13	n/a	MN023265	genseq-2 16S	160
	TH3_PAP13_2	GBIFCH01223119	sp. 13	n/a	MN023266	genseq-1 16S	
Thraulus (Masharikella) samueli	TH2_PNG159_1	GBIFCH01223121	sp. 19	MN038385	MN023283	genseq-2 CO1, 16S	600
	TH2_PNG159_2	GBIFCH01223120	sp. 19	MN038386	n/a	genseq-1 CO1	
	TH2_PNG159_3	GBIFCH01223122	sp. 19	MN038387	MN023284	genseq-2 CO1, 16S	
Thraulus (Masharikella) sp. B	TH2_PAP17_1	GBIFCH01223123	sp. 44	n/a	MN023305	genseq-3 16S	550
*Nonnullidensalvarezi*	NO2_PNG106_1	GBIFCH01223124	sp. 30	MN038371	MN023249	genseq-1 CO1, 16S	1400–2200
	NO2_PNG90_1	GBIFCH01223127	sp. 27	MN038374	MN023245	genseq-2 CO1, 16S	
	NO2_PNG106_3	GBIFCH01223126	n/a	n/a	n/a	n/a	
	NO2_PNG106_2	GBIFCH01223125	sp. 36	MN038372	MN023244	genseq-2 CO1, 16S	
*Nonnullidensanga*	NO4_PNG96_1	GBIFCH01223129	n/a	n/a	n/a	n/a	1700
*Nonnullidensboonsoongi*	NO3_PNG150_1	GBIFCH01223133	sp. 31	MN038376	n/a	genseq-2 CO1	1400–2500
	NO3_PNG172_3	GBIFCG01223130	n/a	n/a	n/a	n/a	
	NO3_PNG172_1	GBIFCH01223132	n/a	n/a	n/a	n/a	
	NO3_PNG172_2	GBIFCH01223131	sp. 37	n/a	MN023241	genseq-2 16S	
*Nonnullidenscozzarolae*	NO3_PNG142_1	GBIFCH01223140	sp. 28	MN038375	n/a	genseq-2 CO1	1400–1800
	NO3_PNG173_3	GBIFCH01223139	sp. 26	n/a	MN023294	genseq-2 16S	
	NO3_PNG173_2	GBIFCH01223138	sp. 26	MN038378	n/a	genseq-2 CO1	
	NO3_PNG173_1	GBIFCH01223137	sp. 26	MN038377	MN023293	genseq-2 CO1, 16S	
	NO3_PNG87_1	GBIFCH01223134	sp. 29	MN038379	MN023311	genseq-1 CO1, 16S	
	NO3_PNG87_2	GBIFCH01223135	sp. 29	n/a	MN023290	genseq-2 16S	
	NO3_PNG87_3	GBIFCH01223136	sp. 29	n/a	MN022291	genseq-2 16S	
*Nonnullidensfuyugensis*	NO2_PNG166_3	GBIFCH01223141	n/a	n/a	n/a	n/a	1700
	NO2_PNG166_1	GBIFCH01223142	sp. 44	n/a	MN023235	genseq-2 16S	
*Nonnullidenskaltenbachi*	NO1_PNG117_1	GBIFCH01223143	sp. 21	n/a	MN023239	genseq-1 16S	80–400
	NO6_PNG119_1	GBIFCH01223144	sp. 21	n/a	MN023240	genseq-2 16S	
*Nonnullidensmarcelae*	NO4_BH20_1	GBIFCH01223152	sp. 25	n/a	MN023269	genseq-2 16S	100
	NO4_BH20_2	GBIFCH01223151	sp. 25	n/a	MN023270	genseq-1 16S	
*Nonnullidensmoiorum*	NO4_BH20_3	GBIFCH01223147	sp. 39	n/a	MN023313	genseq-1 16S	100
	NO5_BH20_1	GBIFCH01223148	sp. 38	n/a	MN023292	genseq-2 16S	
*Nonnullidenssilvaepumilorum*	BA2_PNG156_2	GBIFCH01223146	n/a	n/a	n/a	n/a	130
*Nonnullidens* sp. A	NO7_PAP9_1	GBIFCH01223145	sp. 22	MN038380	MN023256	genseq-3 CO1, 16S	800
*Nonnullidens* sp. B	NO1_PNG133_1	GBIFCH01223128	sp. 23	n/a	MN023238	genseq-3 16S	1800–2000
*Kosminymphaasarorum*	BA1_PNG106_1	GBIFCH01223156	sp. 40	MN038355	MN023285	genseq-1 CO1, 16S	2200
*Kosminymphabalkei*	BA1_PNG87_1	GBIFCH01223157	sp. 14	n/a	MN023252	genseq-2 16S	1700–1800
	BA1_PNG87_3	GBIFCH01223158	n/a	n/a	n/a	n/a	
*Kosminymphabaruya*	BA1_PNG90_1	GBIFCH01223160	sp. 18	MN038349	MN023299	genseq-2 CO1, 16S	1400
	BA2_PNG172_1	GBIFCH01223159	n/a	n/a	n/a	n/a	
*Kosminymphakalamorum*	BA1_PNG134_1	GBIFCH01223161	sp. 42	n/a	MN023286	genseq-1 16S	1800–2200
	BA4_PNG106_2	GBIFCH01223163	sp. 41	MN038356	MN023288	genseq-2 CO1, 16S	
	BA1_PNG134_2	GBIFCH01223162	sp. 41	MN038347	MN023287	genseq-2 CO1, 16S	
*Kosminymphapaulinae*	BA4_PNG96_1	GBIFCH01223164	n/a	n/a	n/a	n/a	1700
*Kosminymphasabrinae*	BA1_PNG90_2	GBIFCH01223165	sp. 16	n/a	MN023295	genseq-1 16S	1200–2000
	BA1_PNG90_3	GBIFCH01223166	sp. 16	n/a	MN023296	genseq-2 16S	
	BA1_PNG142_1	GBIFCH01223171	n/a	n/a	n/a	n/a	
	BA1_PNG152_2	GBIFCH01223174	n/a	n/a	n/a	n/a	
	BA1_PNG152_1	GBIFCH01223173	sp. 15	n/a	MN023298	genseq-2 16S	
	BA1_PNG171_1	GBIFCH01223167	n/a	n/a	n/a	n/a	
	BA1_PNG134_3	GBIFCH01223177	n/a	n/a	n/a	n/a	
	BA1_PNG153_1	GBIFCH01223176	sp. 15	n/a	MN023297	genseq-2 16S	
	BA1_PNG142_2	GBIFCH01223170	n/a	n/a	n/a	n/a	
	BA1_PNG142_3	GBIFCH01223172	sp. 20	MN038348	MN023255	genseq-2 CO1, 16S	
	BA1_PNG173_3	GBIFCH01223169	sp. 17	n/a	MN023254	genseq-2 16S	
	BA1_PNG152_3	GBIFCH01223175	n/a	n/a	n/a	n/a	
	BA1_PNG173_2	GBIFCH01223168	sp. 17	n/a	MN023253	genseq-2 16S	

## ﻿Results

### ﻿Taxonomic account

#### ﻿Genus Thraulus

Taxon classificationAnimaliaEphemeropteraLeptophlebiidae

Eaton, 1881

0823F45A-A6A9-5921-96EF-62F8F6B54369

##### Diagnosis.

Nymphs of the genus *Thraulus* are adequately characterised by Grant (2024): two dorsal rows of setae on the dorsal face of labrum; width of labrum subequal to width of clypeus (range: 0.81–1.07); lateral margins of clypeus parallel; posterolateral projections on abdominal segments VI–IX, VII–IX or VIII–IX; gills I–VII dissimilar; gill I composed of one or two lanceolate and long lamellae, or a dorsal lanceolate lamella and a ventral oval and fringed lamella; gills II–VII composed of two fringed lamellae along the entire margin, with some species having the basal half of lateral margin bare. Contrary to Grant (2024) the ventral side of labrum can bear not only a row of short stout setae (e.g., Fig. 12B) but in some species scattered stout or thin setae (e.g., Fig. 18B). The hypopharynx does not always bear a “small, rounded, posterolateral projection on arms of the superlingua”, compare for instance Fig. 21F and Fig. 29F.

##### Species composition.

The species *Thrauluseatoni* Grant, 2024 [Indonesia Sulawesi], *Thraulusparentalis* Grant, 2024 [peninsular Malaysia], *Thrauluspetersorum* Grant, 2024 [peninsular Malaysia, east Malaysia Sabah], *Thraulussemicastaneus* (Gillies, 1951) [southern India], *Thraulusthraker* Jacob, 1988 [Bulgaria] cannot be assigned to a subgenus because their nymphs are still unknown.

#### ﻿ Subgenus Thraulus

Taxon classificationAnimaliaEphemeropteraLeptophlebiidae

Eaton, 1881

5F268960-C10D-5150-B425-F2F43AD55B2A

##### Diagnosis.

Identical to the generic diagnosis, except that gill I is always composed of one or two lanceolate and long lamellae.

##### Type species.

*Thraulusbellus* Eaton, 1881 by original designation.

##### Included species.

- Thraulus (Thraulus) amravati Vasanth, Subramanian & Selvakumar, 2022 [southern India],
- T. (T.) bellus Eaton, 1881 [Europe],
- T. (T.) bishopi Peters & Tsui, 1972 [Peninsular Malaysia, Vietnam, southeastern China],
- T. (T.) connubialis Grant, 2024 [east Malaysia Sabah],
- T. (T.) cursus Grant, 2024 [Japan],
- T. (T.) cuspidatus Vasanth, Subramanian & Selvakumar, 2022 [southern India],
- T. (T.) demoulini Peters & Tsui, 1973 [Thailand],
- Th. (Th.) eloisae sp. nov. [Papua New Guinea],
- T. (T.) fatuus Kang & Yang, 1994 [Taiwan, Japan],
- T. (T.) femoratus Li, Liu & Zhou, 2006 [southeastern China],
- Th. (Th.) granti sp. nov. [Papua New Guinea],
- T. (T.) ishiwatai Grant, 2024 [Japan Okinawa],
- T. (T.) jacobusi Srinivasan, Sivaruban & Barathy, 2022 [southern India],
- Th. (Th.) longinquus sp. nov. [Papua New Guinea],
- T. (T.) macilentus Kang & Yang, 1994 [Taiwan, Japan],
- T. (T.) madagasikarensis Grant, 2024 [Madagascar],
- T. (T.) mudumalaiensis Soman, 1991 [southern India],
- Th. (Th.) nabire sp. nov. [Indonesia, Papua],
- T. (T.) nihonensis Grant, 2024 [Japan Okinawa],
- Th. (Th.) noe sp. nov. [Papua New Guinea],
- Th. (Th.) ogea sp. nov. [Papua New Guinea],
- T. (T.) plumeus Selvakumar, Vasanth & Subramanian, 2022 [northern India],
- Th. (Th.) timorensis sp. nov. [Indonesia, West Timor],
- T. (T.) turbinatus (Ulmer, 1909) [Comoros],
- T. (T.) umbrosus Kang & Yang, 1994 [Taiwan],
- T. (T.) vellimalaiensis Vasanth, Subramanian & Selvakumar, 2022 [southern India],
- Th. (Th.) wemale sp. nov. [Indonesia, Seram].

##### Distribution.

Mainly in the Oriental and Palaearctic regions, with only two species known from the Afrotropical area in the islands of Comoros and Madagascar; the subgenus seems absent from continental Africa south of the Sahara, as well as from Australia.

#### ﻿ Thraulus (Thraulus) eloisae sp. nov.

Taxon classificationAnimaliaEphemeropteraLeptophlebiidae

673A0BA7-DB47-5123-AC42-8D352F7DF545

https://zoobank.org/0730D92D-0EB9-415F-8E2B-599FAF4E249E

**Figure 2.**
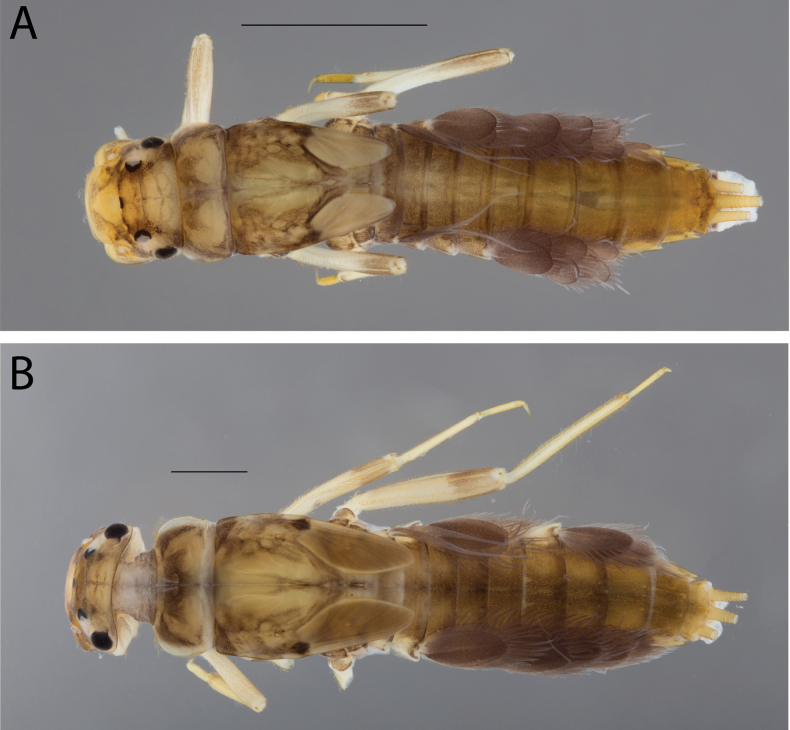
Habitus of **A.**Thraulus (Thraulus) eloisae sp. nov. and **B.**Thraulus (Thraulus) granti sp. nov. Scale bars: 1 mm.

**Figure 3.**
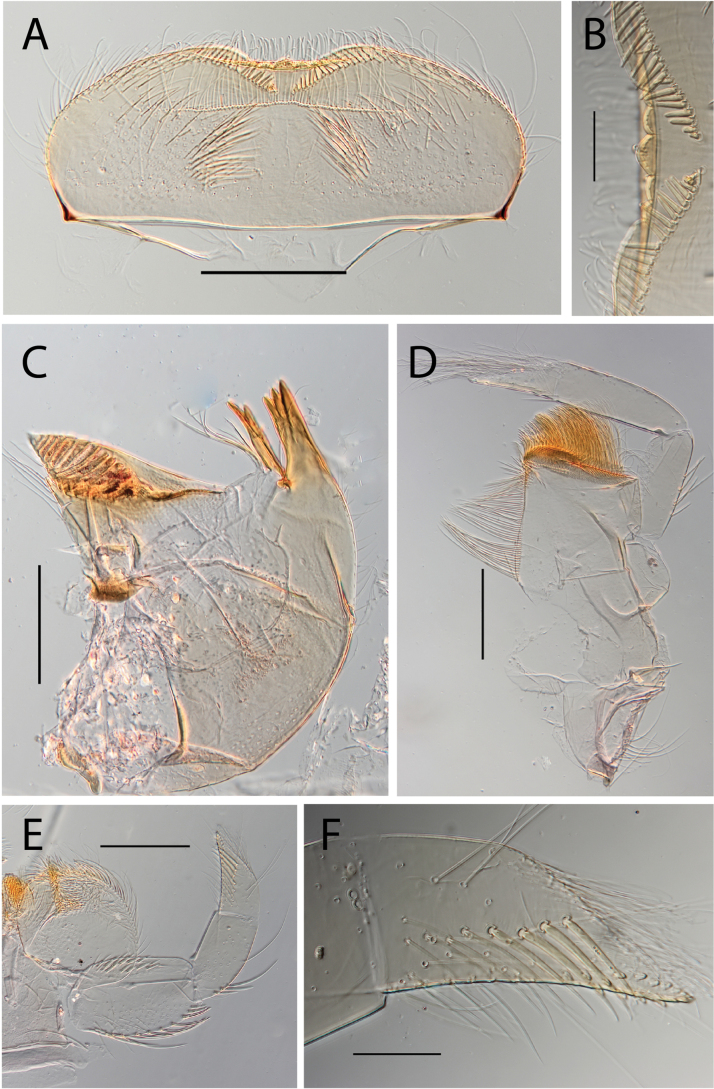
Thraulus (Thraulus) eloisae sp. nov. nymphal mouthparts. **A.** Labrum, dorsal view; **B.** Emargination of the labrum; **C.** Right mandible; **D.** Maxilla; **E.** Labium; **F.** Labial palp, segment III. Scale bars: 200 µm (**A, C–E**); 50 µm (**B, F**).

**Figure 4.**
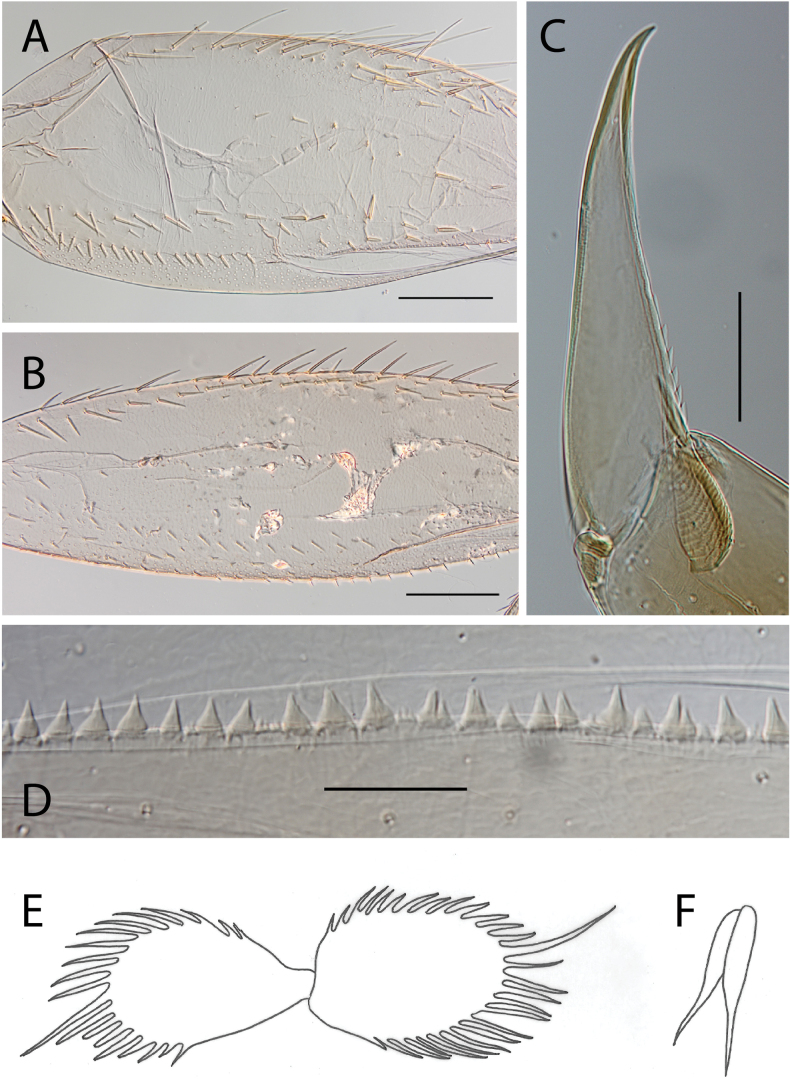
Thraulus (Thraulus) eloisae sp. nov. thorax and abdomen. **A.** Fore femur; **B.** Hind femur; **C.** Claw; **D.** Posterior margin of tergite IX; **E.** Gill IV; **F.** Gill VII. Scale bars: 200 µm (**A, B**); 50 µm (**C, D**).

Figs 2A , 3 , 4 , 34A

##### Material examined.

***Holotype*.** • Papua New Guinea, nymph on slide, Eastern Highlands Province, Marawaka, Ande, 1700–1800 m, 09.XI.2006, 07°01.697'S, 145°49.807'E, M. Balke and Kinibel col [PNG87], GBIFCH01223087 (ZSM). ***Paratypes*.** 3 nymphs in ethanol, GBIFCH01523471, 2 nymphs entirely mounted on slide, GBIFCH01223090, GBIFCH01223089, same data as holotype (MZL). • Papua New Guinea, 1 nymph mounted on slide, GBIFCH01223088, Central Province, Woitape, 1500 m, I.2008, 08°33.178'S, 147°15.481'E, Posman col [PNG167] (MZL).

##### Nymph.

Body length, male: 6–6.5 mm; female: 7–7.5 mm.

##### Diagnosis.

Labrum rectangular, with emargination shallow; proximo-lateral margin of stipes with a single long and stout seta; maxillary palp greatly elongated; outer margin of maxillary palp segment II without stout setae; inner margin of labial palp segment I with 16–18 stout setae; apex of superlingua truncate. Gills II–VI completely fringed with filaments, with ~ 30 filaments on dorsal lamella and ~ 40 on ventral lamella; posterior margin of abdominal segment VIII without denticles; posterolateral projections of the abdomen present on segments VIII–IX.

##### Colouration.

***Head*** yellowish, greyish between ocelli. Antenna yellowish. Compound eye lower portion black, upper portion brownish orange. Mouth parts pale brown. ***Thorax*.** Pronotum pale brown, with greyish areas laterally and medially as in Fig. 2A. Mesonotum greyish brown with scattered lighter maculae. Femur fore femur greyish, mid- and hind femur whitish, distal 1/3 washed with grey. Tibia yellowish orange, with apical macula greyish. Tarsi orange to yellow. ***Abdomen*.** Terga evenly greyish brown, lighter along the sagittal line. Sterna pale brown. Gills pale brown to greyish brown. Terminal filaments uniformly pale brown.

##### Description.

***Head*. *Labrum*** rectangular (Fig. 3A). Ratio labrum width/clypeus width 0.87–0.90. Ratio labrum width/insertion width 1.08–1.10. Medial emargination shallow with rounded denticles (Fig. 3B). Proximal row of setae present, simple. Number of setae on proximal row ~ 75–100. Distal row of setae present, simple. Median part of labrum in ventral view with stout setae in row (Fig. 3B). ***Mandibles*.** Outer margin with row of setae on distal 2/3. Ending of the row before incisor. Right mandible with 10 or 11 setae below the mola (Fig. 3C). ***Maxillae*.** Posterior margin of cardo with few long hair-like setae, together with some long and stout setae (Fig. 34A). Proximo-lateral margin of stipes with a single long and stout seta. Apical-ventral row with 15–18 pectinate setae. Maxillary palp 1.5 × longer than maxilla; maxillary palp segment II 1.17–1.39 × as long as segment I. Maxillary palp segment III 0.77–0.94 as long as segment I (Fig. 3D). Few stout setae on outer margin of segment I. Segment II inner margin with one stout seta. Segment II outer margin without stout setae. Segment III inner margin with three or four stout setae, dorsal surface fully covered with setae, 2.57–2.64 × as long as base width. ***Labium*.** Labial palp segment II 0.59–0.71 as long as segment I. Labial palp segment III 0.64–0.71 as long as segment I (Fig. 3E). Segment I inner margin with 16–18 stout setae, outer margin with 12–15 stout setae. Segment II outer margin with two or three stout setae. Segment III dorsal face with six or seven stout setae (Fig. 3F), 2.00–2.43 × as long as base width. *Hypopharynx* apex of superlingua truncate.

***Thorax*.** Dorsal margin of femur with a row of long, stout, pointed setae; submarginal row with setae as long as those of dorsal margin (Fig. 4A); ventral margin with short, blunt, stout setae and a submarginal row of long, pointed, stout setae, except on hind leg where they are short and blunt (Fig. 4B); central area of upper surface with few long pointed setae. Fore tibia with several rows of numerous simple feathered setae on ventral margin; outer margin with few hair-like setae; middle tibia with two rows of long pointed setae on ventral margin, outer margin with few hair-like setae; hind tibia with a row of long thin setae on ventral margin, upper and lower surfaces covered with simple and some feathered setae, outer margin with short and long, pointed, stout setae. Tarsal claw straight with 5–8 minutes denticles subequal in length (Fig. 4C).

***Abdomen*.** Gill I with single bifid lamella. Gills II to VII with dorsal and ventral lamellae, ventral lamella smaller. Gills II–VI with upper lamella lacking filaments on basal half of outer margin, with ~ 25–30 short filaments, lower lamella with ~ 35–40 short filaments (Fig. 4E); on both lamellae apical filament much longer than the others; gill VII small, with narrow lamellae ending with a single filament (Fig. 4F). Posterior margin of terga IX with denticles (Fig. 4D). Posterior margin of terga V–VIII without denticles. Posterolateral projections present on segments VIII and IX.

##### Derivatio nominis.

This species is dedicated to Eloïse Sartori, daughter of the first author.

#### ﻿ Thraulus (Thraulus) granti sp. nov.

Taxon classificationAnimaliaEphemeropteraLeptophlebiidae

4C6BE539-23B6-5C4F-9C1A-7BAB34A52F25

https://zoobank.org/9E6E09E9-7B68-425A-8175-0470C684C9EF

Figs 2B , 5 , 6

##### Material examined.

***Holotype*.** • Papua New Guinea, nymph on slide, Enga Province, Kumul Lodge at foot of Mt Hagen, 2700 m, 05.XII.2006, 05°47.548'S, 143°58.761'E, M. Balke and Kinibel col [PNG124], GBIFCH01223092 (ZSM). ***Paratypes*.** 4 nymphs in ethanol, GBIFCH01523472, 1 nymph entirely mounted on slide, GBIFCH01223093, same data as holotype (MZL). • Papua New Guinea, one nymph on slide, GBIFCH01223094, Central Province, Kokoda trek, 1390 m, I.2006, 09°00.338'S, 147°44.252'E, Posman col. [PNG173] (MZL).

##### Nymph.

Body length, female: 10.5–11 mm.

##### Diagnosis.

Labrum cordiform, medial emargination shallow with flat denticles; dorsal face of labrum with a simple row in distal position; median part of labrum in ventral view with stout setae in row; apical ventral row of maxilla composed of 19–23 pectinate setae; tarsal claw with 7–10 teeth; gill lamellae on segments II–VI with > 25 filaments each; posterolateral projections of the abdomen present on segments VII–IX.

##### Colouration.

***Head*.** Pale brown, medium brown between ocelli. Antenna broken. Mouth parts pale brown. ***Thorax*.** Pronotum yellowish brown, washed with greyish maculae laterally and medially as in Fig. 2B. Mesonotum greyish brown with scattered lighter maculae; anterolateral base of forewing pads with a blackish dot. Femur yellowish with distal 1/3 greyish; forefemur also with a middle greyish band. Tibia yellowish, apex of fore tibia greyish. Tarsi yellowish. ***Abdomen*.** Terga evenly greyish brown with a pair of lighter maculae on segments II–VII; segments VIII and IX darker, segment X lighter. Sterna yellowish grey in the middle, yellowish on sides, segments VIII and IX darker, brownish. Gills greyish brown, upper lamella darker. Terminal filaments uniformly yellowish.

##### Description.

**Figure 5.**
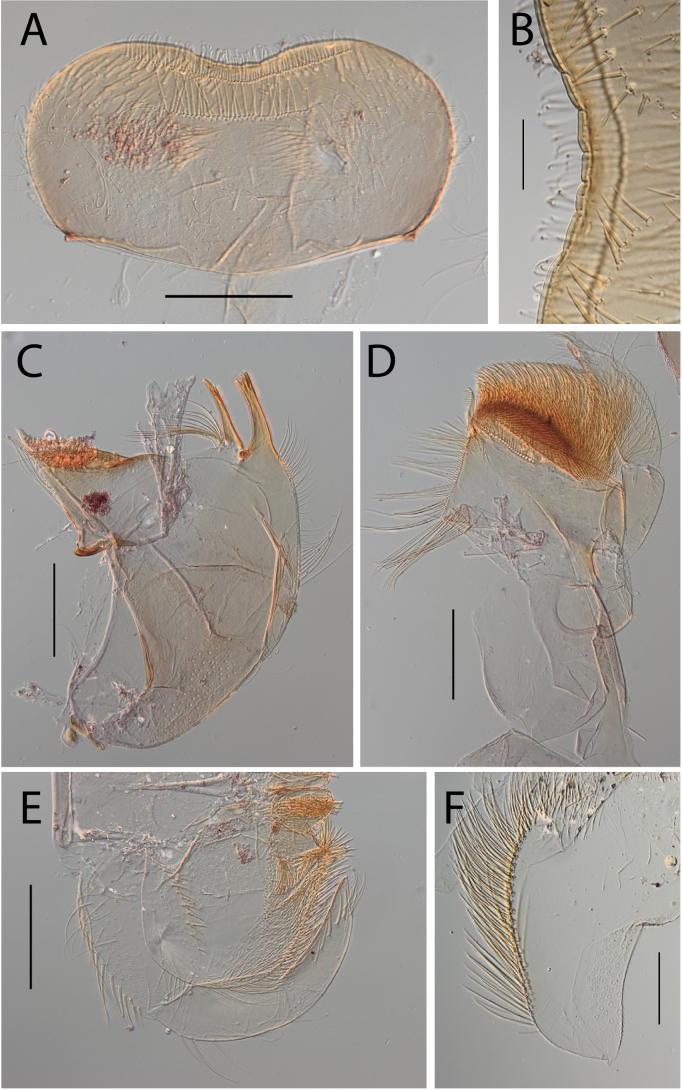
Thraulus (Thraulus) granti sp. nov. nymphal mouthparts. **A.** Labrum, dorsal view; **B.** Emargination of the labrum; **C.** Right mandible; **D.** Maxilla **E.** Labium **F.** Superlingua of hypopharynx. Scale bars: 200 µm (**A, C–E**); 50 µm (**B**); 100 µm (**F**).

***Head*. *Labrum*** cordiform (Fig. 5A). Ratio labrum width/clypeus width 0.90–0.95. Ratio labrum width/insertion width 1.23–1.24. Medial emargination shallow with flat denticles. Proximal row of setae present, simple. Number of setae on proximal row ~ 26–40. Distal row of setae present, simple. Median part of labrum in ventral view with stout setae in row (Fig. 5B). ***Mandibles*.** Outer margin with row of setae on distal 2/3. Ending of the row near incisor. Right mandible with 10–17 setae below the mola (Fig. 5C). ***Maxillae*.** Posterior margin of cardo with few hair-like setae and three or four stout and long setae in submarginal position. Proximo-lateral margin of stipes with a single long and stout seta. Apical-ventral row with 19–23 pectinate setae. Maxillary palp segment II 0.89–1.00 as long as segment I. Maxillary palp segment III 0.67–0.80 as long as segment I (Fig. 5D). Stout setae on outer margin of segment I absent. Segment II inner margin with 3–7 stout setae, outer margin with 3–5 stout setae. Segment III inner margin with four or five stout setae, dorsal surface with few setae at apex, 1.60–2.10 × as long as base width. ***Labium*.** Labial palp segment II 0.69–0.74 as long as segment I. Labial palp segment III 0.96–0.86 as long as segment I (Fig. 5E). Segment I inner margin with 9–15 stout setae, outer margin with 15–20 stout setae. Segment II outer margin with two to four stout setae. Segment III dorsal face with five or six setae, 3.00–3.38 × as long as base width. ***Hypopharynx*.** Apex of superlingua truncate (Fig. 5F).

**Figure 6.**
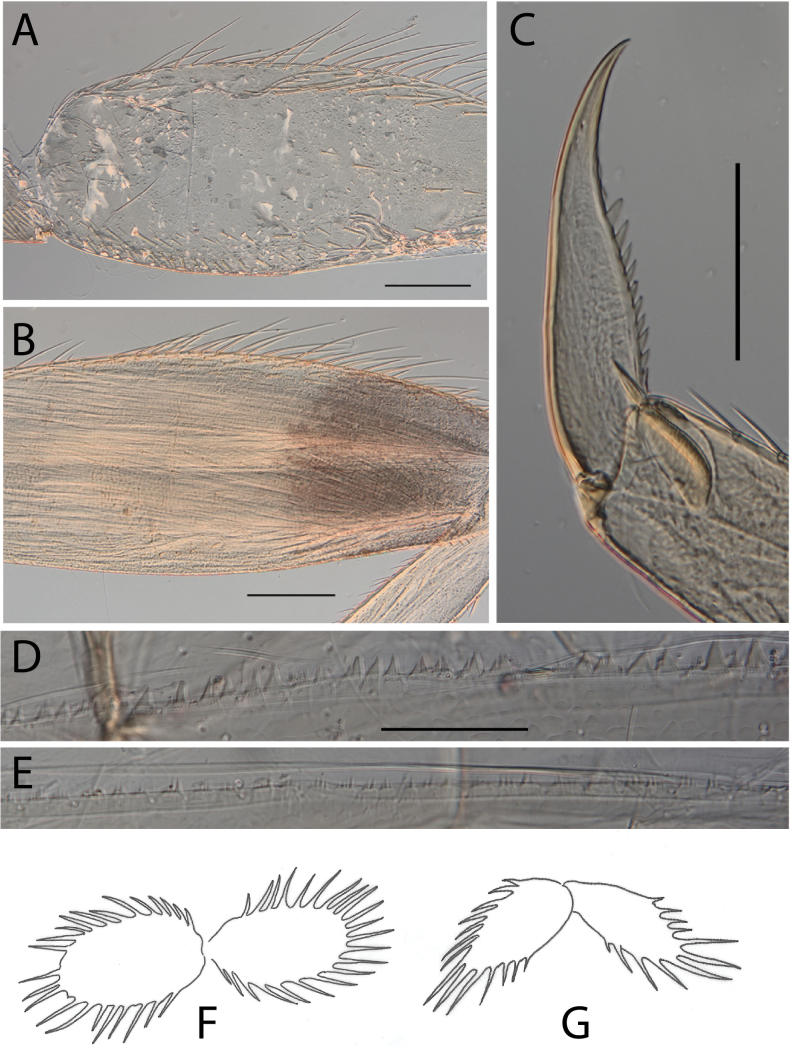
Thraulus (Thraulus) granti sp. nov. thorax and abdomen. **A.** Fore femur; **B.** Hind femur; **C.** Claw; **D.** Posterior margin of tergite IX; **E.** Posterior margin of tergite VIII; **F.** Gill IV; **G.** Gill VII. Scale bars: 200 µm (**A, B**); 100 µm (**C**); 50 µm (**D, E**).

***Thorax*.** Dorsal margin of femur with a row of long, stout, pointed setae; irregular submarginal row with setae as long as or half the size of those of dorsal margin (Fig. 6A); ventral margin with shorter, stout setae and a submarginal row of long and pointed stout setae, except on hind leg where those on ventral margin are short and blunt (Fig. 6B); central area of upper surface with few long, pointed setae somewhat arranged in a longitudinal row. Fore tibia with several rows of numerous simple, feathered setae on ventral margin; outer margin of fore tibia with few hair-like setae; middle tibia with two rows of long, pointed setae on ventral margin, outer margin with numerous hair-like setae; hind tibia with a row of long thin setae on ventral margin, upper and lower surface covered with simple and some feathered setae, outer margin with short and long, pointed, stout setae. Tarsal claw straight with 7–10 denticles progressively larger apically (Fig. 6C).

***Abdomen*.** Gill I with single bifid lamella. Gills II–VII with dorsal and ventral lamellae, dorsal lamella smaller. Gill II–VI with upper and lower lamella entirely bordered with ~ 30 short filaments (Fig. 6F); gill VII with ~ 15 filaments and ~ 10 filaments on upper and lower lamellae, respectively (Fig. 6G). Posterior margin of terga IX with minute denticles (Fig. 6D), or with small denticles. Posterior margin of terga VIII with minute denticles (Fig. 6E). Posterior margin of terga V–VII without denticles. Posterolateral projections present on segments VII–IX.

##### Derivatio nominis.

This species is dedicated to Prof. Peter Grant (SWOSU, Oklahoma, USA) for his outstanding contribution to the study of the genus *Thraulus*.

#### ﻿ Thraulus (Thraulus) longinquus sp. nov.

Taxon classificationAnimaliaEphemeropteraLeptophlebiidae

FCCCD12E-5C33-593F-B5CC-41CF983A4D9E

https://zoobank.org/E66693AF-FDC7-44F9-9AA6-54CF5AAB4F5D

Figs 7 , 8

##### Material examined.

***Holotype*.** • Papua New Guinea, nymph on slide, Madang Province, Aiome area, 130 m, 11.III.2007, 05°10.593'S, 144°42.800'E, Kinibel col [PNG156], GBIFCH01223098 (ZSM). ***Paratype*.** • Papua New Guinea, one nymph in ethanol, GBIFCH01523473, same data as holotype (MZL).

##### Nymph.

Body length, male: 5.5 mm.

##### Diagnosis.

Labrum cordiform, medial emargination shallow with flat denticles; dorsal face of labrum with a simple row in distal position; median part of labrum in ventral view with stout setae in row; apical ventral row of maxilla composed of 17 pectinate setae; tarsal claw with six teeth; gill lamellae on segments II–VI with > 25 filaments each; posterolateral projections of the abdomen present on segments VII–IX.

##### Colouration.

***Head*.** Yellowish brown, medium brown between ocelli. Antenna yellowish, pedicel dark brown. Compound eye lower portion black, upper portion medium brown. Mouth parts yellowish brown. ***Thorax*.** Pronotum pale brown washed with medium brown. Mesonotum pale brown washed with medium brown. Femur whitish, apex tinted with dark brown. Tibia whitish. Tarsi yellowish. ***Abdomen*.** Terga uniformly dark brown, sagittal line somewhat paler. Sterna greyish brown. Gills lamellae pale grey, filaments greyish purple. Terminal filaments broken.

##### Description.

**Figure 7.**
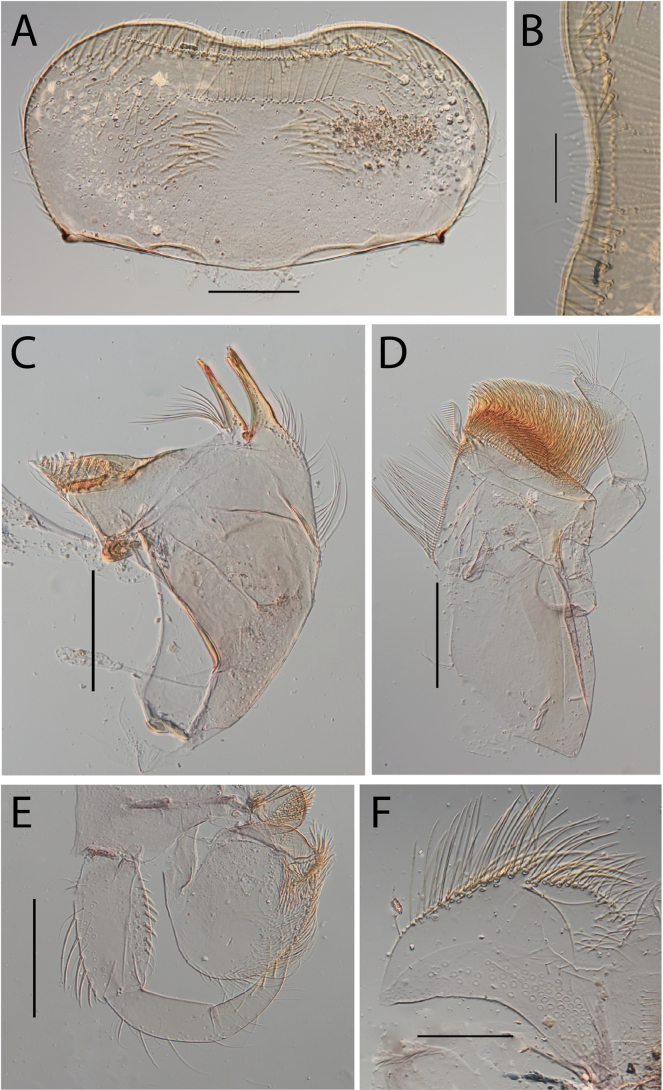
Thraulus (Thraulus) longinquus sp. nov. nymphal mouthparts. **A.** Labrum, dorsal view; **B.** Emargination of the labrum; **C.** Right mandible; **D.** Maxilla; **E.** Labium; **F.** Superlingua of hypopharynx. Scale bars: 100 µm (**A, F**); 50 µm (**B**); 200 µm (**C–E**).

***Head*. *Labrum*** cordiform (Fig. 7A). Ratio labrum width/clypeus width 0.91. Ratio labrum width/insertion width 1.21. Medial emargination shallow with flat denticles. Proximal row of setae present, simple. Number of setae on proximal row ~ 24. Distal row of setae present, simple. Median part of labrum in ventral view with stout setae in row (Fig. 7B). ***Mandibles*.** Outer margin with middle and distal tuft of setae. Right mandible with 12 setae below the mola (Fig. 7C). ***Maxillae*.** Posterior margin of cardo broken. Proximo-lateral margin of stipes with a single long and stout seta. Apical-ventral row with 17 pectinate setae. Maxillary palp segment II 0.82 as long as segment I. Maxillary palp segment III 0.75 as long as segment I (Fig. 7D). Stout setae on outer margin of segment I absent. Segment II inner margin with three stout setae. Segment II outer margin without stout setae. Segment III inner margin with four stout setae. Segment III dorsal surface with few setae at apex. Segment III 1.60 × as long as base width. ***Labium*.** Labial palp segment II 0.68 as long as segment I. Labial palp segment III 0.76 as long as segment I (Fig. 7E). Segment I inner margin with nine stout setae, outer margin with eight stout setae. Segment II outer margin with four stout setae. Segment III dorsal face with four stout setae. Segment III 3.11 × as long as base width. ***Hypopharynx*.** Apex of superlingua pointed (Fig. 7F).

**Figure 8.**
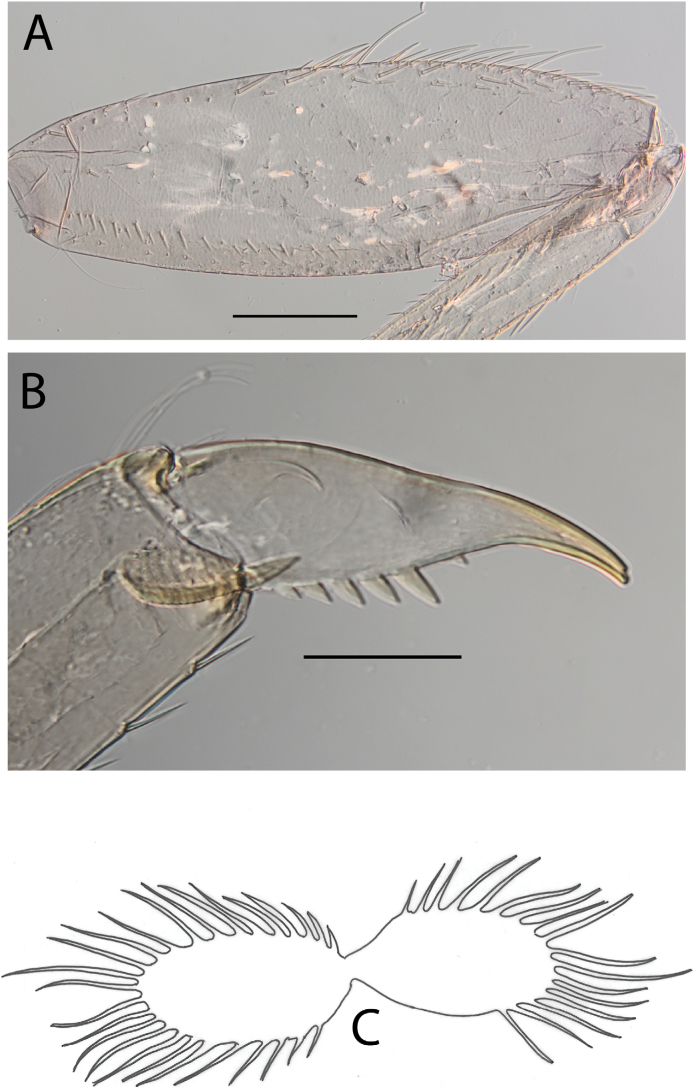
Thraulus (Thraulus) longinquus sp. nov. thorax and abdomen. **A.** Hind femur; **B.** Claw; **C.** Gill IV. Scale bars: 200 µm (**A**); 50 µm (**B**).

***Thorax*.** Dorsal margin of femur with a row of long, stout, pointed setae; irregular submarginal row of shorter pointed setae; ventral margin with short stout setae and a submarginal row of long, pointed, stout setae, except on hind leg where those on ventral margin are very short and blunt (Fig. 8A); central area of upper surface with minute pointed setae. Middle tibia with two rows of long, simple, feathered, or pointed setae on ventral margin, outer margin with few hair-like setae; hind tibia with row of simple long and thin setae on ventral margin, upper and lower surface covered with simple and feathered setae, outer margin with short and long pointed, stout setae and hair-like setae. Tarsal claw straight with six denticles progressively larger apically (Fig. 8B).

***Abdomen*.** Gill I with single bifid lamella. Gills II–VII with dorsal and ventral lamellae of the same size. Gill II–VI with upper lamella lacking filaments on basal half of outer margin, with ~ 25–30 filaments on each lamella (Fig. 8C); gill VII with ~ 15 filaments on each lamella. Posterior margin of terga IX with denticles. Posterior margin of terga VIII with minute denticles. Posterior margin of terga V–VII without denticles. Posterolateral projections present on segments VII–IX.

##### Derivatio nominis.

The Latin adjective *longinquus* means distant or lost, as an indication of the remote place where the species was found.

#### ﻿ Thraulus (Thraulus) nabire sp. nov.

Taxon classificationAnimaliaEphemeropteraLeptophlebiidae

09A3A1A7-4799-5037-9F4F-E58CE6DAA3B6

https://zoobank.org/79B7ED5D-36D8-4347-8776-72A825F2028C

Figs 9 , 10

##### Material examined.

***Holotype*.** • Indonesia, Papua, nymph on slide, Road Nabire-Enarotali KM 62, 340 m, 22.X.2011, 03°31.684'S, 135°42.802'E, M. Balke col [PAP11], GBIFCH01223096 (MZB). ***Paratypes*.** • Indonesia, Papua, 1 nymph in ethanol, GBIFCH01523574, 1 nymph mounted on slide, GBIFCH01223097, same data as holotype (MZL).

##### Nymph.

Body length, male: 4.5 mm.

##### Diagnosis.

Labrum cordiform, medial emargination shallow with flat denticles; median part of labrum in ventral view with stout setae in a row; proximal row of setae on dorsal face of labrum with 19–23 setae; apical ventral row of maxilla composed of 15–17 pectinate setae; tarsal claw with eight or nine teeth, distal one much larger; gill lamellae on segments II–VI fringed only on distal half, with < 20 filaments on each.

##### Colouration.

***Head*.** Greyish brown, clypeus medium brown. Antenna yellowish. Compound eye lower portion black, upper portion brownish red. Mouth parts pale brown, labrum and mandibles medium brown. ***Thorax*.** Pronotum yellowish brown, greyish brown on edges and in the middle. Mesonotum pale brown, washed with greyish maculae. Femur whitish with distal 1/3 greyish. Tibia yellowish. Tarsi yellowish. ***Abdomen*.** Terga: segments II–VIII medium brown, posterior 1/3 dark brown, segment IX–X medium brown. Sterna greyish brown, posterior 1/3 medium brown. Gills greyish brown to greyish purple. Terminal filaments broken.

##### Description.

**Figure 9.**
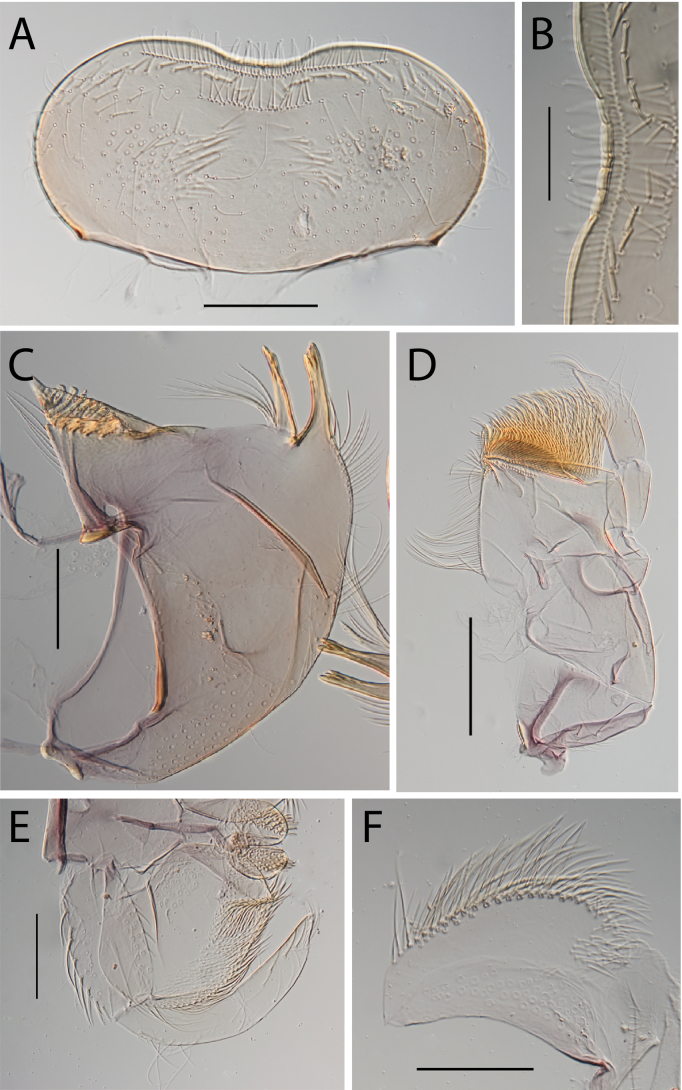
Thraulus (Thraulus) nabire sp. nov. nymphal mouthparts. **A.** Labrum, dorsal view; **B.** Emargination of the labrum; **C.** Right mandible; **D.** Maxilla; **E.** Labium; **F.** Superlingua of hypopharynx. Scale bars: 100 µm (**A, C, E, F**); 50 µm (**B**); 200 µm (**D**).

***Head*. *Labrum*** cordiform (Fig. 9A). Ratio labrum width/clypeus width 0.81–0.90. Ratio labrum width/insertion width 1.15–1.26. Medial emargination shallow with flat denticles. Proximal row of setae present simple. Number of setae on proximal row ~ 19–23. Distal row of setae present, simple. Median part of labrum in ventral view with stout setae in row (Fig. 9B). ***Mandibles*.** Outer margin with middle and distal tuft of setae. Right mandible with eight to nine setae below the mola (Fig. 9C). ***Maxillae*.** Posterior margin of cardo with few hair-like setae and one or two stout and long setae in submarginal position. Proximo-lateral margin of stipes with a single long and stout seta. Apical-ventral row with 15–17 pectinate setae. Maxillary palp segment II 0.88–0.90 as long as segment I. Maxillary palp segment III 0.76–0.85 as long as segment I (Fig. 9D). Stout setae on outer margin of segment I absent. Segment II inner margin with three or four stout setae. Segment II outer margin with 3–5 stout setae. Segment III inner margin with 2–4 stout setae. Segment III 1.86–2.00 × as long as base width. ***Labium*.** Labial palp segment II 0.78–0.81 as long as segment I. Labial palp segment III 0.69–0.74 as long as segment I (Fig. 9E). Segment I inner margin with 8–13 stout setae, outer margin with nine stout setae. Segment II outer margin with three or four stout setae. Segment III dorsal face with four or five stout setae. Segment III 2.86–3.29 × as long as base width. ***Hypopharynx*.** Apex of superlingua somewhat pointed (Fig. 9F).

**Figure 10.**
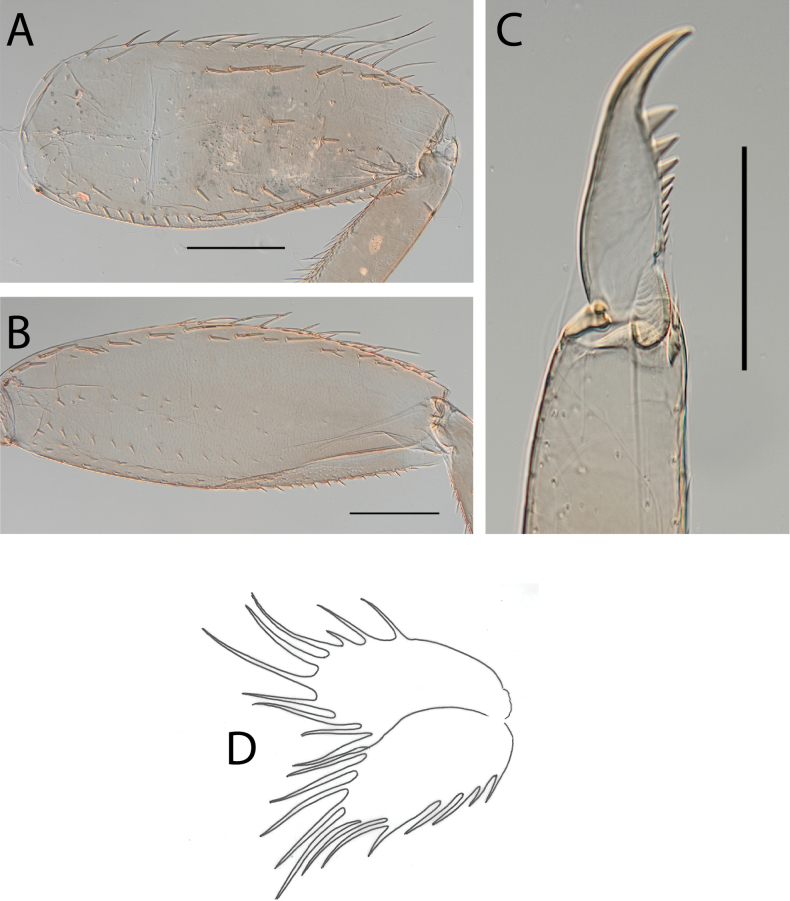
Thraulus (Thraulus) nabire sp. nov. thorax and abdomen. **A.** Fore femur; **B.** Hind femur; **C.** Claw; **D.** Gill IV. Scale bars: 200 µm (**A, B**); 100 µm (**C**).

***Thorax*.** Dorsal margin of femur with a row of long, stout, pointed setae; submarginal row with long setae decreasing in size towards apex (Fig. 10A); ventral margin with short and stout setae and a submarginal row of long and pointed stout setae, except on hind leg where those on ventral margin are short and blunt (Fig. 10B); central area of upper surface with few long and pointed setae. Fore tibia with several rows of numerous mostly feathered setae on ventral margin; outer margin of fore tibia with few hair-like setae; middle tibia with two rows of long and simple or feathered, pointed setae on ventral margin, outer margin with numerous hair-like setae; hind tibia with a row of simple short thin setae on ventral margin, upper and lower surfaces covered with simple and feathered setae, outer margin with short and long, pointed, stout setae. Tarsal claw hooked with eight or nine denticles progressively larger, except distal one much larger (Fig. 10C).

***Abdomen*.** Gill I with single bifid lamella. Gills II–VII with dorsal and ventral lamellae, dorsal lamella smaller. Gill II–VI with upper lamella lacking filaments on basal half of outer margin, with < 20 short filaments, lower lamella with < 20 short filaments (Fig. 10D). Posterior margin of terga IX with denticles. Posterior margin of terga VIII without denticles, or with minute denticles. Posterior margin of terga V–VII without denticles. Posterolateral projections present on segments VI–IX.

##### Derivatio nominis.

This species is named after the district of Nabire where it can be found and is a noun in apposition.

#### ﻿ Thraulus (Thraulus) noe sp. nov.

Taxon classificationAnimaliaEphemeropteraLeptophlebiidae

F817B76E-7248-5635-A839-B78534428E89

https://zoobank.org/D44BDA28-13FE-4DD1-9274-4FD03E421881

**Figure 11.**
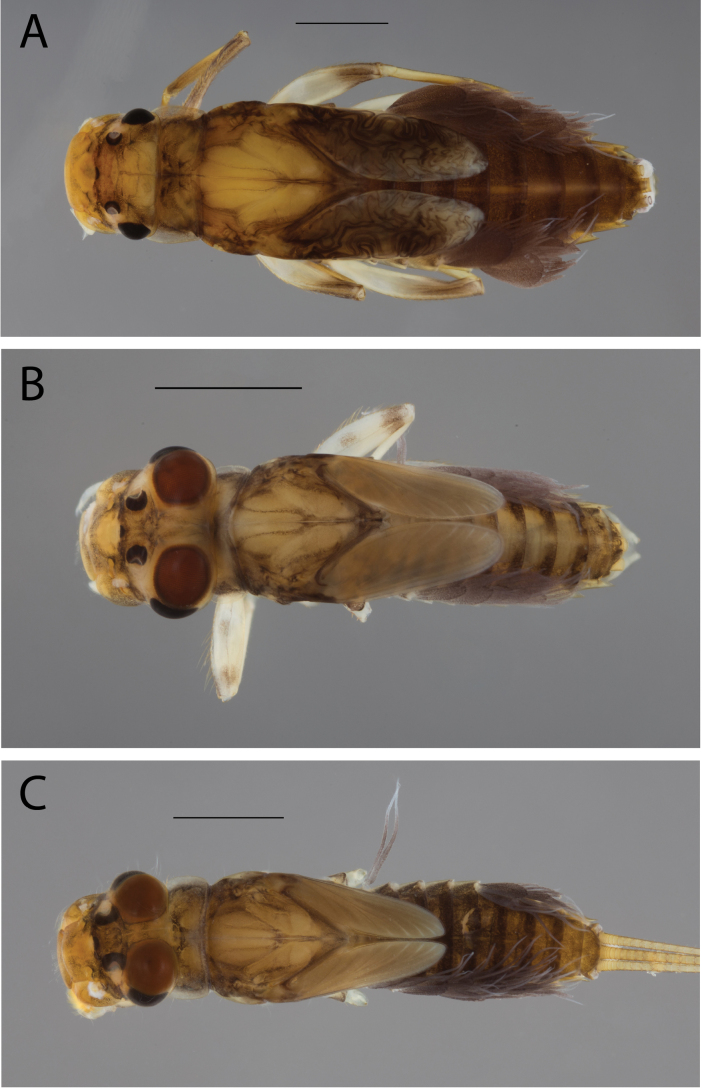
Habitus of Thraulus (Thraulus) noe sp. nov. (**A**), Thraulus (Thraulus) ogea sp. nov. (**B**) and Thraulus (Thraulus) wemale sp. nov. **(C).** Scale bars: 1 mm.

**Figure 12.**
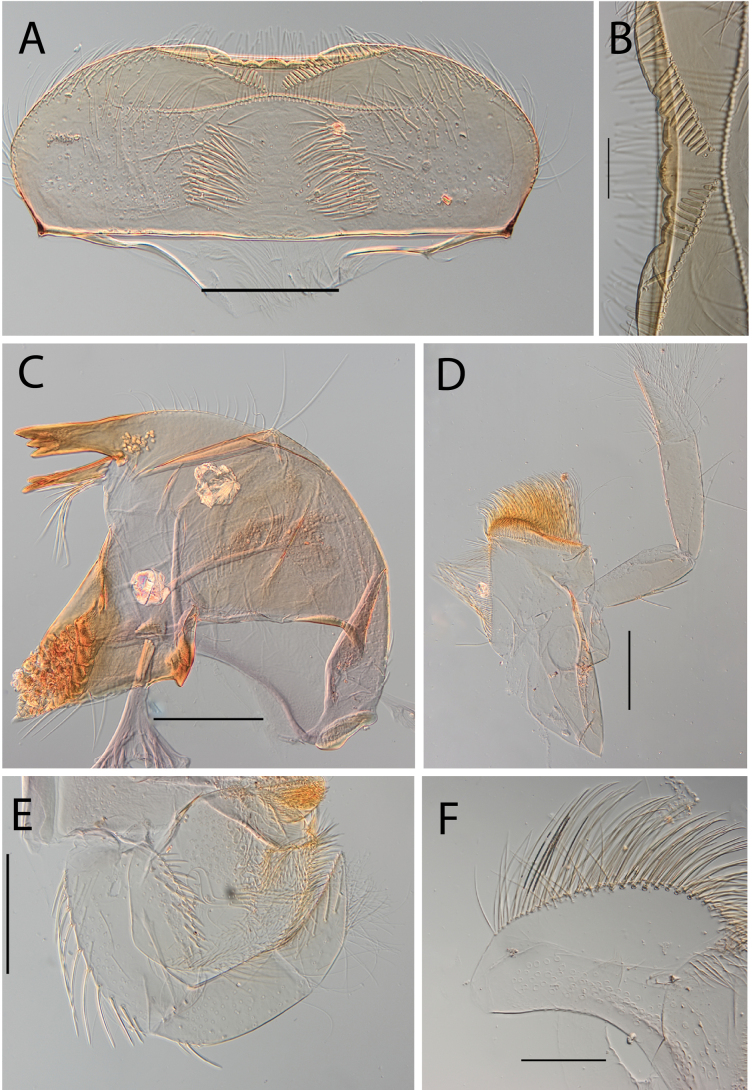
Thraulus (Thraulus) noe sp. nov. nymphal mouthparts. **A.** Labrum, dorsal view; **B.** Emargination of the labrum; **C.** Right mandible; **D.** Maxilla **E.** Labium **F.** Superlingua of hypopharynx. Scale bars: 200 µm (**A, C–E**); 50 µm (**B**); 100 µm (**F**).

**Figure 13.**
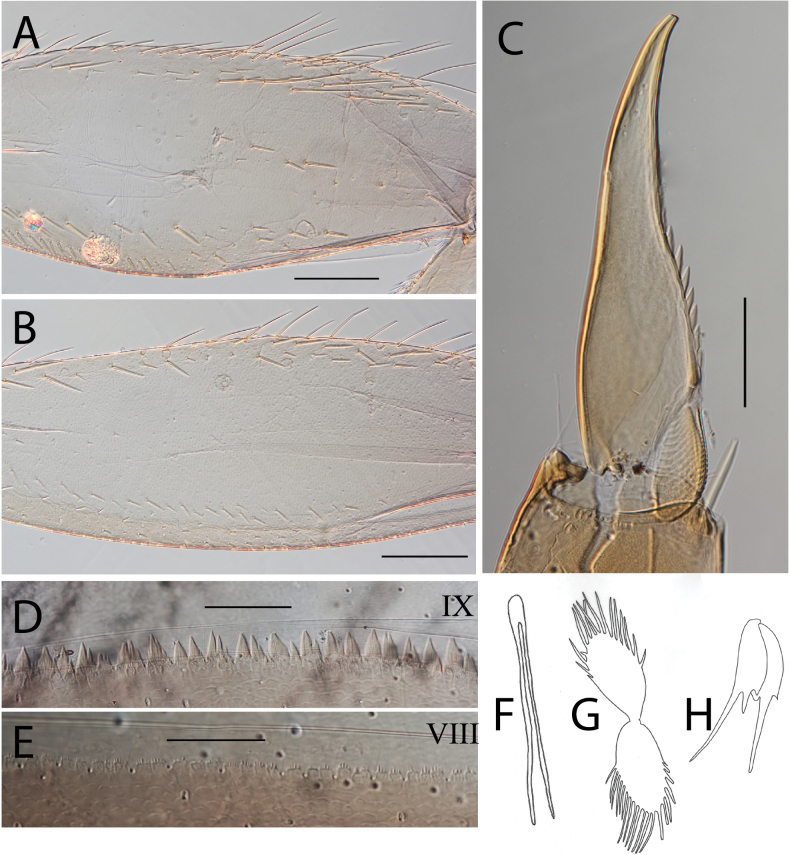
Thraulus (Thraulus) noe sp. nov. thorax and abdomen. **A.** Fore femur; **B.** Hind femur; **C.** Claw; **D.** Posterior margin of tergite IX; **E.** Posterior margin of tergite VIII; **F.** Gill I; **G.** Gill IV, **H.** Gill VII. Scale bars: 200 µm (**A, B**); 50 µm (**C–E**).

Figs 11A , 12 , 13 , 20A

##### Material examined.

***Holotype*.** • Papua New Guinea, nymph on slide, Central Province, Woitape, 700 m, I.2008, 8°31.290'S, 147°13.684'E, Posman col [PNG166], GBIFCH01223114 (ZSM). ***Paratypes*.** • Papua New Guinea, 2 nymphs in ethanol (UFVB), 8 nymphs in ethanol, GBIFCH01523475, 3 entirely mounted on slide, GBIFCH01223113, GBIFCH01223112, GBIFCH01223111, same data as holotype (MZL). *Other material.* • Papua New Guinea, one nymph on slide, GBIFCH01223109, Central Province, Kokoda Trek, 1390 m, I. 2008, 09°00.338'S, 147°44.252'E. Posman col [PNG173] (MZL). • Papua New Guinea, one nymph on slide, GBIFCH01223110, Eastern Highlands Province, Marawaka, Ande, 1700–1800 m, 9.XI.2006, approx. 07°01.697'S, 145°49.807'E, M. Balke & Kinibel col [PNG87] (MZL).

##### Nymph.

Body length, male: 5.1–5.2 mm Body length, female: 6.3–6.5 mm.

##### Diagnosis.

Labrum rectangular, with emargination narrow; proximo-lateral margin of stipes with one long and stout seta together with two small and stout setae; maxillary palp greatly elongated; outer margin of maxillary palp segment II with 1–3 stout setae; inner margin of labial palp segment I with 20–26 stout setae; apex of superlingua rounded. Gills II–VI without filaments on basal half, with ~ 15 filaments on dorsal lamella and ~ 20 on ventral lamella; posterior margin of abdominal segment VIII with minute denticles.

##### Colouration.

***Head*.** Vertex greyish brown, darker between ocelli. Antenna yellowish, pedicel greyish. Compound eye lower portion black, upper portion reddish brown. Mouth parts and clypeus medium brown. ***Thorax*.** Pronotum greyish brown. Mesonotum pale brown washed with greyish brown laterally (Fig. 11A). Metanotum dark brown. Femur: forefemur almost completely greyish brown; mid- and hind femora whitish, apical 1/3 brownish. Tibia yellowish, apex yellowish brown. Tarsi yellowish brown. ***Abdomen*.** Terga dark brown, posterior margin blackish. Sterna yellowish brown in females to dark brown in males. Gills purplish brown. Terminal filaments uniformly yellowish brown.

##### Description.

***Head*. *Labrum*** rectangular (Fig. 12A). Ratio labrum width/clypeus width 0.88–0.92. Ratio labrum width/insertion width 1.12–1.13. Medial emargination narrow with rounded denticles. Proximal row of setae present, simple. Number of setae on proximal row 69–87. Distal row of setae present, simple. Median part of labrum in ventral view with stout setae in a row (Fig. 12B). ***Mandibles*.** Outer margin with row of setae on distal 2/3, row ending before incisor. Right mandible with 7–10 setae below the mola (Fig. 12C). ***Maxillae*.** Posterior margin of cardo broken. Proximo-lateral margin of stipes with one long stout seta together with two small stout setae. Apical-ventral row with 15–17 pectinate setae. Maxillary palp extremely long, almost 2 × longer than maxilla; segment II 1.3–1.8 × as long as segment I. Maxillary palp segment III 0.77–0.99 as long as segment 1 (Fig. 12D). Stout setae on outer margin of segment I present. Segment II inner margin with two stout setae, outer margin with 1–3 stout setae. Segment III inner margin with three or four stout setae, dorsal surface with few setae at apex. Segment III 2.46–3.00 × as long as base width. ***Labium*.** Labial palp segment II 0.67–0.71 as long as segment 1. Labial palp segment III 0.63–0.70 as long as segment 1 (Fig. 12E). Segment I inner margin with 20–26 stout setae, outer margin with 12–15 stout setae. Segment II outer margin with two or three stout setae. Segment III dorsal face with six stout setae. Segment III 2.15–2.29 × as long as base width. ***Hypopharynx*.** Apex of superlingua rounded (Fig. 12F).

***Thorax*.** Dorsal margin of femur with a row of long stout setae, and a submarginal row of same setae; ventral margin with shorter and stouter setae (Fig. 13A, B); upper surface with few long and pointed but stout setae. Tarsal claw straight with 4–7 subequal denticles (Fig. 13C).

***Abdomen*.** Gill I with single bifid lamella (Fig. 13F). Gills II–VII with dorsal and ventral lamellae, dorsal lamella smaller. Gills II–VI with ~ 15 filaments on upper lamella and ~ 20 on the ventral one, filamentous projections lacking on proximal half (Fig. 13G); gill VII with three filaments on each lamella, one much longer than the other two (Fig. 13H). Posterior margin of terga IX with denticles (Fig. 13D). Posterior margin of terga VIII with minute denticles (Fig. 13E). Posterior margin of terga V–VII without denticles. Posterolateral projections present only on segment VIII–IX.

***Eggs*.** General shape elongated. Size 190 μm × 75 μm. Chorionic surface smooth, with longitudinal ridges weakly elevated, interrupted partially in the equatorial area. No attachment structures. Micropyle in equatorial position (Fig. 20A).

##### Derivatio nominis.

This species is dedicated to Noe Sartori, grandson of the first author, and is a noun in apposition.

#### ﻿ Thraulus (Thraulus) ogea sp. nov.

Taxon classificationAnimaliaEphemeropteraLeptophlebiidae

820127E1-D2DE-59DE-99F2-15EE7EBEADB7

https://zoobank.org/2BDF258C-A3F5-45ED-84A1-6D29BBBDFC80

**Figure 14.**
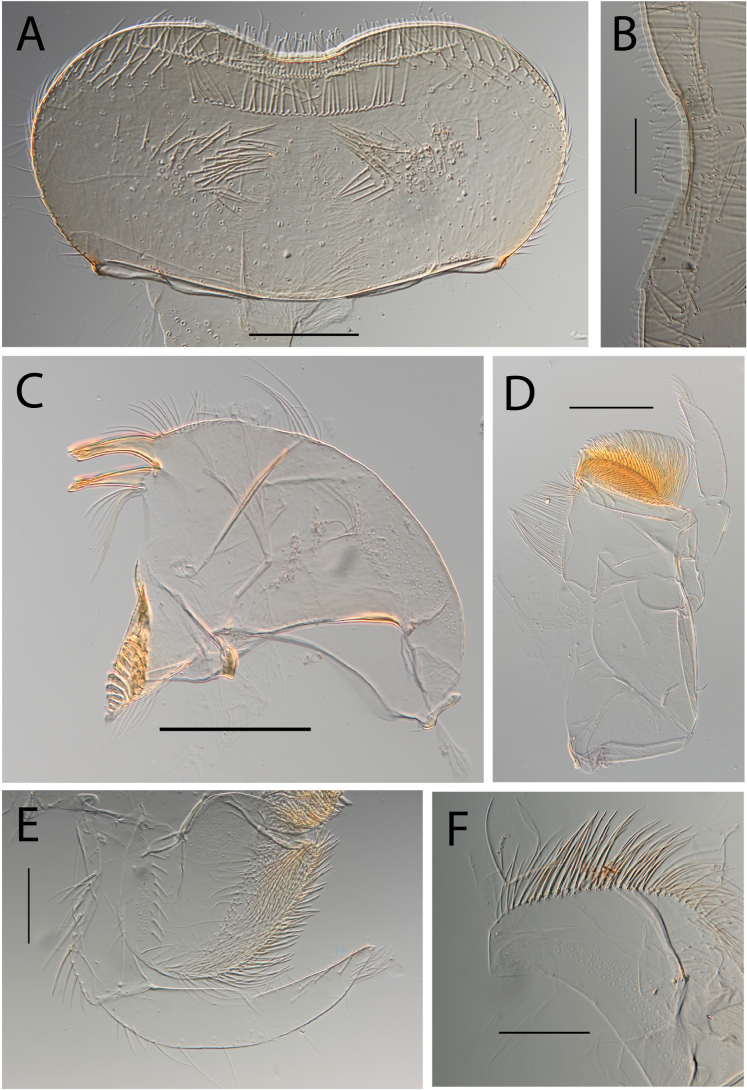
Thraulus (Thraulus) ogea sp. nov. nymphal mouthparts. **A.** Labrum, dorsal view; **B.** Emargination of the labrum; **C.** Right mandible; **D.** Maxilla **E.** Labium; **F.** Superlingua of hypopharynx. Scale bars: 100 µm (**A, C, E, F**); 50 µm (**B**); 200 µm (**D**).

**Figure 15.**
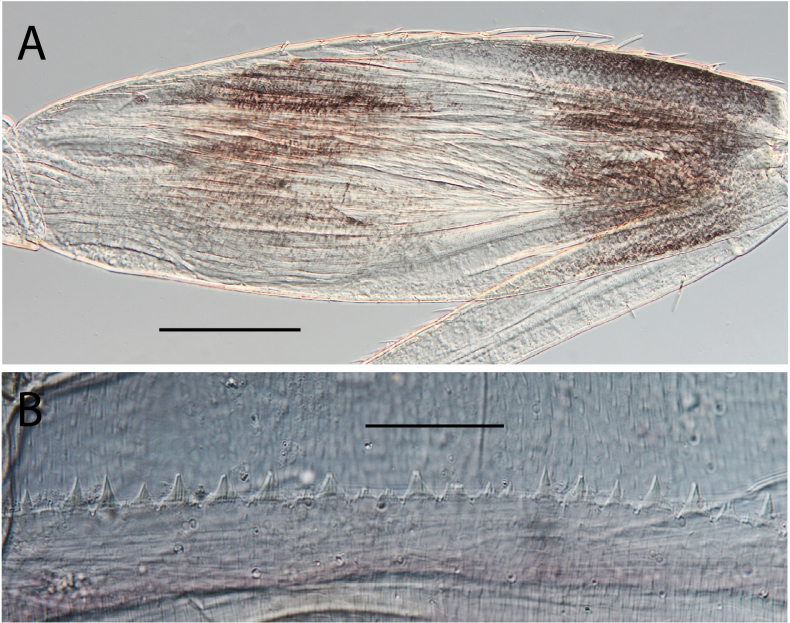
Thraulus (Thraulus) ogea sp. nov. thorax and abdomen. **A.** Hind femur; **B.** Posterior margin of tergite IX. Scale bars: 200 µm (**A**); 50 µm (**B**).

Figs 11B , 14 , 15 , 20B–D

##### Material examined.

***Holotype*.** • Papua New Guinea, nymph on slide, Madang Province, Trans Gogol, 30 m, II.2008, 5°18.0915'S, 145°36.4532'E, BRC col [PNG179], GBIFCH01223108 (ZSM). ***Paratypes*.** • Papua New Guinea, 4 nymphs in ethanol (UFVB), 22 nymphs in ethanol, GBIFCH01513476, 2 nymphs entirely mounted on slides, GBIFCH01223107, GBIFCH01223106, same data as holotype (MZL).

##### Nymph.

Body length, male: 3.6–3.9 mm Body length, female: 4.2–4.4 mm.

##### Diagnosis.

Labrum cordiform, medial emargination shallow with flat denticles; median part of labrum in ventral view with stout setae in a row; outer margin of mandibles with tuft of setae in medium and distal positions; posterior margin of cardo with few hair-like setae; gill lamellae on segments II–VI without filaments on basal half, with < 20 filaments on each; posterolateral projections of the abdomen present on segments VII–IX.

##### Colouration.

***Head*.** Pale brown, greyish brown between ocelli and eyes. Antenna whitish. lower portion black, upper portion reddish brown, outer ommatidia dark brown. Mouth parts: clypeus greyish brown. ***Thorax*.** Pronotum greyish brown, with two pale brown markings medially. Mesonotum greyish brown, washed with dark brown laterally and medially (Fig. 11B). Metanotum greyish brown. Femur yellowish brown, with one pale brown marking in the middle and one distally. Tibia uniformly yellowish. Tarsi uniformly yellowish. ***Abdomen*.** Terga medium brown, posterior margin dark brown. Sterna yellowish grey. Gills purplish brown. Terminal filaments uniformly yellowish white.

##### Description.

***Head*. *Labrum*** cordiform (Fig. 14A). Ratio labrum width/clypeus width 0.93–0.94. Ratio labrum width/insertion width 1.29–1.30. Medial emargination shallow with flat denticles. Proximal row of setae present, simple. Number of setae on proximal row 35–47. Distal row of setae present, simple. Median part of labrum in ventral view with only scattered stout setae (Fig. 14B). ***Mandibles*.** Outer margin with middle tuft of setae, and with distal tuft of setae. Right mandible with nine or ten setae below the mola (Fig. 14C). ***Maxillae*.** Posterior margin of cardo with few hair-like setae. Proximo-lateral margin of stipes with a single stout and medium-sized seta. Apical-ventral row with 25–29 pectinate setae. Maxillary palp segment II 0.77–1.05 as long as segment I. Maxillary palp segment III 0.69–0.73 as long as segment I (Fig. 14D). Stout setae on outer margin of segment I absent. Segment II inner margin with five stout setae, outer margin with five or six stout setae. Segment III inner margin with three stout setae, dorsal surface with few setae at apex. Segment III 1.89–2.57 × as long as base width. ***Labium*.** Labial palp segment II 0.81–0.82 as long as segment I. Labial palp segment III 0.71–0.81 as long as segment I (Fig. 14E). Segment I inner margin with 8–11 stout setae, outer margin with 12 or 13 stout setae. Segment II outer margin with four or five stout setae. Segment III, dorsal face with three or four stout setae. Segment III 3.0–3.57 as long as base width. ***Hypopharynx*.** Apex of superlingua truncate (Fig. 14F).

***Thorax*.** Dorsal margin of femur with a row of stout and long setae, and a submarginal row with same setae; ventral margin with shorter and stouter setae; upper surface with few long and pointed stout setae (Fig. 15A). Tarsal claw hooked with 4–6 denticles progressively larger apically.

***Abdomen*.** Gill I with single bifid lamella. Gills II–VII with dorsal and ventral lamellae, dorsal lamella smaller. Gill II to VI with seven to ten filaments on upper lamella and 12–14 on the ventral one, filamentous projections lacking on proximal half; gill VII with three or four filaments on each lamella. Posterior margin of terga IX with denticles. Posterior margin of terga IX with denticles (Fig. 15B). Posterior margin of terga VIII with minute denticles. Posterior margin of terga V–VII without denticles. Posterolateral projections present on segments VII–IX.

***Eggs*.** General shape elongated. Size 190 μm × 75 μm. Chorionic surface smooth and uplifted compared to attachment structures (Fig. 20B). Attachment structures composed of nine long slender filaments attached to each pole (Fig. 20C); presence of two transverse filaments at the micropyle opening (Fig. 20D). Micropyle in equatorial position.

##### Derivatio nominis.

This species is named after the ethnic group Ogea, who live close to the Gogol River, and is a noun in apposition.

#### ﻿ Thraulus (Thraulus) timorensis sp. nov.

Taxon classificationAnimaliaEphemeropteraLeptophlebiidae

3AB9A5D3-50E4-55A3-9265-4BA483FF654D

https://zoobank.org/930B6137-34C5-4AFD-B744-9AF48144D4EF

Figs 16 , 17 , 34E

##### Material examined.

***Holotype*.** • Indonesia, Timor, nymph on slide, GBIFCH01223095, some gills in ethanol, GBIFCH01523478, Naikliu area, restpools in dry forest,130 m, 03.X.2011, 09°58.425'S, 123°41.439'E, M. Balke col [TIM11] (MZB).

##### Nymph.

Body length, female: 6.5 mm.

##### Diagnosis.

Labrum cordiform, medial emargination shallow without denticles; dorsal face of labrum with multiple rows in distal position; tarsal claw hooked with 15 teeth and a palisade of four other teeth in distal position; gill lamellae on segments II–VI with > 25 filaments each; posterolateral projections of the abdomen present on segments VIII and IX.

##### Colouration.

Femur, tibia, and tarsi whitish.

##### Description.

**Figure 16.**
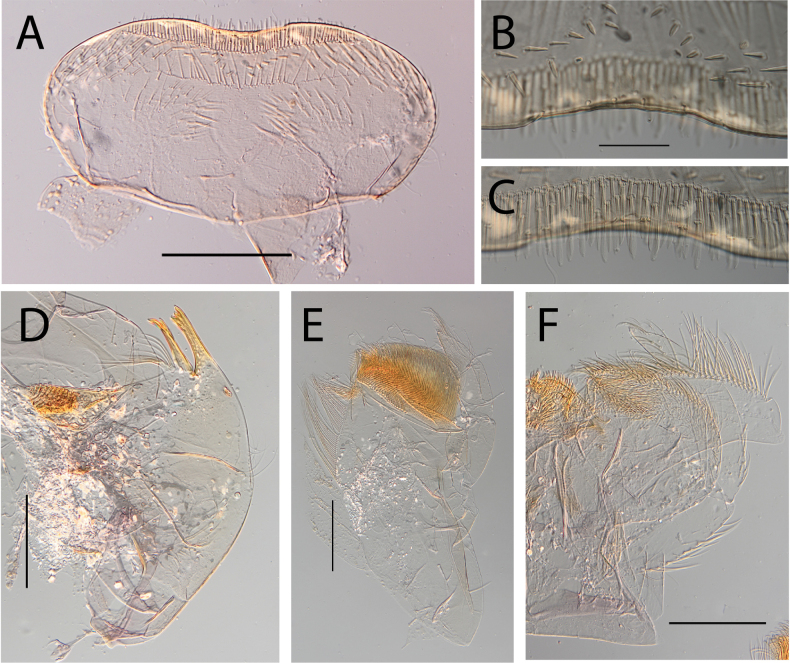
Thraulus (Thraulus) timorensis sp. nov. nymphal mouthparts. **A.** Labrum, dorsal view; **B.** Emargination of the labrum; **C.** Detail of the distal row of setae; **D.** Right mandible; **E.** Maxilla; **F.** Labium Scale bars: 200 µm (**A, D–F**); 50 µm (**B, C**).

***Head*. *Labrum*** cordiform (Fig. 16A). Ratio labrum width/clypeus width 0.95. Ratio labrum width/insertion width 1.39. Medial emargination shallow without denticles. Proximal row of setae present, simple. Number of setae on proximal row ~ 29. Distal row of setae multiple (Fig. 16C). Median part of labrum in ventral view with stout setae in a row (Fig. 16B). ***Mandibles*.** Outer margin with middle tuft of setae. Right mandible with ten setae below mola (Fig. 16D). ***Maxillae*.** Posterior margin of cardo broken. Proximo-lateral margin of stipes with single long stout seta (Fig. 34E). Apical-ventral row with 22 pectinate setae. Maxillary palp segment II 0.82 as long as segment I. Maxillary palp segment III 0.78 as long as segment I (Fig. 16E). Stout setae on outer margin of segment I absent. Segment II inner margin with five stout setae, outer margin without stout setae. Segment III inner margin with five stout setae, dorsal surface with few setae at apex. Segment III 1.67 × as long as base width. ***Labium*.** Labial palp segment II 0.76 as long as segment I. Labial palp segment III 0.76 as long as segment I (Fig. 16F). Segment I inner margin with two stout setae, outer margin with 12 stout setae. Segment II outer margin with ten stout setae. Segment III dorsal face with five stout setae. Segment III 2.90 × as long as base width. ***Hypopharynx*.** Apex of superlingua truncate (Fig. 16F).

**Figure 17.**
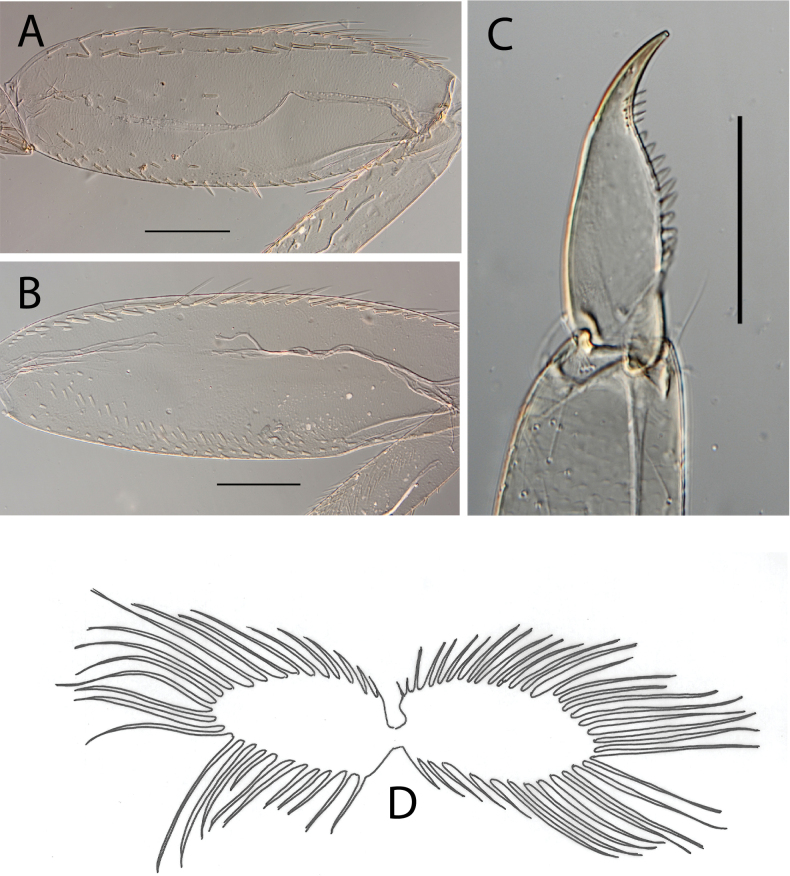
Thraulus (Thraulus) timorensis sp. nov. thorax and abdomen. **A.** Fore femur; **B.** Hind femur; **C.** Claw; **D.** Gill IV. Scale bars: 200 µm (**A, B**); 50 µm (**C**).

***Thorax*.** Dorsal margin of femur with a row of long, stout and pointed setae; regular submarginal row with setae as long as those of dorsal margin (Fig. 17A); ventral margin with short and pointed setae and a submarginal row of short and blunt but stout setae, except on hind leg where those on ventral margin are short and blunt (Fig. 17B); central area of upper surface with few long pointed setae. Middle tibia with two rows of long pointed setae on ventral margin, outer margin with few hair-like setae; hind tibia with a row of long thin setae on ventral margin, upper and lower surfaces covered with simple and some feathered setae, outer margin with long and short pointed stout setae. Tarsal claw hooked with 15 denticles, together with a palisade of four small denticles at apex (Fig. 17C).

***Abdomen*.** Gill I with single bifid lamella. Gills II–VII with dorsal and ventral lamellae, dorsal lamella smaller. Gills II–VI with upper lamella entirely bordered with ~ 35 very long filaments, lower lamella with ~ 30 very long filaments (Fig. 17D); gill VII smaller but with the same number of long filaments. Posterior margin of terga IX with denticles. Posterior margin of terga VIII with minute denticles. Posterior margin of terga V–VII without denticles. Posterolateral projections present on segments VIII–IX.

##### Derivatio nominis.

This species is named after the island from where it comes. Although we have a single nymph in hand, we think important to formally named it, since it possesses distinct characters; moreover, it is the first Leptophlebiidae described from this island and only the second mayfly species (Kaltenbach and Gattolliat 2017).

#### ﻿ Thraulus (Thraulus) wemale sp. nov.

Taxon classificationAnimaliaEphemeropteraLeptophlebiidae

0082BB0C-EE8F-526C-B02C-82AC58D055F4

https://zoobank.org/ACB801CB-1E07-4D9F-B65C-E9203F46D7FB

**Figure 18.**
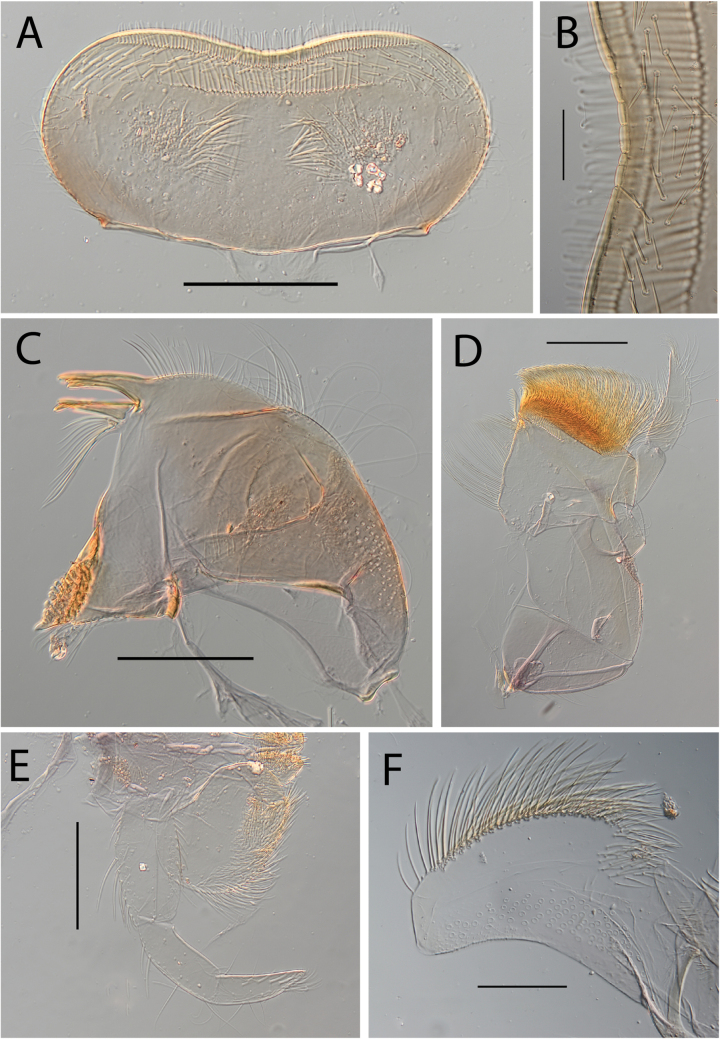
Thraulus (Thraulus) wemale sp. nov. nymphal mouthparts. **A.** Labrum, dorsal view; **B.** Emargination of the labrum; **C.** Right mandible; **D.** Maxilla; **E.** Labium; **F.** Superlingua of hypopharynx. Scale bars: 200 µm (**A, C–E**); 50 µm (**B**); 100 µm (**F**).

**Figure 19.**
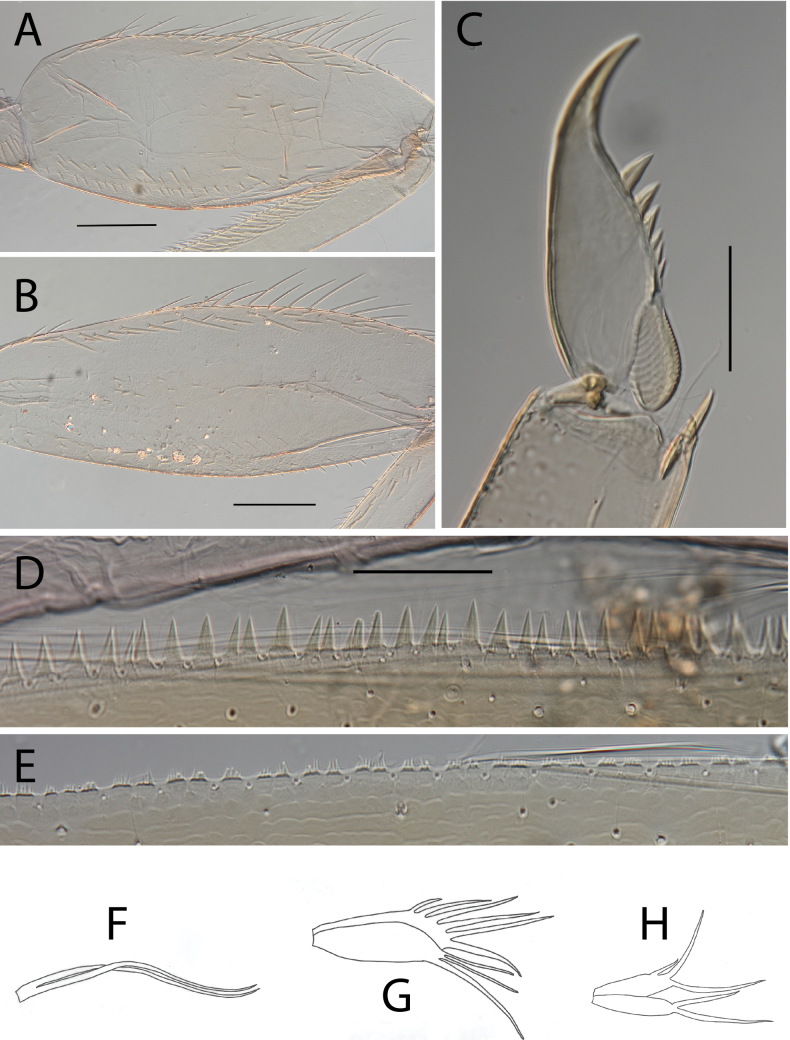
Thraulus (Thraulus) wemale sp. nov. thorax and abdomen. **A.** Fore femur; **B.** Hind femur; **C.** Claw; **D.** Posterior margin of tergite IX; **E.** Posterior margin of tergite VIII; **F.** Gill I; **G.** Gill IV; **H.** Gill VII. Scale bars: 200 µm (**A, B**); 50 µm (**C–E**).

**Figure 20.**
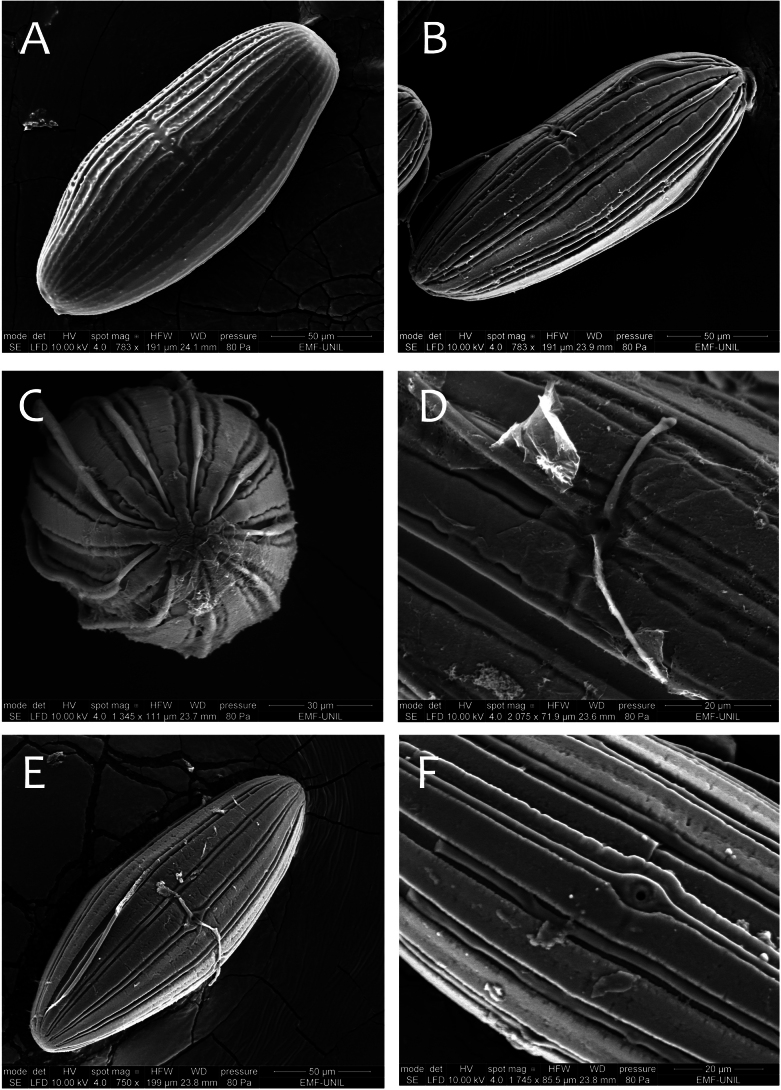
Egg structure in toto (**A, B, E**), polar view (**C**), detail of chorion and micropyle (**D, F**). **A.**Thraulus (Thraulus) noe sp. nov.; **B–D.**Thraulus (Thraulus) ogea sp. nov.; **E, F.**Thraulus (Thraulus) wemale sp. nov.

Figs 11C , 18 , 19 , 20E, F

##### Material examined.

***Holotype*.** • Indonesia, Maluku, Seram, nymph on slide, Kanikeh, 607 m, 7.IV.2012, 3°06.524'S / 129°28.796E, M. Balke col [AMB07], GBIFCH01223099 (MZB). ***Paratypes*.** • Indonesia, Maluku, Seram, 9 nymphs in ethanol, GBIFCH01523477, 2 entirely mounted on slide, GBIFCH01223105, GBIFCH01223102, same data as holotype (MZL). • Indonesia, Maluku, Seram, 1 nymph in ethanol (UFVB), 2 entirely mounted on slide, GBIFCH01223104, GBIFCH01223103, Roho-Kanikeh village, waterholes near Roho, 117 m, 7.IV.2012, 3°01.193'S / 129°23.480'E, M. Balke col [AMB06] (MZL). • Indonesia, Maluku, Seram, 1 nymph entirely mounted on slide, GBIFCH01223100, small puddles Kanikeh-Wainsela, 800–1350 m, 9.IV.2012, 3°08.679'S / 129°28.403'E, M. Balke col [AMB10] (MZL). • Indonesia, Maluku, Seram, 1 nymph entirely mounted on slide, GBIFCH01223101, near Huahulo village, 110 m, 13.IV.2012, 2°59.069'S / 129°20.539'E, M. Balke col. [AMB13] (MZL). • Indonesia, Maluku, Seram, 1 nymph entirely mounted on slide, GBIFCH01223149, Gunung Salahutu, stream, shaded at waterfall, 125 m, 3.IV.2012, 3°33.318'S / 128°17.711'E, M. Balke col. [AMB03] (MZL).

##### Nymph.

Body length, male: 4.8–5 mm, female: 5.7–6 mm.

##### Diagnosis.

Labrum cordiform, medial emargination shallow with flat denticles; median part of labrum in ventral view with stout setae in a row; outer margin of mandibles with row of setae on distal 2/3; posterior margin of cardo with many hair-like setae and one stout long seta in submarginal position; gill lamellae on segments II–VI with < 20 filaments, without filaments on basal half ; posterolateral projections of the abdomen present on segments VI–IX.

##### Colouration.

***Head*.** Pale brown, darker between ocelli in males, greyish brown in females. Antenna whitish. Compound eye lower portion black, upper portion reddish brown. Mouth parts pale brown to greyish brown. ***Thorax*.** Pronotum medium brown, darker in anterior part and laterally (Fig. 11C). Mesonotum whitish brown, darker laterally, with greyish marks along the median suture. Metanotum pale brown. Femur greyish white, dorsal surface with two large dark grey marks proximally and distally. Tibia uniformly pale brown. Tarsi uniformly pale brown. ***Abdomen*.** Terga uniformly medium to dark brown, tergites IX and X paler in males. Sterna yellowish grey. Gills purplish brown. Terminal filaments uniformly pale brown.

##### Description.

***Head*. *Labrum*** cordiform (Fig. 18A). Ratio labrum width/clypeus width 0.96–1.03. Ratio labrum width/insertion width 1.31–1.39. Medial emargination shallow with flat denticles. Proximal row of setae present, simple. Number of setae on proximal row 33–45. Distal row of setae present, simple. Median part of labrum in ventral view with only scattered stout setae (Fig. 18B). ***Mandibles*.** Outer margin with row of setae on distal 2/3 ending near incisor. Right mandible with 7–11 setae below the mola (Fig. 18C). ***Maxillae*.** Posterior margin of cardo with many hair-like setae and one stout long seta in submarginal position. Proximo-lateral margin of stipes with a single stout long seta. Apical-ventral row with 18–23 pectinate setae. Maxillary palp segment II 0.70–1.00 × as long as segment I. Maxillary palp segment III 0.67–0.82 as long as segment 1 (Fig. 18D). Stout setae on outer margin of segment I absent. Segment II inner margin with four or five stout setae, outer margin with 4–6 stout setae. Segment III inner margin with 4–6 stout setae, dorsal surface with few setae at apex. Segment III 1.8–2.22 × as long as base width. ***Labium*.** Labial palp segment II 0.72–0.90 as long as segment I. Labial palp segment III 0.67–0.78 as long as segment I (Fig. 18E). Segment I inner margin with 9–13 stout setae, outer margin with 11 or 12 stout setae. Segment II outer margin with 3–5 stout setae. Segment III dorsal face with 3–5 stout setae. Segment III 3.20–3.57 × as long as base width. ***Hypopharynx*.** Apex of superlingua truncate (Fig. 18F).

***Thorax*.** Dorsal margin of femur with a row of stout, long setae, and a submarginal row with same setae; ventral margin with shorter and stouter setae; upper surface with few long, pointed stout setae (Fig. 19A, B). Tarsal claw hooked with 4–7 denticles progressively larger apically (Fig. 19C).

***Abdomen*.** Gill I with single bifid lamella (Fig. 19F). Gills II–VII with dorsal and ventral lamellae, dorsal lamella smaller. Gills II–VI with < 10 filaments on each lamella (Fig. 19G); gill VII with two or three filaments on each lamella (Fig. 19H). Posterior margin of terga IX with denticles (Fig. 19D). Posterior margin of terga VII–VIII with minute denticles (Fig. 19E). Posterior margin of terga V–VI without denticles. Posterolateral projections present on segment VI–IX.

***Eggs*.** General shape elongated. Size 190 μm x 75 μm. Chorionic surface smooth and uplifted compared to attachment structures. Attachment structures composed of 12 long and slender filaments attached to each pole and able to inflate (Fig. 20E). At the micropyle opening, presence of two transverse short filaments, easily broken (Fig. 20F). Micropyle in equatorial position.

##### Derivatio nominis.

This species is named to honour the ancient ethnic group Wemale, who still inhabits the inland of Seram where the specimens of this species were collected and is a noun in apposition.

#### ﻿ Thraulus (Thraulus)

Taxon classificationAnimaliaEphemeropteraLeptophlebiidae

sp. A

8D89F12E-C98C-5C03-93F5-5C596E757A7B

Figs 21 , 22 , 23

##### Material examined.

• Papua New Guinea, nymph on slide, Madang Province, Keki, Adalbert Mts, 400 m, 29.XI.2006, 04°43.058'S, 145°24.437'E, Binatang Boys col [PNG119], GBICH01223091 (MZL).

##### Diagnosis.

**Nymph.** Labrum trapezoidal; ventral side of the labrum with only scattered stout setae; labial palp with segment III > 4 × as long as base width. Posterior margin of abdominal segment VI–VII with well-developed denticles.

##### Colouration.

Femur whitish, with distal 1/3 greyish; forelegs greyish along the dorsal margin. Tibia whitish, apex greyish. Tarsi yellowish.

##### Description.

**Figure 21.**
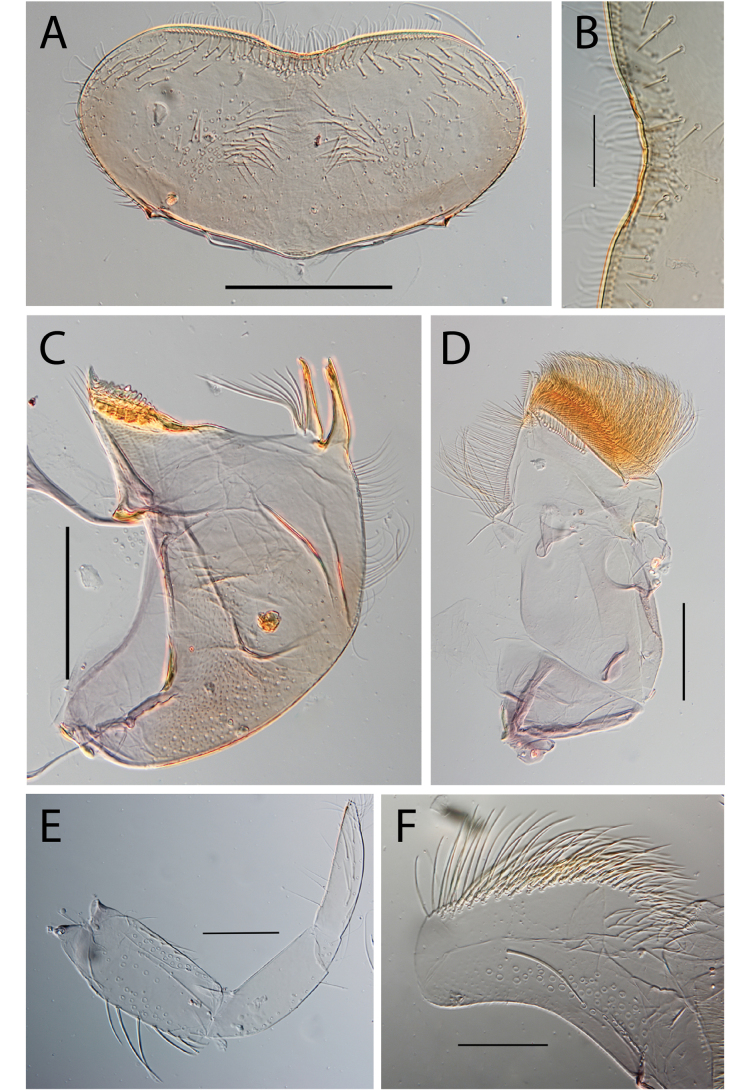
Thraulus (Thraulus) sp. **A.** Nymphal mouthparts. **A.** Labrum, dorsal view; **B.** Emargination of the labrum; **C.** Right mandible; **D.** Maxilla; **E.** Labium; **F.** Superlingua of hypopharynx. Scale bars: 200 µm (**A, C, D**); 50 µm (**B**); 100 µm (**E, F**)

***Head*. *Labrum*** trapezoid (Fig. 21A). Ratio labrum width/clypeus width 1.07. Ratio labrum width/insertion width 1.52. Medial emargination shallow with flat denticles. Proximal row of setae simple, shifted to distal margin (Fig. 21B). Number of setae on proximal row ~ 30. Distal row of setae present, simple. Median part of labrum in ventral view with only scattered stout setae. ***Mandibles*.** Outer margin with row of setae on distal 2/3. Ending of the row before incisor. Right mandible with eight setae below the mola (Fig. 21C). ***Maxillae*.** Posterior margin of cardo with very few hair-like setae and a long and stout seta in submarginal position. Proximo-lateral margin of stipes with a single long and stout seta. Apical-ventral row with 26 pectinate setae. Maxillary palps lost (Fig. 21D). ***Labium*.** Labial palp segment II 0.87 as long as segment I. Labial palp segment III 0.89 as long as segment I (Fig. 21E). Segment I inner margin with five stout setae, outer margin with eight stout setae. Segment II outer margin with two stout setae. Segment III dorsal face with eight stout setae. Segment III 4.40 × as long as width base. ***Hypopharynx*.** Apex of superlingua truncate (Fig. 21F).

**Figure 22.**
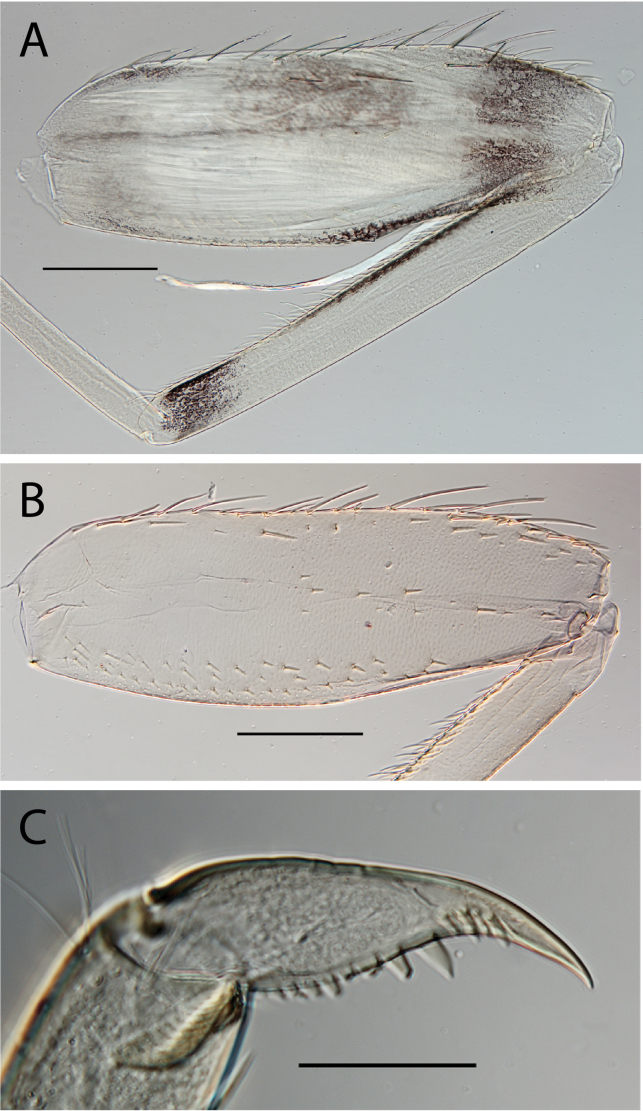
Thraulus (Thraulus) sp. A. Thorax. **A.** Fore femur; **B.** Mid femur; **C.** Claw. Scale bars: 200 µm (**A, B**); 50 µm (**C**).

***Thorax*.** Dorsal margin of femur with a row of long, stout, pointed setae (Fig. 22A); irregular submarginal row on fore femur, regular on mid femur (Fig. 22B), absent on hind femur; ventral margin with short, stout setae and a submarginal row of long, pointed stout setae, except on hind leg where those on ventral margin are short and blunt; central area of upper surface with few long, pointed setae. Fore tibia with several rows of numerous mostly feathered setae on ventral margin; outer margin of fore tibia with few hair-like setae; middle tibia with two rows of long, simple or feathered, pointed setae on ventral margin, outer margin with few hair-like setae; hind tibia with a row of simple short, thin setae on ventral margin, upper and lower surface covered with simple and feathered setae, outer margin with short and long pointed stout setae. Tarsal claw straight with eight or nine denticles progressively larger, with a palisade of four small denticles at apex (Fig. 22C).

**Figure 23.**
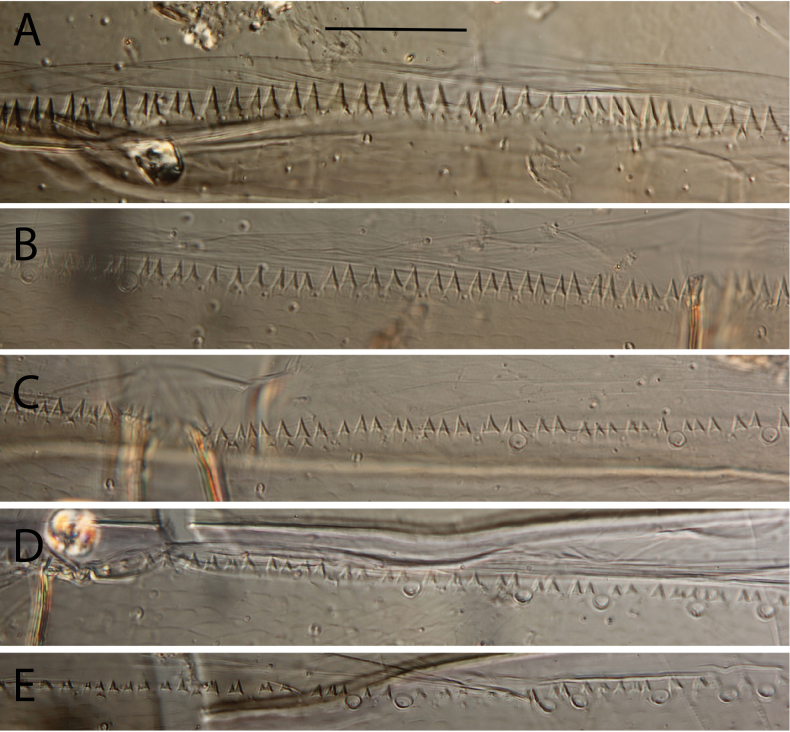
Thraulus (Thraulus) sp. A. Abdomen. **A.** Posterior margin of tergite IX; **B.** Posterior margin of tergite VIII; **C.** Posterior margin of tergite VII; **D.** Posterior margin of tergite VI; **E.** Posterior margin of tergite V. Scale bar: 50 µm.

***Abdomen*.** Gills lost. Posterior margin of terga VI–IX with denticles (Fig. 23A–D). Posterior margin of terga V with minute denticles (Fig. 23E). Posterolateral projections present on segments VII–IX.

### ﻿Differential diagnoses among species of the subgenus Thraulus

New Guinean species can be identified with the key provided at the end of this work. Three species present peculiar gills, with filaments only present in the distal half of each lamella. To our knowledge, this character has never been reported on other *Thraulus* species, although some of them can have a lateral margin devoid of filaments, such as Thraulus (T.) turbinatus or T. (T.) madagasikarensis (Grant, 2024: figs 141, 150). Thraulus (Th.) noe, Th. (Th.) ogea, and Th. (Th.) wemale are nevertheless considered as belonging to the subgenus Thraulus.

The rectangular labrum found in Th. (Th.) noe and Th. (Th.) eloisae is unique among all *Thraulus* nymphs where the labrum is either cordiform or trapezoid. Th. (Th.) noe and Th. (Th.) eloisae are also unique by the size of the maxillary palp almost the double of maxilla length, a character found in no other species of the tribe Thraulini. All other New Guinean species have a labrum emargination with flat denticles, so without crenation according to Grant (2024). Therefore, they differ from T. (T.) bellus, T. (T.) cursus, T. (T.) cuspidatus, T. (T.) connubialis, T. (T.) ishiwatai, T. (T.) nihonensis, T. (T.) plumeus, and Th. (T.) vellimalaiensis which have a large denticle in the middle of the emargination.

Thraulus (Th.) timorensis has a labrum which distal row of setae is multiple, whereas it is simple in other species. It also possesses a palisade of 4 small teeth on the claw besides the normal teeth, also present in Th. (Th.) sp. A. Posterolateral expansions of abdomen are present on segments VI–IX in Th. (Th.) nabire, on segments VII–IX in Th. (Th.) granti, Th. (Th.) longinquus and Th. (Th.) sp. A, whereas they are present on segments VIII–IX only in Th. (T.) amravati, T. (T.) bishopi, T. (T.) demoulini, T. (T.) fatuus, T. (T.) jacobusi, T. (T.) macilentus, T. (T.) mudumalaiensis, and T. (T.) umbrosus (Grant 2024; Isack et al. 2022; Soman 1991).

#### ﻿ Subgenus Masharikella

Taxon classificationAnimaliaEphemeropteraLeptophlebiidae

Peters, Gillies & Edmunds, 1964 stat. nov.

9558DEE9-0AD5-53EE-9C26-385D5308F8F0

##### Diagnosis.

Identical to the generic diagnosis, except that gill I is always composed of one dorsal lanceolate lamella and a large oval fimbriate ventral lamella.

##### Type species.

*Masharikellafasciatus* (Kimmins, 1956) by original designation.

##### Included species.

Thraulus (Masharikella) fasciatus (Kimmins, 1956), comb. nov. [Uganda, Zimbabwe], T. (M.) gopalani Grant & Sivaramakrishnan, 1985, comb. nov. [southern India], Th. (M.) iteris sp. nov. [Papua New Guinea], Th. (M.) johannisluci sp. nov. [Papua New Guinea], T. (M.) malabarensis Vasanth, Subramanian & Selvakumar, 2022, comb. nov. [southern India], T. (M.) opifer Grant, 2024, comb. nov. [northern Australia], Th. (M.) pascalae sp. nov. [Indonesia, Papua], Th. (M.) samueli sp. nov. [Papua New Guinea], T. (M.) thiagarajani Balasubramanian & Muthukatturaja, 2019, comb. nov. [southern India], and T. (M.) torrentis (Gillies, 1964), comb. nov. [Tanzania, Angola].

##### Distribution.

The subgenus exhibits a disjunct distribution in Africa, southern India, New Guinea, and northern Australia. Currently, it is surprisingly not recorded from Oriental southeast Asia, suggesting the species found in New Guinea originate from tropical Australia. This may indicate a Gondwanan origin for the subgenus. Its presence in Madagascar is expected but yet not proved.

#### ﻿ Thraulus (Masharikella) iteris sp. nov.

Taxon classificationAnimaliaEphemeropteraLeptophlebiidae

E7EE67EF-0184-55C2-903B-39CE0943E58E

https://zoobank.org/DF5B8A4A-914C-4FD0-8F7E-FEC6252F8276

**Figure 24.**
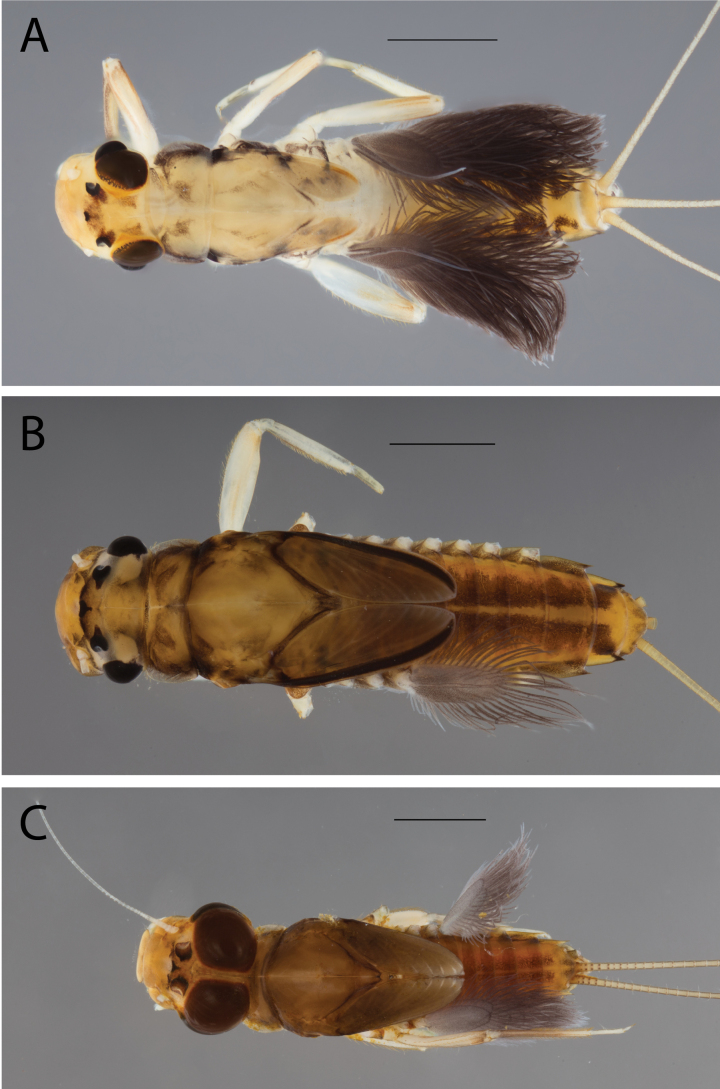
Habitus of Thraulus (Masharikella) samueli sp. nov. (**A**), Thraulus (Masharikella) iteris sp. nov. (**B**) and Thraulus (Masharikella) pascalae sp. nov. (**C**). Scale bars: 1 mm.

**Figure 25.**
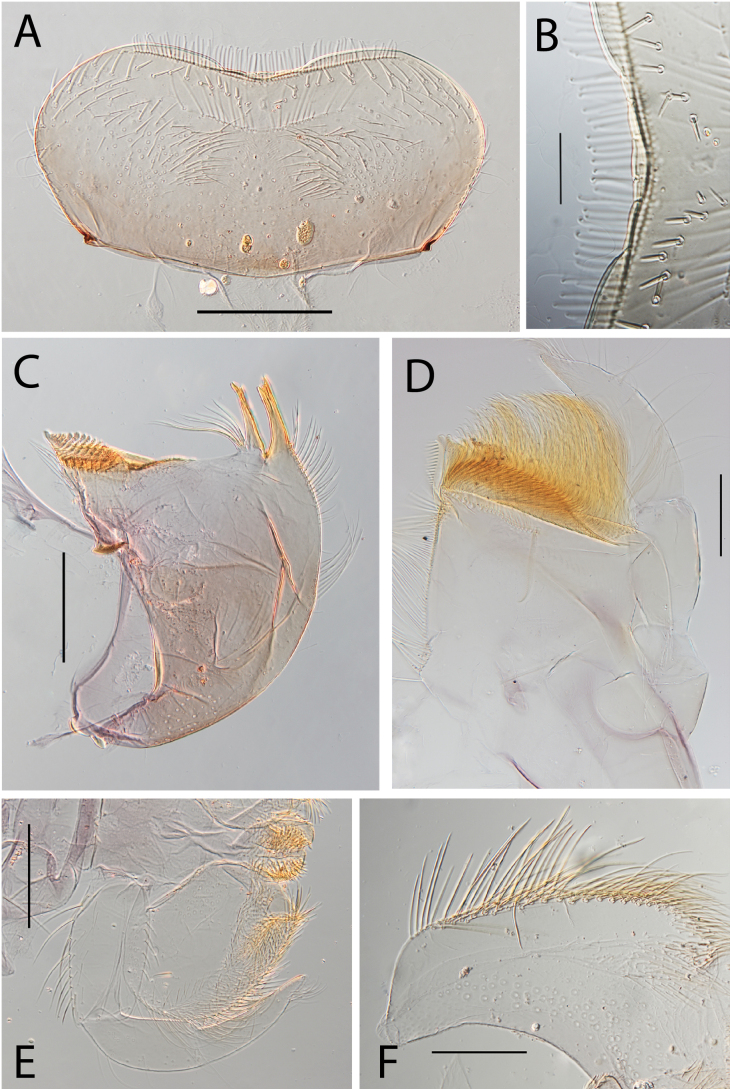
Thraulus (Masharikella) iteris sp. nov. nymphal mouthparts. **A.** Labrum, dorsal view; **B.** Emargination of the labrum; **C.** Right mandible; **D.** Maxilla; **E.** Labium; **F.** Superlingua of hypopharynx. Scale bars: 200 µm (**A, C, E**); 50 µm (**B**); 100 µm (**D, F**).

**Figure 26.**
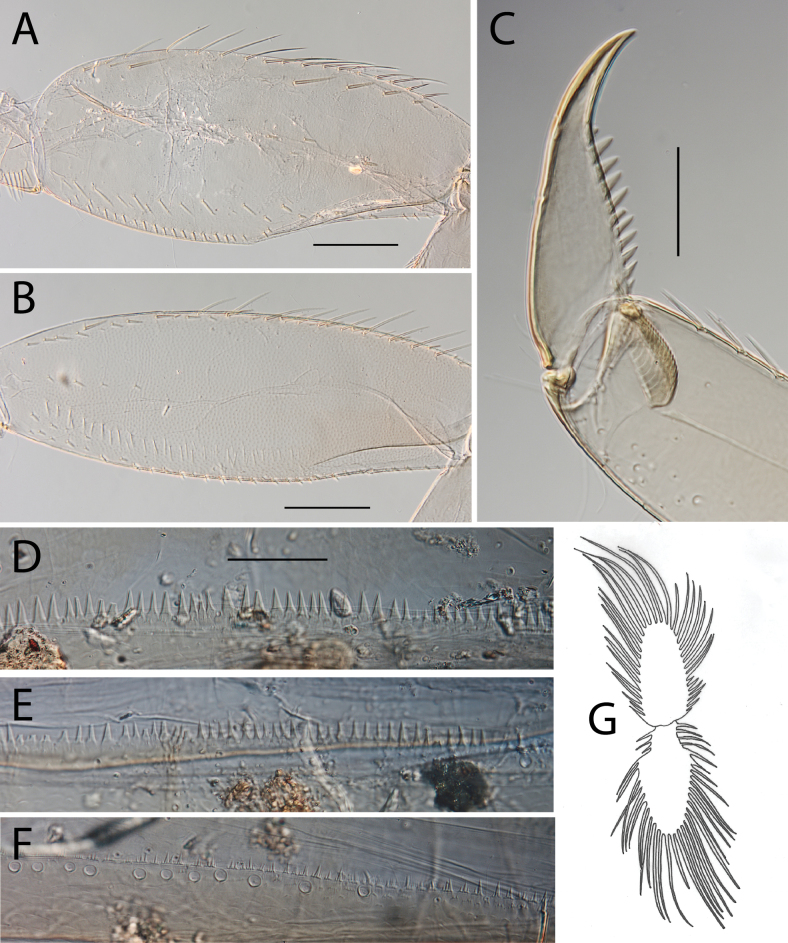
Thraulus (Masharikella) iteris sp. nov. thorax and abdomen. **A.** Fore femur; **B.** Hind femur; **C.** Claw; **D.** Posterior margin of tergite IX; **E.** Posterior margin of tergite VIII; **F.** Posterior margin of tergite VII; **G.** Gill IV. Scale bars: 200 µm (**A, B**); 50 µm (**C–F**).

Figs 24B , 25 , 26 , 34B

##### Material examined.

***Holotype*.** • Papua New Guinea, nymph on slide, Central Province, Kokoda Trek, 980 m, I.2008, 09°15.933'S, 147°36.590'E, Posman col [PNG169], GBIFCH01223115 (ZSM). ***Paratypes*.** • Papua New Guinea, 1 nymph entirely mounted on slide, GBIFCH01223116, 1 nymph in ethanol, GBIFCH01523480, same data as holotype (MZL). • Papua New Guinea, 1 nymph entirely mounted on slide, GBIFCH01223117, some gills in ethanol, GBIFCH01523479, Central Province, Kokoda Trek, 320 m, I.2008, 09°19.236'S, 147°31.791'E, Posman col [PNG168] (MZL).

##### Nymph.

Body length, female: 5.8–6.2 mm.

##### Diagnosis.

Labrum width smaller than clypeus width; proximal row of setae on dorsal face of labrum with < 30 setae; ventral side of labrum with stout setae in a row; maxillary palp segment III 1.7–2.2 × as long as base width; posterior margin of abdominal segment VIII with well-developed denticles.

##### Colouration.

***Head*.** Yellowish, pale brown between ocelli and below compound eyes. Antenna yellowish. Mouth parts pale brown, mandibles medium brown. ***Thorax*.** Pronotum medium brown, dark brown laterally and posteriorly, as well as two elongated dots close to the median line. Mesonotum medium brown, dark brown laterally and on the anterolateral corners; costal and subcostal areas of forewing pads strongly tinted with black (Fig. 24B). Metanotum medium brown. Femur whitish, apex washed with pale orange. Tibia whitish, inner margin and apex washed with grey. Tarsi whitish. ***Abdomen*.** Terga brownish orange, with two longitudinal dark brown large stripes on segments I–IX; segment X medium brown. Sterna brownish orange, lateral margins washed with grey, posterolateral projections XIII–IX black. Gills purplish grey. Terminal filaments uniformly pale brown.

##### Description.

***Head*. *Labrum*** cordiform (Fig. 25A). Ratio labrum width/clypeus width 0.86–0.97. Ratio labrum width/insertion width 1.26–1.30. Medial emargination shallow with flat denticles. Proximal row of setae present, simple. Number of setae on proximal row 19–23. Distal row of setae present, simple. Median part of labrum in ventral view with stout setae in row (Fig. 25B). ***Mandibles*.** Outer margin with middle and distal tuft of setae. Right mandible with 9–11 setae below the mola (Fig. 25C). ***Maxillae*.** Posterior margin of cardo with many hair-like setae and one stout and long seta in submarginal position (Fig. 34B). Proximo-lateral margin of stipes with a single stout and medium size seta. Apical-ventral row with 23–25 pectinate setae. Maxillary palp segment II 0.82–0.86 as long as segment I. Maxillary palp segment III 0.71–0.77 as long as segment I (Fig. 25D). Stout setae on outer margin of segment I absent. Segment II inner margin with two to five stout setae. Segment II outer margin with three or four stout setae. Segment III inner margin with 4–6 stout setae, dorsal surface with few setae at apex. Segment III 1.82–2.44 × as long as base width. ***Labium*.** Labial palp segment II 0.67–0.80 as long as segment I. Labial palp segment III 0.79–0.90 as long as segment I (Fig. 25E). Segment I inner margin with 5–9 stout setae, outer margin with 10–12 stout setae. Segment II outer margin with one or two stout setae. Segment III dorsal face with 5–7 stout setae. Segment III 3.50–3.80 × as long as base width. ***Hypopharynx*.** Apex of superlingua emarginated (Fig. 25F).

***Thorax*.** Dorsal margin of femur with a row of long, stout, pointed setae; submarginal row with shorter setae; ventral margin with short, blunt, stout setae and a submarginal row of feathered setae; central area of upper surface without setae (Fig. 26A–B). Outer margin of fore tibia without setae; mid tibia with a row of stout, pointed setae on ventral margin; outer margin with only some hair-like setae; hind tibia covered with numerous simple and feathered setae, outer margin with a dense row of stout, pointed setae. Tarsal claw hooked, with 10–11 subequal denticles (Fig. 26C).

***Abdomen*.** Gill I dorsal lamella narrow and lanceolate, ventral lamella shaped as other gills. Gills II–VII with dorsal and ventral lamellae of the same size. Gill II to VI with ~ 40 filaments on each lamella (Fig. 26G); gill VII with ~ 25 filaments on each lamella. Filamentous projections along entire margin. Posterior margin of terga VIII and IX with denticles (Fig. 26D–E). Posterior margin of terga VII with minute denticles (Fig. 26F). Posterior margin of terga V–VI without denticles. Posterolateral projections present on segment VII–IX.

##### Derivatio nominis.

This species is named after the Latin word *iter*, meaning path, to remember the new species has been collected along the trail from Port Moresby to Kokoda.

#### ﻿ Thraulus (Masharikella) johannisluci sp. nov.

Taxon classificationAnimaliaEphemeropteraLeptophlebiidae

A2A45575-0579-55AA-94A1-77CB092F8CAB

https://zoobank.org/D08FCD43-CAF3-443D-BBAE-C922C7A272C8

Figs 27 , 28 , 34F

##### Material examined.

***Holotype*.** • Papua New Guinea, nymph on slide, Central Province, Kokoda Trek, 980 m, I.2008, 09°15.933'S, 147°36.590'E, Posman col [PNG169], GBIFCH01223118 (ZSM). ***Paratype*.** • Papua New Guinea, 1 nymph in ethanol, GBIFCH01523481, same data as holotype (MZL).

##### Nymph.

Body length, female: 4.5 mm.

##### Diagnosis.

Labrum width equal to clypeus width; proximal row of setae on dorsal face of labrum with > 35 setae; maxillary palp segment III < 1.5 × as long as base width; tarsal claw with four teeth, distal one much larger than the others; posterior margin of abdominal segment VII–VIII without denticles; posterolateral projections of the abdomen present on segments VII–IX.

##### Colouration.

***Head*.** Pale brown washed with grey, triangular yellowish area between front ocellus and clypeus. Mouth parts greyish brown. ***Thorax*.** Pronotum greyish brown, dark brown on lateral margins and along the sagittal line. Mesonotum pale brown washed with grey. Metanotum pale brown washed with grey. Femur upper surface greyish brown except apex yellowish, lower surface pale grey. Tibia yellowish brown, apex greyish. Tarsi yellowish to orange- brown. ***Abdomen*.** Terga more or less uniformly greyish brown, segments II–VII with a dark dot above the gill insertion, segments I–VIII with a paler sagittal line. Sterna yellowish grey in median part, segments I–VII greyish laterally, segment IX darker. Gills purplish grey. Terminal filaments uniformly pale brown.

##### Description.

**Figure 27.**
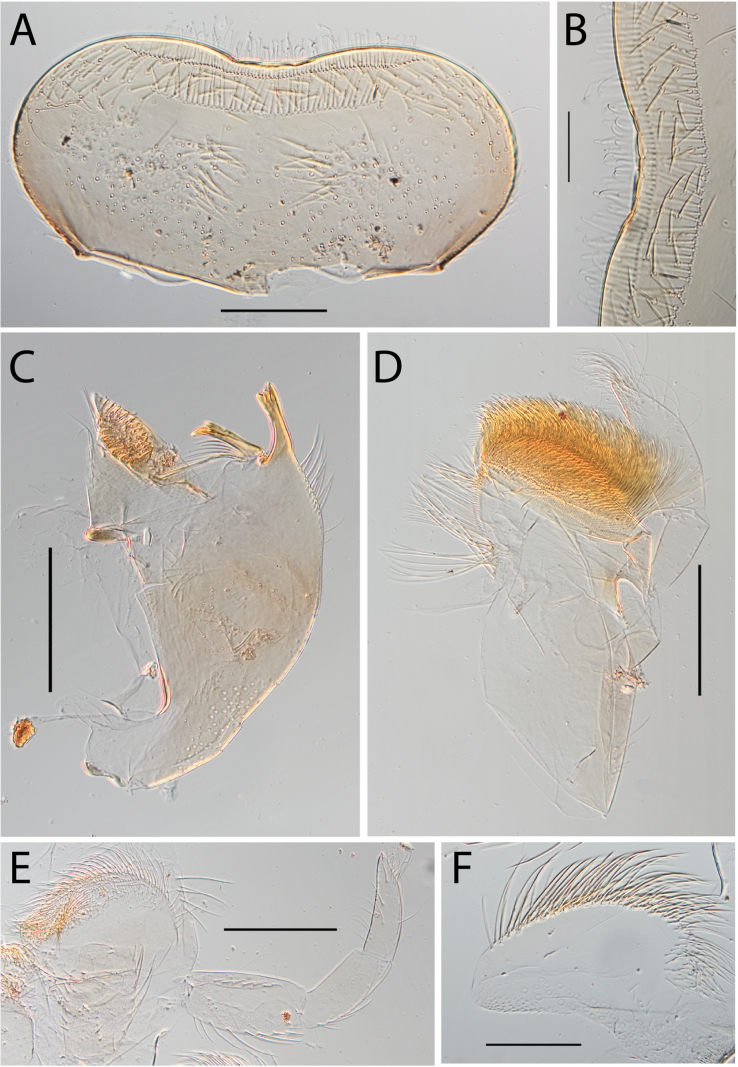
Thraulus (Masharikella) johannisluci sp. nov. nymphal mouthparts. **A.** Labrum, dorsal view; **B.** Emargination of the labrum; **C.** Right mandible; **D.** Maxilla; **E.** Labium; **F.** Superlingua of hypopharynx. Scale bars: 100 µm (**A, F**); 50 µm (**B**); 200 µm (**C–E**).

***Head*. *Labrum*** cordiform (Fig. 27A). Ratio labrum width/clypeus width 1.00. Ratio labrum width/insertion width 1.40. Medial emargination shallow with flat denticles. Proximal row of setae present simple. Number of setae on proximal row 42. Distal row of setae present, simple. Median part of labrum in ventral view with only scattered stout setae (Fig. 27B). ***Mandibles*.** Outer margin with row of setae on distal 1/2. Ending of the row near incisor. Right mandible with nine setae below the mola (Fig. 27C). ***Maxillae*.** Posterior margin of cardo broken. Proximo-lateral margin of stipes with two long and two short stout setae (Fig. 34F). Apical-ventral row with 24 pectinate setae. Maxillary palp segment II 0.81 as long as segment I. Maxillary palp segment III 0.80 as long as segment I (Fig. 27D). Stout setae on outer margin of segment I absent. Segment II inner margin with two stout setae. Segment II outer margin without stout setae. Segment III inner margin with three stout setae, dorsal surface with few setae at apex. Segment III 1.45 × as long as base width. ***Labium*.** Labial palp segment II 0.69 as long as segment I. Labial palp segment III 0.88 as long as segment I (Fig. 27E). Segment I inner margin with seven stout setae, outer margin with eight stout setae. Segment II outer margin with three stout setae. Segment III dorsal face with three stout setae. Segment III 3.0 × as long as base width. ***Hypopharynx*.** Apex of superlingua rounded (Fig. 27F).

**Figure 28.**
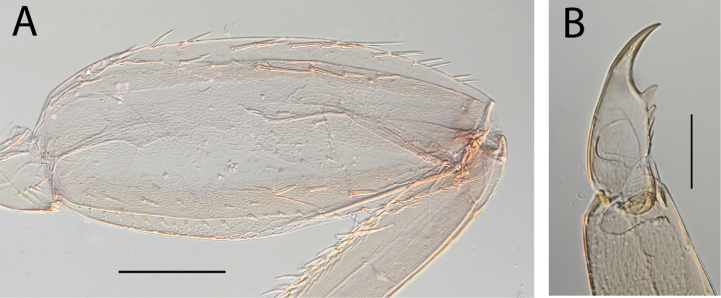
Thraulus (Masharikella) johannisluci sp. nov. thorax. **A.** Fore femur; **B.** Claw Scale bars: 100 µm (**A**); 50 µm (**B**).

***Thorax*.** Dorsal margin of femur with a row of long, stout, pointed setae; submarginal row with setae of irregular length, as long or half the length of dorsal margin; ventral margin with short, blunt, stout setae and a submarginal irregular row of simple setae (Fig. 28A); central area of upper surface with few stout, pointed setae. Fore tibia covered with numerous simple and feathered setae on ventral margin; outer margin of fore tibia without setae; mid- and hind tibia with a row of stout, pointed setae on ventral margin, outer margin without setae on mid tibia, with a sparse row of stout and pointed setae on hind tibia. Tarsal claw hooked with 4 denticles progressively larger, except distal much larger (Fig. 28B).

***Abdomen*.** Gill I dorsal lamella narrow and lanceolate, ventral lamella shaped as other gills. Gills II–VII with dorsal and ventral lamellae of the same size. Gill I with ~ 20 filaments on the ventral lamella. Gill II–VI with 25–30 filaments on each lamella. Posterior margin of terga IX with denticles. Posterior margin of terga V–VIII without denticles. Posterolateral projections present on segment VII–IX.

##### Derivatio nominis.

This species is named after our colleague and friend Dr Jean-Luc Gattolliat (Naturéum, Lausanne), in recognition of his outstanding contributions to the Baetidae in general and to those of New Guinea in peculiar.

#### ﻿ Thraulus (Masharikella) pascalae sp. nov.

Taxon classificationAnimaliaEphemeropteraLeptophlebiidae

061A328C-B70C-5699-B1B3-DE24EB9F867F

https://zoobank.org/4EC98EE3-F6A1-4BB6-9385-E2BA9A0D4837

**Figure 29.**
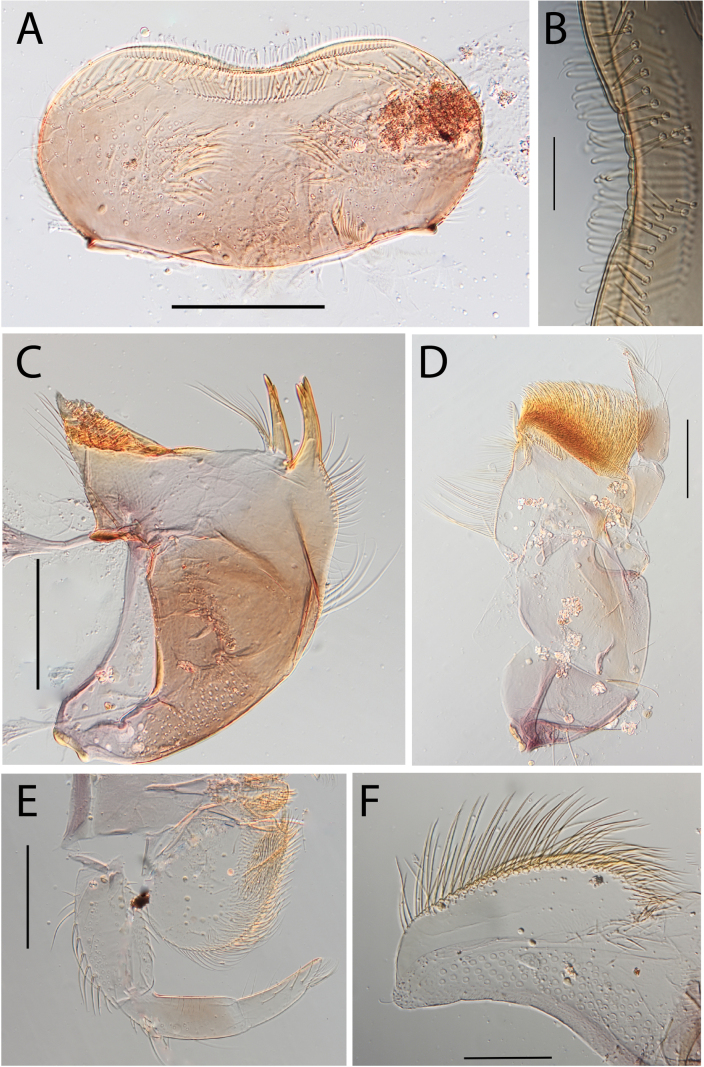
Thraulus (Masharikella) pascalae sp. nov. nymphal mouthparts. **A.** Labrum, dorsal view; **B.** Emargination of the labrum; **C.** Right mandible; **D.** Maxilla; **E.** Labium; **F.** Superlingua of hypopharynx. Scale bars: 200 µm (**A, C–E**); 50 µm (**B**); 100 µm (**F**).

**Figure 30.**
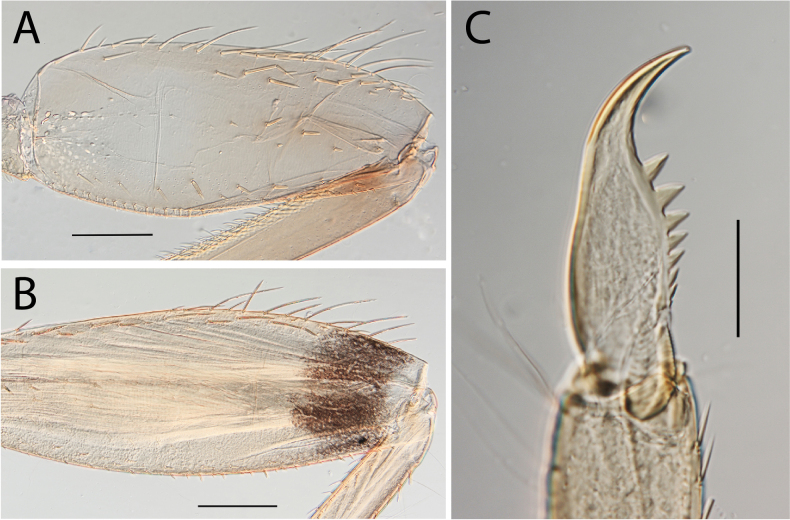
Thraulus (Masharikella) pascalae sp. nov. thorax. **A.** Fore femur; **B.** Hind femur; **C.** Claw. Scale bars: 200 µm (**A, B**); 50 µm (**C**).

Figs 24C , 29 , 30 , 34G

##### Material examined.

***Holotype*** • Indonesia, Papua, nymph on slide, Road Nabire–Enarotali KM 95, 160 m, 22.X.2011, 03°34.193'S, 135°49.246'E, M. Balke col [PAP13], GBIFCH01223119 (MZB). ***Paratypes*.** • Indonesia, Papua, one nymph on slide, GBIFCH01223150, 2 nymphs in ethanol, GBIFCH01523482, same data as holotype (MZL).

##### Nymph.

Body length, male: 5 mm.

##### Diagnosis.

Apical ventral row of maxilla composed of 25–35 pectinate setae; outer margin of maxillary palp II with five stout setae; outer margin of labial palp segment II with five stout setae; tarsal claw with six or seven teeth progressively larger; posterior margin of abdominal segment IX with well-developed denticles; posterolateral projections of the abdomen present on segments VI–IX.

##### Colouration.

***Head*.** Pale brown, medium brown between ocelli. Antenna whitish. Compound eye lower portion black, upper portion reddish brown. Mouth parts pale brown. ***Thorax*.** Pronotum medium brown. Mesonotum medium brown, parapsidal sutures dark brown. Metanotum medium brown. Femur yellowish, apex with an orange to pale brown dot. Tibia: fore- and middle tibiae yellowish, hind tibia orange to brown. Tarsi whitish. ***Abdomen*.** Terga medium brown, with a pale and narrow sagittal line, segment IX lighter with two dark maculae, segment X greyish brown (Fig. 24C). Sterna pale brown, posterior margin of segments II to VIII medium brown. Gills with lamellae pale grey, filaments greyish purple. Terminal filaments yellowish, articulations of ~ 15 first segments tinted in brownish purple.

##### Description.

***Head*. *Labrum*** cordiform (Fig. 29A). Ratio labrum width/clypeus width 0.93. Ratio labrum width/insertion width 1.29. Medial emargination shallow with flat denticles. Proximal row of setae present, simple. Number of setae on proximal row ~ 31. Distal row of setae present, simple. Median part of labrum in ventral view with stout setae in row (Fig. 29B). ***Mandibles*.** Outer margin with middle and distal tufts of setae. Ending of the row near incisor. Right mandible with ten setae below the mola (Fig. 29C). ***Maxillae*.** Posterior margin of cardo with numerous hair-like setae and few stout setae, with a long and stout seta in submarginal position. Proximo-lateral margin of stipes with a single stout and long seta (Fig. 34G). Apical-ventral row with 20 pectinate setae. Maxillary palp segment II 0.85 as long as segment I. Maxillary palp segment III 0.69 as long as segment I (Fig. 29D). Stout setae on outer margin of segment I absent. Segment II inner margin with five stout setae. Segment II outer margin with five stout setae. Segment III inner margin with four stout setae, dorsal surface with few setae at apex. Segment III 1.73 × as long as base width. ***Labium*.** Labial palp segment II 0.84 as long as segment I. Labial palp segment III 0.74 as long as segment I (Fig. 29E). Segment I inner margin with nine stout setae, outer margin with ten stout setae. Segment II outer margin with five stout setae. Segment III dorsal face with four stout setae. Segment III 3.11 × as long as base width. ***Hypopharynx*.** Apex of superlingua emarginated (Fig. 29F).

***Thorax*.** Dorsal margin of femur with a row of long, stout, pointed setae; submarginal row with setae of irregular length, row as long or half the length of dorsal margin; ventral margin with short, blunt, stout setae and a submarginal irregular row of simple setae (Fig. 30A–B); central area of upper surface with few long, pointed setae. Fore tibia with three rows of numerous simple and feathered setae on ventral margin; outer margin of fore tibia with few hair-like setae; middle tibia with two rows of long, pointed setae on ventral margin, outer margin with numerous hair-like setae; hind tibia with a row of long, thin setae on ventral margin, with a sublateral row of simple and feathered setae, ventral surface covered with simple and feathered setae, outer margin with long, pointed stout setae. Tarsal claw hooked with six or seven denticles progressively larger apically (Fig. 30C).

***Abdomen*.** Gill I dorsal lamella narrow and lanceolate, ventral lamella shaped as other gills. Gills II–VII with dorsal and ventral lamellae of the same size. Gill II–VI with ~ 25 filaments on each lamella, gill VII with ~ 15 filaments. Posterior margin of terga IX with denticles. Posterior margin of terga VIII with minute denticles. Posterior margin of terga V–VII without denticles. Posterolateral projections present on segments VI–IX.

##### Derivatio nominis.

This species is dedicated to Dr Pascale Derleth Sartori, wife of the first author.

#### ﻿ Thraulus (Masharikella) samueli sp. nov.

Taxon classificationAnimaliaEphemeropteraLeptophlebiidae

B1B9E9DD-1109-53B1-BCCF-3C4E9BA8A96F

https://zoobank.org/2C6CACC2-018C-4A55-B60A-24B0DF238096

**Figure 31.**
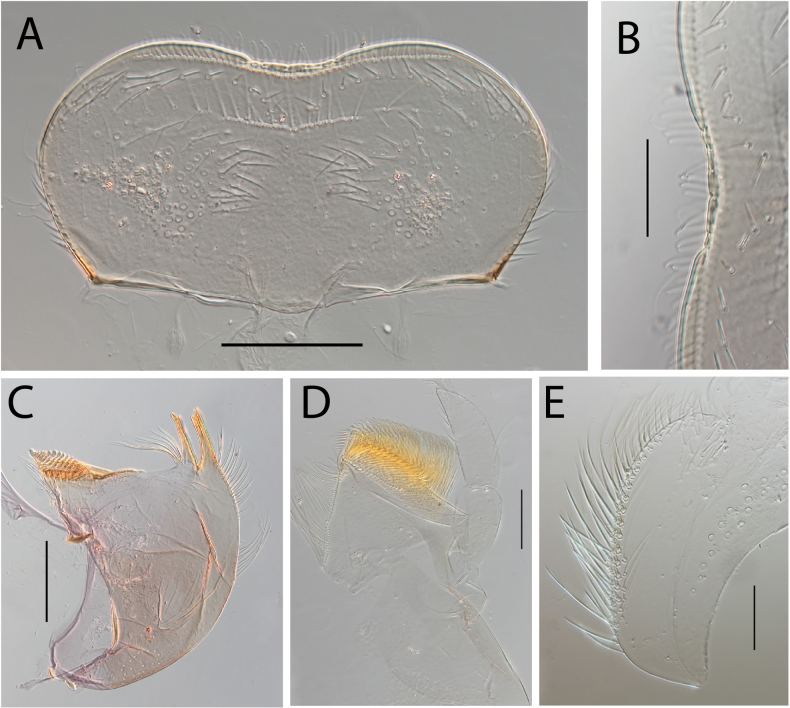
Thraulus (Masharikella) samueli sp. nov. nymphal mouthparts. **A.** Labrum, dorsal view; **B.** Emargination of the labrum; **C.** Right mandible; **D.** Maxilla; **E.** Superlingua of hypopharynx. Scale bars: 200 µm (**A, C, D**); 50 µm (**B**); 100 µm (**E**).

**Figure 32.**
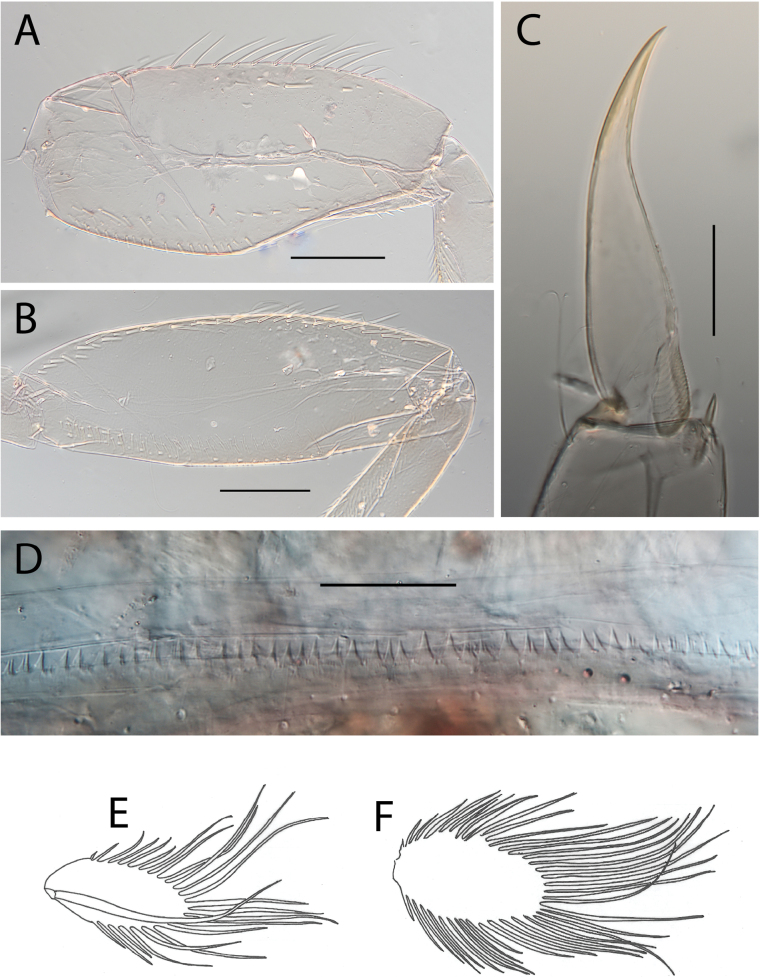
Thraulus (Masharikella) samueli sp. nov. thorax and abdomen. **A.** Fore femur; **B.** Hind femur; **C.** Claw; **D.** Posterior margin of tergite IX; **E.** Gill I; **F.** Gill IV (dorsal lamella only). Scale bars: 200 µm (**A, B**); 50 µm (**C, D**).

Figs 24A , 31 , 32 , 64A, B

##### Material examined.

***Holotype*.** • Papua New Guinea, male nymph on slide, National Capital District, Varirata National Park, 600 m a.s.l., 09°26.13'S / 147°22.09'E, 16.XII.2007, Balke and Sagata col [PNG159], GBIFCH01223120 (ZSM). ***Paratypes*.** • Papua New Guinea, 4 nymphs in ethanol, GBIFCH01523483, 2 nymphs entirely mounted on slide, GBIFCH01223121, GBIFCH01223122, same data as holotype (MZL).

##### Nymph.

Body length, male: 4.5–4.8 mm; female: 4.9–5.2 mm.

##### Diagnosis.

Tarsal claw straight, without teeth; apical ventral row of maxilla composed of 16–20 pectinate setae; posterior margin of abdominal segment IX with minute denticles; posterolateral projections of the abdomen present on segments VIII–IX only.

##### Colouration.

***Head*.** Yellowish brown, darker between ocelli. Triangular brown mark present on inner margin of compound eye. Antenna yellowish. Compound eye lower portion black, upper portion dark brown, not rounded but oblique and elongated. Mouth parts yellowish. ***Thorax*.** Pronotum yellowish washed with greyish brown laterally, irregular brown mark close to median line. Mesonotum yellowish washed with greyish laterally; distal half of costal field of fore wing pad orangish. Metanotum yellowish washed with greyish laterally. Femur yellowish white, dorsal surface apically washed with orange (Fig. 24A). Tibia whitish, washed with brown on dorsal surface. Tarsi yellowish white. ***Abdomen*.** Terga orangish with dark brown rectangular marks laterally on segments I to VI, subtriangular on segment VII and IX, segment VIII entirely dark brown. Sterna uniformly whitish becoming yellowish on the last segments. Gills purplish black. Terminal filaments yellowish white washed with orange proximally.

##### Description.

***Head*. *Labrum*** cordiform (Fig. 31A). Ratio labrum width/clypeus width 0.89–0.98. Ratio labrum width/insertion width 1.22–1.24. Medial emargination shallow with flat denticles. Proximal row of setae present, simple. Number of setae on proximal row ~ 16–37. Distal row of setae present, simple. Median part of labrum in ventral view with stout setae in row (Fig. 31B). ***Mandibles*.** Outer margin with middle and distal tuft of setae. Right mandible with 6–11 setae below mola (Fig. 31C). ***Maxillae*.** Posterior margin of cardo with few hair-like setae and a long and stout seta in submarginal position. Proximo-lateral margin of stipes with a single stout and medium size seta. Apical-ventral row with 16–20 pectinate setae. Maxillary palp segment II 0.79–0.92 as long as segment I. Maxillary palp segment III 0.62–0.68 as long as segment I (Fig. 31D). Stout setae on outer margin of segment I absent. Segment II inner margin with two or three stout setae. Segment II outer margin without stout setae. Segment III inner margin with 2–4 stout setae, dorsal surface with few setae at apex. Segment III 1.5–2.0 × as long as base width. ***Labium*.** Labial palp segment II 0.72–0.76 as long as segment I. Labial palp segment III 0.79–0.92 as long as segment I. Segment I inner margin with 7–12 stout setae, outer margin with 8–12 stout setae. Segment II outer margin without or with one or two stout setae. Segment III dorsal face with five or six stout setae. Segment III 3.0–3.67 × as long as base width. ***Hypopharynx*.** Apex of superlingua emarginated (Fig. 31E).

***Thorax*.** Dorsal margin of femur with a row of long, stout, pointed setae; incomplete submarginal row, sometimes with scattered long, pointed setae; ventral margin with short, blunt, stout setae; central area of upper surface without setae (Fig. 32A–B). Inner margin of fore- and mid- tibia covered with several rows of simple and feathered setae; inner margin of hind tibia with several rows of simple and feathered setae; outer margin of fore- and mid- tibia naked, with a row of stout, pointed setae on hind tibia, especially developed in the distal half. Tarsal claw straight. Denticles absent (Fig. 32C).

***Abdomen*.** Gill I dorsal lamella narrow and lanceolate, ventral lamella shaped as other gills (Fig. 32E). Gills II–VII with dorsal and ventral lamellae of the same size. Ventral lamella of gill I with ~ 25 long filaments; dorsal and ventral lamellae of gills II–VI with ~ 40 very long filaments each (Fig. 32F); gill VII with ~ 20 filaments on each lamella. Posterior margin of terga IX with small denticles (Fig. 32D). Posterior margin of terga V–VIII without denticles. Posterolateral projections on segments VIII–IX.

***Eggs*.** General shape elongated. Size ca 185 μm × 80 μm. Chorionic surface with irregular longitudinal ridges connected transversally, forming rectangular smooth areas in-between (Fig. 64A). On each pole arise basally six equally distant pairs of long, apically hooked attachment structure (Fig. 64B). Micropyle not visible.

##### Derivatio nominis.

This species is dedicated to Samuel Sartori, grandson of the first author.

#### ﻿ Thraulus (Masharikella)

Taxon classificationAnimaliaEphemeropteraLeptophlebiidae

sp. B

9FE71F2E-93C9-5D74-955B-84C4FDCECCC7

Fig. 33

##### Material examined.

• Indonesia, Papua, one nymph on slide, Road Nabire–Enarotali KM 52, 555 m, 23.X.2011, 03°30.107'S, 135°42.971'E, M. Balke col [PAP17], GBIFCH01223123 (MZL).

##### Diagnosis.

**Nymph.** Labrum width smaller than clypeus width; proximal row of setae on dorsal face of labrum with < 30 setae; ventral side of labrum with scattered stout setae; maxillary palp segment III ~ 1.7 × as long as base width; posterior margin of abdominal segment VIII with minute denticles; posterolateral projections on the abdomen present on segments VII–IX.

##### Description.

**Figure 33.**
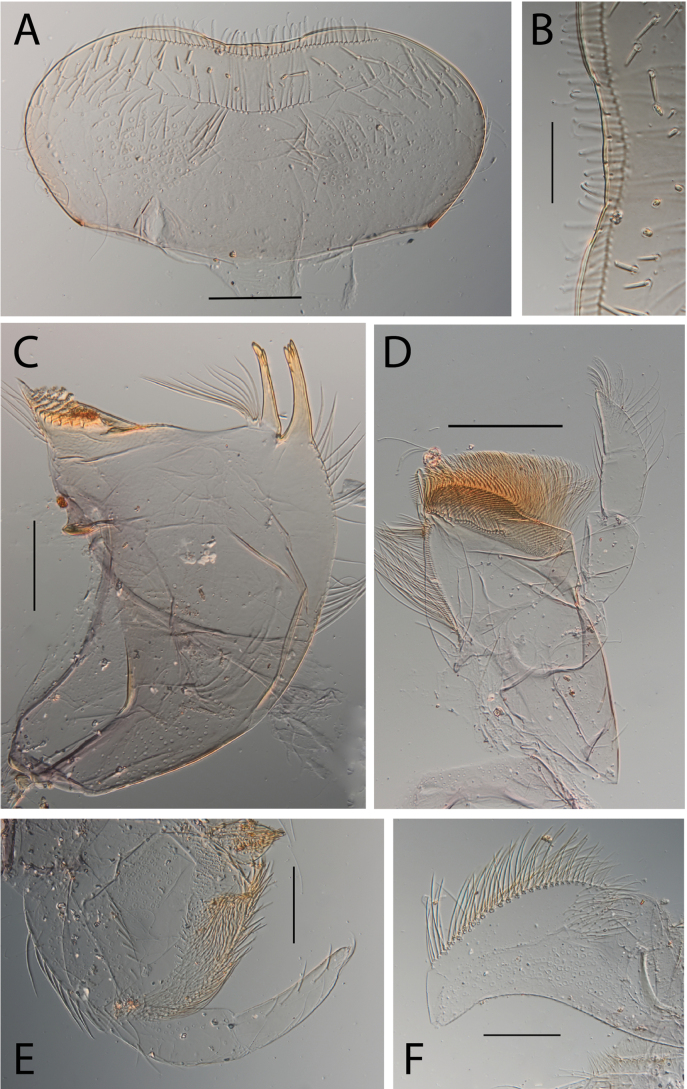
Thraulus (Masharikella) sp. B. Nymphal mouthparts. **A.** Labrum, dorsal view; **B.** Emargination of the labrum; **C.** Right mandible; **D.** Maxilla; **E.** Labium; **F.** Superlingua of hypopharynx. Scale bars: 100 µm (**A, C, E, F**); 50 µm (**B**); 200 µm (**D**).

***Head*. *Labrum*** cordiform (Fig. 33A). Ratio labrum width/clypeus width 0.95. Ratio labrum width/insertion width 1.33. Medial emargination shallow with flat denticles. Proximal row of setae present simple. Number of setae on proximal row 23. Distal row of setae present, simple. Median part of labrum in ventral view with only scattered stout setae (Fig. 33B). ***Mandibles*.** Outer margin with middle and distal tuft of setae. Right mandible with 11 setae below the mola (Fig. 33C). ***Maxillae*.** Posterior margin of cardo with few hair-like setae. Proximo-lateral margin of stipes with two long and two short stout setae. Apical-ventral row with 24 pectinate setae. Maxillary palp segment II 0.78 as long as segment I. Maxillary palp segment III 0.74 as long as segment I (Fig. 33D). Stout setae on outer margin of segment I absent. Segment II inner margin with three stout setae. Segment II outer margin with four stout setae. Segment III inner margin with three stout setae, dorsal surface with few setae at apex. Segment III 1.70 × as long as base width. ***Labium*.** Labial palp segment II 0.71 as long as segment I. Labial palp segment III 0.90 as long as segment I (Fig. 33E). Segment I inner margin with seven stout setae, outer margin with ten stout setae. Segment II outer margin with one stout seta. Segment III dorsal face with five stout setae. Segment III 3.00 × as long as base width. ***Hypopharynx*.** Apex of superlingua emarginated (Fig. 33F).

***Abdomen*.** Gill I dorsal lamella narrow and lanceolate, ventral lamella shaped as other gills. Gill I with ~ 18 filaments. Posterior margin of terga IX with denticles. Posterior margin of terga VII–VIII with minute denticles. Posterior margin of terga V–VI without denticles. Posterolateral projections present on segments VII–IX.

### ﻿Differential diagnoses among species of the subgenus Masharikella

Until now, only six species of the subgenus Masharikella were known; New Guinean material almost allows to double this number. All species from New Guinea can be easily separated using the key provided at the end of this work. All examined species have a labrum emargination with flat denticles; we interpret this character as similar to “anterior emargination of labrum without crenations” in the revision of Grant (2024, p. 14). Our species therefore differ from T. (M.) malabarensis which possesses a single large and rounded crenation (denticle in our wording), and from T. (M.) torrentis which possesses several small and rounded crenations. In New Guinean species, outer margin of mandibles is composed of a row of setae ending near incisors (in Th. (M.) johannisluci) or is adorned with two tufts of setae in middle and near the incisors (in Th. (M.) pascalae). Therefore, those possessing this character, i.e., Th. (M.) iteris, Th. (M.) samueli and Th. (M.) sp. B differ from those already described. Th. (M.) johannisluci and Th. (M.) pascalae possess a labrum with shallow anterior emargination which differ from the one of T. (M.) gopalani which is narrow and rectangular; the row of setae on outer margin of mandibles ending near incisors is different from the one present in T. (M.) thiagarajani. Th. (M.) pascalae has abdominal posterolateral projections on segments VI–IX, which differs from Th. (M.) fasciatus (VIII–IX) and T. (M.) opifer (VII–IX). Therefore, Th. (M.) johannisluci is most similar to T. (M.) opifer described from northern Australia. It can be separated by the ventral side of the labrum which possesses scattered stout setae, whereas these setae are in a row in T. (M.) opifer, as well as by the claw which is adorned with four teeth in Th. (M.) johannisluci and 8–11 teeth in T. (M.) opifer. Finally, it is worth to mention that Th. (M.) samueli is remarkable in having claws straight and edentate, a character which is only shared with Th. (M.) sp. AW3 from northern Queensland, Australia (Dean 1999).

#### ﻿Genus Nonnullidens

Taxon classificationAnimaliaEphemeropteraLeptophlebiidae

Grant & Peters, 1993

E35DB7C1-E537-5F9E-86ED-4551AF3E2EEC

##### Diagnosis.

Ventral lamella of gills highly reduced or absent, ventral lamella of first gill with < 8 processes, even absent in some species; claw with generally 3–6 teeth, rarely up to 8; posterior margin of abdominal segment VIII generally without denticles; posterolateral expansions on abdominal segments VIII–IX, rarely VII–IX.

##### Composition.

*Nonnullidensalvarezi* sp. nov. [Papua New Guinea], *N.anga* sp. nov. [Papua New Guinea], *N.billhilli* Grant & Peters, 1993 [Papua New Guinea, New Britain], *N.boonsoongi* sp. nov. [Papua New Guinea], *N.cozzarolae* sp. nov. [Papua New Guinea], *N.depapricus* Kluge, 2013 [Indonesia, Papua], *N.fuyugensis* sp. nov. [Papua New Guinea], *N.hsui* (Peters & Tsui, 1972) [Papua New Guinea], *N.kaltenbachi* sp. nov. [Papua New Guinea], *N.marcelae* sp. nov. [Indonesia, Papua], *N.mariae* (Peters & Tsui, 1972) [Papua New Guinea], *N.moiorum* sp. nov. [Indonesia, Papua], *N.niger* Kluge, 2013 [Indonesia, Papua], *N.reductus* Kluge, 2013 [Indonesia, Papua], *N.silvaepumilorum* sp. nov. [Papua New Guinea], *N.variegatus* Kluge, 2013 [Indonesia, Papua]

##### Distribution.

Species of the genus *Nonnullidens* are restricted to New Guinea; *N.billhilli* is also reported from New Britain (Grant and Peters 1993).

#### ﻿ Nonnullidens alvarezi sp. nov.

Taxon classificationAnimaliaEphemeropteraLeptophlebiidae

7B61E377-8201-5D8E-B4CB-50B7A852B291

https://zoobank.org/F499747D-58E5-4F86-BC77-9A19F3583E57

Figs 36B , 37 , 38

##### Material examined.

***Holotype*.** • Papua New Guinea, one nymph on slide, Eastern Highlands Province, Akameku – Brahmin, Bismarck Range, 2200 m, 23.XI.2006, 05°56.801'S, 145°22.238'E, M. Balke & Kinibel col [PNG106], GBIFCH01223124 (ZSM). ***Paratypes*.** • Papua New Guinea, 2 nymphs in ethanol (UFVB), 2 nymphs on slide, GBIFCH01223125, GBIFCH01223126, 7 nymphs in ethanol, GBIFCH01523456 same data as holotype (MZL). • Papua New Guinea, one nymph on slide, GBIFCH01223127, gills and legs in ethanol, GBIFCH01523457, Gulf Province, Marawaka, Mala, 1400 m, 11.XI.2006, 07°05.664'S, 145°44.467'E, M. Balke & Kinibel col [PNG90] (MZL).

##### Nymph.

Body length, male: 5.5 mm; female: 6.5 mm.

##### Diagnosis.

Femora uniformly dark brown, without transverse bands; tarsal claw with 6–8 teeth; gill I–II uni-lamellate, without ventral lamella; gills III–VI with 5–8 filaments on dorsal lamella and four or five filaments on ventral lamella, gill VII with four or five filaments on dorsal lamella and one or two filaments on ventral lamella.

##### Colouration.

***Head*.** Vertex dark brown, frons medium brown with an oval pale brown macula near the frons ocellus. Antenna broken. Compound eye upper portion reddish brown. Mouth parts pale brown. ***Thorax*.** Pronotum medium brown, lateral margins dark brown as well as medio anteriorly. Mesonotum pale brown washed with dark brown as in Fig. 36B. Femur greyish brown, except the base whitish. Tibia yellowish grey, apex of fore tibia blackish. Tarsi pale brown. ***Abdomen*.** Terga dark brown, with a thin sagittal line lighter on segments V–VII, becoming wider on segments VIII–X, less visible on mature nymphs. Sterna greyish brown, segments VIII–IX darker. Gills greyish purple. Terminal filaments yellowish.

##### Description.

***Head*. *Labrum*** cordiform (Fig. 37A). Ratio labrum width/clypeus width 0.99–1.10. Ratio labrum width/insertion width 1.35–1.53. Medial emargination shallow with flat denticles. Proximal row of setae present, simple. Number of setae on proximal row ~ 45–61. Distal row of setae present, simple. Median part of labrum in ventral view with scattered thin setae (Fig. 37B). ***Mandibles*.** Outer margin with row of setae on distal 1/2. Ending of the row near incisor. Right mandible with 6–10 setae below the mola (Fig. 37C). ***Maxillae*.** Posterior margin of cardo with few hair-like and stout setae, together with one long and stout seta in submarginal position. Proximo-lateral margin of stipes with a bunch of six stout setae increasing in size distally. Apical-ventral row with 21–24 pectinate setae. Maxillary palp segment II 0.83–0.91 as long as segment I. Maxillary palp segment III 0.67–0.82 as long as segment I (Fig. 37D). Stout setae on outer margin of segment I absent. Segment II inner margin with 2–4 stout setae. Segment II outer margin with 1–4 stout setae. Segment III inner margin with 4–6 stout setae, dorsal surface with few setae at apex. Segment III 1.83–2.00 × as long as base width. ***Labium*.** Labial palp segment II 0.72–0.80 as long as segment I. Labial palp segment III 0.78–0.88 as long as segment I (Fig. 37E). Segment I inner margin with 10–15 stout setae, outer margin with 9–12 stout setae. Segment II outer margin with four or five stout setae. Segment III dorsal face with 3–5 stout setae. Segment III 3.00–3.86 × as long as base width. ***Hypopharynx*.** Apex of superlingua truncate (Fig. 37F).

***Thorax*.** Dorsal margin of femur with a row of very long, stout, pointed setae; an irregular submarginal row of pointed setae as long as or a little bit shorter than those on the dorsal margin on fore femur, more regular on mid- and hind femur; ventral margin with short, stout setae and an irregular submarginal row of long, pointed stout setae; central area of upper surface with few long and short, stout, pointed setae (Fig. 38A–B). Fore tibia with two rows of long simple or feathered setae on ventral margin, dorsal margin with few hair-like setae; middle tibia with a row of short, simple, pointed setae on ventral margin and a submarginal row of long, simple setae, outer margin with numerous hair-like setae; hind tibia with two rows of simple and feathered long, stout setae on ventral margin, outer margin with short and long pointed stout setae and numerous hair-like setae. Tarsal claw slightly hooked with 6–8 denticles progressively larger distally (Fig. 38C).

***Abdomen*.** Gills I and II with single lamella (Fig. 38E–F). Gills III–VII with dorsal and ventral lamellae, ventral lamella smaller. Gill I with three filaments on dorsal lamella, without ventral lamella, gill II with four or five filaments on dorsal lamella, without ventral lamella, gills III–VI with 5–8 filaments on dorsal lamella and four or five on ventral lamella (Fig. 38G), gill VII with four or five filaments on dorsal lamella and one or two filaments on ventral lamella (Fig. 38H). Posterior margin of terga IX with minute denticles (Fig. 38D). Posterior margin of terga V–VIII without denticles. Posterolateral projections present on segments VIII–IX.

##### Derivatio nominis.

This species is dedicated to Prof. Nadir Alvarez, current director of Naturéum and instigator of the Cozzarolo et al. paper who revealed the diversity of New Guinean Thraulini.

#### ﻿ Nonnullidens anga sp. nov.

Taxon classificationAnimaliaEphemeropteraLeptophlebiidae

E532969E-6CC7-50D2-BDC3-8D5AE062D693

https://zoobank.org/D7CF7D8A-EF53-4573-9115-EFD4B9EA8116

Figs 35A , 39 , 40

##### Material examined.

***Holotype*.** • Papua New Guinea, one nymph on slide, Morobe Province, Menyamya, Mt Inji, 1700 m, 14.XI.2006, approx. 07°14.813'S, 146°01.330'E, M. Balke & Kinibel col [PNG96], GBIFCH01223129 (ZSM). ***Paratype*.** • Papua New Guinea, one nymph in ethanol, GBIFCH01523458, same data as holotype (MZL).

##### Nymph.

Body length, female: 4.8 mm.

##### Diagnosis.

Labrum cordiform; dorsal face of labrum with proximal and distal rows multiple; femora pale brown with two transverse dark brown bands in middle and at apex; gill I with ventral lamella composed of a single filament; gill II–VI with six or seven filaments on dorsal lamella and two or three on ventral lamella; gill VII with four or five filaments on dorsal lamella and a single one on ventral lamella.

##### Colouration.

***Head*.** Vertex greyish brown, with characteristic yellowish maculae posteriorly, frons greyish brown with a middle pale brown oval macula. Antenna: scape greyish, pedicel and flagellum broken. Mouth parts pale brown. ***Thorax*.** Pronotum pale brown, washed with medium brown laterally and medio-anteriorly. Mesonotum pale brown, washed with medium brown as in Fig. 35A. Femur yellowish brown, with a large median greyish brown stripe and another one at apex. Tibia yellowish. Tarsi pale brown. ***Abdomen*.** Terga dark brown, segments VII–X somewhat lighter, pale brown along the sagittal line. Sterna yellowish grey, yellowish on lateral margins. Gills greyish brown. Terminal filaments yellowish, basally yellowish brown.

##### Description.

***Head*. *Labrum*** cordiform (Fig. 39A). Ratio labrum width/clypeus width 1.05. Ratio labrum width/insertion width 1.36. Medial emargination narrow with flat denticles. Proximal row of setae present, multiple. Number of setae on proximal row ~ 104. Distal row of setae multiple. Median part of labrum in ventral view with scattered thin setae (Fig. 39B). ***Mandibles*.** Outer margin with row of setae on distal 1/2. Ending of the row near incisor. Right mandible with seven setae below the mola (Fig. 39C). ***Maxillae*.** Posterior margin of cardo with many long hair-like setae. Proximo-lateral margin of stipes twisted. Apical-ventral row with 23 pectinate setae. Maxillary palp segment II 0.88 as long as segment I. Maxillary palp segment III 0.68 as long as segment I (Fig. 39D). Stout setae on outer margin of segment I absent. Segment III dorsal surface half covered with setae. Segment III 1.55 × as long as base width. ***Labium*.** Labial palp segment II 0.82 as long as segment I. Labial palp segment III 0.71 as long as segment I (Fig. 39E). Segment I inner margin with nine stout setae, outer margin with 14 stout setae. Segment II outer margin with four stout setae. Segment III dorsal face with four stout setae. Segment III 2.67 × as long as base width. ***Hypopharynx*.** Apex of superlingua truncate.

***Thorax*.** Dorsal margin of femur with a row of very long, thin, pointed setae; regular submarginal row of pointed setae as long as those on the dorsal margin; ventral margin with relatively long, pointed setae and an irregular submarginal row of long, pointed thin setae (Fig. 40A–B); central area of upper surface with very long pointed setae, as long as those on the dorsal margin. Fore tibia with two or three rows of long simple or feathered setae on ventral margin, dorsal margin with few hair-like setae; middle tibia with a row of short, simple, pointed setae on ventral margin and a submarginal row of long, simple setae, outer margin with numerous hair-like setae; hind tibia with a marginal row of simple short, thin setae and a submarginal row of long to very long setae on ventral margin, outer margin with long to very long pointed setae and hair-like setae (Fig. 40C). Tarsal claw hooked with three or four denticles progressively larger, except distal much larger.

***Abdomen*.** Gill I with both lamellae. Gills II–VII with dorsal and ventral lamellae, ventral lamella smaller. Gill I with three filaments on dorsal lamella, ventral lamella with a single filament (Fig. 40E); gill II–VI with six or seven filaments on dorsal lamella and two or three on ventral lamella (Fig. 40F); gill VII with four or five filaments on dorsal lamella and a single one on ventral lamella (Fig. 40G). Posterior margin of terga IX with minute denticles (Fig. 40D). Posterior margin of terga V–VIII without denticles. Posterolateral projections present on segments VIII–IX.

##### Derivatio nominis.

The specific name is to recognize the Anga people who live in the area where the new species has been found and is a noun in apposition.

#### ﻿ Nonnullidens boonsoongi sp. nov.

Taxon classificationAnimaliaEphemeropteraLeptophlebiidae

635CB6BF-9057-5845-9748-8555C7AF9BC6

https://zoobank.org/D859EC1C-8301-4AE7-950E-93103A864EC5

Figs 36A , 41 , 42

##### Material examined.

***Holotype*.** • Papua New Guinea, one nymph on slide, Central Province, Kokoda Trek, 1400 m, I. 2008, 09°01.952'S, 147°44.455'E, Posman col [PNG172], GBIFCG01223130 (ZSM). ***Paratypes*.** two nymphs on slide, GBIFCH01223131, GBIFCH01223132, 10 nymphs in ethanol, GBIFCH01523459, same data as holotype (MZL). ***Other material***. • Papua New Guinea, one nymph on slide, GBIFCH01223133, gills and legs in ethanol, GBIFCH01523460, Western Highlands, Simbai area, 2500 m, 8. III.2007, 05°14.202'S, 144°33.651'E, Kinibel col [PNG150] (MZL).

##### Nymph.

Body length, male: 5 mm; female: 5.5 mm.

##### Diagnosis.

Femora uniformly dark brown, without transverse bands; tarsal claw with 6–8 teeth; gill I and II uni-lamellate, without ventral lamella; gills III–VI with five or six filaments on dorsal lamella and a single or two filaments on ventral lamella, gill VII with three filaments on dorsal lamella and a single filament on ventral lamella.

##### Colouration.

***Head*.** Vertex dark brown, with characteristic yellowish maculae posteriorly, pale brown between lateral ocelli and eyes, frons medium brown with a middle pale brown oval macula. Antenna broken. Compound eye upper portion reddish brown. Mouth parts pale brown. ***Thorax*.** Pronotum medium brown, dark brown laterally and anteromedially as in Fig. 36A. Mesonotum pale brown washed with dark brown. Femur greyish brown, whitish at base. Tibia yellowish, apex greyish brown on fore- and mid-tibia. Tarsi yellowish brown. ***Abdomen*.** Terga dark brown, paler along the sagittal line on segments VII–IX. Sterna pale brown, greyish brown in the middle, pale brown on each side of a sagittal line. Gills greyish brown. Terminal filaments yellowish.

##### Description.

***Head*. *Labrum*** cordiform (Fig. 41A). Ratio labrum width/clypeus width 0.99–1.04. Ratio labrum width/insertion width 1.36–1.45. Medial emargination shallow with flat denticles. Proximal row of setae present, simple. Number of setae on proximal row ~ 42–69. Distal row of setae present, simple. Median part of labrum in ventral view with scattered thin setae (Fig. 41B). ***Mandibles*.** Outer margin with row of setae on distal 1/2. Ending of the row near incisor. Right mandible with eight or nine setae below the mola (Fig. 41C). ***Maxillae*.** Posterior margin of cardo with few hair-like setae, together with two stout and long setae in submarginal position. Proximo-lateral margin of stipes with a bunch of five stout setae of irregular length. Apical-ventral row with 22–24 pectinate setae. Maxillary palp segment II 0.86–1.00 as long as segment I. Maxillary palp segment III 0.64–0.95 as long as segment I (Fig. 41D). Stout setae on outer margin of segment I absent. Segment II inner margin with two or three stout setae. Segment II outer margin without stout setae. Segment III inner margin with four stout setae, dorsal surface with few setae at apex. Segment III 1.67–2.00 × as long as base width. ***Labium*.** Labial palp segment II 0.70–0.80 as long as segment I. Labial palp segment III 0.74–0.80 as long as segment I (Fig. 41E). Segment I inner margin with 13–15 stout setae, outer margin with 12–15 stout setae. Segment II outer margin with three or four stout setae. Segment III dorsal face with 3–5 stout setae. Segment III 2.91–3.30 × as long as base width. ***Hypopharynx*.** Apex of superlingua truncate (Fig. 41F).

***Thorax*.** Dorsal margin of femur with few long, stout, pointed setae; an irregular submarginal row of pointed setae as long as or a little bit shorter than those on the dorsal margin; ventral margin with short, stout setae and an irregular submarginal row of long, pointed stout setae; central area of upper surface with numerous long and short, stout, pointed setae (Fig. 42A). Fore tibia with several rows of long simple or feathered setae on ventral margin, dorsal margin with few hair-like setae; middle tibia with a row of short, simple, pointed setae on ventral margin and a submarginal row of long, simple setae, dorsal margin with few hair-like setae; hind tibia with two rows of simple and feathered long, stout setae on ventral margin, dorsal margin with short and long pointed stout setae and few hair-like setae. Tarsal claw slightly hooked with seven or eight denticles progressively larger apically (Fig. 42B).

***Abdomen*.** Gill I–II with single lamella. Gills III–VII with dorsal and ventral lamellae, ventral lamella smaller. Gill I uni-lamellate, with two or three filaments (Fig. 42D), gill II uni-lamellate with four filaments (Fig. 42E), gills III–VI with five or six filaments on dorsal lamella and a single or two filaments on ventral lamella (Fig. 42F), gill VII with three filaments on dorsal lamella and a single filament on ventral lamella (Fig. 42G). Posterior margin of terga IX with minute denticles (Fig. 42C). Posterior margin of terga V–VIII without denticles. Posterolateral projections present on segments VIII–IX.

##### Derivatio nominis.

This species is dedicated to Prof. Boonsatien Boonsoong (Kasetsart University, Bangkok, Thailand) for his outstanding contributions to our knowledge of Southeast Asian Ephemeroptera.

#### ﻿ Nonnullidens cozzarolae sp. nov.

Taxon classificationAnimaliaEphemeropteraLeptophlebiidae

7E5B29C3-9383-5B07-AF5F-0BB0881451AC

https://zoobank.org/63C20EB0-B347-4FF7-BB59-78751985C53E

Figs 35C , 43 , 44

##### Material examined.

***Holotype*.** • Papua New Guinea, one nymph on slide, Eastern Highlands Province, Marawaka Ande, 1700–1800 m, 9.XI.2006, approx. 07°01.697'S, 145°49.807'E, M. Balke & Kinibel col [PNG87], GBIFCH01223134 (ZSM). ***Paratypes*.** • Papua New Guinea, 2 nymphs on slide, GBIFCH01223135, GBIFCH01223136, 5 nymphs in ethanol, GBIFCH01523461, same data as holotype (MZL). • Papua New Guinea, 3 nymphs on slide, GBIFCH01223137, GBIFCH01223138, GBIFCH01223139, one nymph in ethanol, GBIFCH01523462, Central Province, Kokoda Trek, 1390 m, I. 2008, 09°00.338'S, 147°44.252'E, Posman col [PNG173] (MZL). • Papua New Guinea, one nymph on slide GBIFCH01223140, 2 nymphs in ethanol, GBIFCH01523463 Western Highlands Province, Kundum, 1400 m, 3.III.2007, 05°16.096'S, 144°27.869'E, Kinibel col [PNG142] (MZL).

##### Nymph.

Body length, male: 5 mm; female: 5.25 mm.

##### Diagnosis.

Femora uniformly dark brown, without transverse bands; gill I with ventral lamella composed of one or two filaments; gills II–VI with four to six filaments on dorsal lamella and three or four on ventral lamella, gill VII with 2–4 filaments on each lamella; posterolateral projections present on segments VII–IX.

##### Colouration.

***Head*.** Vertex greyish brown, with characteristic yellowish maculae posteriorly, frons medium brown with a middle pale brown oval macula. Antenna: scape yellowish, pedicel and flagellum pale brown. Compound eye upper portion reddish brown. Mouth parts pale brown. ***Thorax*.** Pronotum pale brown, dark brown laterally and medio-anteriorly. Mesonotum greyish brown laterally, with a pale brown central area as in Fig. 35C. Femur greyish brown, lighter at base. Fore tibia greyish brown, mid- and hind tibia pale brown, apex greyish brown. Tarsi pale brown. ***Abdomen*.** Terga dark brown, with a pale brown macula of the middle of segments IV–IX, increasing in size posteriorly, segment X almost completely pale brown. Sterna pale brown, greyish brown in the middle on each side of a sagittal line pale brown. Gills greyish brown. Terminal filaments yellowish to pale brown.

##### Description.

***Head*. *Labrum*** cordiform (Fig. 43A). Ratio labrum width/clypeus width 1.06–1.13. Ratio labrum width/insertion width 1.46–1.56. Medial emargination shallow with flat denticles. Proximal row of setae present, simple. Number of setae on proximal row ~ 33–58. Distal row of setae present, simple. Median part of labrum in ventral view with only scattered stout setae (Fig. 43B). ***Mandibles*.** Outer margin with row of setae on distal 1/2. Ending of the row near incisor. Right mandible with 7–10 setae below the mola (Fig. 43C). ***Maxillae*.** Posterior margin of cardo with few hair-like and some stout setae, together with one long and stout seta in submarginal position. Proximo-lateral margin of stipes with a bunch of ten stout medium size and short setae. Apical-ventral row with 22–29 pectinate setae. Maxillary palp segment II 0.80–1.00 as long as segment I. Maxillary palp segment III 0.68–0.86 as long as segment I (Fig. 43D). Stout setae on outer margin of segment I absent. Segment II inner margin with four or five stout setae. Segment II outer margin without or with one stout seta. Segment III inner margin with 3–5 stout setae, dorsal surface with few setae at apex. Segment III 1.57–1.83 × as long as base width. ***Labium*.** Labial palp segment II 0.71–0.94 as long as segment I. Labial palp segment III 0.73–0.81 as long as segment I (Fig. 43E). Segment I inner margin with 10–12 stout setae, outer margin with 10–12 stout setae. Segment II outer margin with 3–5 stout setae, dorsal face with 3–5 stout setae. Segment III 2.90–3.40 × as long as base width. ***Hypopharynx*.** Apex of superlingua truncate (Fig. 43F).

***Thorax*.** Dorsal margin of femur with a row of long, stout, pointed setae; a regular submarginal row of pointed setae as long as or a little bit shorter than those on the dorsal margin; ventral margin with short, stout setae and an irregular submarginal row of long, pointed stout setae; central area of upper surface with numerous long and short, stout, pointed setae (Fig. 44A–B). Fore tibia with several rows of long simple or feathered setae on ventral margin, dorsal margin with numerous hair-like setae; middle tibia with a row of short, simple, pointed setae on ventral margin and a submarginal row of long, simple setae, dorsal margin with numerous hair-like setae; hind tibia with two rows of simple long, stout setae on ventral margin, dorsal margin with short and long pointed stout setae and numerous hair-like setae. Tarsal claw slightly hooked with 4–6 denticles progressively larger apically (Fig. 44C).

***Abdomen*.** Gill I with both lamellae. Gills II–VII with dorsal and ventral lamellae, ventral lamella smaller. Gill I with three or four filaments on dorsal lamella, ventral lamella with a single or two filaments (Fig. 44E), gills II–VI with 4–6 filaments on dorsal lamella and three or four on ventral lamella (Fig. 44F), gill VII with 2–4 filaments on each lamella (Fig. 44G). Posterior margin of terga IX with minute denticles (Fig. 44D). Posterior margin of terga V–VIII without denticles. Posterolateral projections present on segments VII–IX.

##### Derivatio nominis.

This species is dedicated to Dr Camille-Sophie Cozzarolo, who performed the first genetic work which revealed the diversity of New Guinean Thraulini.

#### ﻿ Nonnullidens fuyugensis sp. nov.

Taxon classificationAnimaliaEphemeropteraLeptophlebiidae

B6062997-E0F3-5C1A-8C13-19CB3E686F78

https://zoobank.org/7B72BFEB-75C3-4B37-BC81-01EDEE1D2CA7

**Figure 34.**
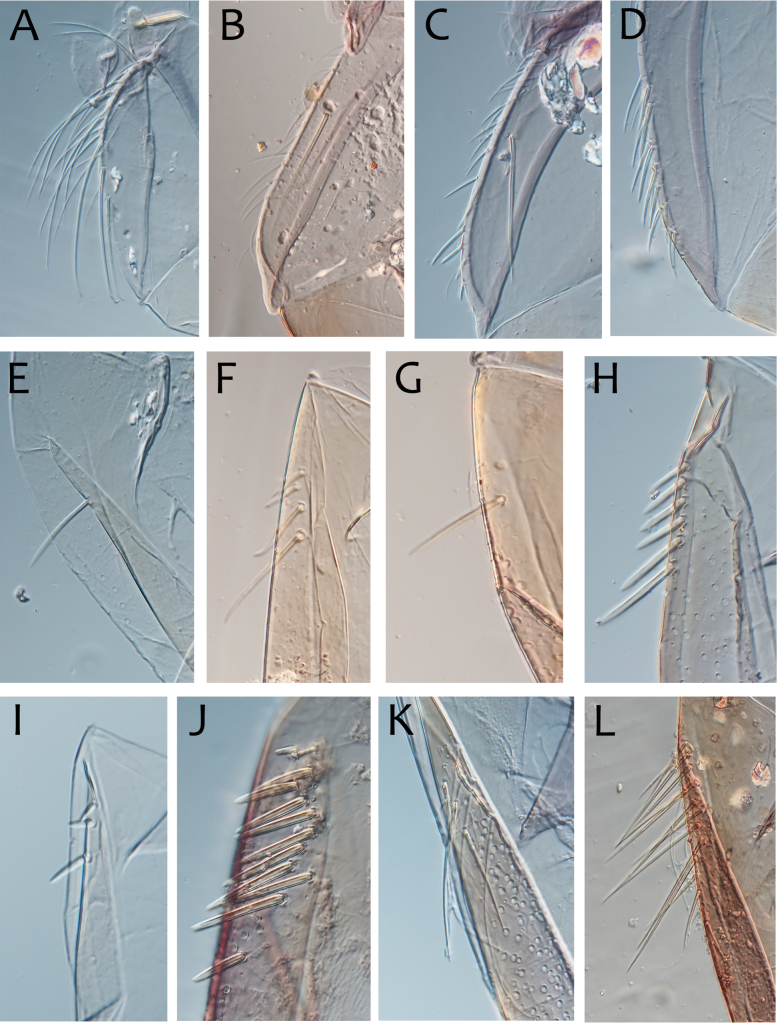
Maxillae: posterior margin of cardo (**A–D**) and proximo-lateral margin of stipes (**E–L**). **A.**Thraulus (Thraulus) eloisae sp. nov.; **B.**Thraulus (Masharikella) iteris sp.nov.; **C.***Nonnullidensfuyugensis* sp.nov. **D***Kosminymphakalamorum* sp. nov.; **E.**Thraulus (Thraulus) timorensis sp. nov.; **F.**Thraulus (Masharikella) johannisluci sp. nov. **G**Thraulus (Masharikella) pascalae sp. nov.; **H.***Nonnullidens* sp. A; **I.***Nonnullidensmoiorum* sp.nov.; **J.***Kosminymphapaulinae* sp. nov.; **K.***Kosminymphabalkei* sp. nov.; **L.***Kosminymphabaruya* sp. nov.

**Figure 35.**
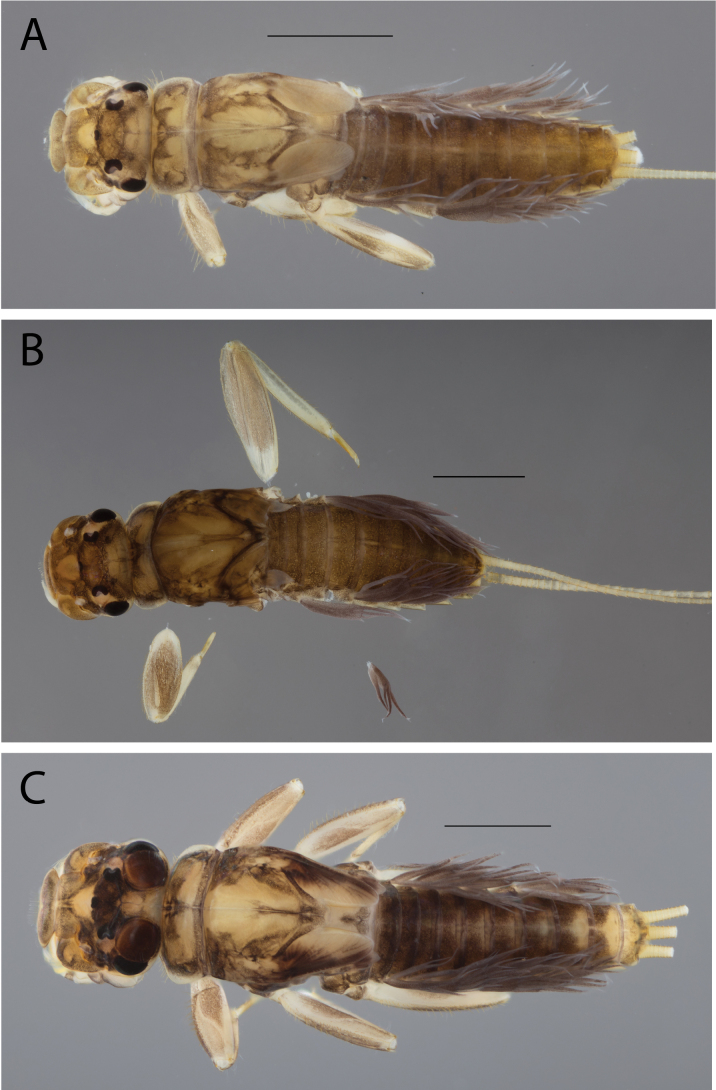
Habitus of *Nonnullidensanga* sp. nov. (**A**), *Nonnullidensfuyugensis* sp. nov. (**B**) and *Nonnullidenscozzarolae* sp. nov. (**C**). Scale bars: 1 mm.

Figs 34C , 35B , 45 , 46 , 64C, D

##### Material examined.

***Holotype*.** • Papua New Guinea, one nymph on slide, Central Province, Woitape, 1700 m, I. 2008, 08°31.290'S, 147°13.684'E, Posman col [PNG166], GBIFCH01223141 (ZSM). ***Paratypes*.** • Papua New Guinea, one nymph on slide GBIFCH01223142, 4 nymphs in ethanol, GBIFCH01523464, same data as holotype (MZL).

##### Nymph.

Body length, female: 4.5–5.0 mm.

##### Diagnosis.

Femora uniformly dark brown, without transverse bands; gill I uni-lamellate, without ventral lamella; gills II–VI with four or five filaments on dorsal lamella and two filaments on ventral lamella, gill VII with three or four filaments on dorsal lamella and a single one on ventral lamella.

##### Colouration.

***Head*.** Vertex greyish brown, with characteristic yellowish maculae posteriorly, pale brown between lateral ocelli and eyes, frons medium brown with a middle pale brown oval macula. Antenna broken. Mouth parts pale brown. ***Thorax*.** Pronotum medium brown, greyish brown laterally and antero-medially. Mesonotum pale brown, washed with greyish brown laterally (Fig. 35B). Femur greyish brown, whitish at base. Tibia yellowish to pale brown. Tarsi greyish brown. ***Abdomen*.** Terga dark brown, with a paler sagittal line increasing in width on segments VIII–X. Sterna yellowish brown to greyish brown. Gills greyish purple. Terminal filaments yellowish.

##### Description.

***Head*. *Labrum*** cordiform (Fig. 45A). Ratio labrum width/clypeus width 1.04–1.10. Ratio labrum width/insertion width 1.45–1.53. Medial emargination shallow with flat denticles. Proximal row of setae present, simple. Number of setae on proximal row ~ 36–37. Distal row of setae present, simple. Median part of labrum in ventral view with scattered thin setae (Fig. 45B). ***Mandibles*.** Outer margin with row of setae on distal 1/2. Ending of the row near incisor. Right mandible with 7–9 setae below the mola (Fig. 45C). ***Maxillae*.** Posterior margin of cardo with few hair-like and some stout setae (Fig. 34C). Proximo-lateral margin of stipes with a bunch of six stout medium size and long setae. Apical-ventral row with 23–27 pectinate setae. Maxillary palp segment II 0.79–0.80 as long as segment I. Maxillary palp segment III 0.79–0.80 as long as segment I (Fig. 45D). Stout setae on outer margin of segment I absent. Segment II inner margin with three or four stout setae. Segment II outer margin without stout setae. Segment III inner margin with five stout setae, dorsal surface with few setae at apex. Segment III 1.69–2.10 × as long as base width. ***Labium*.** Labial palp segment II 0.84–0.86 as long as segment I. Labial palp segment III 0.81–0.84 as long as segment I (Fig. 45E). Segment I inner margin with 11–14 stout setae, outer margin with 14 stout setae. Segment II outer margin with four or five stout setae. Segment III dorsal face with three or four stout setae. Segment III 3.20–3.40 × as long as base width. ***Hypopharynx*.** Apex of superlingua truncate (Fig. 45F).

**Figure 36.**
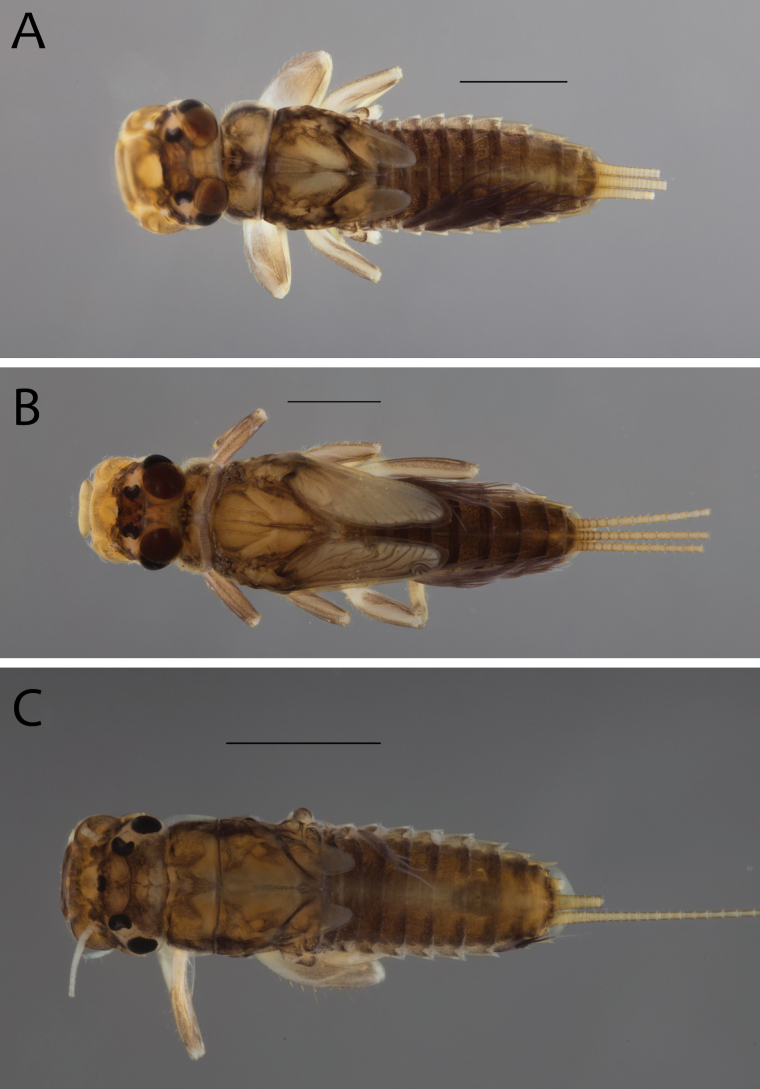
Habitus of *Nonnullidensboonsoongi* sp. nov. (**A**), *Nonnullidensalvarezi* sp. nov. (**B**) and *Nonnullidensmoiorum* sp. nov. (**C**). Scale bars: 1 mm.

**Figure 37.**
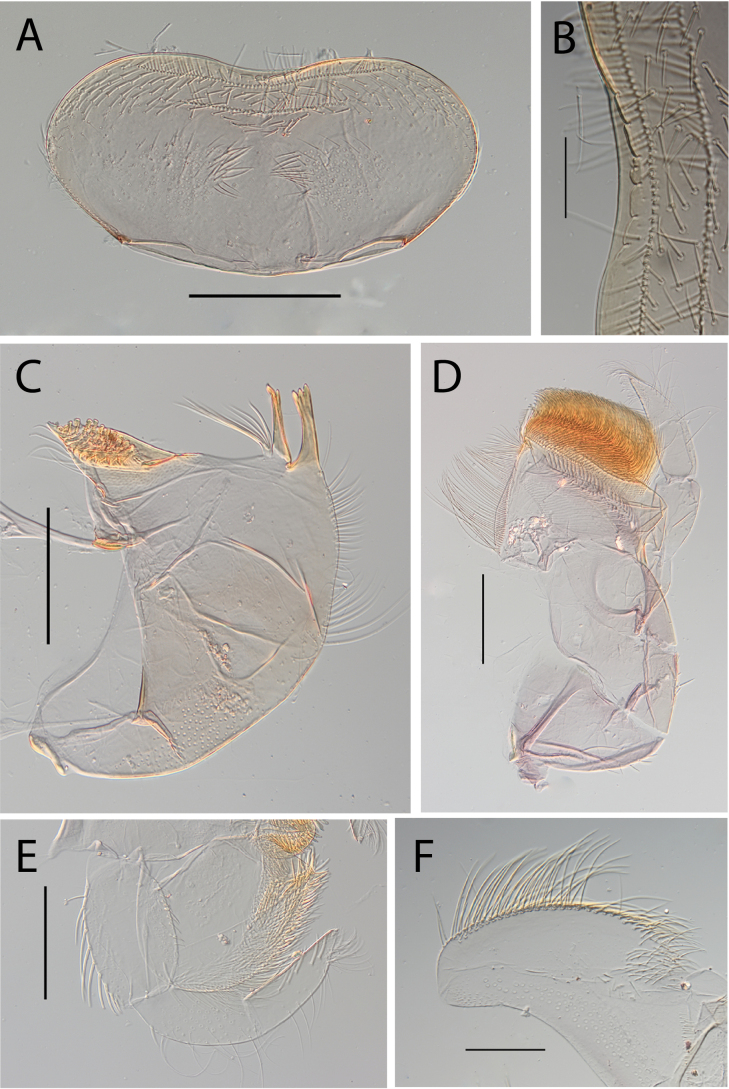
*Nonnullidensalvarezi* sp. nov. nymphal mouthparts. **A.** Labrum, dorsal view; **B.** Emargination of the labrum; **C.** Right mandible; **D.** Maxilla; **E.** Labium; **F.** Superlingua of hypopharynx. Scale bars: 200 µm (**A, C–E**); 50 µm (**B**); 100 µm (**F**).

**Figure 38.**
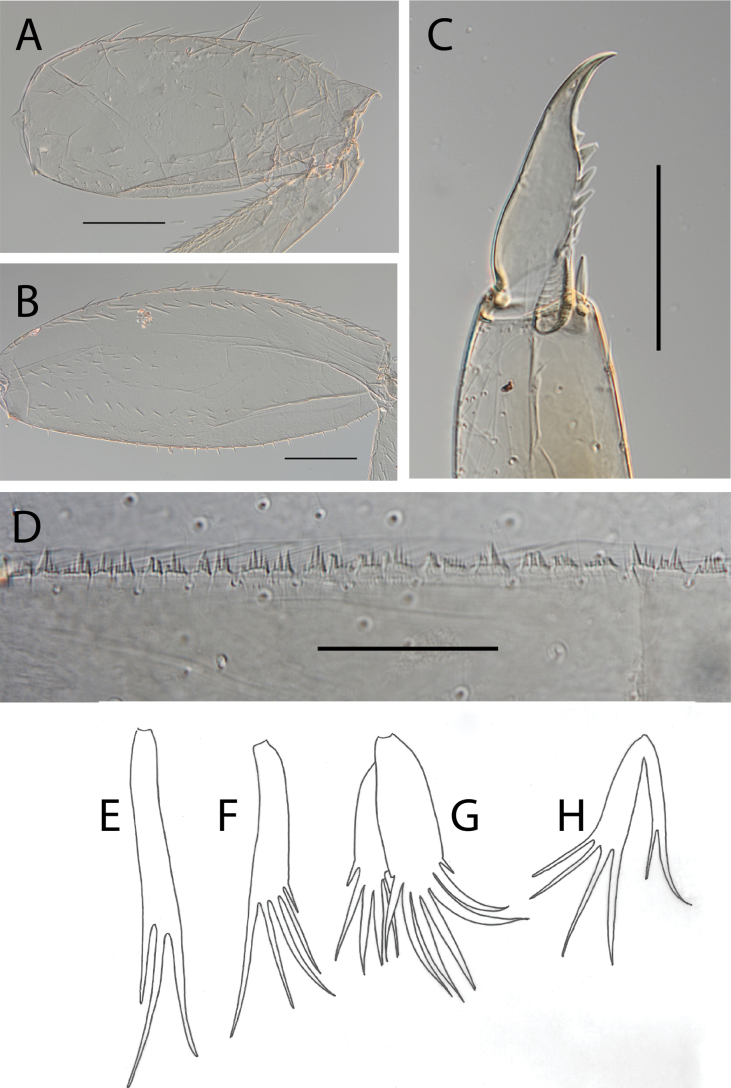
*Nonnullidensalvarezi* sp. nov. thorax and abdomen. **A.** Fore femur; **B.** Hind femur; **C.** Claw; **D.** Posterior margin of tergite IX; **E.** Gill I, **F.** Gill II; **G.** Gill IV; **H.** Gill VII. Scale bars: 200 µm (**A, B**); 50 µm (**C, D**).

**Figure 39.**
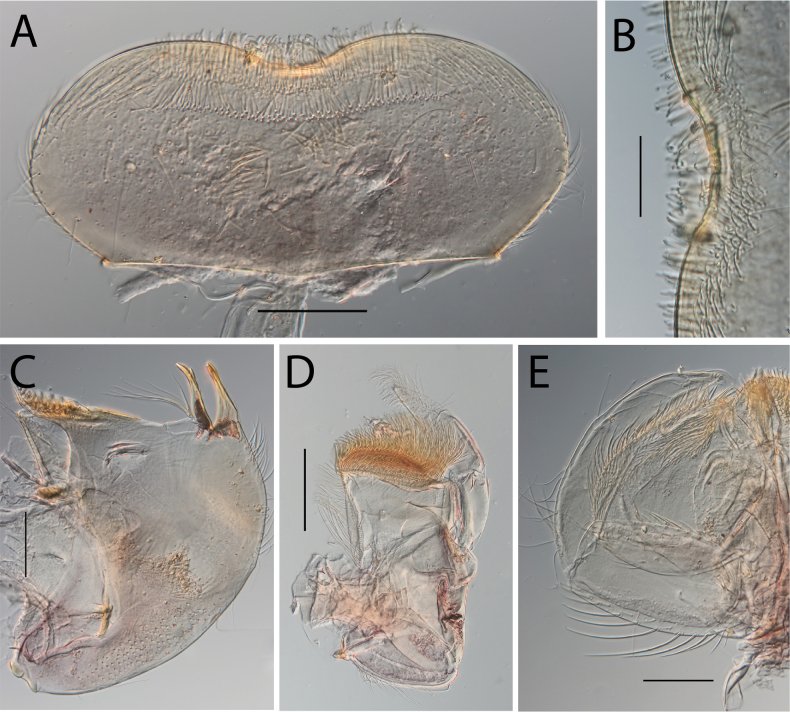
*Nonnullidensanga* sp. nov. nymphal mouthparts. **A.** Labrum, dorsal view; **B.** Emargination of the labrum; **C.** Right mandible; **D.** Maxilla; **E.** Labium. Scale bars: 100 µm (**A, C, E**); 50 µm (**B**); 200 µm (**D**).

**Figure 40.**
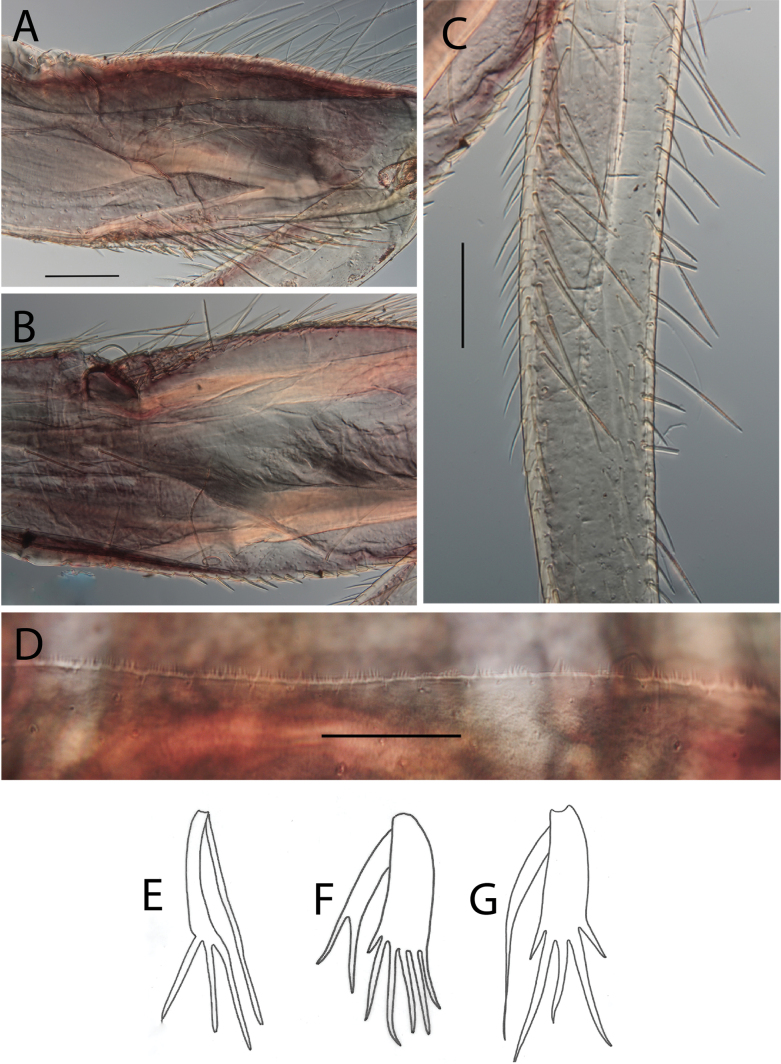
*Nonnullidensanga* sp. nov. thorax and abdomen. **A.** Fore femur; **B.** Hind femur; **C.** Detail of hind tibia; **D.** Posterior margin of tergite IX; **E.** Gill I; **F.** Gill IV; **G.** Gill VII. Scale bars: 100 µm (**A–C**); 50 µm (**D**).

**Figure 41.**
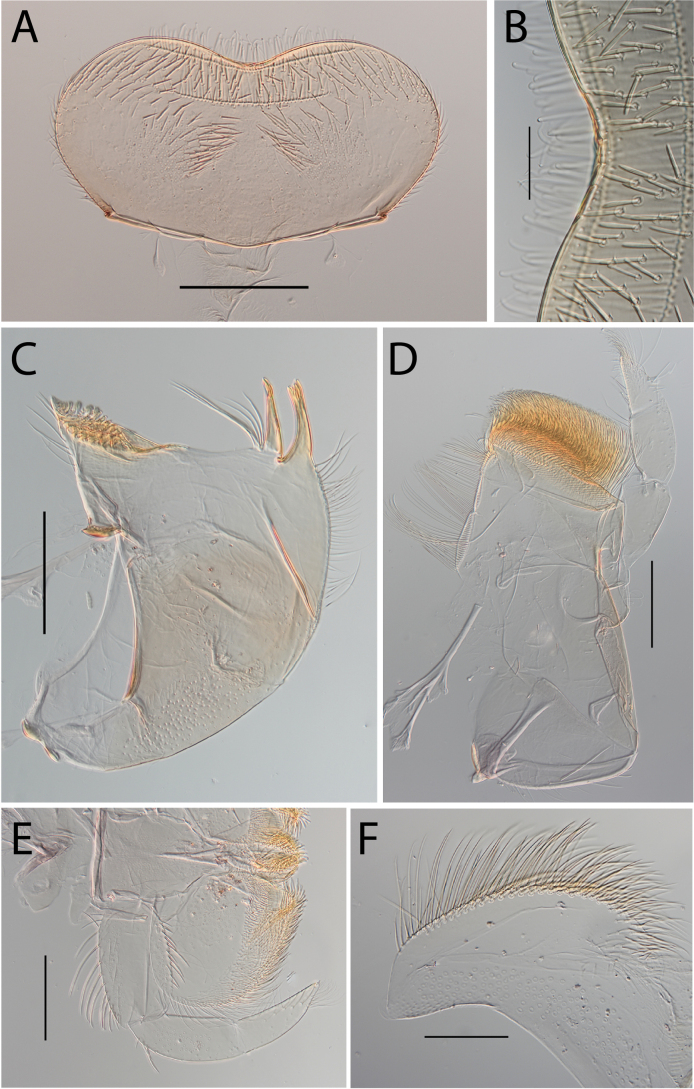
*Nonnullidensboonsoongi* sp. nov. nymphal mouthparts. **A.** Labrum, dorsal view; **B.** Emargination of the labrum; **C.** Right mandible; **D.** Maxilla; **E.** Labium; **F.** Superlingua of hypopharynx. Scale bars: 200 µm (**A, C–E**); 50 µm (**B**); 100 µm (**F**).

**Figure 42.**
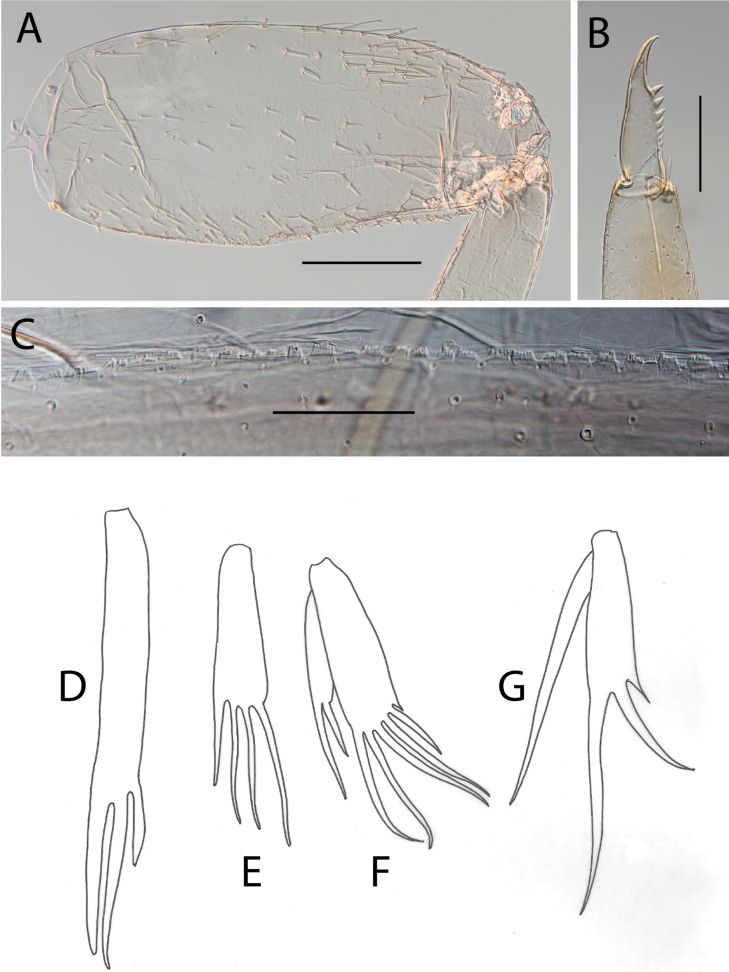
*Nonnullidensboonsoongi* sp. nov. thorax and abdomen. **A.** Fore femur; **B.** Claw; **C.** Posterior margin of tergite IX; **D.** Gill I; **E.** Gill II; **F.** Gill IV; **G.** Gill VII. Scale bars: 200 µm (**A**); 50 µm (**B, C**).

**Figure 43.**
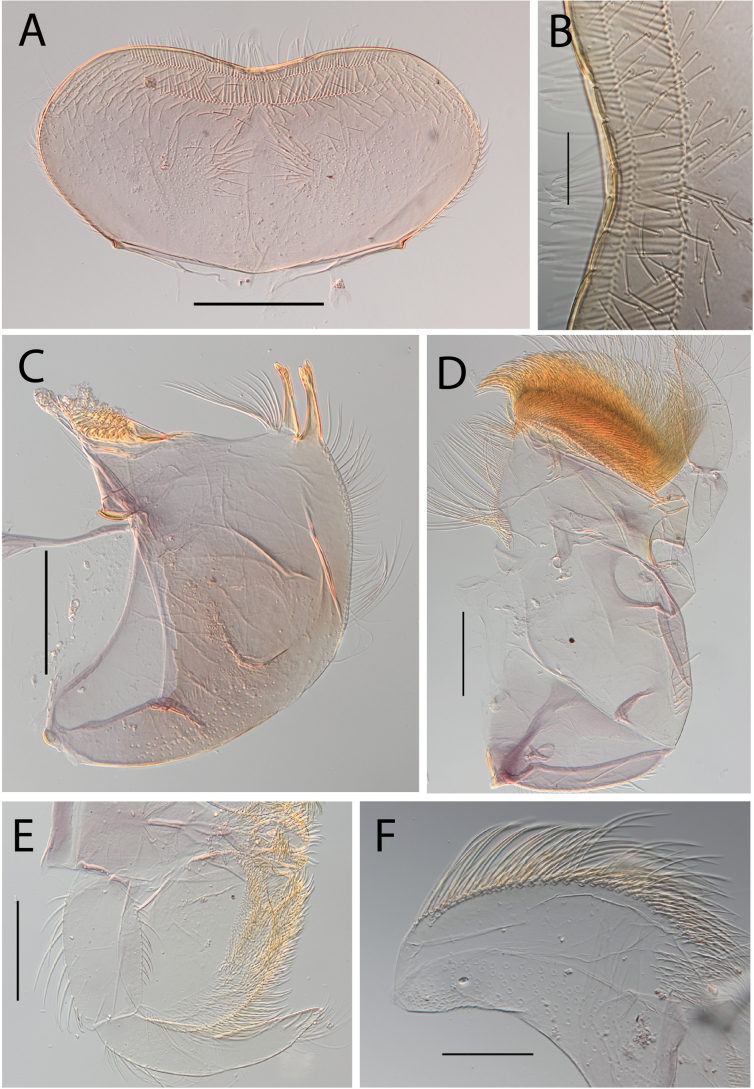
*Nonnullidenscozzarolae* sp. nov. nymphal mouthparts. **A.** Labrum, dorsal view; **B.** Emargination of the labrum; **C.** Right mandible; **D.** Maxilla; **E.** Labium; **F.** Superlingua of hypopharynx. Scale bars: 200 µm (**A, C–E**); 50 µm (**B**); 100 µm (**F**).

**Figure 44.**
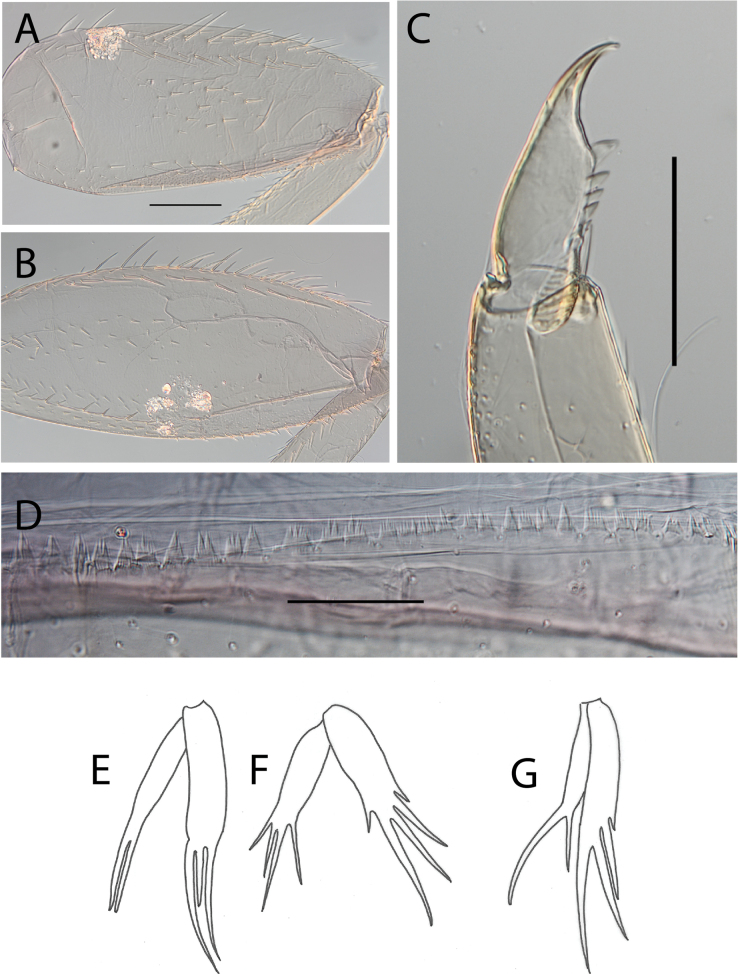
*Nonnullidenscozzarolae* sp. nov. thorax and abdomen. **A.** Fore femur; **B.** Hind femur; **C.** Claw; **D.** Posterior margin of tergite IX; **E.** Gill I; **F.** Gill IV; **G.** Gill VII. Scale bars: 200 µm (**A, B**); 50 µm (**C, D**).

**Figure 45.**
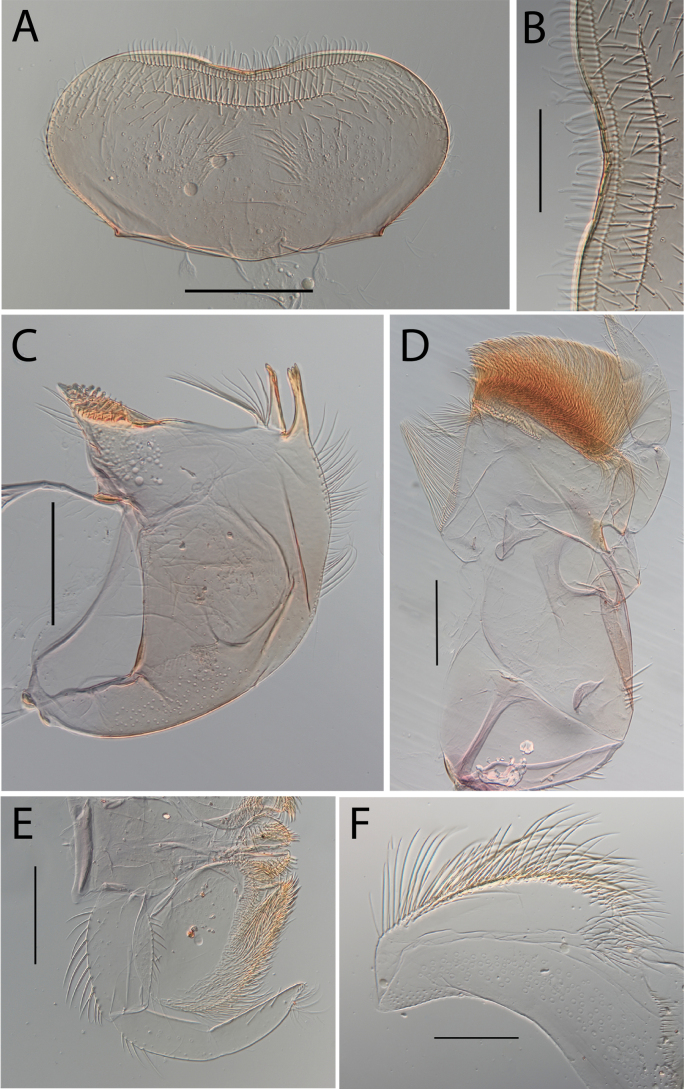
*Nonnullidensfuyugensis* sp. nov. nymphal mouthparts. **A.** Labrum, dorsal view; **B.** Emargination of the labrum; **C.** Right mandible; **D.** Maxilla; **E.** Labium; **F.** Superlingua of hypopharynx. Scale bars: 200 µm (**A, C–E**); 50 µm (**B**); 100 µm (**F**).

**Figure 46.**
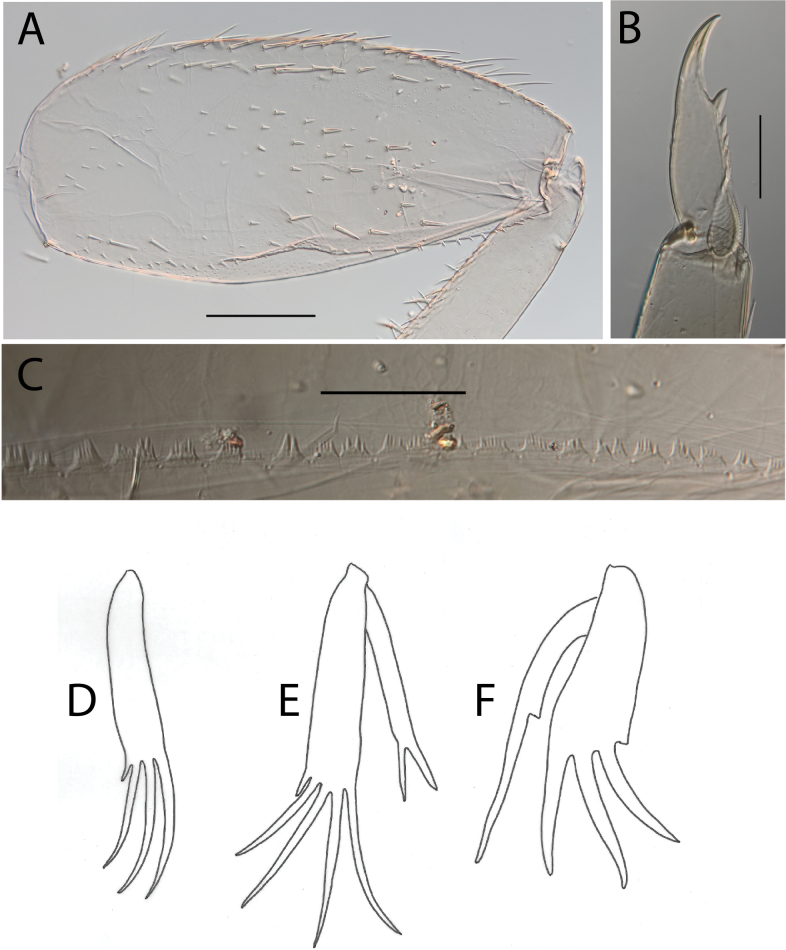
*Nonnullidensfuyugensis* sp. nov. thorax and abdomen. **A.** Fore femur; **B.** Claw; **C.** Posterior margin of tergite IX; **D.** Gill I; **E.** Gill IV; **F.** Gill VII. Scale bars: 200 µm (**A**); 50 µm (**B, C**).

***Thorax*.** Dorsal margin of femur with a row of long, stout, pointed setae; a regular submarginal row of pointed setae as long as or a little bit shorter than those on the dorsal margin, shorter on hind femur; ventral margin with short, stout setae and an irregular submarginal row of long, pointed stout setae; central area of upper surface with numerous long and short, stout, pointed setae (Fig. 46A). Fore tibia with several rows of long simple or feathered setae on ventral margin, dorsal margin with numerous hair-like setae; middle tibia with a row of short, simple, pointed setae on ventral margin and a submarginal row of long, simple setae, dorsal margin with numerous hair-like setae; hind tibia with two rows of simple and feathered long, stout setae on ventral margin, dorsal margin with short and long pointed stout setae and numerous hair-like setae. Tarsal claw slightly hooked with four or five denticles progressively larger apically (Fig. 46B).

***Abdomen*.** Gill I with single lamella. Gills II–VII with dorsal and ventral lamellae, ventral lamella smaller. Gill I uni-lamellate with three or four filaments (Fig. 46D), gills II–VI with four or five filaments on dorsal lamella and two filaments on ventral lamella (Fig. 46E), gill VII with three or four filaments on dorsal lamella and a single one on ventral lamella (Fig. 46F). Posterior margin of terga IX with minute denticles (Fig. 46C). Posterior margin of terga V–VIII without denticles. Posterolateral projections present on segments VIII–IX.

***Eggs*.** General shape rounded. Size 130 μm × 80 μm. Chorionic surface smooth without visible attachment structures (Fig. 64C). Micropyle in equatorial position (Fig. 64D).

##### Derivatio nominis.

The specific name honours the tribes of the Fuyug (Fuyughe) people, who live where the new species has been found.

#### ﻿ Nonnullidens kaltenbachi sp. nov.

Taxon classificationAnimaliaEphemeropteraLeptophlebiidae

33D473FC-81D4-5A31-AFA6-35E7F1E0115E

https://zoobank.org/C3C11F2D-11BD-4E96-9DF8-CE87A5C92490

Figs 47 , 48

##### Material examined.

***Holotype*.** • Papua New Guinea, one nymph on slide, Madang Province, Highway near Madang, ford, 80 m, 2–3.XII.2006, 05°24.405'S, 145°38.213'E, Binatang Boys col [PNG117], GBIFCH01223143, some gills and legs in ethanol, GBIFCH01523465 (ZSM). ***Paratype*.** • Papua New Guinea, one nymph on slide, GBIFCH01223144, Madang Province, Keki, Adalbert Mts, 400 m, 22.XI.2006, 04°43.058'S, 145°24.437'E, Binatang Boys col [PNG119] (MZL).

##### Diagnosis.

**Nymph.** Labrum emargination narrow and very deep with flat denticles; femora pale brown with two transverse dark brown bands in middle and at apex; central area of upper surface of femora with very few long and pointed setae; posterior margin of abdominal segments IX with minute denticles; posterolateral expansions on the abdomen present on segments VIII–IX.

##### Colouration.

Forefemur yellowish, washed with greyish brown in distal half, mid- and hind femur yellowish with a brownish band and in the middle and at apex. Tibia yellowish. Tarsi yellowish.

##### Description.

**Figure 47.**
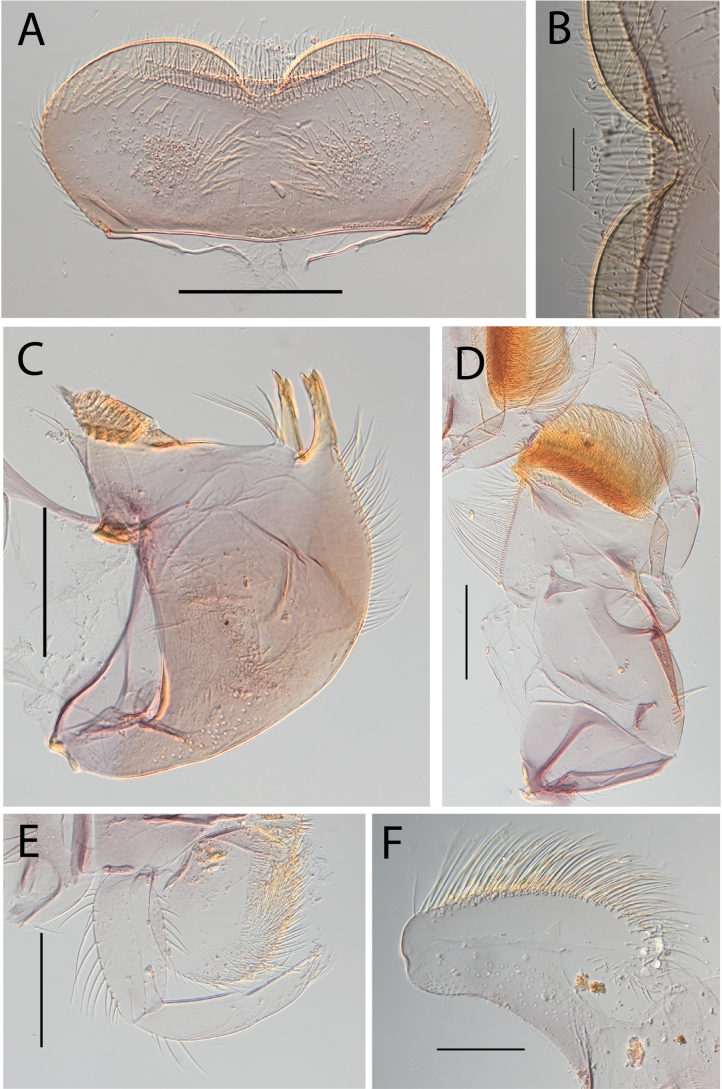
*Nonnullidenskaltenbachi* sp. nov. nymphal mouthparts. **A.** Labrum, dorsal view; **B.** Emargination of the labrum; **C.** Right mandible; **D.** Maxilla; **E.** Labium; **F.** Superlingua of hypopharynx. Scale bars: 200 µm (**A, C–E**); 50 µm (**B**); 100 µm (**F**).

***Head*. *Labrum*** cordiform (Fig. 47A). Ratio labrum width/clypeus width 1.03–1.07. Ratio labrum width/insertion width 1.40–1.42. Medial emargination deep, narrow with flat denticles. Proximal row of setae present, simple, shifted to distal margin. Number of setae on proximal row ~ 69–75. Distal row of setae present, simple. Median part of labrum in ventral view with scattered thin setae (Fig. 47B). ***Mandibles*.** Outer margin with row of setae on distal 1/2. Ending of the row near incisor. Right mandible with 4–6 setae below the mola (Fig. 47C). ***Maxillae*.** Posterior margin of cardo with few hair-like setae, together with one stout and long seta in submarginal position. Proximo-lateral margin of stipes with a bunch of six stout setae increasing in size distally, most distal much longer than the others. Apical-ventral row with 23–26 pectinate setae. Maxillary palp segment II 0.79–0.84 as long as segment I. Maxillary palp segment III 0.61 as long as segment I (Fig. 47D). Stout setae on outer margin of segment I absent. Segment II inner margin with one stout seta. Segment II outer margin without or with up to two stout setae. Segment III inner margin without or with up to three stout setae, dorsal surface half covered with setae. Segment III 1.25–1.50 × as long as base width. ***Labium*.** Labial palp segment II 0.68–0.86 as long as segment I. Labial palp segment III 0.58–0.66 as long as segment I (Fig. 47E). Segment I inner margin with 6–10 stout setae outer margin with 9–13 stout setae. Segment II outer margin with two or three stout setae, dorsal face with two or three stout setae. Segment III 2.71–2.75 × as long as base width. ***Hypopharynx*.** Apex of superlingua truncate (Fig. 47F).

**Figure 48.**
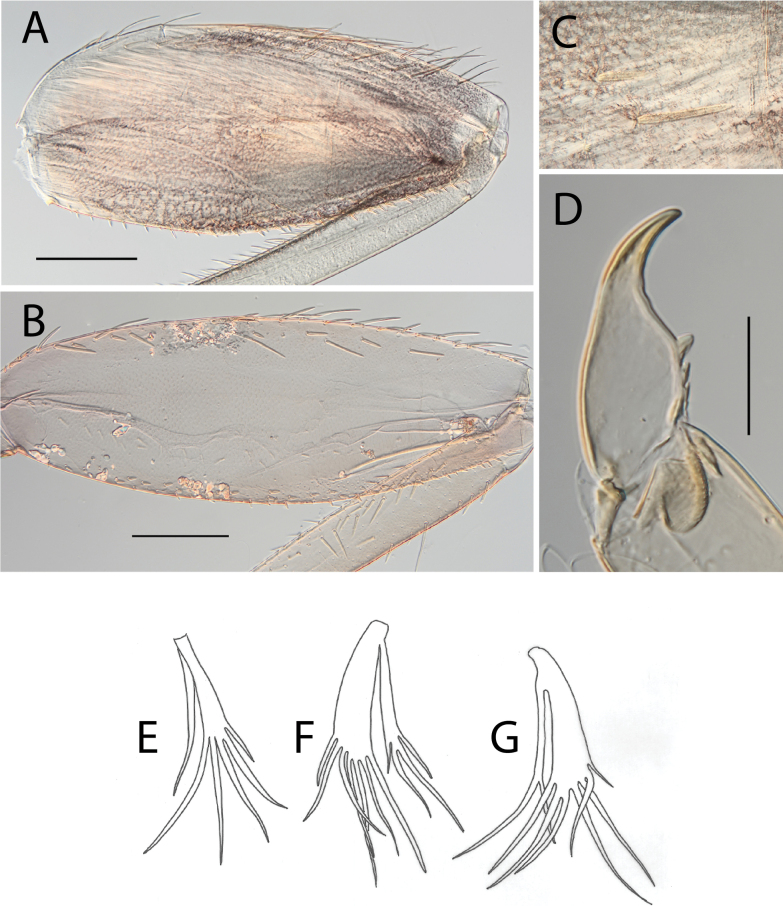
*Nonnullidenskaltenbachi* sp. nov. thorax and abdomen. **A.** Fore femur; **B.** Hind femur; **C.** Setae on dorsal surface of fore femur; **D.** Claw; **E.** Gill I; **F.** Gill IV; **G.** Gill VII. Scale bars: 200 µm (**A, B**); 50 µm (**C, D**).

***Thorax*.** Dorsal margin of femur with a row of long, pointed setae; irregular submarginal row of long, pointed setae as long as those on the dorsal margin; ventral margin with short, pointed setae and an irregular submarginal row of longer and blunt setae (Fig. 48A–B); central area of upper surface with very few long, pointed setae (Fig. 48C). Fore tibia with several rows of long simple or feathered setae on ventral margin, dorsal margin with few hair-like setae; middle tibia with one row of short, simple, pointed setae, and an irregular submarginal row of long, pointed setae on ventral margin, dorsal margin with numerous hair-like setae; hind tibia with a row of short simple setae on ventral margin and a submarginal row of long, simple, pointed setae, outer margin with long and short pointed setae and numerous hair-like setae. Tarsal claw hooked with three denticles progressively larger apically (Fig. 48D).

***Abdomen*.** Gill I with both lamellae. Gills II–VII with dorsal and ventral lamellae, ventral lamella smaller. Gill I with five filaments on dorsal lamella and a single filament on ventral lamella (Fig. 48E), gills II–VI with seven or eight filaments on dorsal lamella and three or four filaments on ventral lamella (Fig. 48F), gill VII with seven filaments on dorsal lamella and two filaments on ventral lamella (Fig. 48G). Posterior margin of terga IX with minute denticles. Posterior margin of terga V–VIII without denticles. Posterolateral projections present on segments VIII–IX.

##### Derivatio nominis.

This species is dedicated to Dr Thomas Kaltenbach (Naturéum, Lausanne) who revealed the megadiversity of the mayfly genus *Labiobaetis* in New Guinea.

#### ﻿ Nonnullidens marcelae sp. nov.

Taxon classificationAnimaliaEphemeropteraLeptophlebiidae

FF9A8430-E3A9-5ADC-8837-4C60CFF258A7

https://zoobank.org/2BAB3F0A-8774-47AD-AB4D-A7944381E091

Figs 49 , 50

##### Material examined.

***Holotype*.** • Indonesia, Papua, one nymph on slide, Sorong Island, 95 m, 19.II.2006, 00°49.354'S, 131°24.196'E, M. Balke & Tindige col [BH20], GBIFCH01223151 (MZB). ***Paratypes*.** • Indonesia, Papua, one nymph on slide, GBIFCH01223152, one nymph in ethanol, GBIFCH01523466, same data as holotype (MZL).

##### Nymph.

Body length, female: 3.5 mm.

##### Diagnosis.

Labrum emargination narrow without denticles; central area of upper surface of femora with very few short blunt setae; femora pale brown with two transverse dark brown bands in middle and at apex; posterior margin of abdominal segments IX with well-developed denticles; posterolateral expansions on the abdomen present on segments VIII–IX.

##### Colouration.

***Head*.** Vertex greyish brown, clypeus pale brown. Antenna broken. Mouth parts pale brown. ***Thorax*.** Pronotum pale brown, greyish brown on lateral margins, a greyish brown triangular macula in antero median position. Mesonotum pale brown, washed with greyish brown posteriorly (Fig. 50C). Femur yellowish, washed with pale brown in median area and at apex. Tibia uniformly yellowish. Tarsi yellowish. ***Abdomen*.** Terga uniformly greyish brown, posterior margin of segments IX and X pale brown. Sterna yellowish, sterna I–VII washed with greyish brown laterally. Gills greyish brown, filaments pale grey. Terminal filaments yellowish.

##### Description.

**Figure 49.**
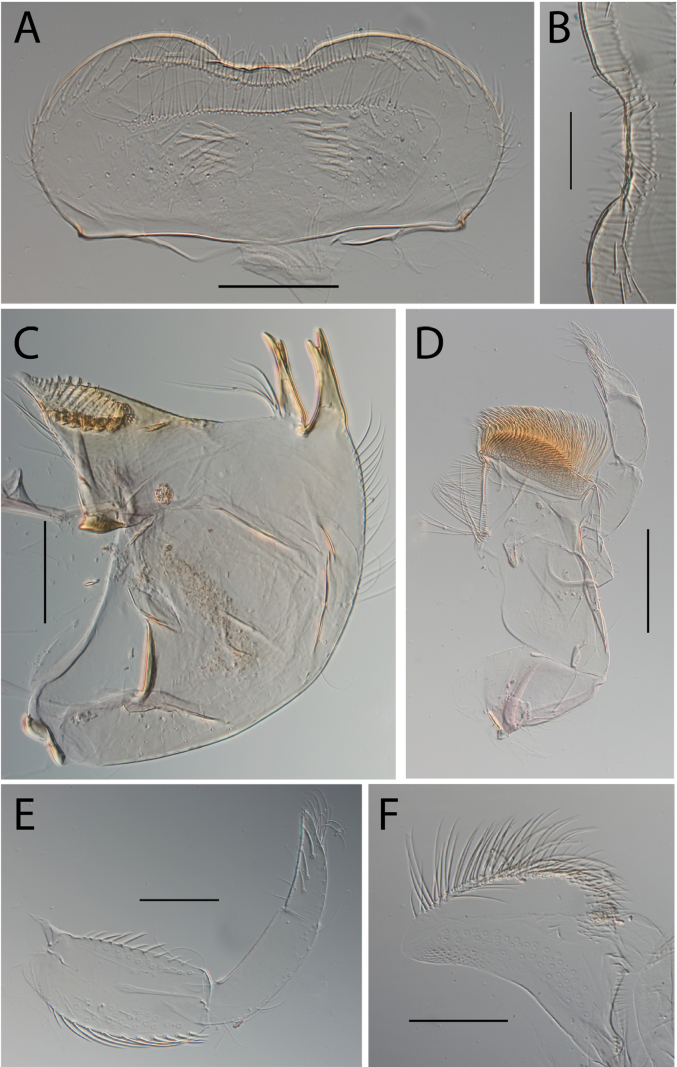
*Nonnullidensmarcelae* sp. nov. nymphal mouthparts. **A.** Labrum, dorsal view; **B.** Emargination of the labrum; **C.** Right mandible; **D.** Maxilla; **E.** Labium; **F.** Superlingua of hypopharynx. Scale bars: 100 µm (**A, C, E, F**); 50 µm (**B**); 200 µm (**D**).

***Head*. *Labrum*** rounded (Fig. 49A). Ratio labrum width/clypeus width 0.97–1.00. Ratio labrum width/insertion width 1.19–1.24. Medial emargination narrow without denticles. Proximal row of setae present, simple. Number of setae on proximal row ~ 51–75. Distal row of setae present, simple. Median part of labrum in ventral view with scattered thin setae (Fig. 49B). ***Mandibles*.** Outer margin with row of setae on distal 1/2. Ending of the row before incisor. Right mandible with 8–12 setae below the mola (Fig. 49C). ***Maxillae*.** Posterior margin of cardo with few hair-like setae, together with one stout and long seta in submarginal position. Proximo-lateral margin of stipes with a single stout and long seta. Apical-ventral row with 23–25 pectinate setae. Maxillary palp segment II 0.76–0.91 as long as segment I. Maxillary palp segment III 0.62–0.73 as long as segment I (Fig. 49D). Stout setae on outer margin of segment I absent. Segment II inner margin with three stout setae, outer margin without stout setae. Segment III inner margin with three or four stout setae, dorsal surface with few setae at apex, or half covered with setae. Segment III 1.78–2.05 × as long as base width. ***Labium*.** Labial palp segment II 0.75 as long as segment I. Labial palp segment III 0.56 as long as segment I (Fig. 49E). Segment I inner margin with ten stout setae, outer margin with 13 stout setae. Segment II outer margin without stout setae dorsal face with three stout setae. Segment III 2.71 × as long as base width. ***Hypopharynx*.** Apex of superlingua somewhat pointed (Fig. 49F).

**Figure 50.**
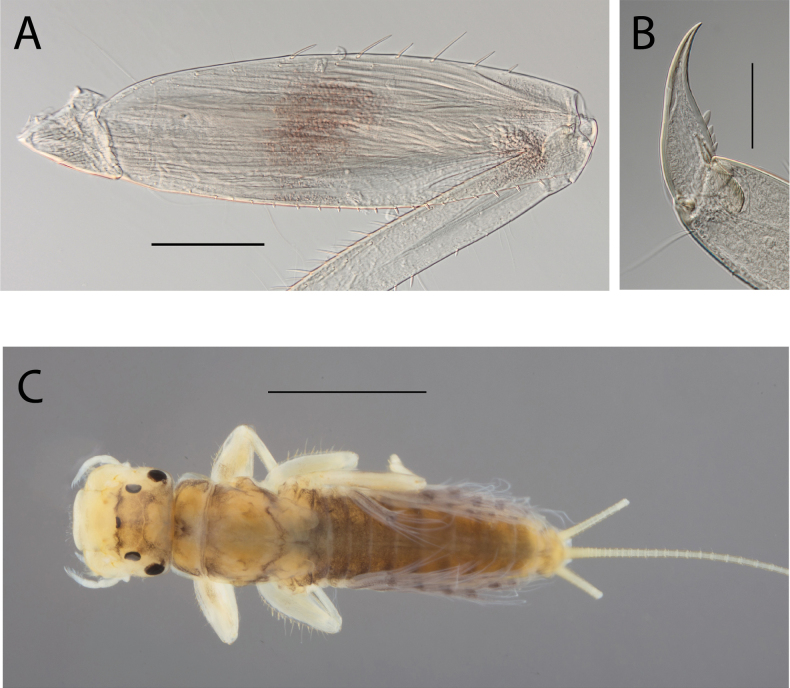
*Nonnullidensmarcelae* sp. nov. thorax. **A.** Hind femur; **B.** Claw; **C.** Habitus. Scale bars: 200 µm (**A**); 50 µm (**B**); 1 mm (**C**).

***Thorax*.** Dorsal margin of femur with a row of few (< 12) long, pointed setae; irregular submarginal row of blunt setae shorter than those on the dorsal margin; ventral margin with short, pointed setae and an irregular submarginal row of longer and blunt setae; central area of upper surface with very few short blunt setae (Fig. 50A). Fore tibia with several rows of long simple or feathered setae on ventral margin, dorsal margin with few hair-like setae; middle tibia with two rows of long, simple, pointed setae on ventral margin, dorsal margin with few hair-like setae; hind tibia with two rows of long and short feathered and simple setae on ventral margin, outer margin with short pointed setae and few hair-like setae. Tarsal claw straight with 2–4 denticles progressively larger apically (Fig. 50B).

***Abdomen*.** Gills II–VII with dorsal and ventral lamellae, ventral lamella smaller. Gill I broken; gill II–VI with six or seven filaments on dorsal lamella and four on ventral lamella; gill VII with five filaments on dorsal lamella and three or four on ventral lamella. Posterior margin of terga IX with denticles. Posterior margin of terga VIII without or with minute denticles. Posterior margin of terga V–VII without denticles. Posterolateral projections present on segments VIII–IX.

##### Derivatio nominis.

This species is dedicated to Dr Marcela Miranda de Lima (Viçosa, Brazil).

#### ﻿ Nonnullidens mariae

Taxon classificationAnimaliaEphemeropteraLeptophlebiidae

(Peters & Tsui, 1972)

5D86B460-12A5-5F69-A56F-8E182E52E81D

Fig. 51

Thraulus mariae Peters & Tsui, 1972 Orient. Insects 6(1): 3 (male, female, nymph).
Barba mariae ; Grant and Peters 1993 Trans. Am. Entomol. Soc. 119(2): 149 (new genus).
***nec***Nonnullidensmariae ; Kluge, 2013 Zootaxa 3722(1): 48 (new combination).

##### Material examined.

***Paratypes*.** • Papua New Guinea two nymphs in ethanol, GBIFCH01523467, one nymph on two slides GBIFCH01223153 (whole body), GBIFCH01223154 (gills), three female imagos in ethanol, GBIFCH01523468, Morobe Province, Bulolo River, east of Wau, 985 m, approx 7°20'16"S,146°43'42"E, 15–27.X.1964, W.L & J.G. Peters col (MZL).

##### Nymph.

Body length, male: 6.5–7 mm.

##### Diagnosis.

Medial emargination of labrum narrow with rounded denticles; ventral face of labrum with scattered thin setae; femora pale brown with two transverse dark brown bands in middle and at apex; posterior margin of abdominal segments V–VIII with well-developed denticles.

##### Colouration.

**Figure 51.**
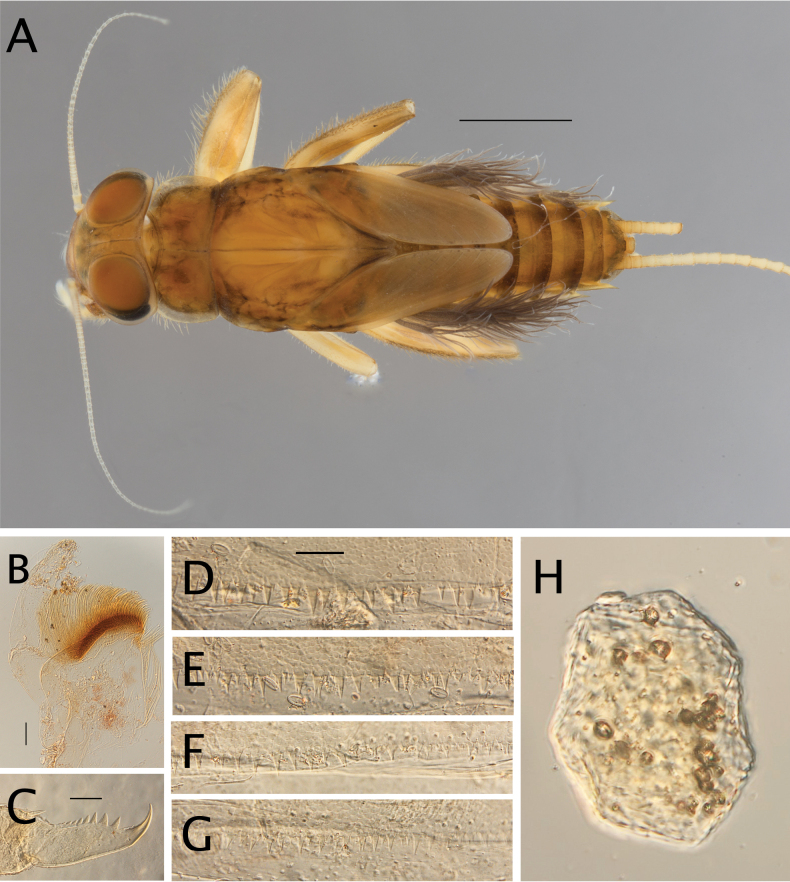
*Nonnullidensmariae* (Peters & Tsui, 1973). **A.** Habitus; **B.** Maxilla; **C.** Claw; **D.** Posterior margin of tergite IX; **E.** Posterior margin of tergite VIII; **F.** Posterior margin of tergite VII; **G.** Posterior margin of tergite VI; **H.** Egg. Scale bars: 1 mm (**A**); 100 µm (**B**); 50 µm (**C–G**).

***Head*.** Greyish brown, oval pale brown maculae in front of the median ocellus. Antenna yellowish, pedicel greyish brown. Compound eyes: lower portion black, upper portion orangish. Mouth parts yellowish brown. ***Thorax*.** Pronotum brownish orange, two triangular maculae on each side of the sagittal line (Fig. 51A). Mesonotum pale brown, washed with grey laterally. Femur greyish brown, apex pale brown, a pale brown band in the distal 1/3. Tibia yellowish, apex ringed with black. Tarsi yellowish. ***Abdomen*.** Terga medium brown, posterior margin of terga dark brown, as well as lateral sides. Sterna yellowish brown, posterior margin of sterna greyish brown laterally, nervous ganglia slightly tinted with greyish purple. Gills greyish purple. Terminal filaments pale brown, yellowish at apex.

##### Description.

***Head*. *Labrum*** cordiform. Ratio labrum width/clypeus width 1.13. Ratio labrum width/insertion width 1.35. Medial emargination narrow with rounded denticles. Proximal row of setae present, simple. Number of setae on proximal row ~ 95–100. Distal row of setae simple. Median part of labrum in ventral view with scattered thin setae. ***Mandibles*.** Outer margin with row of setae on entire margin. Ending of the row near incisor. Right mandible with 7–9 setae below the mola. ***Maxillae*.** Posterior margin of cardo with numerous thin and stout setae. Proximo-lateral margin of stipes twisted. Apical-ventral row with 25–27 pectinate setae. Maxillary palp segment II 0.88–0.93 as long as segment I. Maxillary palp segment III 0.68–0.79 as long as segment I (Fig. 51B). Stout setae on outer margin of segment I present. Segment II inner margin without stout setae, outer margin without stout setae. Segment III inner margin without stout setae, dorsal surface fully covered with setae. Segment III 1.67–1.80 × as long as base width. ***Labium*.** Labial palp segment II 0.80–0.81 as long as segment I. Labial palp segment III 0.62–0.63 as long as segment I. Segment I inner margin with 17–20 stout setae, outer margin with 14–16 stout setae. Segment II outer margin with 17–20 stout setae. Segment III dorsal face with four stout setae. Segment III 2.97–3.02 × as long as base width. ***Hypopharynx*.** Apex of superlingua truncate.

***Thorax*.** Dorsal margin of femur with a row of long, stout, pointed setae; irregular submarginal rows of shorter and pointed setae on fore femur, regular row of shorter, pointed setae on hind femur; ventral margin with short, stout setae and a submarginal row of long, pointed stout setae, except on hind leg where those of the submarginal row are short, pointed; central area of upper surface with long, pointed setae, shorter on hind femur. Fore tibia with several rows of long, simple setae on ventral margin, outer margin with few hair-like setae; middle tibia with two rows of long, simple pointed setae on ventral margin, outer margin with few hair-like setae; hind tibia with a row of simple long, thin setae on ventral margin, upper and lower surface covered with simple and feathered setae, outer margin with short and long pointed stout setae and hair-like setae. Tarsal claw hooked, with seven or eight denticles progressively larger apically (Fig. 51C).

***Abdomen*.** Gills I–VII with dorsal and ventral lamellae, ventral lamella smaller. Gill I with ~ eight filaments on upper lamella and five on lower lamella, gills II–V with ~ 8–10 filaments on each lamella, gill VI with 5–7 filaments on each lamella, gill VII with four to five filaments on each lamella. Posterior margin of terga V–IX with denticles (Fig. 51D–G). Posterolateral projections present on segments VII–IX.

***Eggs*.** General shape rounded. Size 140–145 μm × 85–90 μm (Fig. 51H). Chorionic surface smooth. No attachment structures visible. Micropyle not visible.

#### ﻿ Nonnullidens moiorum sp. nov.

Taxon classificationAnimaliaEphemeropteraLeptophlebiidae

897C3C11-0981-5174-8E24-6AB829BA0BA0

https://zoobank.org/351C4A31-0E6A-4EDB-811A-B774DB157B49

**Figure 52.**
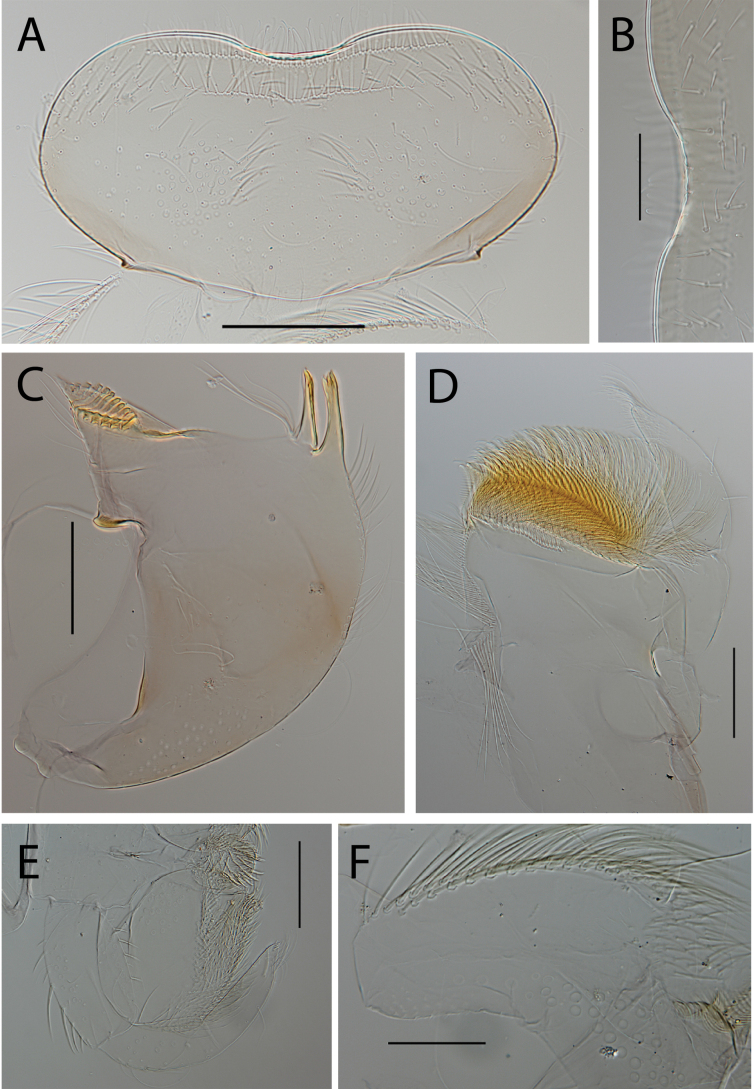
*Nonnullidensmoiorum* sp. nov. nymphal mouthparts. **A.** Labrum, dorsal view; **B.** Emargination of the labrum; **C.** Right mandible; **D.** Maxilla; **E.** Labium; **F.** Superlingua of hypopharynx. Scale bars: 100 µm (**A, C–E**); 50 µm (**B, F**).

**Figure 53.**
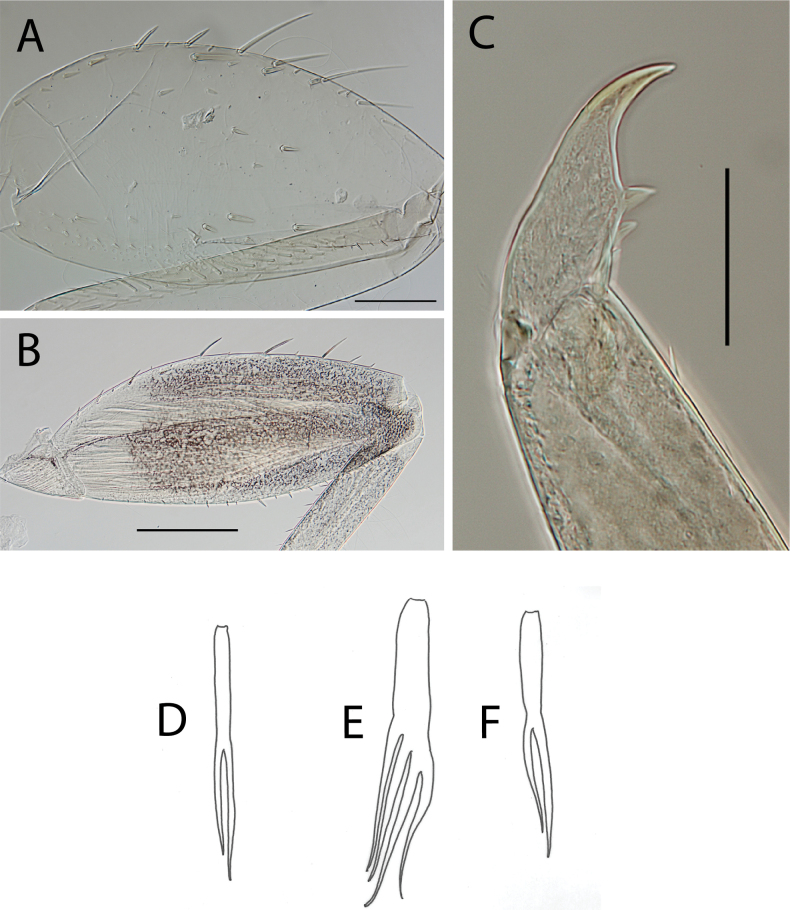
*Nonnullidensmoiorum* sp. nov. thorax and abdomen. **A.** Fore femur; **B.** Hind femur; **C.** Claw; **D.** Gill I; **E.** Gill IV; **F.** Gill VII. Scale bars: 100 µm (**A**); 200 µm (**B**); 50 µm (**C**).

Figs 34I , 36C , 52 , 53

##### Material examined.

***Holotype*.** • Indonesia, Papua, one nymph on slide, Sorong Island, 95 m, 19.II.2006, 00°49.354'S, 131°24.196'E, M. Balke & Tindige col [BH20], GBIFCH01223147 (MZB). ***Paratypes*.** • Indonesia, Papua, one nymph on slide, GBIFCH01223148, one nymph in ethanol, GBIFCH01523469 same data as holotype (MZL).

##### Nymph.

Body length, female: 4.5 mm.

##### Diagnosis.

Femora uniformly dark brown, without transverse bands; tarsal claw with only three teeth; all gills uni-lamellate.

##### Colouration.

***Head*.** Vertex greyish brown, lighter between ocelli, frons pale brown washed with grey. Antenna yellowish, scape greyish. Mouth parts greyish brown. ***Thorax*.** Pronotum medium brown, washed with dark brown laterally, medially, and anteromedially. Mesonotum medium brown, dark brown laterally, with two blackish lines originating from the sides and reaching the posterior margin (Fig. 36C). Femur greyish brown, lighter at base. Tibia yellowish, fore tibia washed with grey apically. Tarsi yellowish. ***Abdomen*.** Terga medium brown, a lighter sagittal line increasing in width posteriorly. Sterna yellowish, segments I–VII washed with greyish brown laterally. Gills greyish brown. Terminal filaments yellowish.

##### Description.

***Head*. *Labrum*** cordiform (Fig. 52A). Ratio labrum width/clypeus width 1.02. Ratio labrum width/insertion width 1.45. Medial emargination shallow without denticles. Proximal row of setae present, simple. Number of setae on proximal row ~ 30–40. Distal row of setae present, simple. Median part of labrum in ventral view with scattered thin setae (Fig. 52B). ***Mandibles*.** Outer margin with row of setae on distal 1/2. Ending of the row before incisor. Right mandible with six setae below the mola (Fig. 52C). ***Maxillae*.** Posterior margin of cardo broken. Proximo-lateral margin of stipes with one medium size and two short stout setae (Fig. 34I). Apical-ventral row with 20–25 pectinate setae. Maxillary palp segment II 0.76–1.0 as long as segment I. Maxillary palp segment III 0.76–0.9 as long as segment I (Fig. 52D). Stout setae on outer margin of segment I absent. Segment II inner margin with two or three stout setae. Segment II outer margin with two stout setae. Segment III inner margin with three or four stout setae, dorsal surface with few setae at apex. Segment III 1.86–2.07 × as long as base width. ***Labium*.** Labial palp segment II 0.73–0.75 as long as segment I. Labial palp segment III 0.67–0.80 as long as segment I (Fig. 52E). Segment I inner margin with 7–9 stout setae, outer margin with 6–9 stout setae. Segment II, outer margin with 2–4 stout setae. Segment III, dorsal face with two or three stout setae. Segment III 2.88–3.33 × as long as base width. ***Hypopharynx*.** Apex of superlingua truncate (Fig. 52F).

***Thorax*.** Dorsal margin of femur with a row of few (< 12) long and pointed setae; irregular submarginal row of blunt, pointed setae shorter or as long as those on the dorsal margin; ventral margin with short, pointed setae and an irregular submarginal row of longer, blunt setae; central area of upper surface with very few short pointed and blunt setae (Fig. 53A–B). Fore tibia with several rows of long simple or feathered setae on ventral margin, dorsal margin with numerous hair-like setae; middle tibia with one row of short, simple, pointed setae, and an irregular submarginal row of short, pointed setae on ventral margin, dorsal margin with numerous hair-like setae; hind tibia with two rows of long and short feathered and simple setae on ventral margin, dorsal margin with long pointed setae and numerous hair-like setae. Tarsal claw slightly hooked with three denticles progressively larger apically (Fig. 53C).

***Abdomen*.** All gills uni-lamellate, ventral lamella lacking; gill I with two filaments (Fig. 53D), gills II–VI with four or five filaments (Fig. 53E), gill VII with two filaments (Fig. 53F). Posterior margin of terga IX with minute denticles. Posterior margin of terga V–VIII without denticles. Posterolateral projections present on segments VIII–IX.

##### Derivatio nominis.

The species name is to recognise the Moi people, a native tribe of Sorong, who fight to keep their intact environment and their way of living.

#### ﻿ Nonnullidens silvaepumilorum sp. nov.

Taxon classificationAnimaliaEphemeropteraLeptophlebiidae

6C62AFD7-45D8-5534-A43C-F1E34726C655

https://zoobank.org/6AD6773C-6F26-4D4D-ABB9-EA2C77194FD4

Figs 54 , 55

##### Material examined.

***Holotype*.** • Papua New Guinea, one nymph on slide, Madang Province, Aiome area 130 m, 11.III.2007, 05°10.593'S, 144°42.800'E, Kinibel col [PNG156], GBIFCH01223146 (ZSM). ***Paratype*.** • Papua New Guinea, one nymph in ethanol, GBIFCH01523470, same data as holotype (MZL).

##### Nymph.

Body length, female: 5 mm.

##### Diagnosis.

Median emargination of labrum shallow with flat denticles; ventral surface of labrum with stout setae in row; femora pale brown with two transverse dark brown bands in middle and at apex; posterolateral expansions present on segments VII–IX.

##### Colouration.

***Head*.** Medium brown, washed with greyish brown between ocelli, pale brown between lateral ocelli and compound eyes, occiput with pale brown vermiform maculae around the sagittal line, clypeus dark brown. Antenna broken. Mouth parts pale brown. ***Thorax*.** Pronotum pale brown, dark brown anteriorly and laterally, as well as along the sagittal line. Mesonotum pale brown, washed with dark brown laterally and along the distal half of the sagittal line, fore wing pads medium brown, with a dark brown spot at the base. Femur pale brown, with a large medium brown band in the middle and at apex. Tibia yellowish, with a dark brown ring at apex. Tarsi yellowish to pale brown. ***Abdomen*.** Terga segments I and II entirely dark brown, segments III and IV medium brown with a dark brown macula in anteromedian position, this macula with a light brown longitudinal line on segments V–VII, segments VII–X dark brown. Sterna yellowish grey, segments III–VI with a dark brown macula in anteromedian position. Gills pale grey, tinted with purple. Terminal filaments broken.

##### Description.

**Figure 54.**
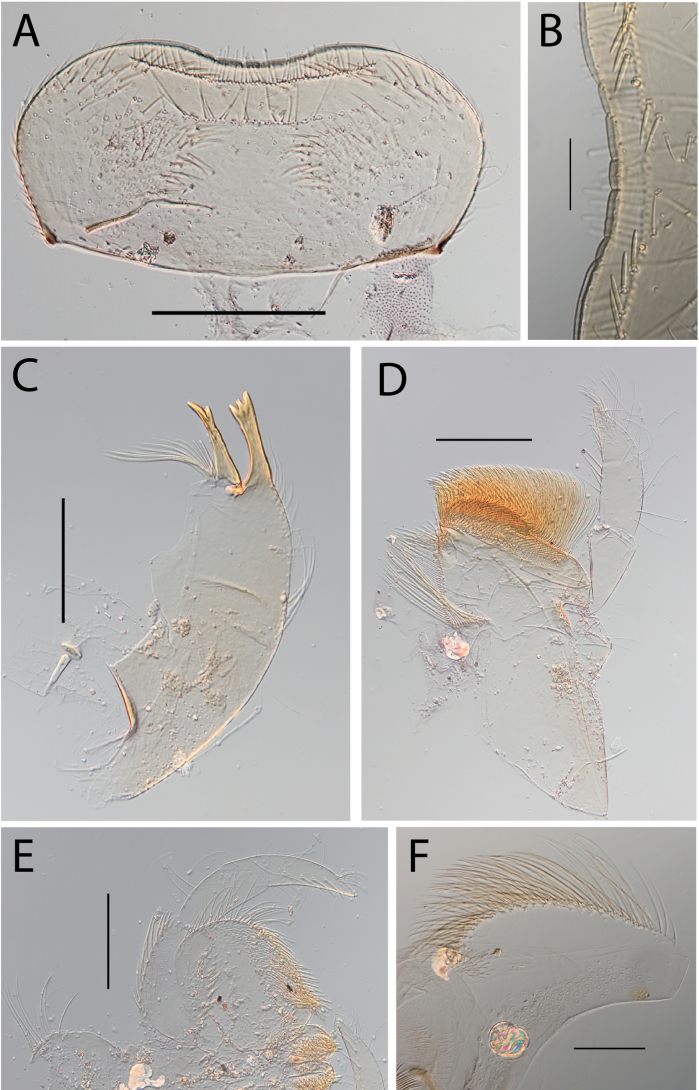
*Nonnullidenssilvaepumilorum* sp. nov. nymphal mouthparts. **A.** Labrum, dorsal view; **B.** Emargination of the labrum; **C.** Right mandible; **D.** Maxilla; **E.** Labium; **F.** Superlingua of hypopharynx. Scale bars: 100 µm (**A, F**); 50 µm (**B**); 200 µm (**C–E**).

***Head*. *Labrum*** cordiform (Fig. 54A). Ratio labrum width/clypeus width 0.95. Medial emargination shallow with flat denticles. Proximal row of setae present, simple. Number of setae on proximal row ~ 22. Distal row of setae present, simple. Median part of labrum in ventral view with stout setae in row (Fig. 54B). ***Mandibles*.** Outer margin with row of setae on distal 1/2. Ending of the row near incisor. Right mandible with eight setae below the mola (Fig. 54C). ***Maxillae*.** Posterior margin of cardo broken. Proximo-lateral margin of stipes with one long and stout seta. Apical-ventral row with 16 pectinate setae. Maxillary palp segment II 0.84 as long as segment I. Maxillary palp segment III 0.67 as long as segment I (Fig. 54D). Stout setae on outer margin of segment I absent. Segment II inner margin with four stout setae, outer margin with four stout setae. Segment III inner margin with four stout setae, dorsal surface with few setae at apex. Segment III 1.64 × as long as base width. ***Labium*.** Labial palp segment II 0.94 as long as segment I. Labial palp segment III 0.82 as long as segment I (Fig. 54E). Segment I inner margin with 14 stout setae, outer margin with 13 stout setae. Segment II outer margin with seven stout setae. Segment III dorsal face with six stout setae. Segment III 3.11 × as long as base width. ***Hypopharynx*.** Apex of superlingua truncate (Fig. 54F).

**Figure 55.**
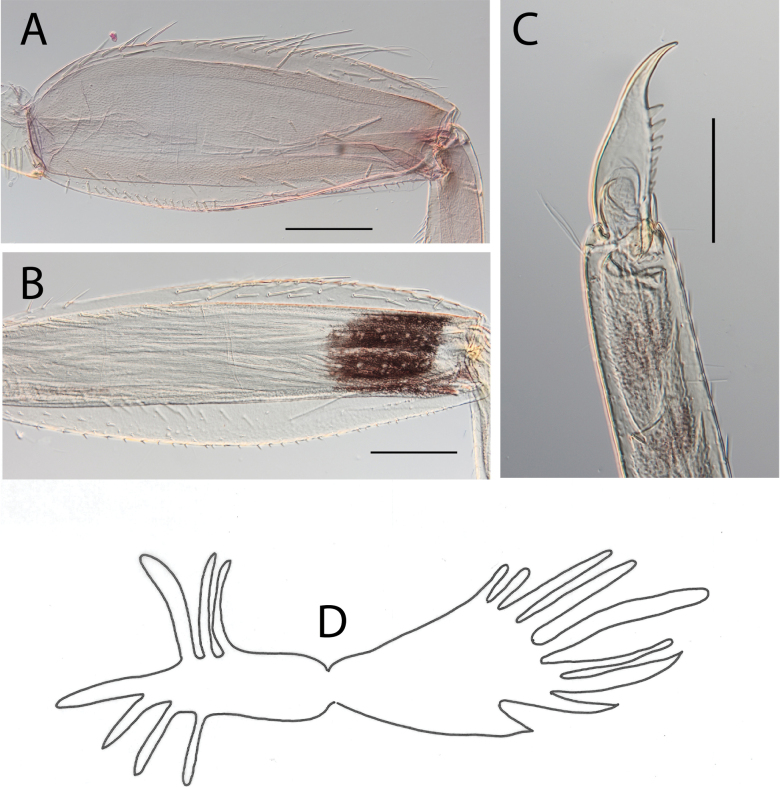
*Nonnullidenssilvaepumilorum* sp. nov. thorax and abdomen. **A.** Fore femur; **B.** Hind femur; **C.** Claw; **D.** Gill IV. Scale bars: 200 µm (**A, B**); 100 µm (**C**).

***Thorax*.** Dorsal margin of fore femur with long, stout, pointed setae, and a submarginal row of long, stout, pointed setae; dorsal margin of mid- and hind femur with a row of long, stout, pointed setae; a submarginal row of pointed setae as long as or a little bit shorter than those on the dorsal margin; ventral margin with short, stout setae and an irregular submarginal row of long, pointed stout setae, except on hind leg where those on ventral margin are shorter; central area of upper surface with few short or long stout and pointed setae (Fig. 55A–B). Fore tibia with two or three rows of long simple or feathered setae on ventral margin, dorsal margin with numerous hair-like setae; middle tibia with two rows of long, simple, pointed setae on ventral margin, outer margin with numerous hair-like setae; hind tibia with two rows of simple and feathered long, stout setae on ventral margin, outer margin with a row of long and short simple stout setae, with numerous hair-like setae. Tarsal claw hooked with 4–6 denticles progressively larger apically (Fig. 55C).

***Abdomen*.** Gill I with both lamellae. Gills II–VII with dorsal and ventral lamellae of the same size. Gills very small, gill I with five or six filaments on each lamella, gills II–VI with 7–10 filaments on each lamella (Fig. 55D). Posterior margin of terga IX with denticles. Posterior margin of terga VIII without or with minute denticles. Posterior margin of terga V–VII without denticles. Posterolateral projections present on segments VII–IX.

##### Derivatio nominis.

The species name is a Latin word composed of silva (forest) and pumilo (dwarf) to recognise the Melanesian Dwarf tribe of Aiome, and the land where the specimens of this new species come from.

#### ﻿ Nonnullidens

Taxon classificationAnimaliaEphemeropteraLeptophlebiidae

sp. A

B34BFFD0-095A-5567-AE1E-E16808A35CD1

Figs 34H , 56 , 57

##### Material examined.

• Indonesia, Papua, one nymph on slide, GBIFCH01223145, Road Nabire–Enarotali KM 55, 774 m, 22.X.2011, 03°29.796'S, 135°43.885'E, M. Balke col [PAP9] (MZL).

##### Diagnosis.

**Nymph.** Labrum trapezoidal; dorsal face of labrum with simple row in distal position; femora pale brown with two transverse dark brown bands in middle and at apex.

##### Colouration.

Femur yellowish brown, with a large median greyish brown stripe and another one at apex (Fig. 57A–B). Tibia yellowish. Tarsi yellowish.

##### Description.

**Figure 56.**
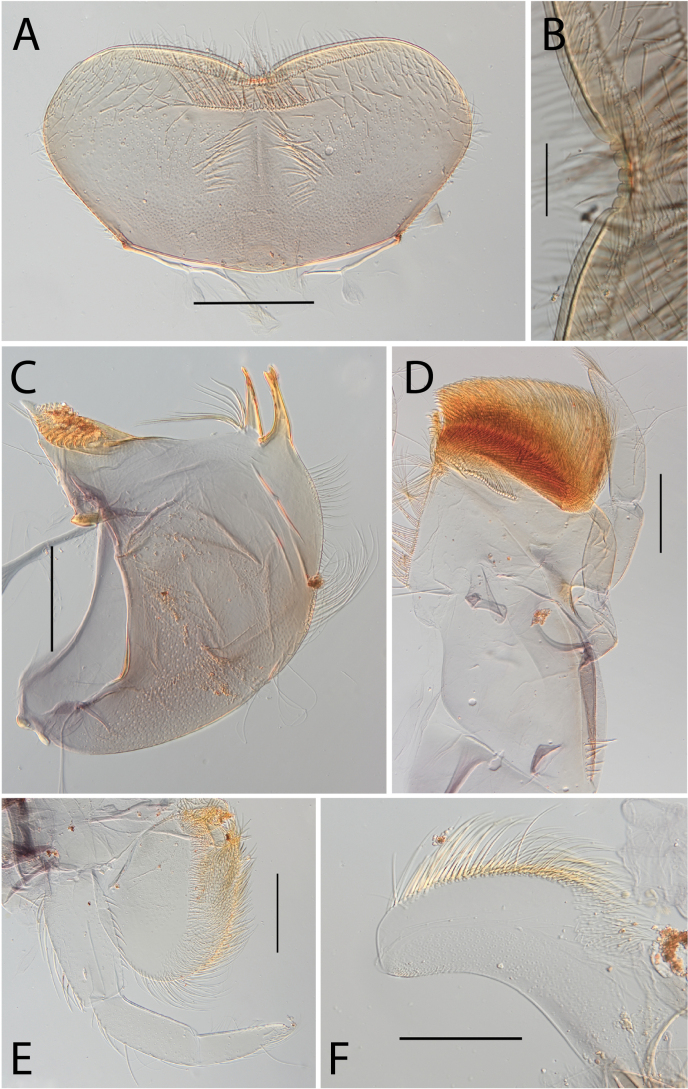
*Nonnullidens* sp. A. Nymphal mouthparts. **A.** Labrum, dorsal view; **B.** Emargination of the labrum; **C.** Right mandible; **D.** Maxilla; **E.** Labium; **F.** Superlingua of hypopharynx. Scale bars: 200 µm (**A, C–E**); 50 µm (**B**); 100 µm (**F**).

***Head*. *Labrum*** trapezoid (Fig. 56A). Ratio labrum width/clypeus width 1.11. Ratio labrum width/insertion width 1.56. Medial emargination narrow with flat denticles. Proximal row of setae present, simple. Number of setae on proximal row ~ 46. Distal row of setae present, simple. Median part of labrum in ventral view with scattered thin setae (Fig. 56B). ***Mandibles*.** Outer margin with row of setae on distal 1/2. Ending of the row before incisor. Right mandible with seven setae below the mola (Fig. 56C). ***Maxillae*.** Posterior margin of cardo with few hair-like setae, together with one stout and long seta in submarginal position. Proximo-lateral margin of stipes with a bunch of ~ six stout setae increasing in size distally (Fig. 34H). Apical-ventral row with 32 pectinate setae. Maxillary palp segment II 0.73–0.78 as long as segment I. Maxillary palp segment III 0.79–0.81 as long as segment I (Fig. 56D). Stout setae on outer margin of segment I absent. Segment II inner margin with four stout setae, outer margin with four stout setae. Segment III inner margin with six stout setae, dorsal surface with few setae at apex. Segment III 2.36 × as long as base width. ***Labium*.** Labial palp segment II 0.79 as long as segment I. Labial palp segment III 0.67 as long as segment I (Fig. 56E). Segment I inner margin with nine stout setae, outer margin with ten stout setae. Segment II outer margin with five stout setae. Segment III dorsal face with three stout setae. Segment III 3.00 × as long as base width. ***Hypopharynx*.** Apex of superlingua truncate (Fig. 56F).

**Figure 57.**
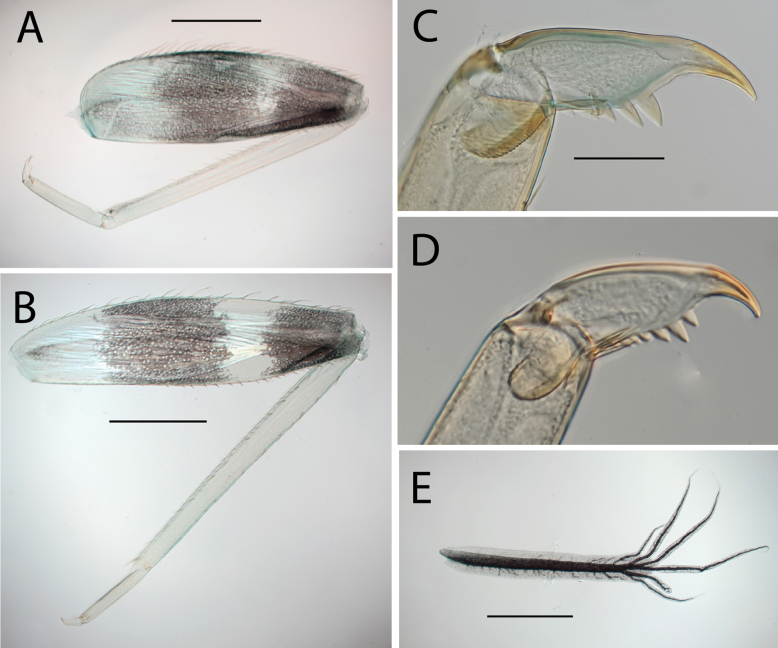
*Nonnullidens* sp. A. Thorax and abdomen. **A.** Fore leg; **B.** Hind leg; **C.** Fore claw; **D.** Hind claw; **E.** Gill IV. Scale bars: 500 µm (**A, B, E**); 100 µm (**C, D**).

***Thorax*.** All legs much longer than in any other species of the *Thraulus* lineage; fore femur 3 × longer than wide (Fig. 57A), hind femur 4 × longer than wide (Fig. 57B). Dorsal margin of femur with a row of long, pointed setae; regular submarginal row of blunt setae shorter than those on the dorsal margin; ventral margin with short, pointed setae and an irregular submarginal row of longer, pointed setae; central area of upper surface with few long and short blunt setae. Fore tibia with several rows of long simple or feathered setae on ventral margin, dorsal margin with few hair-like setae; middle tibia with two rows of short, simple, pointed setae on ventral margin, dorsal margin with numerous hair-like setae; hind tibia with two or three rows of long and short feathered and simple setae on ventral margin, outer margin with short, pointed setae and numerous hair-like setae. Tarsal claw hooked with four or five denticles progressively larger apically (Fig. 57C–D).

***Abdomen*.** Most gills missing. Gill IV very long, uni-lamellate, with 5–6 filaments (Fig. 57E). Filamentous projections on apex. Posterior margin of terga IX with minute denticles. Posterior margin of terga V–VIII without denticles. Posterolateral projections present on segments VIII–IX.

#### ﻿ Nonnullidens

Taxon classificationAnimaliaEphemeropteraLeptophlebiidae

sp. B

FA1D57D1-3190-5024-BA8D-062294E6395A

Figs 58 , 59

##### Material examined.

• Papua New Guinea, one nymph on slide, GBIFCH01223128, Western Highlands Province, Simbai, 1800–2000 m, 25.II.2007, 05°16.330'S, 144°33.176'E, Kinibel col [PNG133] (MZL).

##### Diagnosis.

**Nymph.** Labrum trapezoidal; dorsal face of labrum with multiple rows in distal position; femora pale brown with two transverse dark brown bands in middle and at apex.

##### Colouration.

Femur yellowish, a medium brown transversal band in the middle, apex medium brown (Fig. 59A–B). Tibia yellowish, apex dark brown. Tarsi yellowish. Gills greyish brown.

##### Description.

**Figure 58.**
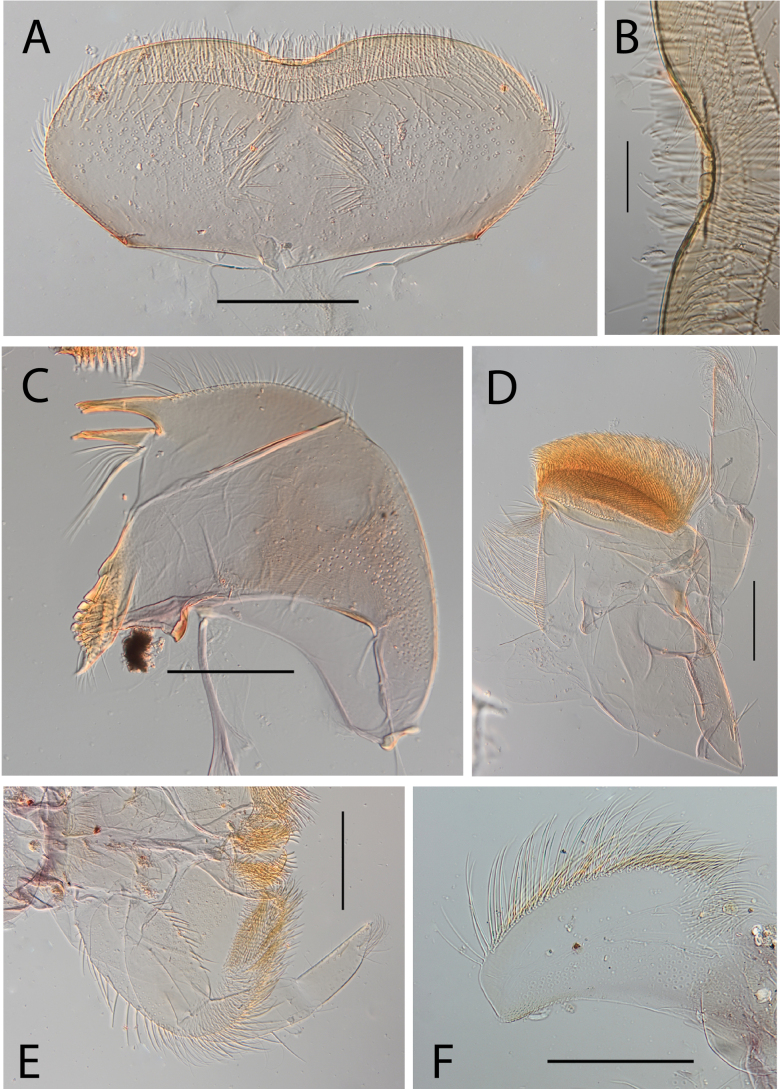
*Nonnullidens* sp. B. Nymphal mouthparts. **A.** Labrum, dorsal view; **B.** Emargination of the labrum; **C.** Right mandible; **D.** Maxilla; **E.** Labium; **F.** Superlingua of hypopharynx. Scale bars: 200 µm (**A, C–E**); 50 µm (**B**); 100 µm (**F**).

***Head*. *Labrum*** trapezoid (Fig. 58A). Ratio labrum width/clypeus width 1.17. Ratio labrum width/insertion width 1.46. Medial emargination narrow with flat denticles. Proximal row of setae present, simple. Number of setae on proximal row ~ 106. Distal row of setae multiple. Median part of labrum in ventral view with scattered thin setae (Fig. 58B). ***Mandibles*.** Outer margin with row of setae on distal 1/2. Ending of the row near incisor. Right mandible with nine setae below the mola (Fig. 58C). ***Maxillae*.** Posterior margin of cardo broken. Proximo-lateral margin of stipes with a bunch of ~ ten stout short and medium size setae. Apical-ventral row with 30 pectinate setae. Maxillary palp segment II 0.76 as long as segment I. Maxillary palp segment III 0.71 as long as segment I (Fig. 58D). Stout setae on outer margin of segment I absent. Segment II inner margin with two stout setae, outer margin without stout setae. Segment III inner margin with one stout seta, dorsal surface half covered with setae. Segment III 1.50 × as long as base width. ***Labium*.** Labial palp segment II 0.79 as long as segment I. Labial palp segment III 0.65 as long as segment I (Fig. 58E). Segment I inner margin with 14 stout setae, outer margin with 15 stout setae. Segment II outer margin with nine stout setae. Segment III dorsal face with two stout setae. Segment III 2.73 × as long as base width. ***Hypopharynx*.** Apex of superlingua truncate (Fig. 58F).

**Figure 59.**
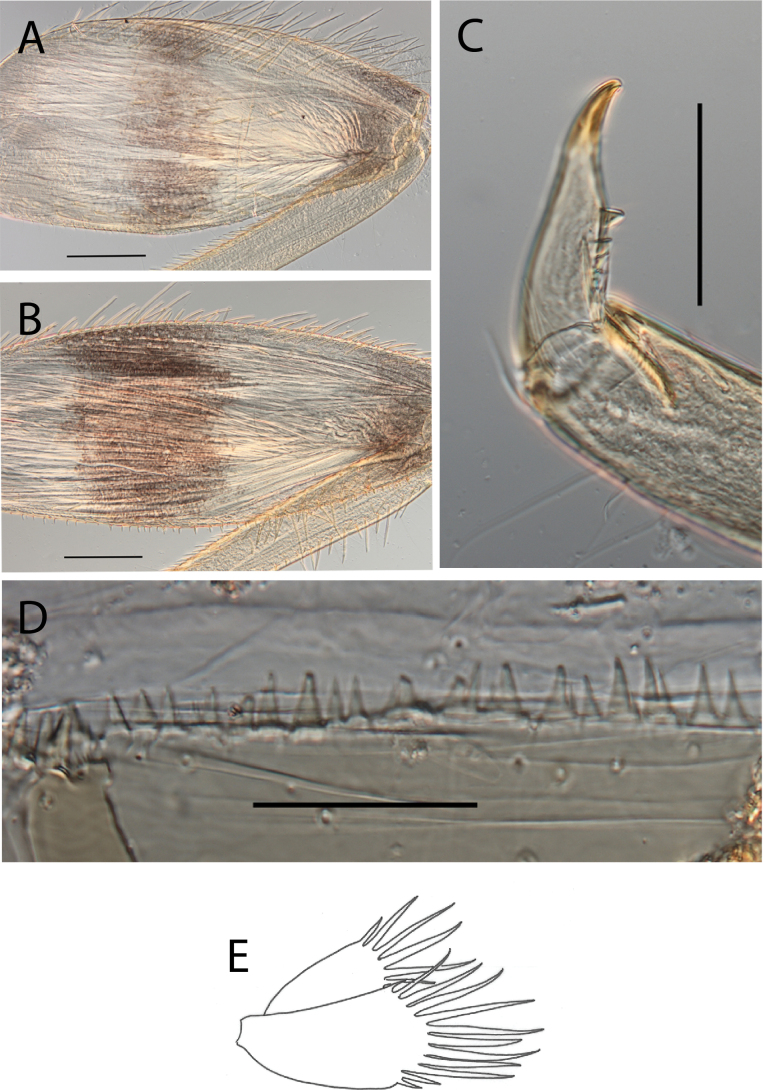
*Nonnullidens* sp. B. Thorax and abdomen. **A.** Fore femur; **B.** Hind femur; **C.** Claw; **D.** Posterior margin of tergite IX; **E.** Gill IV. Scale bars: 200 µm (**A, B**); 100 µm (**C**); 50 µm (**D**).

***Thorax*.** Dorsal margin of femur with a row of very long, thin, pointed setae; regular submarginal row of pointed setae as long as or shorter than those on the dorsal margin, multiple submarginal rows on fore femur (Fig. 59A); ventral margin with short, pointed setae and an irregular submarginal row of long, pointed setae, except on hind femur where they are short and stout; central area of upper surface with very long pointed setae on fore femur, long and short on middle femur and short on hind femur (Fig. 59B). Fore tibia with two or three rows of long simple or feathered setae on ventral margin, dorsal margin with numerous hair-like setae; middle tibia with a row of short, simple, pointed setae on ventral margin and a submarginal row of long, simple setae, outer margin with numerous hair-like setae; hind tibia with a marginal row of simple short, thin setae and a submarginal row of long to very long simple setae on ventral margin, outer margin with long to very long pointed setae and numerous hair-like setae. Tarsal claw slightly hooked, with five or six denticles progressively larger, except distal much larger (Fig. 59C).

***Abdomen*.** Gill I with both lamellae. Gills II–VII with dorsal and ventral lamellae, ventral lamella smaller. Gill I with nine or ten filaments on dorsal lamella and five or six on ventral lamella, gills II–VI with 11–12 filaments on dorsal lamella and six or seven on ventral lamella (Fig. 59E), gill VII with nine or ten filaments on dorsal lamella and four or five on ventral lamella. Posterior margin of terga IX with denticles (Fig. 59D). Posterior margin of terga V–VIII without denticles. Posterolateral projections present on segments VIII–IX.

### ﻿Differential diagnoses among *Nonnullidens* species

Characters allowing to recognise all species of the genus *Nonnullidens* are presented in the key. As already pointed by Kluge (2013), the colouration of the femora allows to separate the species in two groups: those with dorsal surface entirely dark brown, and those with dorsal surface pale brown with two transverse stripes in the middle and at apex. Three species exhibit a labrum trapezoidal instead of cordiform: *N.hsui*, *N.* sp. A, and *N.* sp. B. They can be separated by the number of pectinate setae on the ventral surface of maxillae and by the composition of the distal row on dorsal surface of labrum. The last two species are described in detail but not named due to the scarcity of the material in hand. Generally, *Nonnullidens* species are not complicated to separate based on gill composition, colouration of the femora, and posterolateral projections of the abdomen. Two couples of species present anyway some difficulties to be identified. First, *N.reductus* and *N.fuyugensis* which differ by several small characters, such as the number of setae on the dorsal surface of segment III of labial palp, shape of setae on ventral margin of femora or shape of claws. *Nonnullidensalvarezi* and *N.boonsoongi* secondly, which differ by small differences in femur setae, gill composition and ornamentation of maxillary palp. *Nonnullidensreductus* has been collected in Indonesia Papua at an altitude of less than 250 m, whereas *N.fuyugensis* comes from the Central Province of Papua New Guinea, at an altitude above 1500 m. Although *N.alvarezi* and *N.boonsoongi* inhabit high altitude streams, *N.boonsoongi* is found in Central Province and *N.alvarezi* in Eastern and Gulf provinces of Papua New Guinea.

### ﻿Status of *Nonnullidensmariae*

*Nonnullidensmariae* occupies a special position due to several morphological peculiarities. The nymph has a claw with a number of teeth similar to those of *Kosminympha*; ventral lamella of gills is not so reduced as in the other species of *Nonnullidens* (with the exception of *N.silvaepumilorum*), and the posterolateral projections on the abdomen are present on segments VII–IX, a character which is shared only with *N.cozzarolae* and *N.silvaepumilorum*. Moreover, *N.mariae* is the only species studied here which present well-developed posterior denticles on abdominal tergites V–IX. Finally, eggs of *N.mariae* are quite similar to those of other *Nonnullidens* species, and well different from those of *Thraulus* and *Kosminympha*.

Thus, it is unclear if *N.mariae* represents an atypical member of the genus *Nonnullidens*, or if the species, originally the type species of the genus *Barba* Grant & Peters, 1993, could stand in its own genus; in this case, the genus *Barba* should be removed from synonymy with *Nonnullidens* and revalidated. Based on our cladistic reconstruction (not shown) and the morphology of the eggs, we keep the species *mariae* in the genus *Nonnullidens*, pending the discovery of new evidence.

In any case, we can state that the specimens of *N.mariae*Kluge (2013) collected in Indonesia Papua (Baliem Valley) and which were used to synonymise the genera *Barba* and *Nonnullidens*, are not conspecific with *Barbamariae* sensu Grant & Peters, 1993 (Papua New Guinea, Morobe Province, Bulolo River). Kluge (2013) stated in the revised generic diagnosis of *Nonnullidens*, that posterolateral projections are present on abdominal segments VIII–IX, and that denticles on the posterior margin of abdominal tergite VIII are minute or absent. We can then suppose that the specimens he attributed to “*Nonnullidensmariae*” do possess these characters, which do not match the true *Barbamariae*. The morphology of these specimens is unfortunately too briefly mentioned for allowing any further specific attribution.

#### ﻿ Kosminympha

Taxon classificationAnimaliaEphemeropteraLeptophlebiidae

Sartori & Salles gen. nov.

2A3E6188-7BB8-5BBE-B47E-6423FE6BC107

https://zoobank.org/BDA7E779-B5E6-4C56-BD64-E34A253A49BD

##### Diagnosis.

Gill I similar in shape to the following ones. Ventral lamella of gills smaller but not highly reduced, with ≥ 9 processes; claws with 6–10 teeth; posterior margin of abdominal segment VIII with small denticles; posterolateral expansions on abdominal segments VII–IX, VI–IX or even V–IX. Eggs fusiform with hook-like attachment structures at each pole.

##### Derivatio nominis.

From the greek κόσμημα meaning jewel and νύμφη meaning nymph, to recognise the peculiar beauty of the species in this genus. The gender is feminine.

##### Type species.

*Kosminymphasabrinae* sp. nov. by present designation.

##### Species included.

*Kosminymphaasarorum* sp. nov., *K.balkei* sp. nov., *K.baruya* sp. nov., *K.kalamorum* sp. nov., *K.paulinae* sp. nov., *K.sabrinae* sp. nov.

##### Description.

Size: 10.0–11.0 mm for male mature nymphs; 10.5–12.5 mm for mature female nymphs.

***Head*.** Prognathous; labrum cordiform or trapezoid, anterior emargination either shallow with flat denticles or narrow with pointed denticles; proximal row of setae long, with 80–140 setae; distal row of setae multiple; median part of labrum in ventral view with scattered thin and long setae or with stout and short setae in a row. Outer margin of mandibles with a row of setae. Proximo-lateral margin of stipes with a bunch of setae, either long and thin or short and stout. Apex of superlingua of hypopharynx either truncate or emarginate. ***Thorax*.** Central area of upper face of femora with numerous short or long, pointed, or blunt setae; tarsal claw hooked, with 6–10 teeth. ***Abdomen*.** Gill I similar in shape to the following ones; gill I composed of dorsal and ventral lamellae, each with 9–15 filaments; gills II–VI composed of dorsal and ventral lamellae, each with 15–25 filaments; gill VII composed of dorsal and ventral lamellae, each with 5–15 filaments, depending on species. Posterior margin of abdominal terga II–VIII without or with minute denticles; posterolateral expansions of the abdomen present on segments V–IX, VI–IX or VII–IX. ***Egg.*** Elongated, with attachments structures fixed at each pole, composed of a long branch ending by a double fishhook.

##### Affinities.

Among mayfly nymphs of the subfamily Choroterpinae and of the tribe Thraulini, the genus *Kosminympha* is most similar to *Nonnullidens* by its gill composition. It differs anyway by the less reduced gill ventral lamella, bearing ≥ nine filaments whereas in *Nonnullidens*, ventral lamella bears at most eight filaments (in *N.mariae*, it can also reach ten filaments). Posterolateral projections of the abdomen are present on segments V–IX, VI–IX or VII–IX, whereas they are present on segments VIII–IX in *Nonnullidens* (VII–IX in some species). Posterior margin of tergite VIII always bears small denticles, whereas they are absent in *Nonnullidens* (present and well-developed in *N.mariae*). Finally, the eggs of *Kosminympha* are similar in shape to those of the genus *Thraulus*, being elongated and bearing attachment structures at each pole, whereas they are rounded and without attachment structure in *Nonnullidens*.

##### Distribution.

The genus *Kosminympha* is only known from Papua New Guinea and has been found on the eastern part of the island; the genus has yet not been recorded from Indonesia Papua.

#### ﻿ Kosminympha asarorum sp. nov.

Taxon classificationAnimaliaEphemeropteraLeptophlebiidae

8834D75A-AF72-52FC-B4F9-ECA135D771BC

https://zoobank.org/2DE8617F-9B82-4724-A1AB-3B1880BA5DFB

Figs 60C , 62 , 63

##### Material examined.

***Holotype*.** • Papua New Guinea, one nymph on slide, Eastern Highlands Province, Akameku – Brahmin, Bismarck Range, 2200 m, 23.XI.2006, 05°56.801'S, 145°22.238'E, M. Balke & Kinibel col [PNG106], GBIFCH01223156 (ZSM). ***Paratype*.** • Papua New Guinea, one nymph in ethanol, GBIFCH01523450, same data as holotype (MZL).

##### Nymph.

Body length, female: 10.5 mm.

##### Diagnosis.

Labrum trapezoid; dorsal surface of labrum with distal row multiple; tarsal claw hooked with seven teeth; abdominal tergites predominantly medium to dark brown; central area of upper surface of femora with numerous short, stout, and blunt setae, shorter on hind femur; posterior margin of abdominal segment VII without denticles.

##### Colouration.

**Figure 60.**
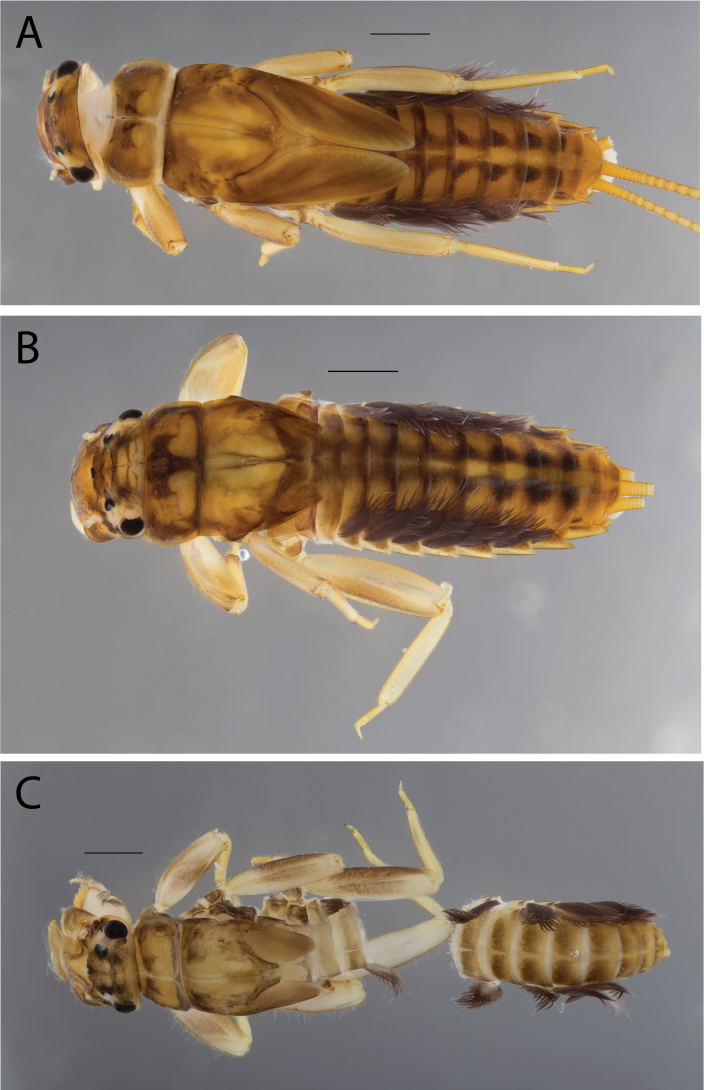
Habitus of *Kosminymphabalkei* sp. nov. (**A**), *Kosminymphakalamorum* sp. nov. (**B**) and *Kosminymphaasarorum* sp. nov. (**C**). Scale bars: 1 mm.

***Head*.** Greyish brown, pale brown between lateral ocelli and compound eyes, pale brown in front of the fore ocellus, clypeus medium brown. Antenna broken. Mouth parts greyish brown. ***Thorax*.** Pronotum pale brown, dark brown laterally and anteriorly, with a double triangular macula along the sagittal line. Mesonotum pale brown, washed with greyish brown, fore wing pads pale brown (Fig. 60C). Femur greyish brown, whitish at base and apex. Tibia uniformly yellowish brown. Tarsi yellowish brown. ***Abdomen*.** Terga greyish brown, posterior margin and sagittal line pale brown. Sterna yellowish. Gills upper lamella blackish, lower lamella greyish. Terminal filaments broken.

##### Description.

***Head*. *Labrum*** trapezoid (Fig. 62A). Ratio labrum width/clypeus width 1.25. Ratio labrum width/insertion width 1.64. Medial emargination shallow with flat denticles. Proximal row of setae present, shifted to distal margin. Number of setae on proximal row ~ 115. Distal row of setae multiple. Median part of labrum in ventral view with scattered thin setae (Fig. 62B). ***Mandibles*.** Outer margin with row of setae on distal 1/2. Ending of the row before incisor. Right mandible with 11 setae below the mola (Fig. 62C). ***Maxillae*.** Posterior margin of cardo with few stout and medium size setae together with some hair-like setae and two or three stout and long setae in submarginal position. Proximo-lateral margin of stipes with a bunch of ~ 15 medium size and stout setae. Apical-ventral row with 36 pectinate setae. Maxillary palp segment II 0.75 as long as segment I. Maxillary palp segment III 0.63 as long as segment I (Fig. 62D). Stout setae on outer margin of segment I absent. Segment II inner margin with four stout setae, outer margin without stout setae. Segment III inner margin with four stout setae, dorsal surface with few setae at apex. Segment III 1.73 × as long as base width. ***Labium*.** Labial palp segment II 0.92 as long as segment I. Labial palp segment III 0.65 as long as segment I (Fig. 62E). Segment I inner margin with 11 stout setae, outer margin with 18 stout setae. Segment II outer margin with ten stout setae. Segment III dorsal face with five stout setae. Segment III 2.77 × as long as base width. ***Hypopharynx*.** Apex of superlingua truncate (Fig. 62F).

***Thorax*.** Dorsal margin of femur with a row of very long, stout, pointed setae (Fig. 63A); an irregular submarginal row of pointed setae as long as or a little bit shorter than those on the dorsal margin, more regular on hind femur; ventral margin with short, stout setae and an irregular submarginal row of long, pointed stout setae, except on hind leg where the submarginal row is regular (Fig. 63B); central area of upper surface with numerous short, stout, blunt setae. Fore tibia with two or three rows of long simple or feathered setae on ventral margin, dorsal margin with numerous hair-like setae; middle tibia with a row of short, simple, pointed setae on ventral margin and a submarginal row of long, simple setae, outer margin with numerous hair-like setae; hind tibia with two or three rows of simple long, stout setae on ventral margin, lower surface with few simple, thin setae, outer margin with short and long pointed stout setae and hair-like setae. Tarsal claw hooked with seven denticles progressively larger apically, except distal much smaller (Fig. 63C).

***Abdomen*.** Gill I with both lamellae. Gills II to VII with dorsal and ventral lamellae of the same size. Gill I with ~ 12 filaments on each lamella, gills II–VI with ~ 20 filaments on each lamella (Fig. 63D), gill VII with ~ ten filaments on each lamella. Filamentous projections on apex. Posterior margin of terga IX with denticles. Posterior margin of terga VIII with minute denticles. Posterior margin of terga V–VII without denticles. Posterolateral projections present on segments VI–IX.

##### Derivatio nominis.

The specific name is to acknowledge the Asaro tribe or “Mudmen” and their living traditions, who inhabit close to where the specimens were collected.

#### ﻿ Kosminympha balkei sp. nov.

Taxon classificationAnimaliaEphemeropteraLeptophlebiidae

2AB4154D-4F43-54D9-B6B4-D837701847FB

https://zoobank.org/5F55D23D-D621-4A8B-B9AE-D5E37EFDED60

Figs 34K , 60A , 65 , 66

##### Material examined.

***Holotype*.** • Papua New Guinea, one nymph on slide, Eastern Highlands Province, Marawaka, Ande, 1700–1800 m, 9.XI.2006, approx. 07°01.697'S, 145°49.807'E, M. Balke & Kinibel col [PNG87], GBIFCH01223158 (ZSM). ***Paratypes*.** • Papua New Guinea, one nymph on slide GBIFCH01223157, 2 nymphs in ethanol, GBIFCH01523449, same data as holotype (MZL).

##### Nymph.

Body length, female: 10.5 mm.

##### Diagnosis.

Labrum cordiform; median part of labrum in ventral view with a row of stout and short setae; abdominal tergites predominantly orange with dark lateral marks; gills II–VI with ~ 15 filaments on each lamella; posterior margin of abdominal segment VII with minute denticles; posterolateral expansions present on abdominal segments V–IX.

##### Colouration.

***Head*.** Medium brown, washed with greyish brown between ocelli, pale brown between lateral ocelli and compound eyes, clypeus medium brown. Antenna uniformly yellowish. Mouth parts brownish. ***Thorax*.** Pronotum medium brown, pale brown laterally, two dark brown arch-like maculae close to the sagittal line. Mesonotum pale brown, medium brown on antero-lateral corners, fore wing pads medium brown (Fig. 60A). Femur uniformly yellowish brown, fore femur with proximal 1/3 whitish. Tibia yellowish orange. Tarsi yellowish orange. ***Abdomen*.** Terga brownish orange, segment II–VII with a pair of more or less triangular dark brown maculae along the sagittal line, segment IX with a pair of square dark brown maculae, segment X entirely brownish orange. Sterna yellowish from segments I–IV, becoming brownish yellow to brownish orange posteriorly. Gills purple. Terminal filaments yellowish, darker near base.

##### Description.

***Head*. *Labrum*** cordiform (Fig. 65A). Ratio labrum width/clypeus width 0.96. Ratio labrum width/insertion width 1.24–1.30. Medial emargination narrow with pointed denticles. Proximal row of setae present, simple. Number of setae on proximal row ~ 82–93. Distal row of setae multiple. Median part of labrum in ventral view with stout setae in row (Fig. 65B). ***Mandibles*.** Outer margin with row of setae on distal 1/2. Ending of the row near incisor. Right mandible with 15–22 setae below the mola (Fig. 65C). ***Maxillae*.** Posterior margin of cardo with many hair-like and long setae, together with two or three long and stout setae in submarginal position. Proximo-lateral margin of stipes with a bunch of ten long hair-like setae (Fig. 34K). Apical-ventral row with 14–15 pectinate setae. Maxillary palp segment II 0.80–0.81 as long as segment I. Maxillary palp segment III 0.57–0.65 as long as segment I (Fig. 65D). Stout setae on outer margin of segment I absent. Segment II inner margin with 1–3 stout setae, outer margin without stout setae. Segment III inner margin with four stout setae, dorsal surface half covered with setae. Segment III 1.53–1.56 × as long as base width. ***Labium*.** Labial palp segment II 0.88 as long as segment I. Labial palp segment III 0.76–0.80 as long as segment I (Fig. 65E). Segment I inner margin with 25–35 stout setae, outer margin with 28–30 stout setae. Segment II outer margin with 12 stout setae. Segment III dorsal face with six or seven stout setae. Segment III 2.53–2.71 × as long as base width. ***Hypopharynx*.** Apex of superlingua emarginated (Fig. 65F).

***Thorax*.** Dorsal margin of femur with a row of very long, thin, pointed setae; irregular submarginal rows of pointed setae as long as or a little bit shorter than those on the dorsal margin, these setae decrease in size from the fore- to the hind femur (Fig. 66A–B); ventral margin with short, stout setae and irregular submarginal rows of long, pointed stout setae, except on hind leg where those on ventral margin are shorter; central area of upper surface with numerous long, stout, pointed setae, shorter on hind femur. Fore tibia with several rows of long simple or feathered setae on ventral margin, dorsal margin with numerous hair-like setae; middle tibia with two rows of long, simple, pointed setae on ventral margin, outer margin with numerous hair-like setae; hind tibia with several rows of simple and feathered long, stout setae on ventral margin, lower surface covered with simple and feathered setae, outer margin with short and long pointed stout setae and hair-like setae. Tarsal claw hooked with eight or nine denticles progressively larger apically (Fig. 66C).

***Abdomen*.** Gill I with both lamellae. Gills II–VII with dorsal and ventral lamellae of the same size. Gill I with ~ 10 filaments on each lamella, gills II–VI with ~ 15 filaments on each lamella, gill VII minute with 5–7 filaments on each lamella. Filamentous projections on apex. Posterior margin of terga IX with denticles. Posterior margin of terga VII–VIII with minute denticles. Posterior margin of terga V–VI without denticles. Posterolateral projections present on segments V–IX.

##### Derivatio nominis.

This species is dedicated to Dr Michael Balke (ZSM, Germany), collector of most of the species treated here.

#### ﻿ Kosminympha baruya sp. nov.

Taxon classificationAnimaliaEphemeropteraLeptophlebiidae

555CC751-CA2D-52E9-B746-4EF76066940C

https://zoobank.org/45A9A359-93B0-40CA-9A59-9299F3393791

**Figure 61.**
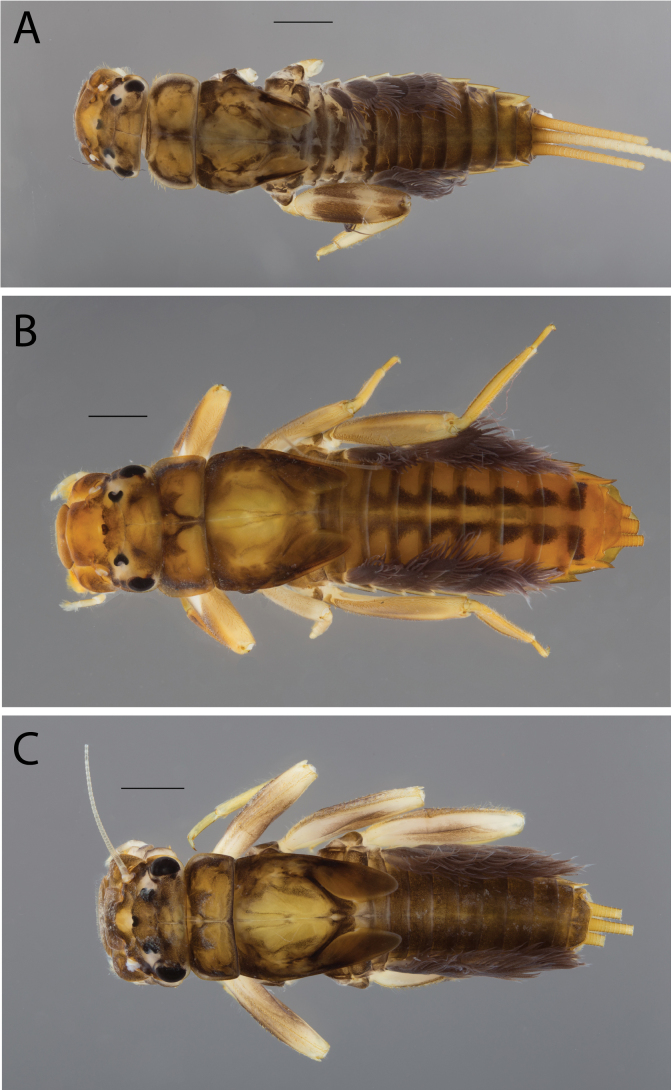
Habitus of *Kosminymphabaruya* sp. nov. (**A**), *Kosminymphasabrinae* sp. nov. (**B**) and *Kosminymphapaulinae* sp. nov. (**C**). Scale bars: 1 mm.

Figs 34L , 61A , 67 , 68

##### Material examined.

***Holotype*.** • Papua New Guinea, nymph on slide, Central Province, Kokoda trek, 1400 m, I.2008, 09°01.952'S, 147°44.455'E, Posman col [PNG172], GBIFCH01223159 (ZSM). ***Paratypes*.** • Papua New Guinea, one nymph in ethanol, GBIFCH01523446, same data as holotype (MZL). • Papua New Guinea, one nymph on slide, GBIFCH01223160, 2 nymphs in ethanol, GBIFCH01523445, Gulf Province, Marawaka, Mala, 1400 m, 11.XI.2006, 07°05.664'S, 145°44.467'E, M. Balke and Kinibel col [PNG90] (MZL).

##### Nymph.

Body length, female: 8.5 mm.

##### Diagnosis.

Labrum cordiform; emargination narrow with pointed denticles; tarsal claw straight with nine or ten teeth; abdominal tergites predominantly medium to dark brown; posterolateral expansions present on abdominal segments VII–IX.

##### Colouration.

***Head*.** Greyish brown, pale brown between antennae and front ocellus. Antenna broken. Mouth parts pale brown, labrum and mandibles dark brown. ***Thorax*.** Pronotum greyish brown, dark brown laterally and medially. Mesonotum pale brown, with greyish maculae as in Fig. 61A. Femur greyish brown, yellowish proximally and subdistally. Tibia uniformly yellowish. Tarsi uniformly yellowish. ***Abdomen*.** Terga uniformly medium brown, with pale brown dots close to gills insertion. Sterna yellowish in median part, posterior margin greyish laterally. Gills brownish purple. Terminal filaments yellowish, darker near base.

**Figure 62.**
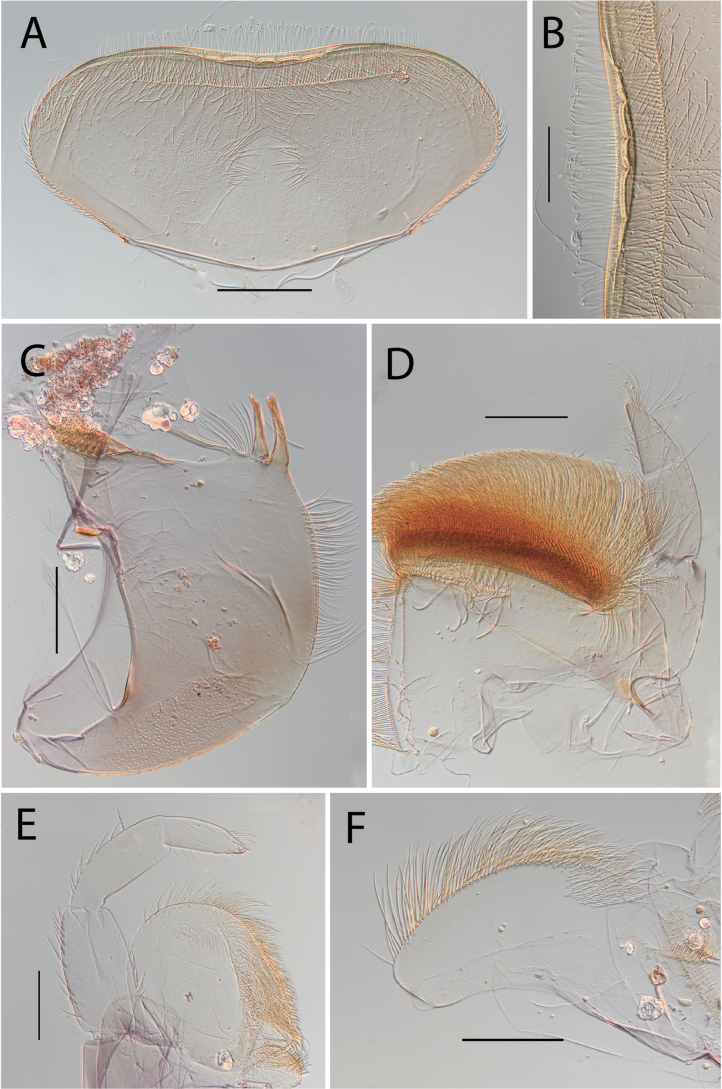
*Kosminymphaasarorum* sp. nov. nymphal mouthparts. **A.** Labrum, dorsal view; **B.** Emargination of the labrum; **C.** Right mandible; **D.** Maxilla; **E.** Labium; **F.** Superlingua of hypopharynx. Scale bars: 200 µm (**A, C–E**); 100 µm (**B**, **F**).

**Figure 63.**
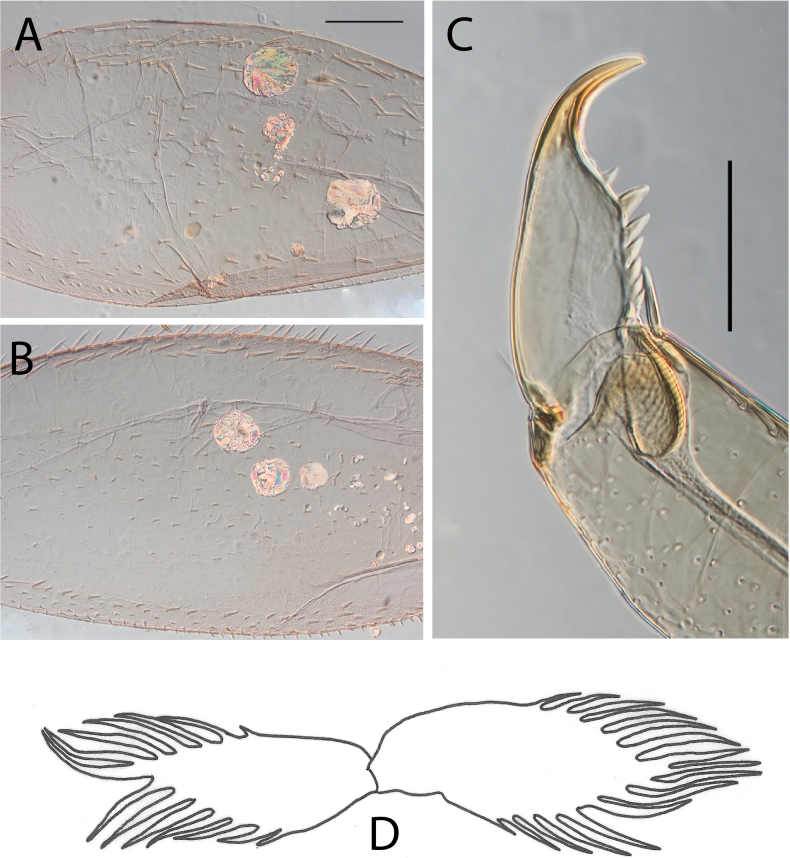
*Kosminymphaasarorum* sp. nov. thorax and abdomen. **A.** Fore femur; **B.** Hind femur; **C.** Claw; **D.** Gill IV. Scale bars: 200 µm (**A, B**); 100 µm (**C**).

##### Description.

***Head*. *Labrum*** cordiform (Fig. 67A). Ratio labrum width/clypeus width 1.06–1.08. Ratio labrum width/insertion width 1.28. Medial emargination narrow with pointed denticles. Proximal row of setae present, simple. Number of setae on proximal row ~ 120–140. Distal row of setae multiple. Median part of labrum in ventral view with scattered thin setae (Fig. 67B). ***Mandibles*.** Outer margin with row of setae on entire margin. Ending of the row near incisor. Right mandible with 14–16 setae below the mola (Fig. 67C). ***Maxillae*.** Posterior margin of cardo with many hair-like setae. Proximo-lateral margin of stipes with a bunch of ~ 15 stout, long, pointed setae (Fig. 34L). Apical-ventral row with 22–24 pectinate setae. Maxillary palp segment II 0.82–0.88 as long as segment I. Maxillary palp segment III 0.75–0.85 as long as segment I (Fig. 67D). Stout setae on outer margin of segment I present. Segment II inner margin with 1–5 stout setae, outer margin with 2–7 stout setae. Segment III inner margin with four or five stout setae, dorsal surface fully covered with setae. Segment III 1.67–1.77 × as long as base width. ***Labium*.** Labial palp segment II 0.77–0.83 as long as segment I. Labial palp segment III 0.60–0.62 as long as segment I (Fig. 67E). Segment I inner margin with 23–25 stout setae, outer margin with 33–38 stout setae. Segment II outer margin with 21–22 stout setae. Segment III dorsal face with three or four stout setae. Segment III 2.56–2.67 × as long as base width. ***Hypopharynx*.** Apex of superlingua emarginated (Fig. 67F).

***Thorax*.** Dorsal margin of femur with a row of very long, thin, pointed setae; irregular submarginal rows of pointed setae as long as or a little bit shorter than those on the dorsal margin, ventral margin with short, stout setae and submarginal rows of long, pointed stout setae, except on hind leg where those on ventral margin are shorter; central area of upper surface with numerous long, stout, pointed setae, shorter on hind femur (Fig. 68A–C). Fore tibia with several rows of long simple or feathered setae on ventral margin, dorsal margin with numerous hair-like setae; middle tibia with several rows of long, simple, pointed setae on ventral margin, outer margin with numerous hair-like setae; hind tibia with several rows of simple and feathered long, stout setae on ventral margin, lower surface covered with simple and feathered setae, outer margin with short and long pointed stout setae and hair-like setae. Tarsal claw hooked with nine or ten denticles progressively larger, except distal much smaller (Fig. 68D).

***Abdomen*.** Gill I with both lamellae. Gills II–VII with dorsal and ventral lamellae of the same size. Gill I with nine or ten filaments on each lamella (Fig. 68J), gills II–VI with ~ 15 filaments on each lamella (Fig. 68L), gill VII with three or four filaments on each lamella (Fig. 68K). Filamentous projections on apex. Posterior margin of terga VII–IX with denticles (Fig. 68G–I). Posterior margin of terga V–VI with minute denticles (Fig. 68E–F). Posterolateral projections present on segment VII–IX.

##### Derivatio nominis.

The specific name is to recognise the tribe Baruya living in the valleys of Wonemara and Marawaka, where some of the specimens come from, and is a noun in apposition.

#### ﻿ Kosminympha kalamorum sp. nov.

Taxon classificationAnimaliaEphemeropteraLeptophlebiidae

9C67D26A-68C3-5566-A388-366F02EE5DE4

https://zoobank.org/98780BA3-8E3F-4683-AA0C-440693701997

Figs 34D , 60B , 69 , 70

##### Material examined.

***Holotype*.** • Papua New Guinea, one nymph on slide, Western Highlands Province, Simbai, 1800–2000 m, 26.II.2007, 05°15.872'S, 144°32.717'E, Kinibel col [PNG134], GBIFCH01223161 (ZSM). ***Paratypes*.** • Papua New Guinea, one nymph on slide, GBICH01223162, 2 nymphs in ethanol, GBIFCH01523447, same data as holotype (MZL). • Papua New Guinea, one nymph on slide, GBIFCH01223163, Eastern Highlands Province, Akameku – Brahmin, Bismarck Range, 2200 m, 23.XI.2006, 05°56.801'S, 145°22.238'E, M. Balke & Kinibel col [PNG106] (MZL).

##### Nymph.

Body length, female: 11.5 mm.

##### Diagnosis.

Labrum trapezoid; median part of labrum in ventral view with scattered thin and long setae; abdominal tergites predominantly orange with dark lateral marks; posterior margin of abdominal segment VII without denticles; posterolateral expansions present on abdominal segments VII–IX.

##### Colouration.

***Head*.** Medium brown, washed with dark brown between ocelli, pale brown between lateral ocelli and compound eyes, clypeus medium brown. Antenna uniformly yellowish. Mouth parts brownish. ***Thorax*.** Pronotum medium brown, dark brown laterally, a large dark brown anchor-like macula straddling the sagittal line. Mesonotum pale brown, medium brown laterally, fore wing pads dark brown (Fig. 60B). Femur: fore femur yellowish brown, with proximal 1/3 whitish, mid- and hind femur pale brown. Tibia yellowish brown, apex of fore tibia dark brown. Tarsi pale brown. ***Abdomen*.** Terga brownish orange, segment II–V with a pair of elongated dark brown maculae along the sagittal line, segments VI–IX with a pair of triangular to square dark brown maculae, segment IX with two small dark stripes along the lateral margins, segment X entirely brownish orange. Sterna yellowish from segments I–IV, becoming brownish yellow to brownish orange posteriorly. Gills purple. Terminal filaments yellowish.

##### Description.

***Head*. *Labrum*** trapezoid (Fig. 69A). Ratio labrum width/clypeus width 1.26–1.27. Ratio labrum width/insertion width 1.69–1.71. Medial emargination shallow with flat denticles. Proximal row of setae simple, shifted to distal margin. Number of setae on proximal row ~ 123–130. Distal row of setae multiple. Median part of labrum in ventral view with scattered thin setae (Fig. 69B). ***Mandibles*.** Outer margin with row of setae on distal 1/2. Ending of the row before incisor. Right mandible with 8–13 setae below the mola (Fig. 69C). ***Maxillae*.** Posterior margin of cardo with few stout and medium size setae (Fig. 34D). Proximo-lateral margin of stipes with a bunch of eight long and stout setae. Apical-ventral row with 34–44 pectinate setae. Maxillary palp segment II 0.77–0.86 as long as segment I. Maxillary palp segment III 0.77–0.86 as long as segment I (Fig. 69D). Stout setae on outer margin of segment I absent. Segment II inner margin with two stout setae, outer margin without or with up to two stout setae. Segment III inner margin with six stout setae, dorsal surface with few setae at apex. Segment III 1.67–2.00 × as long as base width. ***Labium*.** Labial palp segment II 0.84–0.87 as long as segment I. Labial palp segment III 0.60–0.64 as long as segment I (Fig. 69E). Segment I inner margin with 8–10 stout setae. Segment I outer margin with 12–16 stout setae. Segment II outer margin with 6–10 stout setae. Segment III dorsal face without stout setae. Segment III 2.56–3.33 × as long as base width. ***Hypopharynx*.** Apex of superlingua truncate (Fig. 69F).

***Thorax*.** Dorsal margin of fore femur with few stout, short, pointed setae, and a double submarginal row of long, stout, pointed setae (Fig. 70A); dorsal margin of mid- and hind femur with a row of long, thin, pointed setae; a submarginal row of pointed setae as long as or a little bit shorter than those on the dorsal margin (Fig. 70B); ventral margin with short, stout setae and an irregular submarginal row of long, pointed stout setae, except on hind leg where those on ventral margin are shorter; central area of upper surface with numerous short, stout, blunt setae. Fore tibia with two or three rows of long simple or feathered setae on ventral margin, dorsal margin with numerous hair-like setae; middle tibia with one row of short, simple, pointed setae, and a submarginal row of long, simple, pointed stout setae on ventral margin, outer margin with numerous hair-like setae; hind tibia with a row of simple long, stout setae on ventral margin, outer margin with short pointed stout setae and hair-like setae. Tarsal claw hooked with 6–8 denticles progressively larger apically, followed by two tiny protuberances (Fig. 70C).

***Abdomen*.** Gill I with both lamellae. Gills II–VII with dorsal and ventral lamellae of the same size. Gill I with ~ 10 filaments on each lamella, gills II–VI with ~ 15 filaments on each lamella (Fig. 70F), gill VII minute with 5–7 filaments on each lamella. Filamentous projections on apex. Posterior margin of terga IX with minute denticles, or with larger denticles (Fig. 70D). Posterior margin of terga VIII with minute denticles (Fig. 70E). Posterior margin of terga V–VII without denticles. Posterolateral projections present on segments VII–IX.

##### Derivatio nominis.

The specific name is to acknowledge the Kalam tribe of the remote area of Simbai, where the specimens described here come from.

#### ﻿ Kosminympha paulinae sp. nov.

Taxon classificationAnimaliaEphemeropteraLeptophlebiidae

B808E337-80E5-539C-B1EC-90D3B0EA8C7F

https://zoobank.org/B4CB8181-3200-4318-A7DD-C13A07D3E55A

Figs 34J , 61C , 71 , 72

##### Material examined.

***Holotype*.** • Papua New Guinea, nymph on slide, Morobe Province, Menyamya, Mt Inji, 1700 m, 14.XI.2006, approx. 07°14.813'S, 146°01.330'E, M. Balke & Kinibel col [PNG96], GBIFCH01223164 (ZSM). ***Paratype*.** • Papua New Guinea, one nymph in ethanol, GBIFCH01523448, same data as holotype (MZL).

##### Nymph.

Body length, female: 8.2 mm.

##### Diagnosis.

Labrum trapezoid; dorsal surface of labrum with distal row simple; tarsal claw hooked with seven teeth; abdominal tergites predominantly medium to dark brown; central area of upper surface of femora with numerous long, stout, and blunt setae, shorter on hind femur; posterior margin of abdominal segment VII with minute denticles.

##### Colouration.

***Head*.** Greyish brown, pale brown in front of the fore ocellus, whitish between lateral ocelli and compound eyes. Antenna whitish, scape and pedicel greyish white. Mouth parts greyish. ***Thorax*.** Pronotum medium brown, dark brown laterally and anteriorly as in Fig. 61C. Mesonotum medium brown bordered with dark brown, fore wing pads dark brown. Femur medium brown, except proximal part whitish. Tibia uniformly yellowish to pale brown. Tarsi yellowish to pale brown. ***Abdomen*.** Terga uniformly dark brown, sagittal line lighter. Sterna yellowish, segments VIII–IX darker. Gills greyish purple. Terminal filaments yellowish brown.

##### Description.

***Head*. *Labrum*** trapezoid (Fig. 71A). Ratio labrum width/clypeus width 1.28. Ratio labrum width/insertion width 1.71. Medial emargination shallow with flat denticles. Proximal row of setae simple, shifted to anterior margin. Number of setae on proximal row ~ 120. Distal row of setae multiple, shifted to anterior margin. Median part of labrum in ventral view with scattered thin setae (Fig. 71B). ***Mandibles*.** Outer margin with row of setae on distal 1/2. Ending of the row before incisor. Right mandible with 5 setae below the mola (Fig. 71C). ***Maxillae*.** Posterior margin of cardo with ~ 10 stout pointed and long setae. Proximo-lateral margin of stipes with a bunch of ~ 12 medium size and short stout setae (Fig. 34J). Apical-ventral row with 45 pectinate setae. Maxillary palp segment II 0.86 as long as segment I. Maxillary palp segment III 0.67 as long as segment I (Fig. 71D). Stout setae on outer margin of segment I absent. Segment II inner margin without stout setae. Segment II outer margin without stout setae. Segment III inner margin with six stout setae, dorsal surface with few setae at apex. Segment III 1.87 × as long as base width. ***Labium*.** Labial palp segment II 0.75 as long as segment I. Labial palp segment III 0.59 as long as segment I (Fig. 71E). Segment I inner margin with ten stout setae. Segment I outer margin with 17 stout setae. Segment II outer margin with ten stout setae. Segment III dorsal face with two stout setae. Segment III 2.71 × as long as base width. ***Hypopharynx*.** Apex of superlingua truncate (Fig. 71F).

***Thorax*.** Dorsal margin of femur with a row of long, stout, pointed setae; one submarginal row of pointed setae as long as those on the dorsal margin (Fig. 72A), ventral margin with short, stout setae and a submarginal row of pointed stout setae, except on hind leg where those on ventral margin are shorter; central area of upper surface with numerous long, stout, blunt setae, shorter on hind femur. Fore tibia with several rows of long simple or feathered setae on ventral margin, dorsal margin with numerous hair-like setae; middle tibia with a row of short, pointed setae and a submarginal row of long, pointed stout setae on ventral margin, outer margin with numerous hair-like setae; hind tibia with a row of short, pointed setae and a submarginal row of long, pointed simple stout setae on ventral margin, lower surface covered with simple, thin setae, outer margin with short and long pointed stout setae and numerous hair-like setae. Tarsal claw hooked with seven subequal denticles, except median much larger (Fig. 72B–C).

***Abdomen*.** Gill I with both lamellae. Gills II–VII with dorsal and ventral lamellae of the same size, or with ventral lamella smaller. Gill I with ~ 15 filaments on each lamella, gills II–VI with ~ 15 filaments on upper lamella, and ~ 10 on lower lamella, gill VII with ~ 15 filaments on each lamella. Filamentous projections on apex, or along entire margin. Posterior margin of terga IX with denticles. Posterior margin of terga VII–VIII with minute denticles. Posterior margin of terga V–VI without denticles. Posterolateral projections present on segments VI–IX.

##### Derivatio nominis.

This species is dedicated to Pauline Sartori, daughter of the first author.

#### ﻿ Kosminympha sabrinae sp. nov.

Taxon classificationAnimaliaEphemeropteraLeptophlebiidae

DF5F44A6-02E7-5406-A87B-76F073855BFF

https://zoobank.org/2FDD3AD1-27F3-45D6-9FA9-1640AC5C38FB

**Figure 64.**
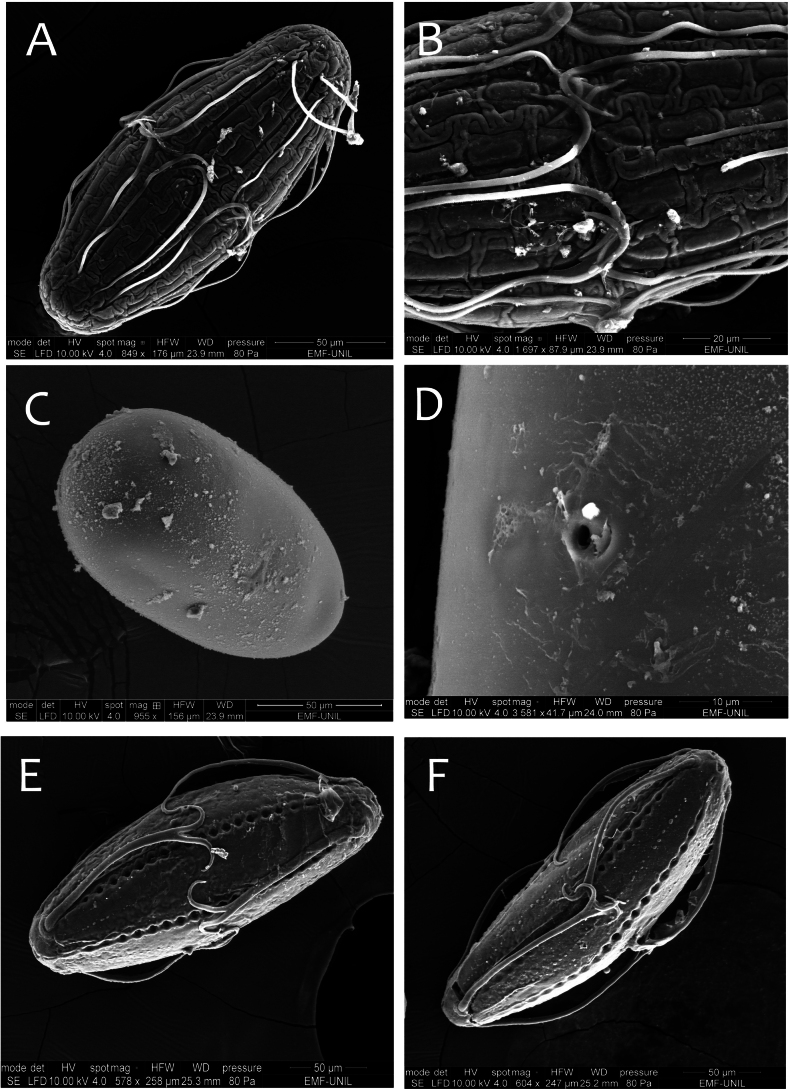
Egg structure in toto (**A, C, E, F**), detail of chorion and micropyle (**B, D**). **A, B.**Thraulus (Masharikella) samueli sp. nov.; **C, D.***Nonnullidensfuyugensis* sp. nov.; **E, F.***Kosminymphasabrinae* sp. nov.

Figs 61B , 64E, F , 73 , 74

##### Material examined.

***Holotype*.** • Papua New Guinea, nymph on slide, Gulf Province, Marawaka, Mala, 1400 m, 11.XI.2006, 07°05.664'S, 145°44.467'E, M. Balke and Kinibel col [PNG90], GBIFCH01223165 (ZSM). ***Paratypes*.** • Papua New Guinea, one nymph in ethanol (UFVB), one nymph on slide, GBIFCH01223166, 3 nymphs in ethanol, GBIFCH01523451, same data as holotype (MZL). • Papua New Guinea, one nymph on slide, GBIFCH01223167, one nymph in ethanol, GBIFCH01523455, Central Province, Kokoda Trek, 1400 m, I. 2008, 09°14.339'S, 147°40.538'E, Posman col [PNG171] (MZL). • Papua New Guinea, 2 nymphs on slide, GBIFCH01223168, GBIFCH01223169, 3 nymphs in ethanol, GBIFCH01523452, Central Province, Kokoda Trek, 1390 m, I. 2008, 09°00.338'S, 147°44.252'E. Posman col [PNG173] (MZL). • Papua New Guinea, 3 nymphs on slide GBIFCH01223170, GBIFCH01223171, GBIFCH01223172, 4 nymphs in ethanol, GBIFCH01523454, Western Highlands Province, Kundum, 1400 m, 3.III.2007, 05°16.096'S, 144°27.869'E, Kinibel col [PNG142] (MZL). • Papua New Guinea, 3 nymphs on slide, GBIFCH01223173, GBIFCH01223174, GBIFCH01223175, one nymph in ethanol, GBIFCH01523453, Madang Province, Simbai area, 1200 m, 10.III.2007, 05°13.389'S, 144°37.285'E, Kinibel col [PNG152] (MZL). • Papua New Guinea, one nymph on slide, GBIFCH01223176, Madang Province, Simbai area, 1200 m, 11.III.2007, 05°13.333'S, 144°37.611'E, Kinibel col [PNG153] (MZL). • Papua New Guinea, one nymph on slide, GBIFCH01223177, Western Highlands Province, Simbai, 1800–2000 m, 26.II.2007, 05°15.872'S, 144°32.717'E, Kinibel col [PNG134] (MZL).

##### Nymph.

Body length, male: 10.5 mm Body length, female: 12.5 mm.

##### Diagnosis.

Labrum cordiform; median part of labrum in ventral view with a row of stout and short setae; abdominal tergites predominantly orange with dark lateral marks; gills II–VI with ~ 25 filaments on each lamella; posterior margin of abdominal segment VII with minute denticles; posterolateral expansions present on abdominal segments VI–IX.

##### Colouration.

***Head*.** Medium brown, washed with greyish brown between ocelli, pale brown between lateral ocelli and compound eyes, clypeus brownish orange. Antenna uniformly yellowish. Compound eye lower portion black, upper portion brownish orange. Mouth parts brownish orange. ***Thorax*.** Pronotum pale brown, lateral margin dark brown, as well as a characteristic double arrow-like macula in the medium pointing towards the head. Mesonotum pale brown bordered with dark brown margins; forewing pads uniformly dark brown. Femur uniformly pale brown, fore femur with proximal 1/3 whitish. Tibia pale to medium brown. Tarsi medium brown. ***Abdomen*.** Terga uniformly brownish orange, segment II–VII with a pair of more or less triangular dark brown maculae along the sagittal line, segment IX with a pair of rectangular dark brown maculae, segment X entirely brownish orange (Fig. 61B). Sterna yellowish from segments I–IV, becoming brownish yellow to brownish orange posteriorly. Gills greyish purple. Terminal filaments uniformly yellowish orange.

##### Description.

***Head*. *Labrum*** cordiform (Fig. 73A). Ratio labrum width/clypeus width 0.96–1.02. Ratio labrum width/insertion width 1.19–1.26. Medial emargination narrow with pointed denticles. Proximal row of setae present, simple. Number of setae on proximal row ~ 88–96. Distal row of setae multiple. Median part of labrum in ventral view with stout setae in row (Fig. 73B). ***Mandibles*.** Outer margin with row of setae on distal 1/2. Ending of the row near incisor. Right mandible with 12–15 setae below the mola (Fig. 73C). ***Maxillae*.** Posterior margin of cardo with many long, stout and pointed setae together with some hair-like setae. Proximo-lateral margin of stipes with a bunch of ~ 10 long hair-like setae. Apical-ventral row with 12–15 pectinate setae. Maxillary palp segment II 0.86–1.00 as long as segment I (Fig. 73D). Maxillary palp segment III 0.67–0.78 as long as segment I. Stout setae on outer margin of segment I absent. Segment II inner margin with one or two stout setae, outer margin with 3–5 stout setae. Segment III inner margin with 3–5 stout setae, dorsal surface half covered with setae. Segment III 1.53–2.40 × as long as base width. ***Labium*.** Labial palp segment II 0.75–0.85 as long as segment I. Labial palp segment III 0.67–0.83 as long as segment I (Fig. 73E). Segment I inner margin with 26–30 stout setae, outer margin with 24–30 stout setae. Segment II outer margin with 12–22 stout setae. Segment III dorsal face with 5–10 stout setae. Segment III 2.56–3.00 × as long as base width. ***Hypopharynx*.** Apex of superlingua emarginated (Fig. 73F).

**Figure 65.**
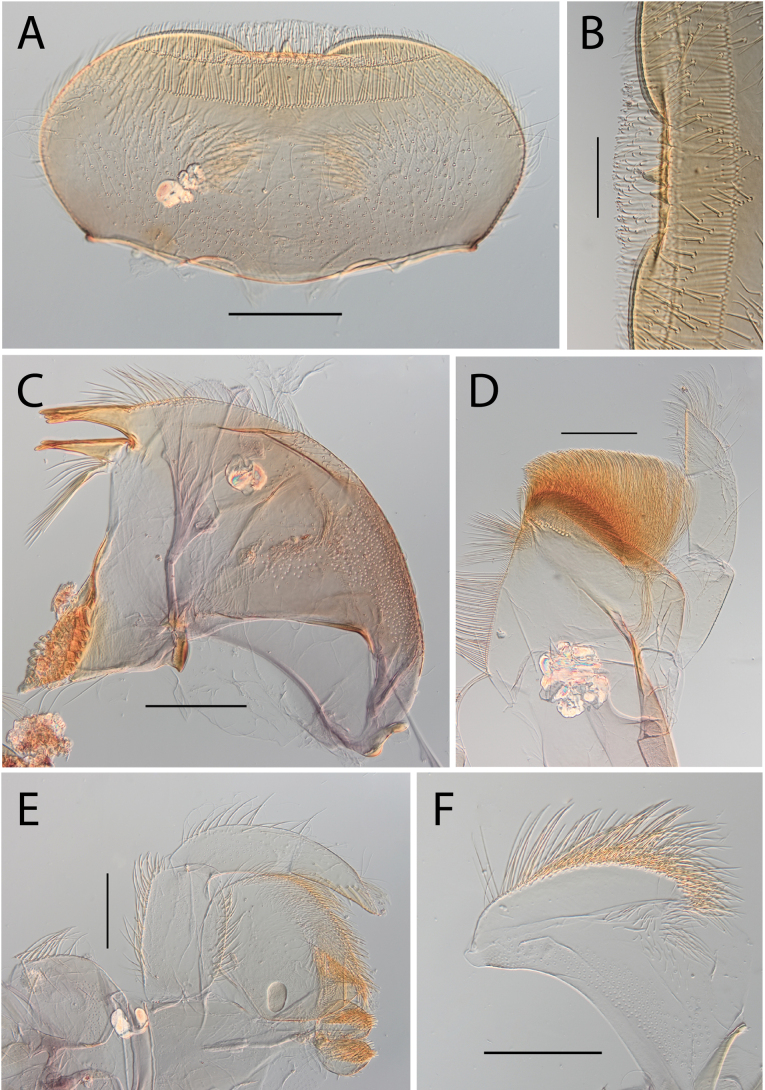
*Kosminymphabalkei* sp. nov. nymphal mouthparts. **A.** Labrum, dorsal view; **B.** Emargination of the labrum; **C.** Right mandible; **D.** Maxilla; **E.** Labium; **F.** Superlingua of hypopharynx. Scale bars: 200 µm (**A, C–F**); 100 µm (**B**).

**Figure 66.**
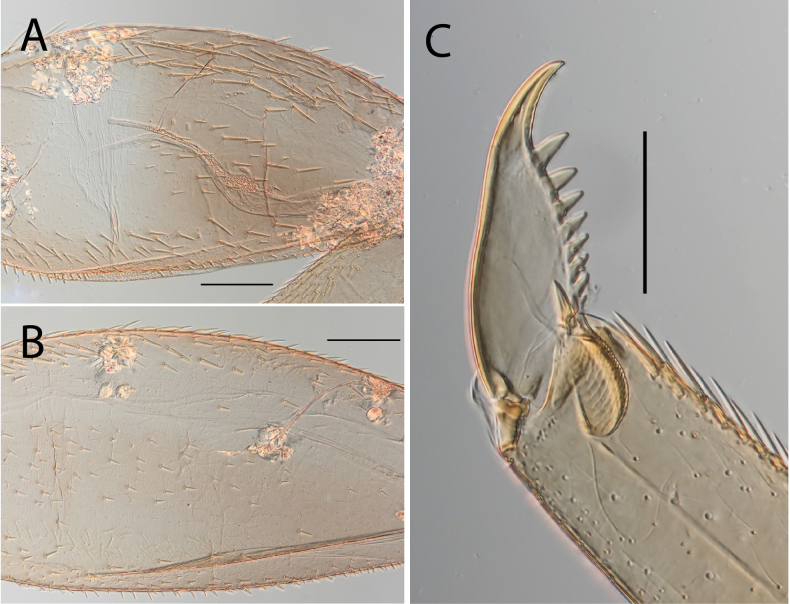
*Kosminymphabalkei* sp. nov. thorax. **A.** Fore femur; **B.** Hind femur; **C.** Claw. Scale bars: 200 µm (**A, B**); 100 µm (**C**).

**Figure 67.**
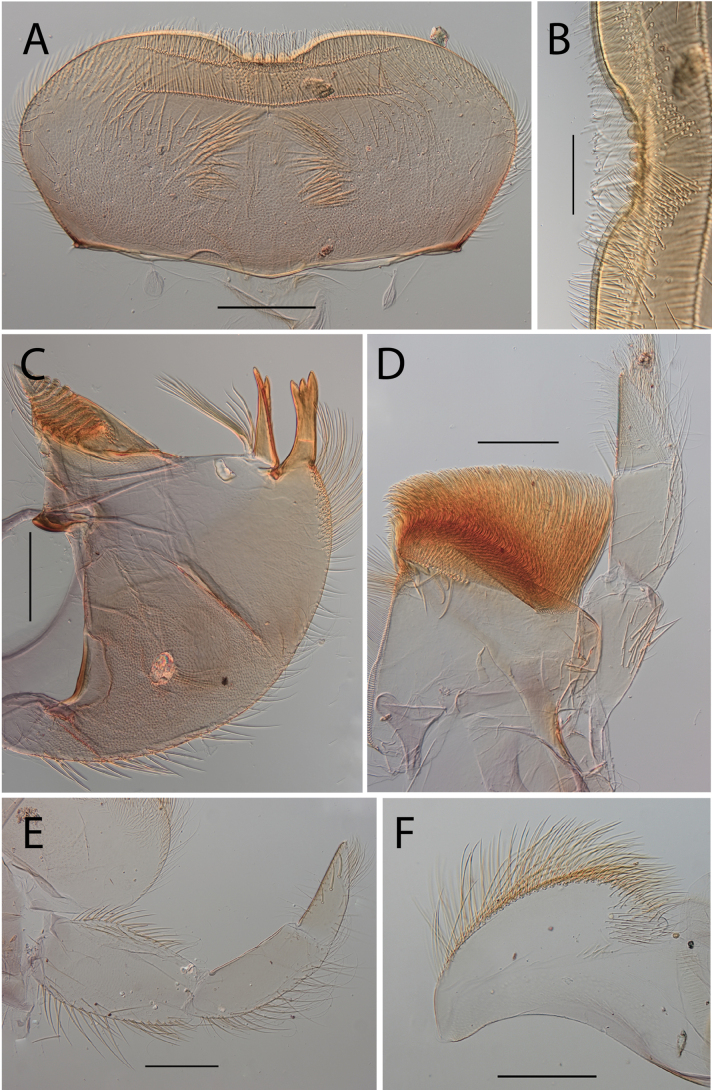
*Kosminymphabaruya* sp. nov. nymphal mouthparts. **A.** Labrum, dorsal view; **B.** Emargination of the labrum; **C.** Right mandible; **D.** Maxilla; **E.** Labium; **F.** Superlingua of hypopharynx. Scale bars: 200 µm (**A, C–F**); 100 µm (**B**).

**Figure 68.**
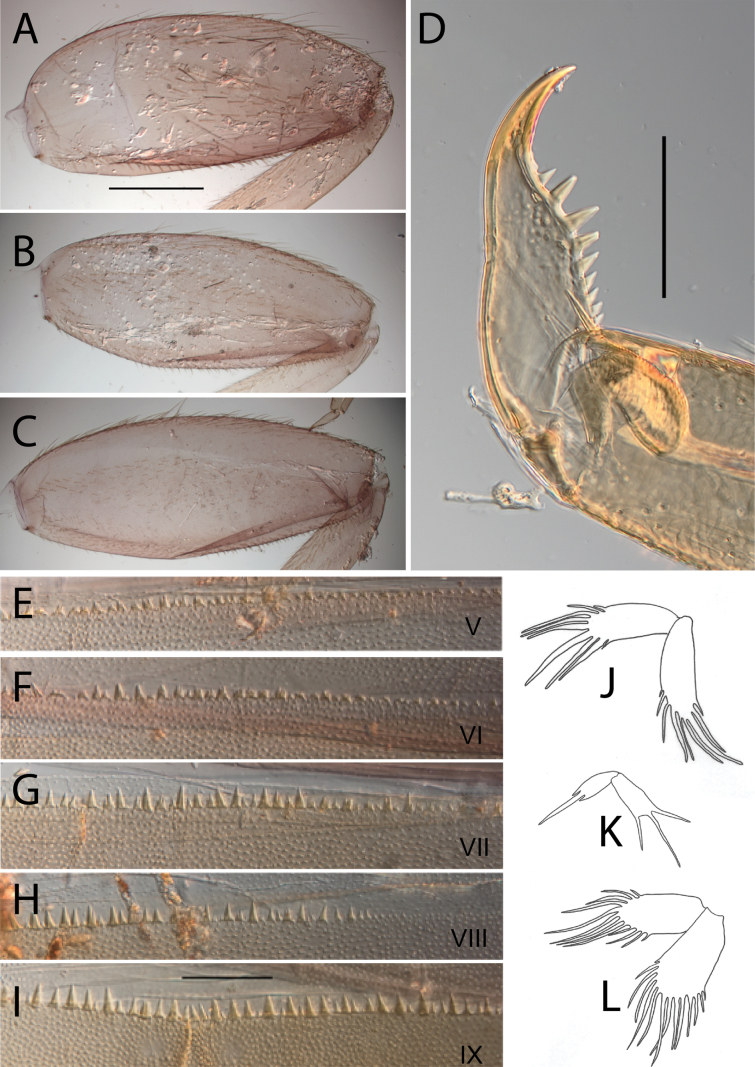
*Kosminymphabaruya* sp. nov. thorax and abdomen. **A.** Fore femur; **B.** Mid femur; **C.** Hind femur; **D.** Claw; **E.** Posterior margin of tergite V; **F.** Posterior margin of tergite VI; **G.** Posterior margin of tergite VII; **H.** Posterior margin of tergite VIII; **I.** Posterior margin of tergite IX; **J.** Gill I; **K.** Gill VII; **L.** Gill IV. Scale bars: 500 µm (**A–C**); 100 µm (**D–I**).

**Figure 69.**
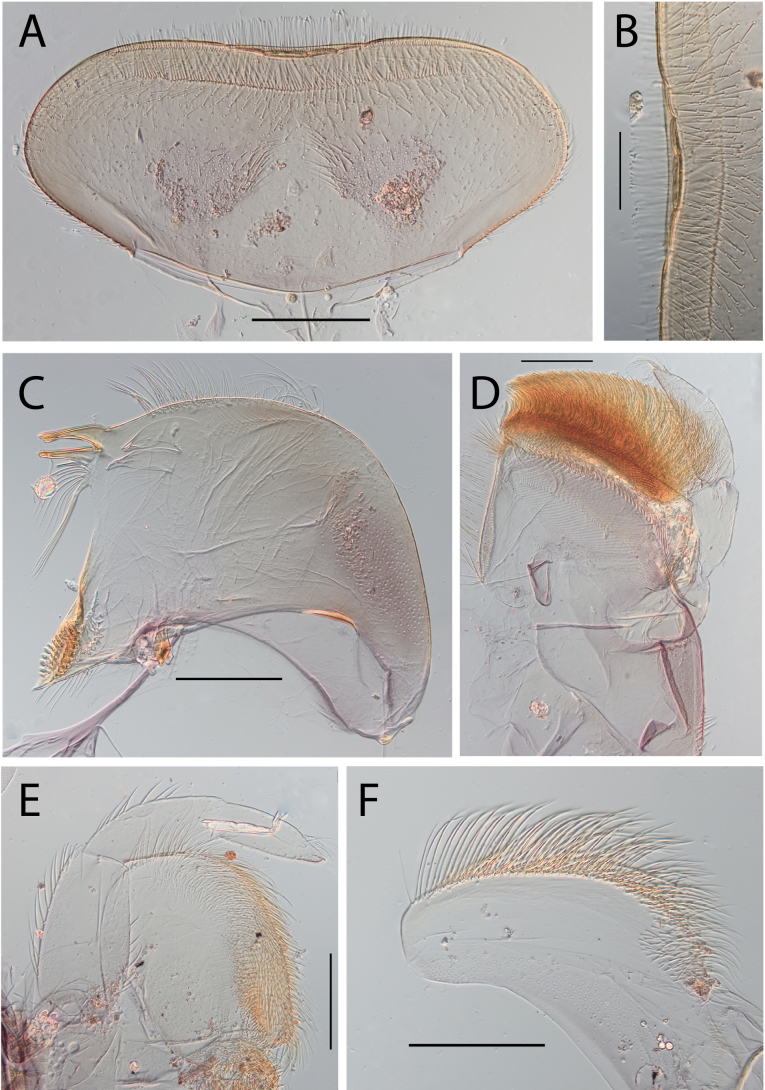
*Kosminymphakalamorum* sp. nov. nymphal mouthparts. **A.** Labrum, dorsal view; **B.** Emargination of the labrum; **C.** Right mandible; **D.** Maxilla; **E.** Labium; **F.** Superlingua of hypopharynx. Scale bars: 200 µm (**A–F**); 100 µm (**B**).

**Figure 70.**
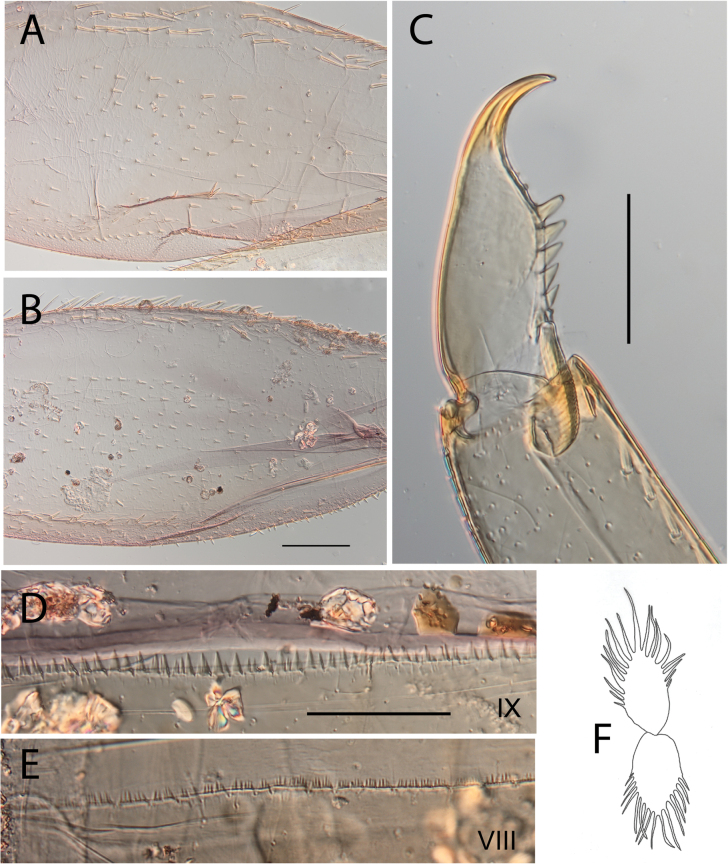
*Kosminymphakalamorum* sp. nov. thorax and abdomen. **A.** Fore femur; **B.** Hind femur; **C.** Claw; **D.** Posterior margin of tergite IX; **E.** Posterior margin of tergite VIII; **F.** Gill IV. Scale bars: 200 µm (**A, B**); 100 µm (**C–E**).

**Figure 71.**
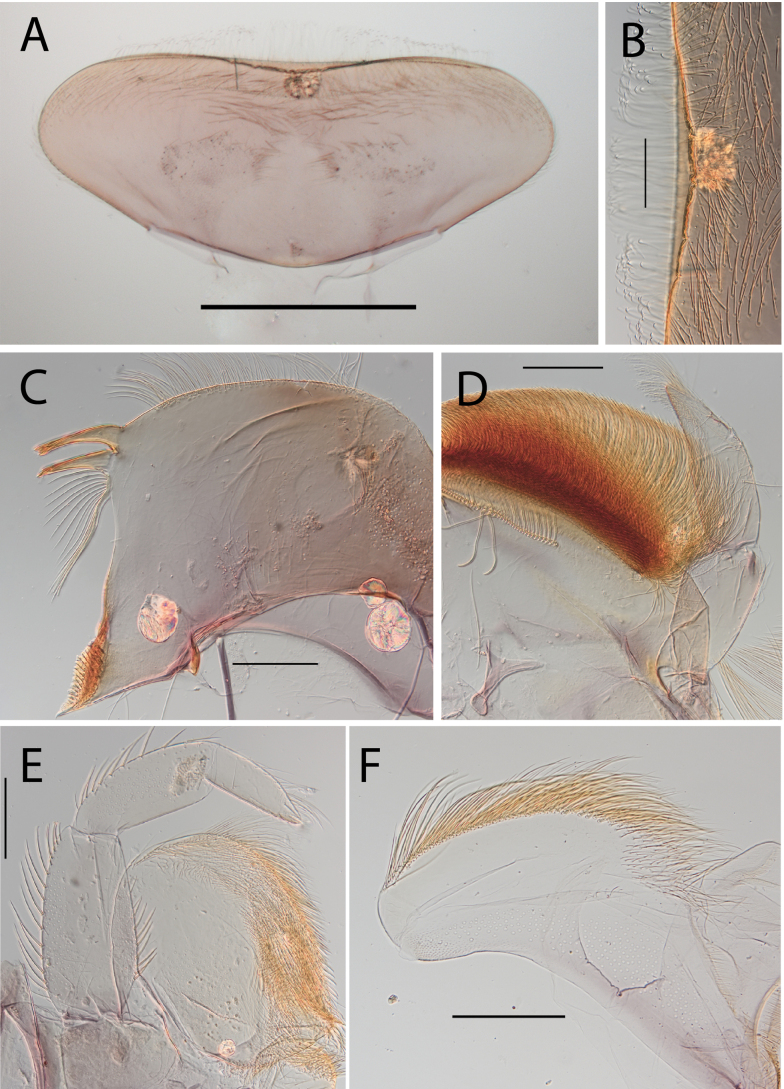
*Kosminymphapaulinae* sp. nov. nymphal mouthparts. **A.** Labrum, dorsal view; **B.** Emargination of the labrum; **C.** Right mandible; **D.** Maxilla; **E.** Labium; **F.** Superlingua of hypopharynx. Scale bars: 200 µm (**A, C–F**); 100 µm (**B**).

**Figure 72.**
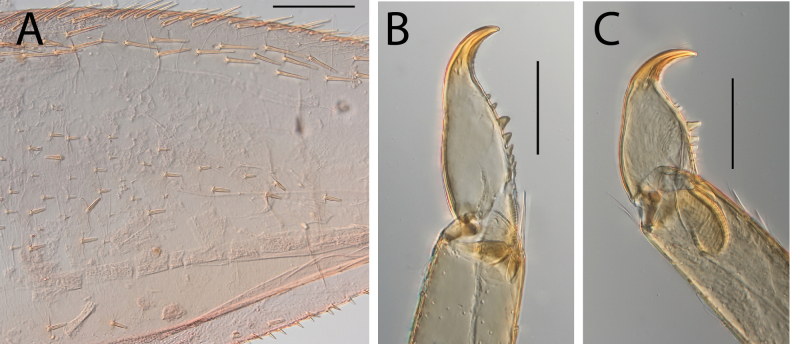
*Kosminymphapaulinae* sp. nov. thorax. **A.** Mid femur; **B.** Fore claw; **C.** Hind claw Scale bars: 200 µm (**A**); 100 µm (**B, C**).

**Figure 73.**
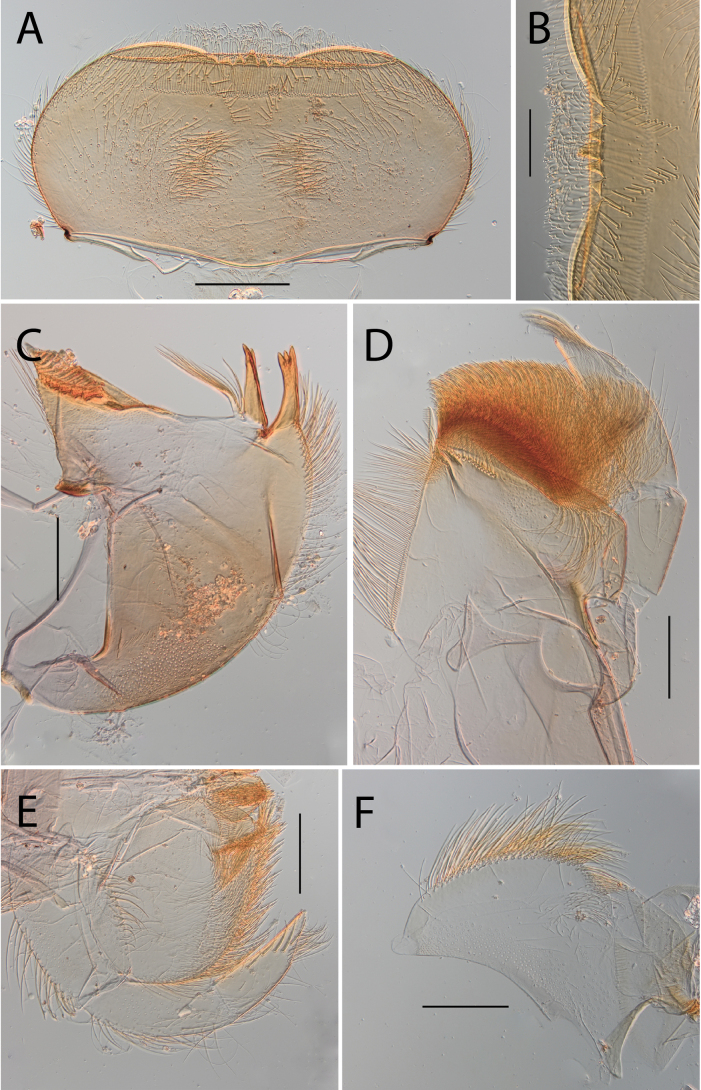
*Kosminymphasabrinae* sp. nov. nymphal mouthparts. **A.** Labrum, dorsal view; **B.** Emargination of the labrum; **C.** Right mandible; **D.** Maxilla; **E.** Labium; **F.** Superlingua of hypopharynx. Scale bars: 200 µm (**A–F**); 100 µm (**B**).

**Figure 74.**
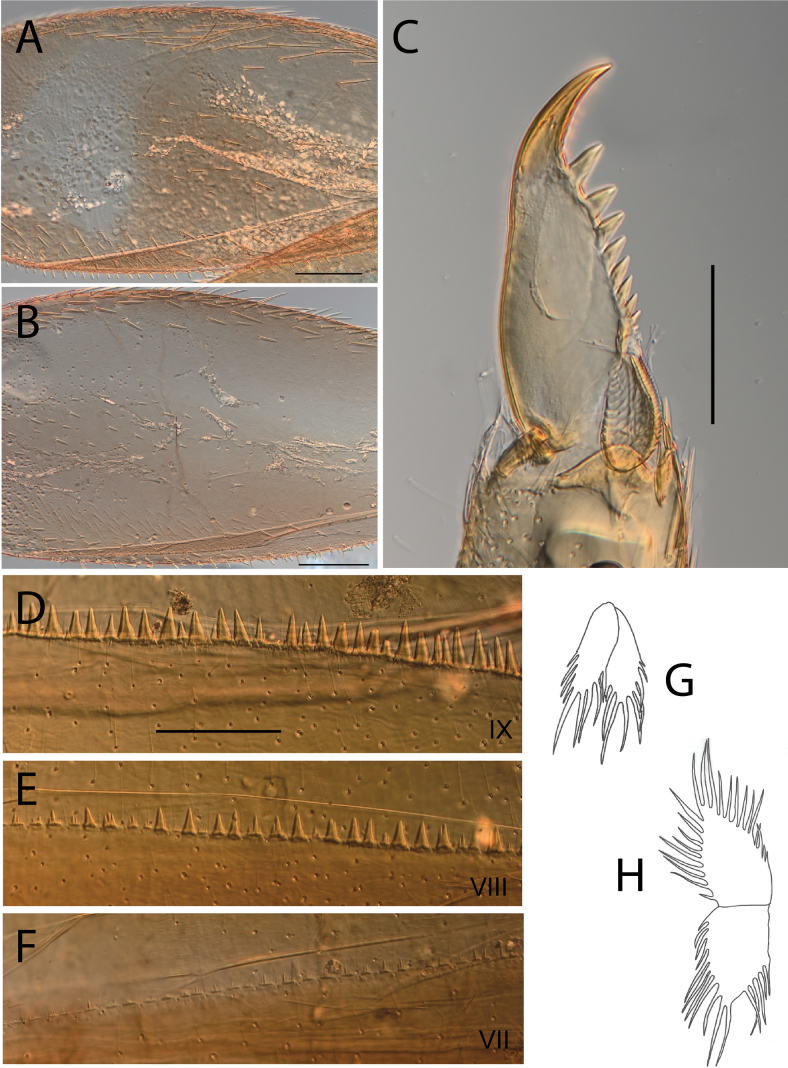
*Kosminymphasabrinae* sp. nov. thorax and abdomen. **A.** Fore femur; **B.** Hind femur; **C.** Claw; **D.** Posterior margin of tergite IX; **E.** Posterior margin of tergite VIII; **F.** Posterior margin of tergite VII; **G.** Gill I; **H.** Gill IV. Scale bars: 200 µm (**A, B**); 100 µm (**C–F**).

***Thorax*.** Dorsal margin of femur with a row of very long, thin, pointed setae; irregular submarginal rows of pointed setae as long as or a little bit shorter than those on the dorsal margin, ventral margin with short, stout setae and submarginal rows of long, pointed stout setae, except on hind leg where those on ventral margin are shorter (Fig. 74A–B); central area of upper surface with numerous long, stout, pointed setae, shorter on hind femur. Fore tibia with several rows of long simple or feathered setae on ventral margin, dorsal margin with numerous hair-like setae; middle tibia with several rows of long, simple, pointed setae on ventral margin, outer margin with numerous hair-like setae; hind tibia with several rows of simple and feathered long, stout setae on ventral margin, lower surface covered with simple and feathered setae, outer margin with short and long pointed stout setae and hair-like setae. Tarsal claw hooked, with eight to ten denticles progressively larger apically (Fig. 74C).

***Abdomen*.** Gill I with both lamellae. Gills II–VII with dorsal and ventral lamellae of the same size. Gill I with ~ 10 filaments on each lamella (Fig. 74G), gills II–VI with 20–25 filaments on each lamella (Fig. 74H), gill VII with 12–15 filaments on each lamella. Filamentous projections on apex, or along entire margin. Posterior margin of terga IX with denticles (Fig. 74D). Posterior margin of terga VIII with minute denticles, or with larger denticles (Fig. 74E). Posterior margin of terga VII with minute denticles (Fig. 74F). Posterior margin of terga V–VI without denticles. Posterolateral projections present on segment VI–IX.

***Eggs*.** General shape elongated. Size 205–210 μm × 90–95 μm. Chorionic surface smooth with longitudinal ridges well marked having the appearance of being segmented. Attachment structures at each pole composed of four anchors with long stem, reaching the middle of the egg (Fig. 64E–F). Micropyle not visible.

##### Derivatio nominis.

This species is dedicated to Sabrina Perret Sartori, daughter in law of the first author.

### ﻿Differential diagnoses among *Kosminympha* species

The identification of the six species of this new genus is rather straight forward using the key proposed. Major characters rely on the labrum shape (trapezoidal in three species and cordiform in three others), colouration of the abdomen (predominantly orange in three species, and dark brown in three others), posterolateral projections of the abdomen and number of filaments on gills. Without any doubt, other species are awaiting to be discovered, so a careful comparison of all characters is needed to confirm identification obtained with the key.

### ﻿Key to the nymphs of Leptophlebiidae from New Guinea and neighbouring islands

We present here a key for the nymphs of all known species, with the exception of *Magnilobuspacificola* (Demoulin, 1969) which is only known from a single female subimago coming from Manus Island, Bismarck Archipelago, and included in the tribe Thraulini (*Thraulus* lineage) by Grant and Peters (1993).

**Table:** 

1	Gill I different in shape from the subsequent ones (e.g., Fig. 2)	**2**
–	Gill I similar in shape as the subsequent ones (e.g., Fig. 35)	**15**
2	Abdominal gill I with 2 slender and very long lamellae (Fig. 13F)	**3 (Thraulus (Thraulus))**
–	Abdominal gill I with dorsal lamella slender and ventral lamella fimbriate (Fig. 32E)	**11** (**Thraulus (Masharikella))**
3	Labrum rectangular (Figs 3A, 12A); maxillary palp much longer than galea-lacinia; posterolateral projections of the abdomen present on segments VIII–IX only	**4**
–	Labrum cordiform (Fig. 9A) or trapezoidal (Fig. 21A); maxillary palp a little bit longer than galea-lacinia; posterolateral projections of the abdomen present on segments VI–IX or VII–IX, rarely on VIII–IX only	**5**
4	Labrum emargination shallow (Fig. 3B); proximo-lateral margin of stipes with a single long and stout seta; outer margin of maxillary palp segment II without stout setae (Fig. 3D); inner margin of labial palp segment I with 16–18 stout setae (Fig. 3E); apex of superlingua truncate; gills II–VI completely fringed with filaments, with ~ 30 filaments on dorsal lamella and ~ 40 on ventral lamella (Fig. 4E); posterior margin of abdominal segment VIII without denticles	**Thraulus (Thraulus) eloisae sp. nov.**
–	Labrum emargination narrow (Fig. 12B); proximo-lateral margin of stipes with one long and stout seta together with two small and stout setae ; outer margin of maxillary palp segment II with 1–3 stout setae (Fig. 12D); inner margin of labial palp segment I with 20–26 stout setae (Fig. 12E); apex of superlingua rounded (Fig. 12F); gills II–VI without filaments on basal half, with ~ 15 filaments on dorsal lamella and ~ 20 on ventral lamella (Fig. 13G); posterior margin of abdominal segment VIII with minute denticles (Fig. 13E)	**Thraulus (Thraulus) noe sp. nov.**
5	Labrum trapezoidal (Fig. 21A); ventral side of the labrum with only scattered stout setae (Fig. 21B); labial palp segment III > 4 × as long as base width (Fig. 21E); posterior margin of abdominal segment VI–VII with well-developed denticles (Fig. 23C, D)	**Thraulus (Thraulus) sp. A**
–	Labrum cordiform (e.g., Figs 5A, 18A); ventral side of the labrum with stout setae in a row (e.g., Figs 14B, 16B); labial palp with segment III < 3.5 × as long as base width (Figs 5E, 14E, 18E); posterior margin of abdominal segment VI–VII without or with minute denticles	**6**
6	Gill lamellae on segments II–VI with < 20 filaments on each (Figs 10D, 19G)	**7**
–	Gill lamellae on segments II–VI with > 25 filaments on each (Figs 6F, 8C, 17D)	**9**
7	Proximal row of setae on dorsal face of labrum with 19–23 setae (Fig. 9A); apical ventral row of maxilla composed of 15–17 pectinate setae (Fig. 9D); tarsal claw with 8 or 9 teeth, distal one much larger (Fig. 10C)	**Thraulus (Thraulus) nabire sp. nov**.
–	Proximal row of setae on dorsal face of labrum with > 30 setae (e.g., Figs 5A, 16A); apical ventral row of maxilla composed of > 18 pectinate setae (e.g., 5D, 18D); tarsal claw with 4–7 teeth (Fig. 8B), distal one equal in size to the others	**8**
8	Outer margin of mandibles with a row of setae on distal 2/3 (Fig. 18C); posterior margin of cardo with many hair-like setae and one stout and long seta in submarginal position; inner margin of maxillary palp segment III with 4–6 stout setae (Fig. 18D); posterior margin of abdominal segment VII with minute denticles (as in Fig. 19E); posterolateral projections of the abdomen present on segments VI–IX	**Thraulus (Thraulus) wemale sp. nov.**
–	Outer margin of mandibles with a tuft of setae in medium and in distal position (Fig. 14C); posterior margin of cardo with few hair-like setae; inner margin of maxillary palp segment III with 3 stout setae (Fig. 14D); posterior margin of abdominal segment VII without denticles; posterolateral projections of the abdomen present on segments VII–IX	**Thraulus (Thraulus) ogea sp. nov.**
9	Dorsal face of labrum with multiple rows in distal position (Fig. 16C); claw hooked with 15 teeth and a palisade of 4 other teeth in distal position (Fig. 17C); posterolateral projections of the abdomen present on segments VIII and IX only	**Thraulus (Thraulus) timorensis sp. nov.**
–	Dorsal face of labrum with a simple row in distal position; claw straight with 6–10 teeth; posterolateral projections of the abdomen present on segments VII–IX	**10**
10	Apical ventral row of maxilla composed of 19–23 pectinate setae (Fig. 5D); outer margin of maxillary palp segment II with 3–5 stout setae; outer margin of labial palp segment I with 15–20 stout setae (Fig. 5E); tarsal claw with 7–10 teeth (Fig. 6C)	**Thraulus (Thraulus) granti sp. nov.**
.–	Apical ventral row of maxilla composed of 17 pectinate setae (Fig. 7D); outer margin of maxillary palp segment II without setae; outer margin of labial palp segment I with < 10 stout setae (Fig. 7E); tarsal claw with 6 teeth (Fig. 8B)	**Thraulus (Thraulus) longinquus sp. nov.**
11	Tarsal claw straight, without teeth (Fig. 32C); apical ventral row of maxilla composed of 16–20 pectinate setae (Fig. 31D); posterior margin of abdominal segment IX with minute denticles (Fig. 32D); posterolateral projections of the abdomen present on segments VIII–IX only	**Thraulus (Masharikella) samueli sp. nov.**
–	Tarsal claw hooked, always with teeth; apical ventral row of maxilla composed of 25–35 pectinate setae; posterior margin of abdominal segment IX with well-developed denticles; posterolateral projections of the abdomen present on segments VI–IX or VII–IX	**12**
12	Outer margin of maxillary palp II with 5 stout setae (Fig. 29D); outer margin of labial palp segment II with 5 stout setae (Fig. 29E); tarsal claw with 6 or 7 teeth progressively larger (Fig. 30C); posterolateral projections of the abdomen present on segments VI–IX	**Thraulus (Masharikella) pascalae sp. nov.**
–	Outer margin of maxillary palp II with at most 4 stout setae; outer margin of labial palp segment II with at most 4 stout setae; tarsal claw with less or more than 6 or 7 teeth subequal or with the last one much larger; posterolateral projections of the abdomen present on segments VII–IX	**13**
13	Labrum width equal to clypeus width; proximal row of setae on dorsal face of labrum with > 35 setae (Fig. 27A); maxillary palp segment III < 1.5 × as long as base width (Fig. 27D); tarsal claw with 4 teeth, distal one much larger than the others (Fig. 28B); posterior margin of abdominal segments VII and VIII without denticles	**Thraulus (Masharikella) johannisluci sp. nov.**
–	Labrum width smaller than clypeus width; proximal row of setae on dorsal face of labrum with < 30 setae; maxillary palp segment III 1.7–2.4 × as long as base width; posterior margin of abdominal segments VII and VIII at least with minute denticles	**14**
14	Ventral side of labrum with stout setae in a row (Fig. 25B); posterior margin of cardo with many hair-like setae and one stout and long seta in submarginal position (Fig. 34B); proximo-lateral margin of stipes with a single stout and medium size seta; maxillary palp segment III 1.8–2.4 × as long as base width (Fig. 25D); labial palp segment III 3.5–3.8 × as long as base width (Fig. 25E); posterior margin of abdominal segment VIII with well-developed denticles (Fig. 26E)	**Thraulus (Masharikella) iteris sp. nov.**
–	Ventral side of labrum with scattered stout setae (Fig. 33B); posterior margin of cardo with few hair-like setae; proximo-lateral margin of stipes with two long and two short stout setae; maxillary palp segment III ca 1.7 × as long as base width (Fig. 33D); labial palp segment III ~ 3.0 × as long as base width (Fig. 33E); posterior margin of abdominal segment VIII with minute denticles	**Thraulus (Masharikella) sp. B**
15	Ventral lamella of gills highly reduced or absent, ventral lamella of first gill with < 8 filaments, even absent in some species (e.g., Fig. 48E–G); claw with generally 3–6 teeth, rarely up to 8 (e.g., 44C); posterior margin of abdominal segment VIII generally without denticles; posterolateral expansions on abdominal segments VIII–IX, rarely VII–IX	**16 (*Nonnullidens*)**
–	Ventral lamella of gills smaller but not highly reduced, with ≥ 10 filaments (e.g., Fig. 63D); claws with 7–10 teeth (e.g., Fig. 74C); posterior margin of abdominal segment VIII with small denticles (e.g., Fig. 74E); posterolateral expansions on abdominal segments VII–IX, VI–IX or even V–IX	**33 (*Kosminympha***)
16	Middle and hind femora pale brown with two transverse dark brown bands, in middle and at apex (e.g., Fig. 57B)	**17**
–	Middle and hind femora uniformly dark brown, sometimes lighter near margins, but never with transverse dark bands (e.g., Fig. 53B)	**27**
17	Fore femora without banding, but with a diffuse brown macula	**18**
–	Fore femora banded as mid- and hindlegs	**19**
18	Labrum with shallow anterior emargination; apical ventral row of maxilla with 27–30 pectinate setae; claw with 4 denticles	***Nonnullidensbillhilli* Grant & Peters, 1993**
–	Labrum with deep anterior emargination (Fig. 47A, B); apical ventral row of maxilla with 23–26 pectinate setae; claw with 3 denticles	***Nonnullidenskaltenbachi* sp. nov.**
19	Shape of the labrum trapezoidal (e.g., Fig. 56A)	**20**
–	Shape of the labrum cordiform (e.g., Fig. 47A)	**22**
20	Apical ventral row of maxilla composed of > 35 pectinate setae	***Nonnullidenshsui* (Peters & Tsui, 1972)**
–	Apical ventral row of maxilla composed of ≤ 32 pectinate setae	**21**
21	Dorsal face of the labrum with simple row in distal position (Fig. 56B)	***Nonnullidens* sp. A**
–	Dorsal face of the labrum with multiple rows in distal position (Fig. 58B)	***Nonnullidens* sp. B**
22	Gills II–IV without ventral lamellae	***Nonnullidensdepapricus* Kluge, 2013**
–	Gills II–IV with ventral lamella bearing ≥ 3 filaments	**23**
23	Dorsal face of the labrum with proximal and distal rows multiple (Fig. 39A)	**24**
–	Dorsal face of the labrum with proximal and distal row simple (e.g., Fig. 49A)	**25**
24	Gill I with ventral lamella composed of 2–4 filaments; gills II–VII with 8–11 filaments on dorsal lamella and 6–7 filaments on ventral lamella	***Nonnullidensvariegatus* Kluge, 2013**
–	Gill I with ventral lamella composed of a single filament (Fig. 40E); gill II–VI with 6 or 7 filaments on dorsal lamella and 2 or 3 on ventral lamella (Fig. 40F); gill VII with 4 or 5 filaments on dorsal lamella and a single one on ventral lamella (Fig. 40G)	***Nonnullidensanga* sp. nov.**
25	Posterolateral expansions present on abdominal segments VIII–IX	***Nonnullidensmarcelae* sp. nov.**
–	Posterolateral expansions present on abdominal segments VII–IX	**26**
26	Median emargination of labrum narrow with rounded denticles; ventral surface of labrum with scattered thin setae; apical ventral surface of maxilla with 25–27 pectinate setae; posterior margin of abdominal segments V–VIII with well-developed denticles (Fig. 51E–G)	***Nonnullidensmariae* (Peters & Tsui, 1972)**
–	Median emargination of labrum shallow with flat denticles; ventral surface of labrum with stout setae in row (Fig. 54B); posterior margin of abdominal segments V–VIII without or with minute denticles	***Nonnullidenssilvaepumilorum* sp. nov.**
27	Gill I with ventral lamella composed of at least 1 filament (Fig. 44E)	**28**
–	Gill I without ventral lamella, uni-lamellate (e.g., Fig. 46D)	**29**
28	Central area of upper surface of femora with small spine-like setae; gills II–VII with ventral lamella with 5–7 filaments; posterolateral expansions present on abdominal segments VIII and IX	***Nonnullidensniger* Kluge, 2013**
–	Central area of upper surface of femora with numerous long and short, stout and pointed setae (Fig. 44A); gills II–VI with ventral lamella with 3 or 4 filaments (Fig. 44F), gill VII with 2–4 filaments on each lamella (Fig. 44G); posterolateral expansions present on abdominal segments VII–IX	***Nonnullidenscozzarolae* sp. nov.**
29	Gill II with ventral lamella composed of 2–4 filaments (Fig. 46E)	**30**
–	Gill II without ventral lamella, uni-lamellate (e.g., Fig. 38F)	**31**
30	Dorsal surface of segment III of labial palp with 1–3 stout setae; ventral margin of femora with sparse very small spine-like setae; tarsal claw strongly hooked, most prominent tooth in middle of claw	***Nonnullidensreductus* Kluge, 2013**
–	Dorsal surface of segment III of labial palp with 3–4 stout setae (Fig. 45E); ventral margin of femora with short and stout setae and an irregular submarginal row of long and pointed stout setae (Fig. 46A); tarsal claw moderately hooked, most prominent tooth in apical 2/3 of claw (Fig. 46B)	***Nonnullidensfuyugensis* sp. nov.**
31	All gills uni-lamellate, lacking ventral lamella (Fig. 53D–F); claw with only 3 teeth (Fig. 53C)	***Nonnullidensmoiorum* sp. nov.**
–	Gills III–VII bilamellate; claw with 6–8 teeth	**32**
32	Proximo-lateral margin of stipes with a bunch of ~ 5 stout setae of irregular length; outer margin of maxillary palp segment II without stout setae (Fig. 41D); gills III–VI with 5 or 6 filaments on dorsal lamella and 1 or 2 filaments on ventral lamella (Fig. 42F), gill VII with 3 filaments on dorsal lamella and a single filament on ventral lamella (Fig. 42G)	***Nonnullidensboonsoongi* sp. nov.**
–	Proximo-lateral margin of stipes with a bunch of ~ 6 stout setae increasing in size distally; outer margin of maxillary palp segment II with 1–4 stout setae (Fig. 37D); gills III–VI with 5 to 8 filaments on dorsal lamella and 4 or 5 on ventral lamella (Fig. 38G), gill VII with 4 or 5 filaments on dorsal lamella and 1 or 2 filaments on ventral lamella (Fig. 38H)	***Nonnullidensalvarezi* sp. nov.**
33	Abdominal tergites predominantly orange with dark lateral marks, triangular or rectangular (e.g., Fig. 60A)	**34**
–	Abdominal tergites predominantly medium to dark brown (e.g., Fig. 61C)	**36**
34	Labrum trapezoid (Fig. 69A); median part of labrum in ventral view with scattered thin and long setae (Fig. 69B); apical ventral surface of maxilla with 30–45 pectinate setae (Fig. 69D); posterior margin of abdominal segment VII without denticles; posterolateral expansions present on abdominal segments VII–IX	***Kosminymphakalamorum* sp. nov.**
–	Labrum cordiform (e.g., Fig. 73A); median part of labrum in ventral view with a row of stout and short setae (e.g., Fig. 73B); apical ventral surface of maxilla with < 20 pectinate setae; posterior margin of abdominal segment VII with minute denticles (Fig. 74F); posterolateral expansions present on abdominal segments V–IX or VI–IX	**35**
35	Gills II–VI with 20–25 filaments on each lamella (Fig. 74H), gill VII with 12–15 filaments on each lamella; posterolateral expansions present on abdominal segments VI–IX	***Kosminymphasabrinae* sp. nov.**
–	Gills II–VI with ~ 15 filaments on each lamella, gill VII minute with 5–7 filaments on each lamella; posterolateral expansions present on abdominal segments V–IX	***Kosminymphabalkei* sp. nov.**
36	Labrum cordiform (Fig. 67A); labrum emargination narrow with pointed denticles (Fig. 67B); apical ventral surface of maxilla with 22–24 pectinate setae (Fig. 67D); tarsal claw with 9 or 10 teeth (Fig. 68D); posterolateral expansions present on abdominal segments VII–IX	***Kosminymphabaruya* sp. nov.**
–	Labrum trapezoid (e.g., Fig. 71A); labrum emargination shallow with flat denticles (e.g., Fig. 71B); apical ventral surface of maxilla with > 30 pectinate setae (e.g., Fig. 62D); tarsal claw hooked with 7 teeth (e.g., Fig. 63C); posterolateral expansions present on abdominal segments VI–IX	**37**
37	Dorsal surface of labrum with distal row simple (Fig. 71A); right mandible with ~ 5 setae below the mola (Fig. 71C); posterior margin of cardo with ~ 10 stout pointed and long setae; dorsal surface of labial palp segment III with 2 stout setae (Fig. 71E); central area of upper surface of femora with numerous long, stout and blunt setae (Fig. 72A); posterior margin of abdominal segment VII with minute denticles	***Kosminymphapaulinae* sp. nov**.
–	Dorsal surface of labrum with distal row multiple (Fig. 62B); right mandible with ~ 11 setae below the mola (Fig. 62C); posterior margin of cardo with few stout and medium size setae together with some hair-like setae and 2 or 3 stout and long setae in submarginal position; dorsal surface of labial palp segment III with 5 stout setae; central area of upper surface of femora with numerous short, stout and blunt setae (Fig. 63B); posterior margin of abdominal segment VII without denticles	***Kosminymphaasarorum* sp. nov.**

## ﻿Discussion

### ﻿Correlation between genetics and morphology

In Cozzarolo et al. (2019), bPTP analysis suggested the presence of 52 MOTUs, including one from Sumatra and another one from Borneo which are not considered here. Of these 50 putative species, ten were excluded from the morphological analysis because of degraded or too young specimens. On the other hand, we treat here three species for which no genetic sequences were obtained. These are *Kosminymphapaulinae*, *Nonnullidensanga*, and *N.silvaepumilorum*. Consequently, 28 species of 31 described here possess at least one genetic sequence identification; Table 1 presents a summary of this information. For 12 species, we have a single sequence, which matches each a single OTU. For the 16 remaining species, they can be split into three categories.

- Strict concordance between morphology and genetic data. This concerns eight species for which we have 2–4 genetic sequences matching the same morphological species. All belong to the genus *Thraulus* sensu lato, except the species *N.kaltenbachi*. It is interesting to note that the later species is the only one for which two haplotypes coming from different localities perfectly match the same species. *Nonnullidenskaltenbachi* is the only lowland species of the genus found in the New Guinea North Coast ecoregion, suggesting that geneflow is still active between these localities.
- Concordance between related MOTUs and morphological species. Four species are concerned here. Generally, two sister MOTUs are morphologically identical, such as in *Nonnullidensmoiorum* (sp. 38 – sp. 39), *Kosminymphakalamorum* (sp. 41 – sp. 42), or Thraulus (Thraulus) granti (sp. 3 – sp. 4); in one case three related OTUs are morphologically identical as in Thraulus (Thraulus) wemale (sp. 6 – sp. 7 – sp. 8), endemic to the island of Makalu Seram. In this case, it is probable that the bPTP model is too sensitive and has over-split the taxa, because of missing haplotypes in between.
- None or low concordance between MOTUs and morphological species. This is the case for the following four species *Kosminymphasabrinae*, *Nonnullidenscozzarolae*, *N.boonsoongi*, and *N.alvarezi*. These species are among the most widespread in New Guinea, so probably exhibiting the highest genetic diversity. But we cannot exclude that each of them is a complex of cryptic species we currently are not able to separate morphologically. *Kosminymphasabrinae* is interesting because it is composed of related MOTUs (sp. 15 – sp. 16 – sp. 17), and a more distant one (sp. 20), suggesting that at least sp. 20 could be a cryptic species. In *N.alvarezi* and *N.boonsoongi*, we find two pairs of OTUs (sp. 30 – sp. 31 and sp. 36 – sp. 37) mixing both species. As shown in the key, both species are close together morphologically, so it is possible that a revision of the species will be necessary. In the clade containing *N.cozzarolae*, some OTUs are mixed between *N.boonsoongi* and *N.alvarezi* (sp. 27, sp. 36, sp. 37), giving more arguments that this complex of species needs further study.

In summary, our morphological analysis is more conservative from a taxonomic point of view than the genetic MOTU’s. But in some cases, this may be due to species on their way of differentiation and not distinguishable based on morphology.

### ﻿Biogeographical considerations

**Figure 75.**
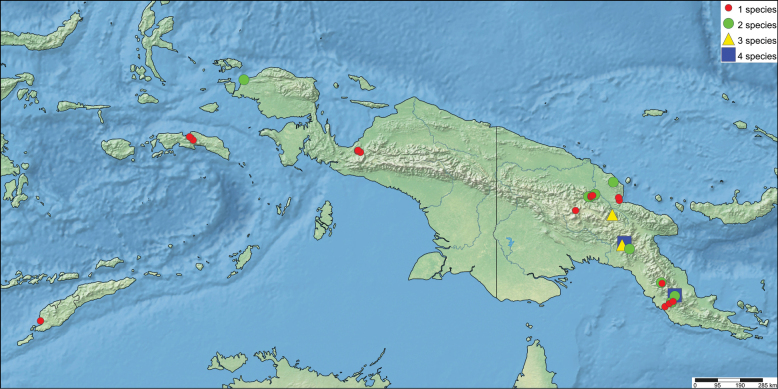
Distribution of localities with number of species found at each.

Of the 33 sampling localities where Leptophlebiidae have been identified, 20 (60%) were inhabited by a single species, nine (28%) by two species, and four (12%) with three and four species (Fig. 75). Among the last category, one locality is situated in the Eastern Highlands Province, between, 1700–1800 m (PNG87) and the other one in Central Province, on Kokoda Trek, at an altitude of ca 1400 m (PNG173). Among those with three recorded species, one locality is situated in the Eastern Highlands Province in the Bismarck Range, ca 2200 m (PNG106) and the other one in the Gulf Province, at 1400 m (PNG90). For instance, in PNG87 can be found together with Thraulus (Thraulus) eloisae, Th. (Th.) noe, *Kosminymphabalkei*, and *Nonnullidenscozzarolae*, while in PNG173, Thraulus (Thraulus) granti, Th. (Th.) noe, *Kosminymphasabrinae*, and *Nonnullidenscozzarolae* are sympatric.

All these high-altitude localities are located in the Papuan Peninsula or at the junction of the Central Mountain Range, the Southwest New Guinea –Trans-Fly lowland and the New Guinean North Coast (Fig. 1). Interestingly, Cozzarolo et al. (2019) estimated that the *Thraulus* lineage colonised the Papuan Peninsula secondarily from already existing terranes, likely corresponding to the New Guinean North Coast and the Southwest New Guinea –Trans-Fly lowland. The speciation events occurring through dispersal or vicariance during mainly the Eocene–Oligocene, followed by speciation occurring in sympatry during the Pliocene–Pleistocene may explain why these altitude areas are the richest among encountered Leptophlebiidae.

**Figure 76.**
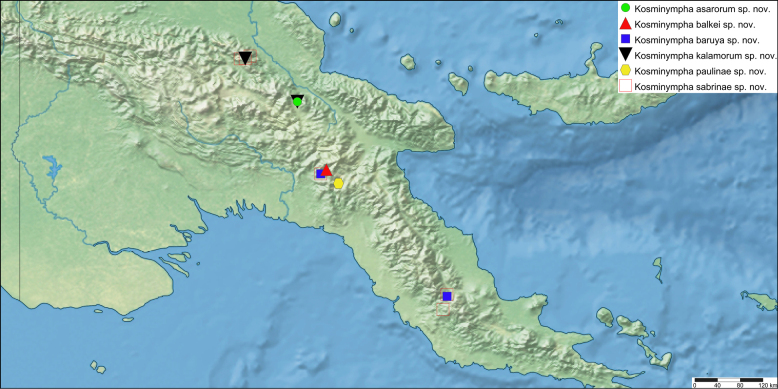
Distribution of *Kosminympha* species in the area.

Species of the genus *Kosminympha* are only reported from Eastern New Guinea and at altitudes ranging from 1200 to 2200 m (Fig. 76). This suggests that the Papuan Peninsula Orogen which occurs in early mid-Oligocene (30–35 Ma) (van Ufford and Cloos 2005) was a major trigger leading to the establishment of a derived lineage confined to highlands still nowadays. This is in accordance with the chronogram proposed by Cozzarolo et al. (2019) which indicate that *Kosminympha* arose in mid-Oligocene.

**Figure 77.**
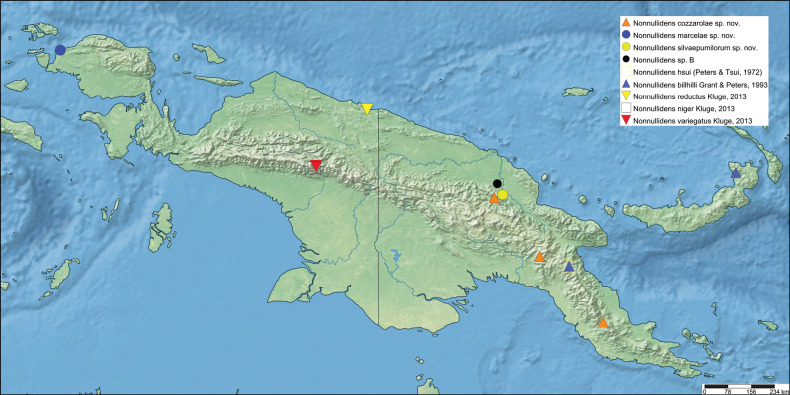
Distribution of *Nonnullidens* species in the area.

**Figure 78.**
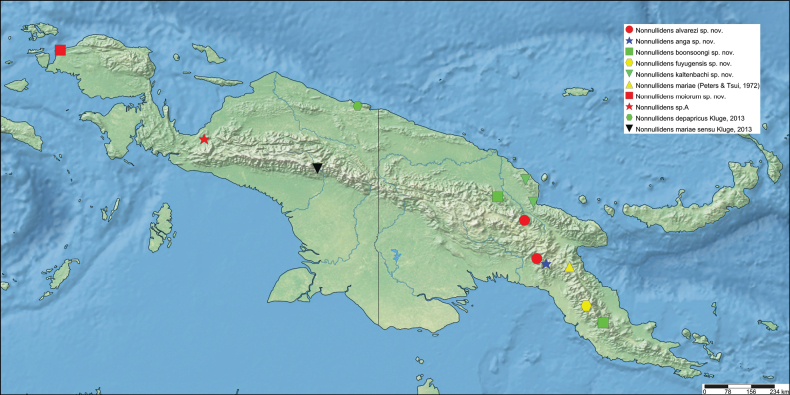
Distribution of *Nonnullidens* species in the area.

*Nonnullidens* is the most diversified genus in New Guinea. Following Cozzarolo et al. (2019) it may have arisen in early Miocene, probably in Southwest New Guinea–Trans fly lowland, then spread to Papuan Peninsula, North Coast and Vogelkopf–Bomberai. Most species are to be found in montane and highland streams, exhibiting a similar pattern than *Kosminympha*. Only four species have been found below 500 m, in the North Coast and Vogelkopf (Figs 77, 78).

**Figure 79.**
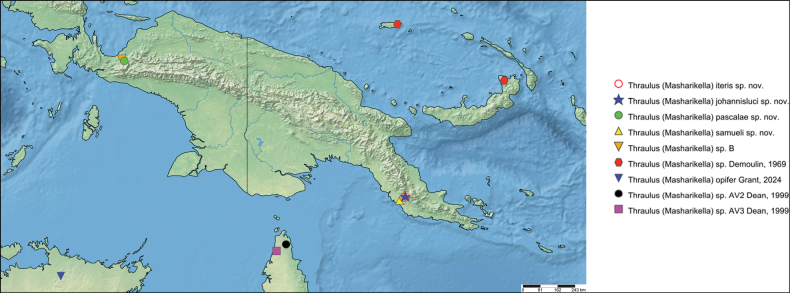
Distribution of Thraulus (Masharikella) species in the area.

Species belonging to Thraulus (Masharikella) were always recorded alone, without any other Leptophlebiidae, except for one locality (PNG169) where two species co-occur, Thraulus (Masharikella) iteris and Th. (M.) johannisluci. Noteworthy is that these localities are located at low altitude (below 500 m) except one in middle altitude (ca 900 m). Species of Thraulus (Masharikella) have never been reported from Southeast Asia, whereas six species are recorded from New Guinea, one from New Britain and Manus Island (Demoulin 1969), and three species colonise exclusively the north of Australia (Grant 2024, Dean 1999) (Fig. 79). This may indicate a Gondwanan origin of the lineage, currently present in East Africa, southern India, and Australasia, and which could not colonise the Wallacea area.

**Figure 80.**
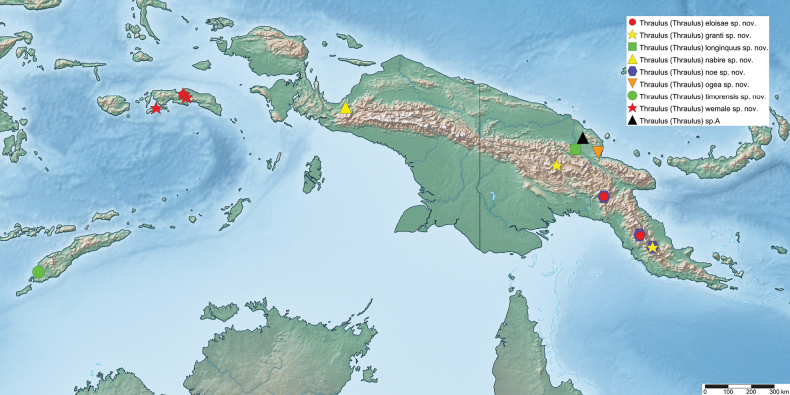
Distribution of Thraulus (Thraulus) species in the area.

In the investigated area, the subgenus Thraulus (Thraulus) is the only one found outside mainland New Guinea, in Maluku Seram and on Timor Island. It is mainly found in lowland streams, but two species reached the highlands. Their phylogenetic positions on the cladogram proposed by Cozzarolo et al. (2019) indicate the occurrence of two independent shifts along the altitudinal gradient. One involves the highland species Thraulus (Thraulus) granti (Central province) which is related to the lowland species Th. (Th.) sp. A (Gulf Province). The other example is Th. (Th.) eloisae, a highland species which sister species is Th. (Th.) noe found at lower elevations in Central and Eastern Highlands Provinces (Fig. 80). Species of Thraulus (Thraulus) are reported from Southeast Asia, but do not seem as diversified as in New Guinea. A single species is reported from Sulawesi and another from Borneo, an undescribed species is mentioned from Sumatra, and two species are reported from Southeast Asia mainland (Grant 2024). As the subgenus is distributed in the Palearctic and Oriental regions, we can suppose a Laurasian origin and a colonisation of New Guinea and surrounding islands from the west.

New Guinea therefore stands at a crossroad between west and east influences. The subgenus Thraulus probably colonised Proto New Guinea in the Oligocene from the west via Sundaland, a large exposed landmass including Borneo, Sumatra, Java, and Peninsular Malaysia during the Pleistocene (Voris 2000). The subgenus Masharikella may have colonised in early Oligocene–late Miocene from the east via Sahul, an emerged landmass including Australia and New Guinea. Interestingly, during the Pleistocene, some river networks were shared between New Guinea and northern Australia, corresponding to the current distribution of the subgenus Masharikella in the area (Voris 2000: fig. 1). The close affinities between Th. (M.) samueli from the Papuan peninsula and Th. (M.) sp. AW3 from northern Queensland brings some support to this hypothesis. In this scenario, the towering orogeny of New Guinea was the driver of the splitting of lowland *Thraulus*-like ancestors into highlands lineages, represented today by *Nonnullidens* and *Kosminympha*, as suggested by Toussaint et al (2014). Some major mayfly families such as Heptageniidae, Ephemerellidae, or even Tricorythidae never reached New Guinea from the west. These conditions allow the diversification of the *Thraulus* lineage in New Guinea, probably by vacant niches due to evolutionary and biogeographical contingencies. Australian Leptophlebiidae are very diverse, but, except for the three Thraulus (Masharikella) species of northern Australia, all belong to the subfamily Atalophlebiinae. This Gondwanan subfamily includes taxa from the Patagonian Shield and Australia (Monjardim et al. 2020), with some lineages reaching the northern tropical areas, such as the genera *Jappa* Harker, 1954, *Atalomicria* Harker, 1954 or *Austrophlebiodes* Campbell & Suter, 1988 (Campbell and Peters 1993; Parnrong and Campbell 1997).

### ﻿Estimation of the biodiversity

Although this contribution greatly improves the Leptophlebiidae biodiversity of New Guinea with more than 30 new species, we think it is just a beginning and many more species await descriptions. Two thirds of the new species indeed are described from a single locality, emphasising once more the importance of micro-endemicity, especially in the highlands. Therefore, we can expect to find new species at each new locality. Based on the coverage by Michael Balke and collaborators, we therefore think the New Guinean leptophlebiid fauna to be approximately two to three times more diverse than currently known. This takes into consideration the ten MOTUs we were not able to study morphologically, which likely represent each a new species. Therefore, we estimate the diversity of New Guinean Leptophlebiidae to be ~ 100 species. Knowing that all these species belong to a single tribe (Thraulini), this makes New Guinea the most diverse area for this lineage in the world, and a true hotspot of biodiversity. This phenomenon is already described for other mayfly species, such as the extraordinary radiation of the genus *Labiobaetis* (Baetidae) (Kaltenbach et al. 2023) with more than 40 species. Even more impressive are the local radiation of the water beetle genus *Exocelina* (Toussaint et al. 2014, 2021) with more than 150 species in the area, or the one of *Trigonopterus* weevils (Coleoptera, Curculionidae) with ~ 500 species in New Guinea and surrounding islands (Letsch et al. 2023).

## Supplementary Material

XML Treatment for Thraulus

XML Treatment for Subgenus Thraulus

XML Treatment for Thraulus (Thraulus) eloisae

XML Treatment for Thraulus (Thraulus) granti

XML Treatment for Thraulus (Thraulus) longinquus

XML Treatment for Thraulus (Thraulus) nabire

XML Treatment for Thraulus (Thraulus) noe

XML Treatment for Thraulus (Thraulus) ogea

XML Treatment for Thraulus (Thraulus) timorensis

XML Treatment for Thraulus (Thraulus) wemale

XML Treatment for Thraulus (Thraulus)

XML Treatment for Subgenus Masharikella

XML Treatment for Thraulus (Masharikella) iteris

XML Treatment for Thraulus (Masharikella) johannisluci

XML Treatment for Thraulus (Masharikella) pascalae

XML Treatment for Thraulus (Masharikella) samueli

XML Treatment for Thraulus (Masharikella)

XML Treatment for Nonnullidens

XML Treatment for Nonnullidens alvarezi

XML Treatment for Nonnullidens anga

XML Treatment for Nonnullidens boonsoongi

XML Treatment for Nonnullidens cozzarolae

XML Treatment for Nonnullidens fuyugensis

XML Treatment for Nonnullidens kaltenbachi

XML Treatment for Nonnullidens marcelae

XML Treatment for Nonnullidens mariae

XML Treatment for Nonnullidens moiorum

XML Treatment for Nonnullidens silvaepumilorum

XML Treatment for Nonnullidens

XML Treatment for Nonnullidens

XML Treatment for Kosminympha

XML Treatment for Kosminympha asarorum

XML Treatment for Kosminympha balkei

XML Treatment for Kosminympha baruya

XML Treatment for Kosminympha kalamorum

XML Treatment for Kosminympha paulinae

XML Treatment for Kosminympha sabrinae

## ﻿Additional information

### Author ORCIDs

Michel Sartori https://orcid.org/0000-0003-3397-3397

Frederico F. Salles https://orcid.org/0000-0001-8331-5929

### Data availability

All of the data that support the findings of this study are available in the main text.
